# Co-delivering macrophage engager mRNA and PD-L1 antibody via tumor-responsive nanoparticles for glioblastoma immunotherapy

**DOI:** 10.1038/s41467-026-71646-y

**Published:** 2026-04-11

**Authors:** Haoge Zhang, Jia Miao, Lin Gao, Xuhong Yang, Zhengcheng Yun, Lei Dong, Wanqing Cheng, Yuqi Wang, Hui Yang, Ying Zhou, Yini Zhu, Jinbing Xie

**Affiliations:** 1 Department of Radiology, Nantong First People’s Hospital, School of Medicine, Southeast University, Nantong, China; 2 Nurturing Center of Jiangsu Province for State Laboratory of AI Imaging & Interventional Radiology, Zhongda Hospital, Southeast University, Nanjing, China; 3 Department of Biochemistry and Molecular Biology, School of Medicine, Southeast University, Nanjing, China; 4 Department of Microbiology and Immunology, School of Medicine, Southeast University, Nanjing, China; 5 Department of Urology, Nantong First People’s Hospital, School of Medicine, Southeast University, Nantong, China

**Keywords:** Cancer microenvironment, CNS cancer, DNA and RNA, Nanoparticles, Immunotherapy

## Abstract

Bispecific immune cell engagers, particularly bispecific T-cell engagers, show limited efficacy in solid tumors such as glioblastoma (GBM) due to systemic toxicities, poor T cell infiltration, and restricted drug penetration. We develop PL@mBiME, a multifunctional lipid nanoparticle (LNP) platform that enables brain tumor–targeted delivery and sustained in vivo expression of mRNA encoding a bispecific macrophage engager (BiME). The BiME simultaneously targets ErbB2 on glioma cells and CD206 on M2 macrophages, reprogramming macrophages toward pro-inflammatory M1 phenotype while promoting macrophage–tumor cell bridging, enhancing tumor cell phagocytosis and antigen presentation. PL@mBiME incorporates pH-responsive charge reversal to improve tumor accumulation and lysosomal escape as well as glutathione-triggered release of surface-conjugated PD-L1 antibody to amplify anti-tumor immunity. Across multiple GBM models, this coordinated activation of innate and adaptive immunity induces tumor regression, prolongs survival, and generates durable immune memory without significant toxicity.

## Abstract (web summary)

Bispecific immune cell engagers show limited efficacy in glioblastoma (GBM) therapy due to poor T cell infiltration and restricted drug penetration. Here this group reports a multifunctional lipid nanoparticle platform that enables brain tumor–targeted delivery and sustained in vivo expression of mRNA encoding a bispecific macrophage engager for GBM treatment.

## Introduction

Glioblastoma (GBM) is the most prevalent malignant primary brain tumor, accounting for approximately 48% of all primary malignant central nervous system (CNS) tumors^1^. Despite advances in surgery, chemotherapy, and radiotherapy, GBM continues to pose a formidable clinical challenge, with poor prognosis and extremely low long-term survival rates^2,3^. Immunotherapy has transformed the treatment landscape for many cancers^4^, but its efficacy in GBM remains limited. Key barriers include the blood–brain barrier (BBB), which restricts the delivery of biologics such as antibodies and nucleic acids^5–9^, and a profoundly immunosuppressive tumor microenvironment (TME), largely shaped by tumor-associated macrophages (TAMs), which comprise up to 30–50% of intratumoral cells^10–12^. Direct interactions between TAMs and tumor cells promote tumor growth and immune evasion^13^, highlighting the need for strategies that both penetrate the BBB and reprogram the immunosuppressive TME.

Bispecific cell engagers (BiCEs) have recently emerged as a promising immunotherapy class, designed to redirect immune effector cells—such as T cells, NK cells, or macrophages—toward tumor cells^14–16^. First-generation BiCEs, such as bispecific T cell engagers (BiTEs), showed efficacy in hematologic malignancies by linking CD3 on T cells with tumor antigens, but their application in solid tumors is hindered by T cell infiltration barriers and cytokine release syndrome (CRS). Second-generation engagers targeting innate immune cells offer an alternative approach with potentially improved safety and efficacy^17,18^. In GBM, TAMs—especially monocyte-derived, M2-like populations characterized by CD206 expression—are highly abundant and plastic, making them an attractive target for redirection^19^. Notably, the RP-182 peptide has been shown to bind CD206 and reprogram M2-like macrophages toward a pro-inflammatory M1 phenotype by promoting lysosomal activation and apoptotic pathways^20^. Besides, ErbB2 (HER2), a receptor tyrosine kinase frequently overexpressed in GBM, has emerged as a viable tumor antigen for targeted therapies^21,22^. Together, these findings support the rationale for a bispecific macrophage engager (BiME) targeting CD206 and ErbB2 to bridge TAMs and glioma cells and promote macrophage-mediated tumor clearance.

However, the clinical translation of BiCEs, including BiMEs, faces significant limitations—such as short half-life, manufacturing complexity, and poor BBB permeability. In contrast, in vivo expression of BiCEs using messenger RNA (mRNA) offers several advantages. mRNA enables flexible, scalable production, avoids genomic integration, and allows host cells to locally produce complex, difficult-to-manufacture proteins in situ^23,24^. For GBM, direct mRNA transfection to the tumor region could overcome delivery limitations and allow precise modulation of the immune microenvironment. Yet, effective mRNA delivery to the brain remains challenging due to the BBB and lack of targeted, responsive delivery systems.

Lipid nanoparticles (LNPs), the leading non-viral mRNA carriers, have demonstrated success in systemic applications but face hurdles in GBM therapy—particularly regarding BBB penetration, tumor specificity, and intracellular delivery efficiency. To address these challenges, multifunctional LNPs incorporating targeting ligands and responsive elements have been developed^25^. For instance, surface decoration with peptides such as Angiopep-2 (A2) facilitates BBB transcytosis via binding to low-density lipoprotein receptor-related protein 1 (LRP-1), which is highly expressed on brain endothelial cells^26,27^. In addition, pH-responsive charge-reversal lipids, such as DCPA, enable enhanced uptake in the acidic TME and promote endosomal escape via protonation and membrane disruption at pH < 6.8^28,29^. Furthermore, glutathione (GSH)-responsive systems can enable tumor-selective release of conjugated therapeutics in the reductive tumor microenvironment, especially relevant in chemoresistance GBM^30,31^, which often exhibits elevated GSH levels.

**Fig. 1:**
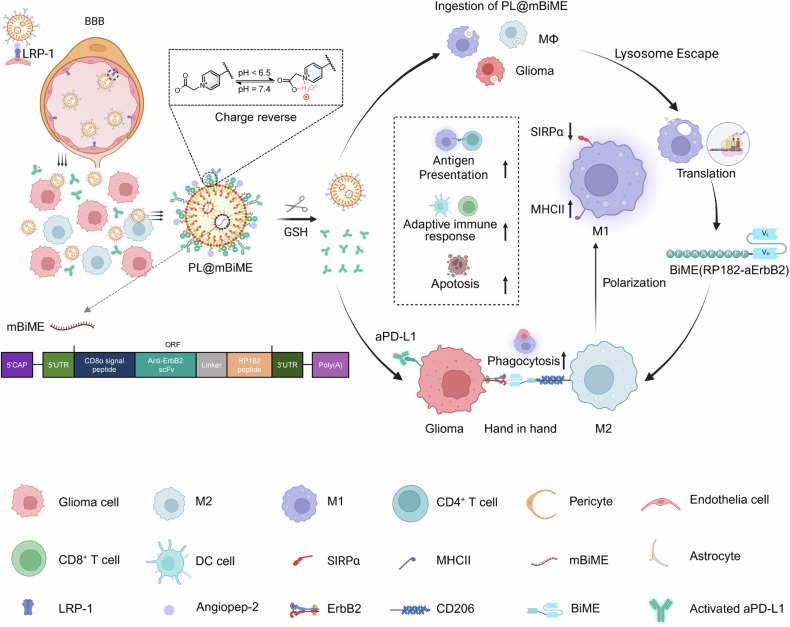
Illustration of PL@mBiME-Mediated TAM Reprogramming and Tumor Cell Phagocytosis to Enhance Both Innate and Adaptive Immunity in GBM Immunotherapy. Created in BioRender. Fei, G. (2026) https://BioRender.com/bq1g0ow.

In this work, we develop an anti-programmed death ligand 1 antibody (aPD-L1) coated acid-responsive charge-reversible lipid nanoparticle (LNP) loaded with mRNA encoding a bispecific macrophage engager (PL@mBiME) (Fig. 1). The BiME consists of a single-chain variable fragment (scFv) targeting ErbB2 and the TAM-reprogramming peptide RP-182 targeting CD206. The LNP is designed to respond to the TME by enhancing tumor uptake via charge reversal and enabling GSH-triggered release of aPD-L1, thereby allowing co-delivery of mBiME mRNA and immune checkpoint blockade specifically within the tumor. Once expressed, BiME increases the interaction between glioma cells and TAMs, reprograms macrophages toward a tumoricidal M1 state, and promotes antigen presentation and phagocytosis. In synergy with PD-L1 blockade, this approach reshapes the GBM immune microenvironment and elicits potent anti-tumor immunity. Our results demonstrate that this strategy significantly enhances mRNA delivery and therapeutic efficacy, offering a promising direction for mRNA-based immunotherapy in GBM.

## Results

### Synthesis and Characterization of pH- and GSH-Responsive PL@mBiME

Consistent with previous reports^21,22^, our analysis of The Cancer Genome Atlas (TCGA) database revealed that ErbB2 is significantly overexpressed in glioma tissues from GBM patients compared to normal brain tissue (Supplementary Fig. 1a). Notably, high ErbB2 expression correlates with poor prognosis, as evidenced by a markedly reduced median survival time in the ErbB2 high-expression group relative to the low-expression group (Supplementary Fig. 1b). These findings suggest that ErbB2 may represent a promising therapeutic target for GBM.

**Fig. 2:**
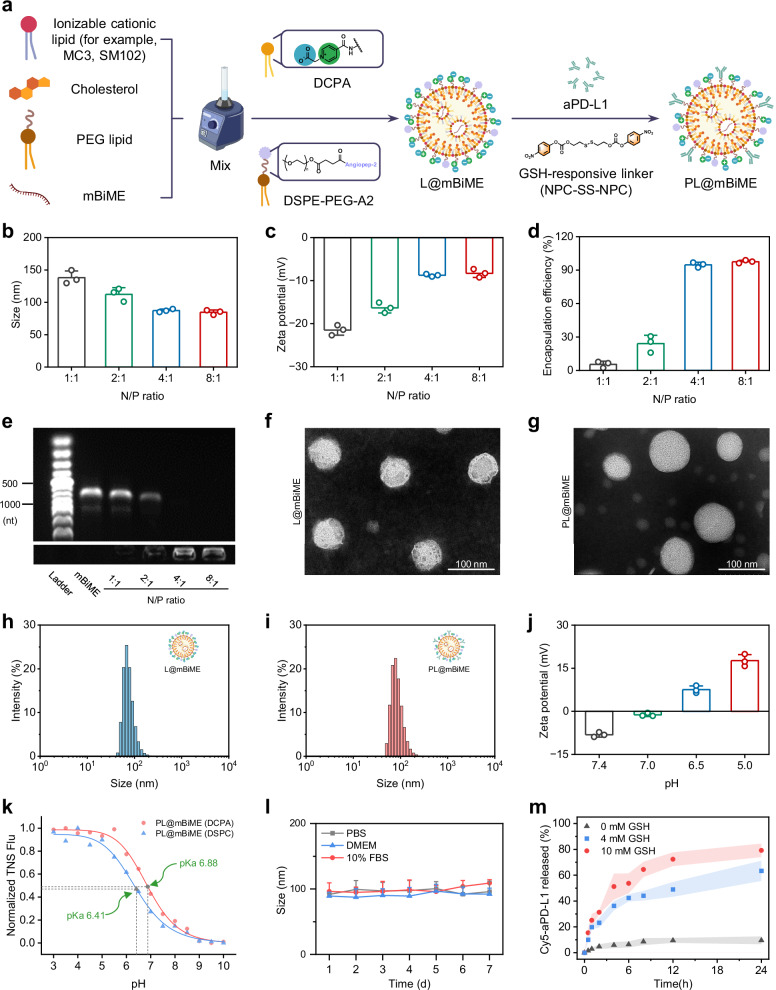
Preparation and characterization of PL@mBiME. **a** Schematic diagram of the synthesis procedure of L@mBiME and PL@mBiME. Created in BioRender. Fei, G. (2026) https://BioRender.com/bq1g0ow. **b**, **c** Particle size and zeta potential of PL@mBiME with different N/P ratios in Tris-HCl buffer (pH = 7.4, 0.01 M), *n* = 3 independent experiments. **d** Encapsulation efficiency assay of PL@mBiME with different N/P ratios by RiboGreen, *n* = 3 independent experiments. **e** Agarose gel electrophoresis analysis of PL@mBiME at different N/P ratios, *n* = 3 independent experiments. **f**, **g** Transmission electron microscope (TEM) image of L@mBiME and PL@mBiME with N/P ratio of 4:1, *n* = 3 independent experiments. Scale bar = 100 nm. **h**, **i** Dynamic light scatterings (DLS) analysis of the mean particle size distribution of L@mBiME and PL@mBiME with N/P ratio of 4:1. **j** Zeta potentials of PL@mBiME at different pH values in Tris-HCl buffer, *n* = 3 independent experiments. **k** pKa determination of PL@mBiME formulations containing DCPA or DSPC via the trinitrobenzenesulfonic acid (TNS) method. **l** Stability characteristics of PL@mBiME in PBS, DMEM, and10% FBS for 7 days, *n* = 3 independent experiments. **m** Responsive release of the Cy5-aPD-L1 in 0, 4, or 10 mM GSH, *n* = 3 independent experiments. Data are presented as mean ± SD. Source data are provided as a Source Data file.

To develop a delivery platform targeting ErbB2, we designed and fabricated an acidity-responsive, charge-reversible lipid nanoparticle (LNP) system for mRNA delivery. The mRNA encodes a bispecific macrophage engager composed of an *ErbB2*-targeting single-chain variable fragment (scFv) fused to RP-182 (Supplementary Table 1), a peptide that selectively binds to CD206, a marker highly expressed on tumor-associated macrophages (TAMs). This construct is referred to as L@mBiME. To further enhance its immunotherapeutic efficacy, the nanoparticle surface was modified with an anti-programmed death ligand 1 antibody (aPD-L1), resulting in the final formulation, PL@mBiME (Fig. 2a).

As a key component of the LNP, we synthesized a acidity-responsive helper lipid, DCPA, following standard synthetic procedures (Supplementary Fig. 2 and Supplementary Fig. 3). To facilitate blood–brain barrier (BBB) penetration and enhance accumulation in GBM lesions, we conjugated the BBB-targeting ligand A2 to DSPE-PEG-NHS, yielding DSPE-PEG-A2 (Supplementary Fig. 4). The final LNP formulation was prepared with SM102, DCPA, cholesterol, and DSPE-PEG-NH₂ at a molar ratio of 50:10:38.5:1.5, and supplemented with approximately 0.1% (M/M) of DSPE-PEG2000-A2. Next, aPD-L1 was stably conjugated to the surface of L@mBiME via a glutathione (GSH)-responsive disulfide linker (NPC-SS-NPC), yielding the final construct PL@mBiME with approximately 120 ± 15 aPD-L1 molecules and 35 ± 5 A2 peptides per LNP. (Supplementary Fig. 5 and Supplementary Table 2).

We next characterized the physicochemical properties of the nanoparticles, including particle size, morphology, zeta potential, and mRNA encapsulation efficiency across different nitrogen-to-phosphorus (N/P) ratios (Fig. 2b–e). As the N/P ratio increased, particle size decreased while zeta potential increased (Fig. 2b, c). Maximum mRNA encapsulation was achieved at N/P ratios ≥ 4:1 (Fig. 2d, e), and transfection efficiency peaked at this ratio, as assessed using PL@mBiME-T2A-EGFP (Supplementary Fig. 6 and Supplementary Fig. 7). Therefore, an N/P ratio of 4:1 was selected for all subsequent experiments. Under this optimized condition, dynamic light scattering (DLS) revealed that L@mBiME and PL@mBiME had an average hydrodynamic diameter of 76.8 ± 5.8 and 86.9 ± 6.3 nm, respectively (Supplementary Table 3). Transmission electron microscopy (TEM) confirmed a uniform spherical morphology for both formulations (Fig. 2f–i).

To assess the pH-responsive charge reversal, PL@mBiME was incubated in buffered solution at different pH. The zeta potential increase as the pH decreased, shifting from −8.15 ± 0.82 mV at pH 7.4 to + 17.65 ± 2.10 mV at pH 5.0, confirming the surface charge reversal under acid condition (Fig. 2j). PL@mBiME formulated with DCPA exhibited an elevated acid dissociation constant (pKa) of 6.88, compared with the control nanoparticles incorporating DSPC (Fig. 2k). This optimized pKa enables charge reversal of DCPA-modified nanoparticles in the weakly acidic tumor microenvironment (pH ~6.5), thereby enhancing electrostatic interaction with negatively charged membranes and facilitating cellular uptake (Fig. 2k). The nanoparticles also exhibited excellent colloidal stability, maintaining consistent size after incubation in PBS, 10% fetal bovine serum (FBS), or high-glucose DMEM at 4 °C for 7 days (Fig. 2l).

We next evaluated the GSH-responsive release of surface-conjugated aPD-L1 using Cy5-labeled antibody. To validate the physiological relevance of this release mechanism, we first measured the extracellular GSH concentration in the tumor-bearing brain, which was found to be 4.75 ± 0.29 mM (Supplementary Fig. 8). Next, we measured the release of aPD-L1 under different concentration of GSH. The results showed that aPD-L1 was gradually released in the presence of GSH, reaching a plateau of approximately 50% and 75% release at 12 h under 4 mM and 10 mM GSH, respectively. In contrast, negligible release was observed in the absence of GSH, indicating the stability of the disulfide bond under non-reducing conditions and its efficient cleavage under reductive conditions (Fig. 2m).

To further investigate the timing of PD-L1 antibody release, we labeled aPD-L1 with FITC and mBiME with Cy5 dye. We then co-incubated PL@mBiME with GL261 cells in medium containing either 0 mM or 4 mM GSH. Confocal microscopy images revealed that, under the 0 mM GSH condition, the PD-L1 antibody co-localized with mBiME inside the cells. In contrast, under the 4 mM GSH condition, most of the PD-L1 antibody was released extracellularly, with minimal co-localization observed with Cy5-mBiME (Supplementary Fig. 9). This result indicates that PD-L1 antibody undergoes extracellular release in response to GSH in the tumor microenvironment prior to cellular uptake.

Collectively, these findings demonstrate that PL@mBiME possesses well-defined pH-sensitive charge reversal, high colloidal stability, and efficient GSH-triggered antibody release, establishing its potential as a versatile nanoplatform for GBM-targeted mRNA immunotherapy.

### PL@mBiME Exhibits pH-Triggered Cellular Uptake, Rapid Lysosomal Escape, and Efficient In Vitro Expression

**Fig. 3:**
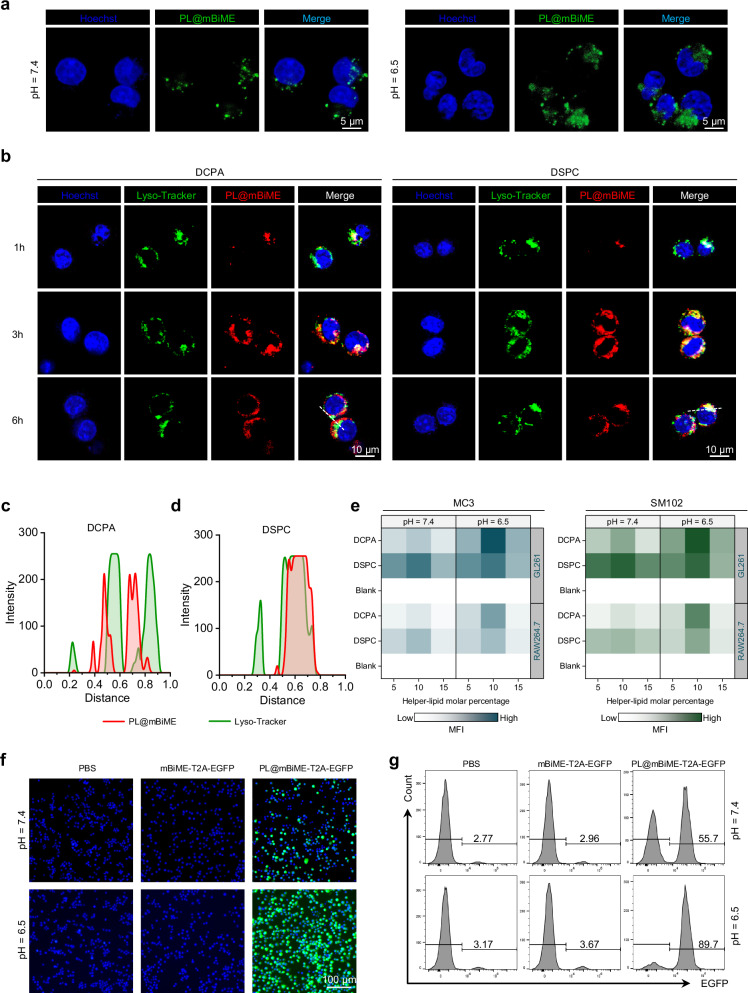
Uptake and expression of PL@mBiME in vitro. **a** Uptake of PL@mBiME by RAW264.7 cells at pH 7.4 or 6.5, *n* = 3 independent experiments. Scale bar = 5 μm. **b** Representative confocal images of RAW264.7 cells incubated with PL@mBiME for 1, 3 or 6 h. Nuclei were stained with Hoechst (blue), endosomes/lysosomes were stained with Lyso-Tracker Green (green), and mRNA was labeled with Cy5 (red), *n* = 3 independent experiments. Scale bar = 10 μm. **c**, **d** Intensity of both red (PL@mBiME) and green (Lyso-tracker) channels along with the corresponding white lines in the “Merged” CLSM images analyzed by Image J software showing their colocalization at 6 h. **e** EGFP expression was measured to test the transfection efficiency of LNPs with different helper lipids at different molar percentages. Transfections were performed in RAW264.7 and GL261 cell lines using MC3 or SM102 formulations, respectively. Transfection efficiency quantified as mean fluorescence intensity (MFI) is shown as the mean of three biological experiments in the heat map, *n* = 3 independent experiments. **f** Expression of EGFP was analyzed by a fluorescence microscope after 24 h transfection of the PL@mBiME-T2A-EGFP to the RAW264.7 cells, *n* = 3 independent experiments. Scale bar = 100 μm. **g** Flow cytometric histograms of PL@mBiME-T2A-EGFP transfection in RAW264.7 cells at pH 7.4 or 6.5, *n* = 3 independent experiments. Source data are provided as a Source Data file.

Efficient nucleic acid expression requires both successful cellular uptake of nanoparticles and effective escape of mRNA from lysosomes. To assess the cellular internalization and lysosomal escape behavior of our LNP platform under acidic conditions, we labeled mBiME with fluorescein isothiocyanate (FITC) and examined uptake by RAW264.7 macrophages and GL261 glioma cells using confocal laser scanning microscopy (CLSM) at pH 7.4 and pH 6.5. Upon incubation with PL@mBiME, RAW264.7 cells exhibited markedly stronger intracellular fluorescence at pH 6.5 compared to pH 7.4, indicating enhanced uptake under acidic conditions (Fig. 3a and Supplementary Fig. 10). A similar trend was observed in GL261 cells (Supplementary Fig. 11). These results suggest that under acidic conditions, DCPA-containing LNPs undergo surface charge reversal from negative to positive, enhancing electrostatic interactions with negatively charged cell membranes and thereby promoting cellular uptake.

Previous studies have shown that such pH-sensitive charge switching can also facilitate lysosomal membrane destabilization and endosomal escape following acidification in lysosomes^32,33^. To investigate lysosomal escape efficiency, RAW264.7 cells were incubated with either DCPA-based or DSPC-based PL@mBiME. Lysosomes were labeled with LysoTracker Green, and co-localization of PL@mBiME (red) and lysosomes (green) was assessed using CLSM. At 3 h post-incubation, strong co-localization was observed in both groups (Pearson correlation coefficient close to 1), indicating successful uptake and localization of Cy5-labeled mBiME within lysosomes. However, by 6 h, the DCPA group showed clear separation between green and red signals (Fig. 3b–d and Supplementary Fig. 12), with a reduced Pearson correlation coefficient, indicating substantial lysosomal escape. In contrast, nanoparticles in the DSPC group largely remained co-localized with lysosomes. These results confirm that DCPA facilitates both enhanced cellular uptake in acidic environments and more efficient lysosomal escape.

To test the expression efficiency, LNPs were formulated with SM102 and either DCPA or DSPC at varying molar ratios. SM102-DSPC LNPs showed minimal difference in EGFP intensity between pH 7.4 and 6.5. In contrast, SM102-DCPA LNPs exhibited significantly higher EGFP expression under acidic conditions, demonstrating superior pH-sensitive transfection capability (Fig. 3e). This trend was consistent in GL261 cells and also observed in LNPs formulated with MC3 lipid. Further experiments confirmed that DCPA-based LNPs mediated efficient EGFP expression in GL261 cells, RAW264.7 cells, and bone marrow–derived macrophages (BMDMs) (Supplementary Fig. 13). At pH 6.5, PL@mBiME induced EGFP expression in 89.7% of RAW264.7 cells and 93.8% of GL261 cells, which was significantly higher than transfection efficiencies at neutral pH (Fig. 3f, g, Supplementary Fig. 14 and Supplementary Fig. 15).

To assess formulation stability, PL@mBiME-T2A-EGFP was stored at 4 °C for 1, 4, and 7 days. Flow cytometry revealed that transfection efficiency remained above 75% even after 7 days, with minimal decline observed within the first 4 days (Supplementary Fig. 16), indicating good storage stability.

Finally, cytotoxicity assays demonstrated that PL@mBiME exhibited negligible toxicity in both RAW264.7 and GL261 cells, even at relatively high concentrations, indicating excellent biocompatibility (Supplementary Fig. 17).

### PL@mBiME Facilitates Tumor Cell and Macrophage Interaction and Enhances Macrophage Polarization and Tumor Cell Phagocytosis In Vitro

**Fig. 4:**
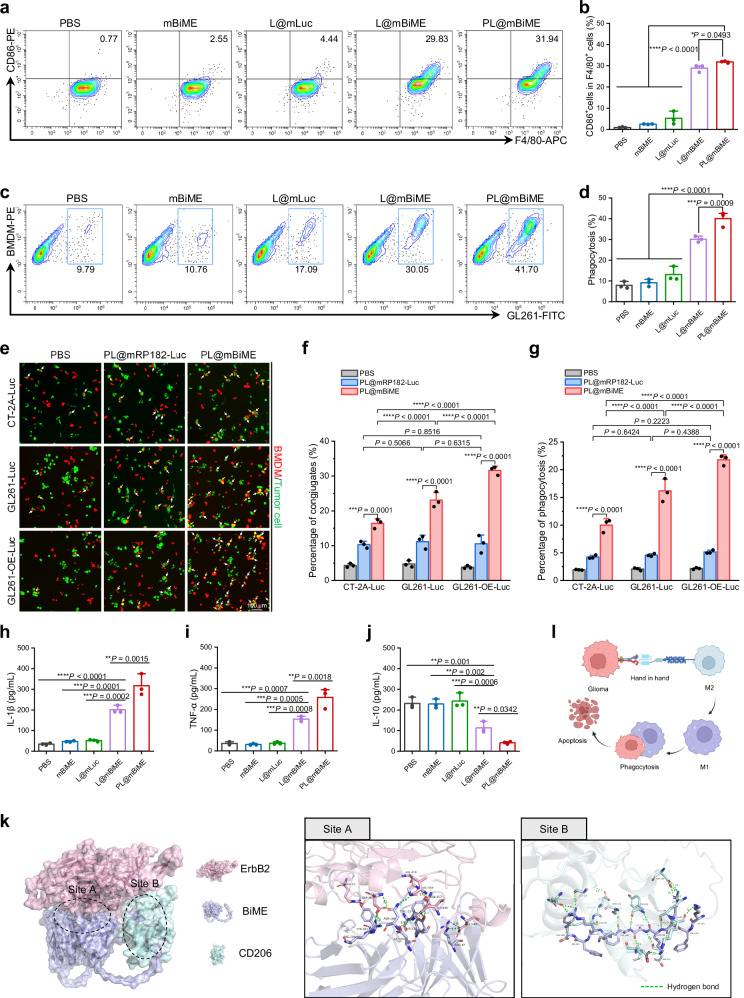
PL@mBiME Induces Macrophage Polarization and Enhances Tumor Cell Phagocytosis In Vitro. **a**, **b** Flow cytometry analysis and statistical charts of BMDMs polarization (M1 CD86) after adding PL@mBiME, *n* = 3 independent experiments. **c**, **d** Flow cytometry and statistical charts of the phagocytosis of glioma cells by macrophages treated with PBS, mBiME, L@mLuc, L@mBiME and PL@mBiME. BMDMs were labeled with CD11b, and GL261 was labeled with DiO, *n* = 3 independent experiments. **e–g** Representative fluorescence microscopy images and quantification of conjugates and phagocytosis between tumor cells (DiO, green) and BMDMs (DiI, red). White arrows indicate conjugate formation between BMDMs and tumor cells, while yellow arrows denote phagocytosis of tumor cells by BMDMs. Scale bar = 100 µm, *n* = 3 independent experiments. **h–j** Changes in the secretion of IL-1β, TNF-α and IL-10 after treatment with different formulations, *n* = 3 independent experiments. **k** In silico prediction of the multivalent docking mode of BiME. The molecular structure and binding conformation of the bispecific BiME molecule with its targets, ErbB2 and CD206, were predicted using AlphaFold3. Overall predicted structure of the BiME molecule, highlighting its distinct domains (left), Predicted binding interfaces and interaction details between BiME and ErbB2 (middle) or CD206 (right), showcasing key residues involved in the molecular recognition. **l** Schematic representation of macrophage-mediated tumor cell engulfment enhanced by PL@mBiME treatment. Created in BioRender. Fei, G. (2026) https://BioRender.com/bq1g0ow. Data are presented as mean ± SD. Statistical analysis was performed by one-way ANOVA with Fisher’s LSD post‑hoc test for (**b**, **d**, **h**–**j**). Statistical analysis was performed by two-way ANOVA with Fisher’s LSD post‑hoc test for f and g. Source data are provided as a Source Data file.

To assess the functional consequences of BiME expression in macrophages, we co-cultured RAW264.7 cells with GL261 glioma cells. The results showed that BiME expression significantly promoted macrophage migration toward tumor cells (Supplementary Fig. 18a–c). RP-182, a component of BiME, is known to reprogram M2-like macrophages to an antitumor M1 phenotype via CD206 engagement. To verify this effect, IL-4–induced M2-polarized BMDMs were treated with different formulations. Flow cytometry and Western blot analysis confirmed that PL@mBiME increased M1 markers (CD86, iNOS) and reduced M2 markers (CD206, ARG-1) (Fig. 4a, b; Supplementary Fig. 19 and Supplementary Fig. 20a, b), resulting in a significantly higher M1/M2 ratio and indicating effective M2-to-M1 repolarization (Supplementary Fig. 20c).

To explore the phagocytosis of macrophages mediated by PL@mBiME, co-culture of GL261 cells and BMDMs treated with various formulations was carried out for 24 h. Flow cytometry analysis revealed that 41.7% of BMDMs phagocytosed tumor cells—4.26- and 3.88-fold higher than PBS and mBiME groups, respectively (Fig. 4c, d). Additionally, apoptosis of GL261 cells induced by macrophages treated with L@mBiME and PL@mBiME increased by 2.52-fold and 3.49-fold, respectively (Supplementary Fig. 21).

Next, to confirm the critical role of ErbB2 in BiME-mediated tumor–macrophage bridging and subsequent phagocytosis, recombinant ErbB2 protein was added to a co-culture of GL261 and RAW264.7 cells to competitively inhibit BiME function. The presence of free ErbB2 significantly reduced phagocytic activity from 38.8% to 25.1% in the PL@mBiME + ErbB2 group (Supplementary Fig. 22), and correspondingly decreased the proportion of apoptotic GL261 cells (Supplementary Fig. 23). Furthermore, we generated GL261-OE-Luc cells by overexpressing ErbB2 in GL261-Luc cells and measured the surface ErbB2 expression in CT-2A-Luc, GL261-Luc, and GL261-OE-Luc cells. Flow cytometry results showed that GL261-Luc cells expressed 47.8% ErbB2, CT-2A-Luc cells expressed 17.8%, and GL261-OE-Luc cells exhibited the highest expression at 93.1% (Supplementary Fig. 24a). Next, to evaluate the specific contribution of the ErbB2 antibody in the BiME construct, we replaced the ErbB2 antibody sequence with luciferase to generate RP182-Luc mRNA. Co-culture of BMDMs with CT-2A-Luc, GL261-Luc, or GL261-OE-Luc cells revealed that, compared to PBS and PL@mRP182-Luc, PL@mBiME significantly increased the formation of physical “bridges” between macrophages and tumor cells and enhanced phagocytic activity in all cell groups (Fig. 4e–g, Supplementary Fig. 24). This effect was ErbB2-dependent, as the higher conjugate formation and phagocytosis were observed only in PL@mBiME-treated group. In contrast, the PL@mRP182-Luc treatment group showed similar levels of “bridges” and phagocytosis across CT-2A-Luc, GL261-Luc, and GL261-OE-Luc cells (Fig. 4e–g, Supplementary Fig. 24). These findings support the essential role of ErbB2 in mediating BiME-dependent macrophage engagement and tumor cell killing.

Furthermore, enzyme-linked immunosorbent assay (ELISA) analysis revealed that treatment with PL@mBiME significantly upregulated the secretion of pro-inflammatory cytokines IL-1β and TNF-α, while simultaneously downregulating the anti-inflammatory cytokine IL-10 (Fig. 4h–j). These results further confirm the shift toward an M1-like, pro-inflammatory macrophage phenotype.

Finally, to provide structural insight into how BiME may simultaneously engage macrophages and tumor cells, we performed molecular docking simulations using AlphaFold3 to model a putative ternary complex between BiME, CD206, and ErbB2. The predicted model revealed two potential interaction interfaces: site A, where the anti-ErbB2 scFv of BiME forms hydrogen-bond with ErbB2, and site B, where the RP-182 domain is positioned within a cavity of CD206 (Fig. 4k). While these docking results are predictive in nature and do not constitute experimental proof of binding, they provide a structural rationale that is consistent with our cellular data, supporting the rational design of BiME as a dual-targeting engager capable of bridging macrophages and tumor cells.

Collectively, these results demonstrate that PL@mBiME effectively reprograms macrophages into a pro-inflammatory, phagocytic phenotype and enhances tumor cell clearance through BiME-mediated immune bridging and cytokine activation (Fig. 4l).

### PL@mBiME Effectively Penetrates the Blood–Brain Barrier and Selectively Accumulates and Expresses in the Tumor Region

**Fig. 5:**
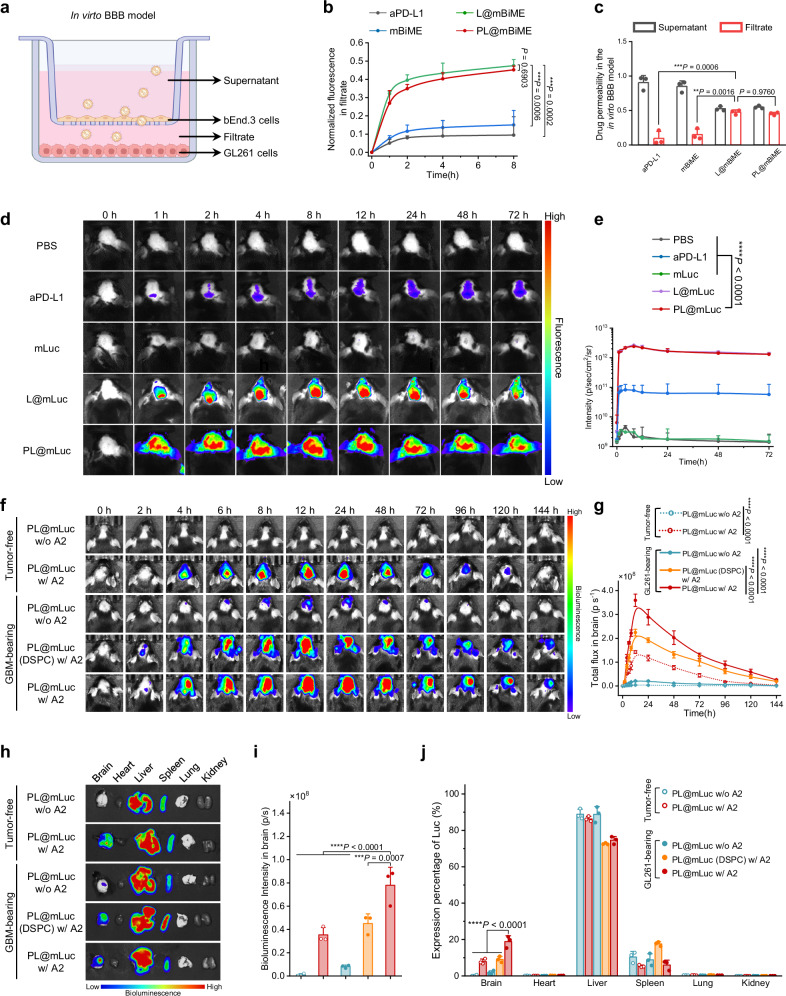
Assessment of penetration ability by PL@mRNA in vitro and in vivo. **a** Schematic illustration of the in vitro BBB model using a transwell system to evaluate the penetration capability of PL@mBiME across the endothelial monolayer. Quantities of PL@mBiME were determined in the supernatant, bEnd.3 cells, filtrate, and GL261 cells. Created in BioRender. Fei, G. (2026) https://BioRender.com/bq1g0ow. **b**, **c** Release and quantification of the PL@mBiME distribution in the chamber after incubation with aPD-L1, mBiME, L@mBiME and PL@mBiME for 1, 2, 4, and 8 h, *n* = 3 independent experiments. **d**, **e** Fluorescence imaging and intensity quantification of tumor sites at 1, 2, 4, 8, 12, 24, 48, and 72 h after *i.v*. injection of GL261 tumor-bearing mice with PBS, aPD-L1, mLuc, L@mLuc and PL@mLuc, *n* = 3 independent experiments. **f**, **g** Bioluminescence imaging of brain in tumor-free and GBM-bearing mice at 0, 2, 4, 6, 8, 12, 24, 48, 72 h, 96 h, 120 h, and 144 h post-administration of various formulations, *n* = 3 independent experiments. **h** Representative bioluminescence imaging of isolated brains from tumor-free versus GBM-bearing mice at 12 h post-administration of various formulations. **i** Quantitative analysis of brain bioluminescence intensity in tumor-free versus GBM-bearing mice at 12 h post-administration of various formulations, *n* = 3 independent experiments. **j** Quantification analysis of the percentage of total bioluminescence signal attributed to major organs (brain, heart, liver, spleen, lung, kidney), *n* = 3 independent experiments. Data are presented as mean ± SD, statistical analysis was performed by one-way ANOVA with Fisher’s LSD post‑hoc test. Source data are provided as a Source Data file.

Systemic delivery of mRNA to the central nervous system remains a major challenge due to the restrictive nature of the BBB. Receptor-mediated endocytosis and transcytosis offer promising strategies to enhance the BBB permeability of brain-targeted nanoparticles. To evaluate the BBB-penetrating capability of PL@mBiME, we established an in vitro BBB model using bEnd.3 brain microvascular endothelial cells cultured in the upper chamber and GL261 glioma cells in the lower chamber (Fig. 5a). After 4 days of incubation, tight junction formation was validated using FITC-dextran. Cy5-labeled formulations were then applied, including aPD-L1, mBiME, L@mBiME, and PL@mBiME. Quantitative analysis of supernatant and filtrate at 1, 2, 4, and 8 h revealed that L@mBiME and PL@mBiME achieved permeability rates of 45.43% and 42%, respectively—2.8- and 2.6-fold higher than aPD-L1 (Fig. 5b, c). These results indicate that the A2 peptide facilitates LRP-1–mediated transcytosis, enhancing nanoparticle transport across the endothelial barrier.

To validate BBB penetration in vivo, GL261 tumor-bearing mice were intravenously injected with Cy7-labeled formulations (aPD-L1, mLuc, L@mLuc, PL@mLuc). Fluorescence imaging showed weak brain signals in PBS, aPD-L1, and mLuc groups at all time points. In contrast, strong brain fluorescence was observed in L@mLuc and PL@mLuc groups, peaking at 12 h post-injection and exceeding the aPD-L1 group by 41.3- and 38.1-fold, respectively (Fig. 5d, e and Supplementary Fig. 25a, b). Signal intensity persisted for over 72 h, indicating efficient BBB crossing and prolonged brain retention (Fig. 5d, e). Ex vivo organ distribution confirmed that peripheral accumulation occurred primarily in the liver and spleen (Supplementary Fig. 25c, d). Bioluminescence imaging at 12 h further confirmed robust mRNA expression in the brains of L@mLuc- and PL@mLuc-treated mice, with signal intensities 66.1- and 89.1-fold higher than the mLuc group, respectively (Supplementary Fig. 26a–d). Ex vivo imaging of isolated brains confirmed strong luciferase expression in the tumor regions, with peripheral expression concentrating in the liver and spleen (Supplementary Fig. 26e, f).

For further visualizing BiME localization within tissues, EGFP expression was examined in brain and liver tissue after injecting PL@mBiME-T2A-EGFP. Strong EGFP fluorescence was observed specifically within the tumor region in brain whereas EGFP expression in liver is relatively even (Supplementary Fig. 26g), suggesting successful tumor-targeted expression in brain. Furthermore, we also measured the release of aPD-L1 payload in both brain tumor and liver tissue. As expected, aPD-L1 was significantly lower in the liver compared to the tumor-bearing brain, due to potentially much lower GSH level (Supplementary Fig. 26h, i).

To further elucidate the individual contributions of the A2 peptide (for blood-brain barrier penetration) and the DCPA lipid (for pH-responsive membrane fusion) within our PL@mLuc platform in vivo, we assessed the biodistribution in both tumor-bearing and tumor-free mice, with or without A2 peptide conjugation on the nanoparticles. Longitudinal bioluminescence imaging (Fig. 5f) and quantitative analysis at 12 h (Fig. 5g) revealed that, A2 peptide conjugation markedly increased brain luciferase expression in both tumor-free and tumor-bearing mice, with an approximately 9.8-fold increase in tumor-bearing mice compared to non-conjugated particles. Strikingly, in tumor-bearing mice, the incorporation of DCPA into the LNPs significantly enhanced luciferase expression in the brain, with a corresponding decrease in expression in the spleen (Fig. 5h–j). These results suggest that the DCPA component in PL@mLuc synergizes with the A2-mediated delivery by enabling more efficient payload release and expression specifically within the acidic tumor milieu.

Overall, these findings demonstrate that the A2 peptide enables efficient BBB penetration via receptor-mediated transcytosis, while the pH-responsive DCPA component facilitates tumor-specific accumulation and expression. Together, these features position PL@mBiME as a promising nanoplatform for targeted mRNA delivery to brain tumors.

### PL@mBiME Effectively Inhibits Tumor Growth and Prolongs Survival in Orthotopic Glioblastoma Mouse Model

**Fig. 6:**
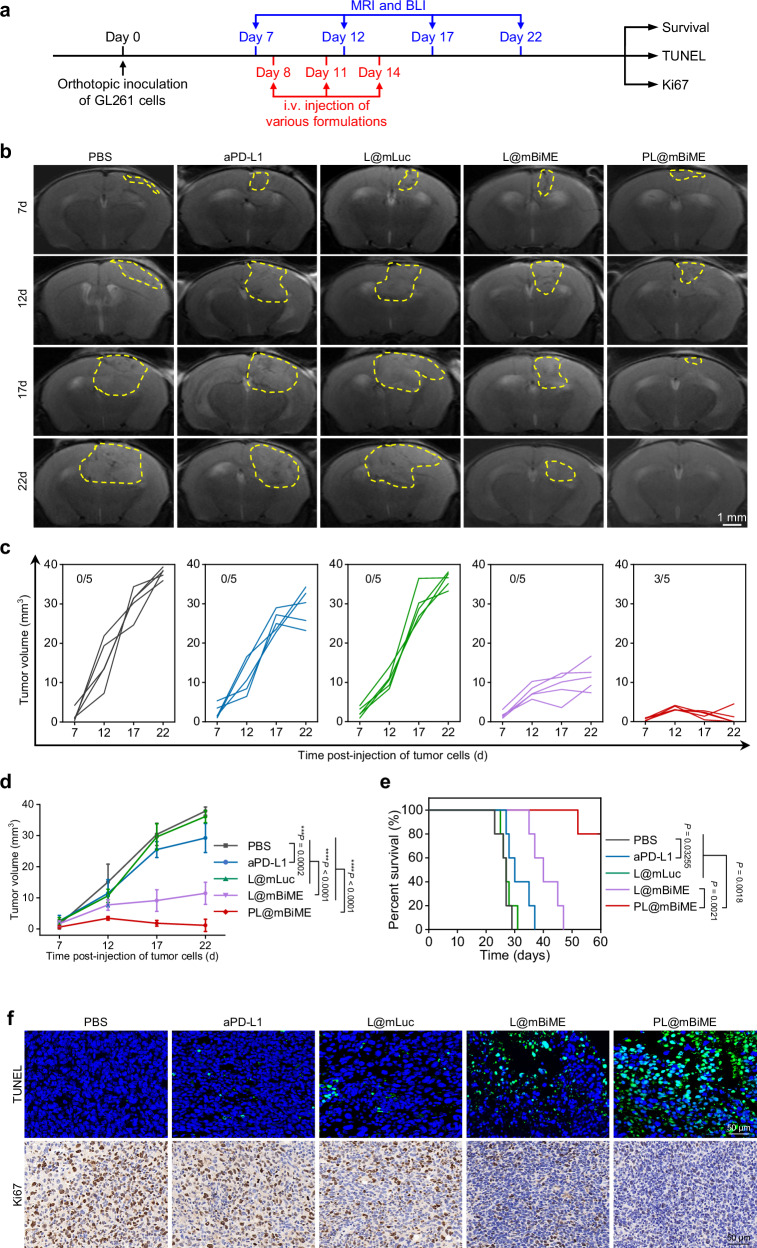
In vivo therapeutic effect of PL@mBiME on GL261 tumor-bearing mice. **a** Schematic diagram of the treatment regimen. **b** Representative MR images and quantified signal intensities of GL261 tumor-bearing mice treated with PBS, aPD-L1, L@mLuc, L@mBiME and PL@mBiME. The yellow dotted line indicates the tumor area. Scale bar = 1 mm. **c**, **d** Relative tumor volumes of mice after different treatments, *n* = 5 independent experiments. **e** Survival curves of GL261 tumor-bearing mice following treatment with different formulations, *n* = 5 independent experiments. The statistical significance was calculated by the log-rank test. **f** Representative images of Ki67 and TUNEL staining in tumor tissues on day 22, *n* = 5 independent experiments. Scale bar = 50 μm. Data are presented as mean±SD, statistical analysis was performed by one-way ANOVA with Fisher’s LSD post‑hoc test. Source data are provided as a Source Data file.

To evaluate the therapeutic efficacy of PL@mBiME in vivo, we first employed GL261 orthotopic glioma model. Tumor-bearing mice were intravenously injected with PBS, aPD-L1, L@mLuc, L@mBiME, or PL@mBiME on days 8, 11, and 14 post-tumor implantation (1 mg kg^−^^1^ for mBiME and 5 mg kg^−^^1^ for aPD-L1) (Fig. 6a). Tumor progression was monitored by MRI every 5 days starting from day 7. Compared to PBS, aPD-L1, and L@mLuc groups, PL@mBiME treatment resulted in the most profound tumor regression, while L@mBiME also led to notable, though less pronounced, inhibition of tumor growth (Fig. 6b–d). Interestingly, the aPD-L1 group showed limited therapeutic benefit, likely due to poor accumulation of the antibody in the brain. This highlights the necessity of combining the A2 peptide for BBB penetration and the DCPA component for pH-responsive delivery. Together, these modifications significantly improved the delivery and therapeutic impact of the PD-L1 antibody and BiME in the brain.

This conclusion was further supported by bioluminescence imaging of luciferase-expressing GL261-Luc tumors. Mice treated with L@mBiME and PL@mBiME exhibited sustained tumor suppression, whereas tumors in the other groups continued to grow rapidly (Supplementary Fig. 27). Survival analysis revealed that mice receiving L@mBiME or PL@mBiME showed significantly extended survival times, with PL@mBiME achieving the best outcome. Remarkably, 80% of mice treated with PL@mBiME exhibited complete tumor regression and remained tumor-free during the study period (Fig. 6e).

Furthermore, we evaluated the efficacy of PL@mBiME in another orthotopic GBM model, CT-2A-Luc. Similar to the GL261-Luc model, both L@mBiME and PL@mBiME demonstrated significant anti-tumor effects, with PL@mBiME treatment resulting in the most potent tumor suppression (Supplementary Fig. 28). Additionally, we tested PL@mRP182-Luc treatment, which lacks the ErbB2 antibody sequence in the mRNA construct. As expected, the anti-tumor effects of PL@mRP182-Luc were reduced, confirming the importance of the ErbB2 antibody for effective tumor suppression. However, in contrast to the GL261-Luc model, no tumors in the PL@mBiME treatment group reached completed regression in the CT-2A-Luc model (Supplementary Fig. 28). This is likely due to the lower expression of ErbB2 on CT-2A cells compared to GL261 cells (Supplementary Fig. 24a).

PL@mBiME shows preferential accumulation in acidic environments; however, whether tumor acidity is critical for its anti-tumor efficacy remained unclear. To investigate this, we knocked out the Ldha1 gene in GL261-Luc cells, which catalyzes the conversion of pyruvate to lactate during glycolysis (Supplementary Fig. 29a). Ldha1 knockout significantly increased the intratumoral pH and concomitantly reduced BiME expression within the tumor area (Supplementary Fig. 29b, c). As a result, the tumor-suppressive efficacy of PL@mBiME was also attenuated in Ldha1-knockout tumors (Supplementary Fig. 29d–f).

Histological analysis on day 22 of GL261 tumor revealed that PL@mBiME significantly suppressed tumor cell proliferation (Ki67 staining), induced pronounced apoptosis (TUNEL staining), and reduced overall tumor burden (H&E staining), compared to all other treatment groups (Fig. 6f; Supplementary Figs. 30–32).

To assess systemic toxicity, body weight was monitored throughout treatment. Mice in the L@mBiME and PL@mBiME groups regained weight as tumors regressed, while weight loss was observed in PBS, aPD-L1, and L@mLuc groups (Supplementary Fig. 33). H&E staining of major organs (heart, liver, spleen, lung, and kidney) revealed no histopathological abnormalities (Supplementary Fig. 34). Biochemical analysis of liver (ALT, AST) and kidney (BUN, Cr) function showed no significant abnormalities or group differences (Supplementary Fig. 35), supporting the biosafety of PL@mBiME treatment.

Given the high accumulation of PL@mBiME in the liver and the expression of CD206 on liver-resident macrophages (Kupffer cells), which is one of the targets of BiME, we next comprehensively assessed the effects of PL@mBiME on Kupffer cells. Flow cytometric analysis revealed that approximately 10% of Kupffer cells expressed the delivered constructs (Supplementary Fig. 36a–c). Nevertheless, PL@mBiME treatment did not alter the overall proportion of Kupffer cells within the liver (Supplementary Fig. 36d, e). Importantly, in contrast to the repolarization observed in tumor-associated macrophages, the phenotype of hepatic Kupffer cells remained unchanged. Key M2 (CD206) and M1 (CD86) polarization markers, as well as the scavenger receptor CD163, showed no significant differences compared to control groups. Furthermore, the phagocytic capacity of Kupffer cells was not significantly altered following PL@mBiME treatment (Supplementary Fig. 36f–m).

We next evaluated the potential impact of PL@mBiME on higher-order brain function, a critical consideration for any intracranial therapy. Cognitive function, particularly spatial learning and memory which is highly dependent on hippocampal integrity, was assessed using the Morris water maze test in GL261 tumor-bearing mice (Supplementary Fig. 37a). To minimize confounding effects of tumor burden on cognitive performance, a tumor-bearing control group with tumor sizes comparable to those of the PL@mBiME-treated group, but receiving no treatment, was included.

Compared to the tumor-bearing control group, PL@mBiME-treated mice exhibited similar escape latency during training (Supplementary Fig. 37b), comparable time spent in the target quadrant during the probe trial (Supplementary Fig. 37c), and an equivalent number of platform crossings (Supplementary Fig. 37d). In contrast, mice in the PBS and other treatment groups showed significantly impaired performance. These differences are likely attributable to the reduced tumor burden achieved by PL@mBiME treatment relative to the other treatment groups.

These results demonstrate that the potent anti-tumor immunity elicited by PL@mBiME does not come at the cost of cognitive impairment. The preservation of complex neural functions underscores the neurological safety of our approach and suggests that the localized immune activation and tumor clearance do not induce damage to cognition-critical neural circuits.

### PL@mBiME Bridges Macrophages with Tumor Cells and Reprograms TAMs to the M1 Phenotype and Enhances Anti-Tumor Efficacy In Vivo

**Fig. 7:**
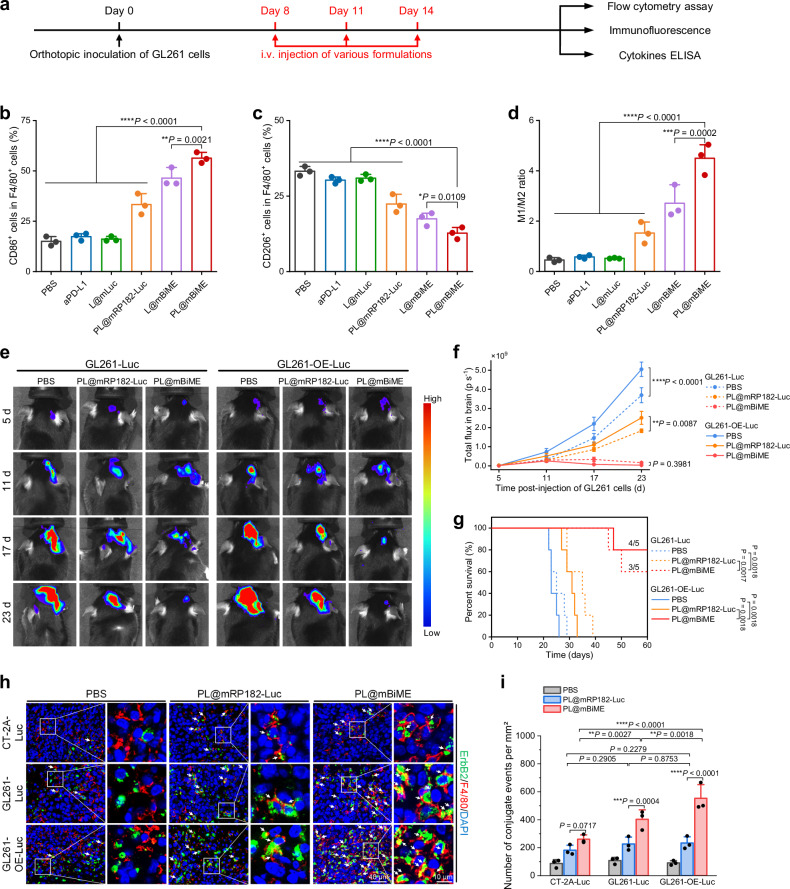
PL@mBiME reprograms TAMs in GBM and increases the phagocytic ability of macrophages. **a** Timeline for examining the in vivo immune response in the GL261 bearing mice induced by PL@mBiME. **b**, **c** Quantification of tumor-infiltrating TAM polarization (M1 marker CD86, M2 marker CD206) cells three days after three treatments with PBS, aPD-L1, L@mLuc, PL@mRP182-Luc, L@mBiME, and PL@mBiME, *n* = 3 independent experiments. **d** Quantification of the M1/M2 ratio in tumor-infiltrating tumor-associated macrophages (TAMs) by flow cytometry, *n* = 3 independent experiments. **e**, **f** Representative IVIS images and quantified signal intensities of GL261-Luc and GL261-OE-Luc tumor-bearing mice treated with PBS, PL@mRP182-Luc and PL@mBiME, *n* = 3 independent experiments. **g** Survival curves of GL261-Luc and GL261-OE-Luc tumor-bearing mice following treatment with different formulations, *n* = 5 independent experiments. **h**, **i** Representative immunofluorescence (IF) images and statistical analysis of macrophage‑tumor cell conjugation (green: ErbB2, red: F4/80, white arrows indicate macrophage‑tumor cell conjugates). Scale bar = 40 µm, 10 µm, *n* = 3 independent experiments. Data are presented as mean ± SD. Statistical analysis was performed by one-way ANOVA with Fisher’s LSD post‑hoc test for (**b**–**d**). Statistical analysis was performed by two-way ANOVA with Fisher’s LSD post‑hoc test for (**f**, **i**). Survival analysis (**g**) was compared using the log-rank test. Source data are provided as a Source Data file.

To investigate whether PL@mBiME reprograms tumor-associated macrophages (TAMs) in vivo, we analyzed macrophage phenotypes in the tumor microenvironment on day 4 post-injection (Fig. 7a). Flow cytometry revealed that both L@mBiME and PL@mBiME treatment increased the proportion of pro-inflammatory M1 macrophages (CD86⁺) and reduced M2 macrophages (CD206⁺), with PL@mBiME producing the most significant shift in the M1/M2 ratio (Fig. 7b–d; Supplementary Figs. 38, 39). These findings were corroborated by immunofluorescence staining of tumor tissues, where PL@mBiME-treated tumors exhibited a notable increase in CD86⁺ macrophages and a decrease in CD206⁺ macrophages (Supplementary Fig. 40), indicating successful M2-to-M1 repolarization in TAMs.

To further assess functional activation, we measured SIRPα (a key receptor transmitting the “don’t eat me” signal) and MHCII (a marker of antigen-presenting capability) in TAMs. PL@mBiME significantly decreased the proportion of SIRPα⁺ cells from 47.0% to 12.1%, while increasing MHCII⁺ macrophages by 5.7-fold, compared to PBS groups (Supplementary Fig. 38b and 41). In vitro, co-culture of GL261 cells with BMDMs treated with various formulations yielded consistent results: PL@mBiME treatment decreased SIRPα⁺ cells from 64.9% to 28.9% and increased MHCII⁺ cells by 6.49-fold (Supplementary Fig. 42), suggesting that PL@mBiME suppresses the CD47–SIRPα axis and enhances antigen presentation.

Next, to determine the role of ErbB2 in mediating the in vivo activity of PL@mBiME, we established orthotopic tumors using GL261-Luc and GL261-OE-Luc cells and treated mice with PBS, PL@mRP182-Luc, or PL@mBiME. Consistent with the oncogenic function of ErbB2, GL261-OE-Luc tumors exhibited significantly accelerated growth compared to GL261-Luc tumors in both the PBS and PL@mRP182-Luc treatment groups (Fig. 7e–g). In contrast, PL@mBiME treatment resulted in pronounced tumor growth inhibition and significantly prolonged survival in both GL261-Luc and GL261-OE-Luc models, with a greater degree of tumor suppression observed in the GL261-OE-Luc model and a higher proportion of tumor-free mice achieved in this group (Fig. 7e–g).

Representative tumors from CT-2A-Luc, GL261-Luc, and GL261-OE-Luc models were analyzed by immunofluorescence. Markedly increased colocalization of ErbB2 (green) and the macrophage marker F4/80 (red) was observed in PL@mBiME-treated tumors across all three models, indicating enhanced macrophage–tumor cell bridging events compared to both PBS- and PL@mRP182-Luc–treated groups. Notably, the extent of macrophage–tumor cell conjugate formation increased with tumor ErbB2 expression, progressing from CT-2A-Luc (low) to GL261-Luc (intermediate) and GL261-OE-Luc (high) tumors (Fig. 7h, i). Together, these findings demonstrate that PL@mBiME promotes macrophage–tumor cell physical interactions in an ErbB2-dependent manner in vivo, with higher tumor ErbB2 expression associated with enhanced therapeutic efficacy.

Finally, ELISA analysis of tumor tissues revealed that PL@mBiME treatment significantly increased pro-inflammatory cytokines—TNF-α, IL-1β, and IFN-γ—while reducing the anti-inflammatory cytokine IL-10 (Supplementary Fig. 43). Together, these results confirm that PL@mBiME reprograms TAMs toward a pro-inflammatory, phagocytic phenotype and contributes to tumor clearance through immune remodeling.

### PL@mBiME Activates Innate and Adaptive Immune Responses in the GBM Microenvironment

**Fig. 8:**
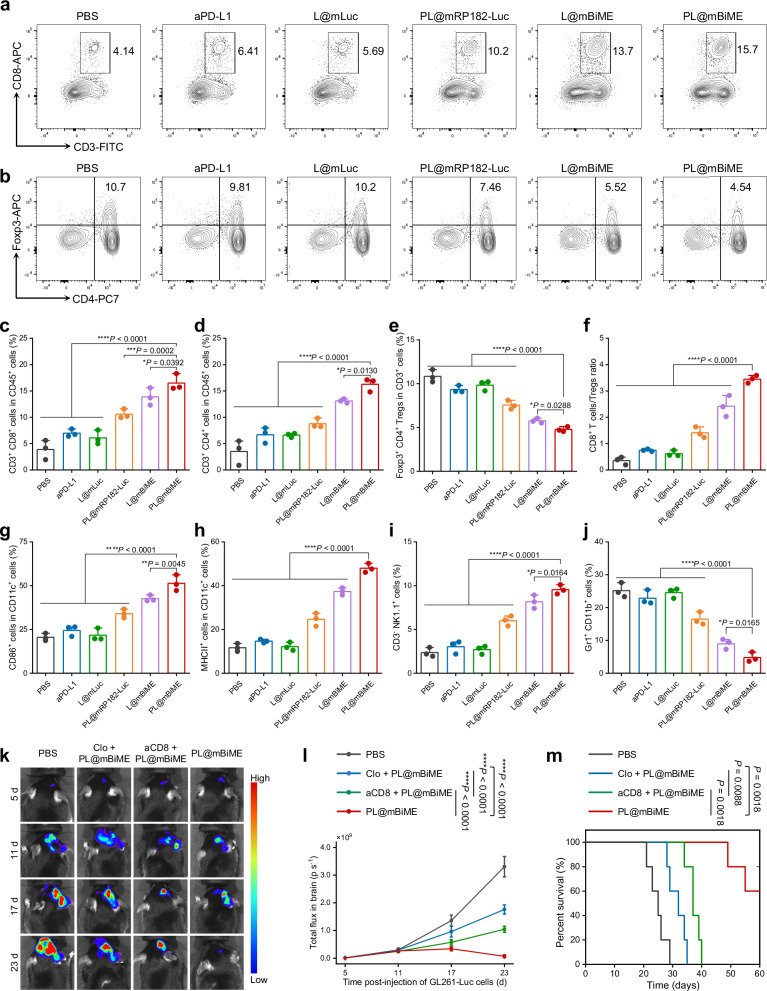
PL@mBiME activates innate and adaptive immune responses in the GBM microenvironment. **a**, **b** Representative flow cytometry plots of tumor-infiltrating CD8 and Treg cells three days after three injections of PBS, aPD-L1, L@mLuc, PL@mRP182-Luc, L@mBiME, and PL@mBiME treatments. **c–j** Flow cytometry statistical quantification diagrams of tumor-infiltrating CD8, CD4, Treg, CD8/Treg, DC (MHCII, CD86), NK, MDSC three days after three injections of PBS, aPD-L1, L@mLuc, L@mBiME, and PL@mBiME treatments, *n* = 3 independent experiments. **k**, **l** Serial in vivo bioluminescence images (**k**) and quantification of tumor flux (**l**) show tumor growth in GL261 tumor-bearing mice treated with PL@mBiME following depletion of the indicated immune cell populations, *n* = 3 independent experiments. **m** Kaplan-Meier survival curves of the corresponding treatment groups, *n* = 5 independent experiments. Data are presented as mean ± SD, statistical analysis was performed by one-way ANOVA with Fisher’s LSD post‑hoc test for (**c**–**j**, **l**). Survival analysis (**m**) was compared using the log-rank test. Source data are provided as a Source Data file.

To investigate downstream immune effects of PL@mBiME treatment, we assessed infiltration of both innate and adaptive immune cells in the tumor microenvironment. Flow cytometry showed that PL@mBiME markedly increased the proportion of CD4 T cell and activated CD8⁺ cytotoxic T lymphocytes (CTLs), reaching 15.7% of CD45⁺ cells—3.8-fold higher than PBS groups. In contrast, regulatory T cells (Tregs; Foxp3⁺CD4⁺) were significantly reduced from 10.7% to 4.5%. The CD8⁺ T cell/Treg ratio was significantly elevated in the PL@mBiME group, indicating a shift toward a more immunostimulatory environment (Fig. 8a–j; Supplementary Figs. 44a and 45). Moreover, markers of mature dendritic cells, including CD86 and MHCII, increased by 2.7-fold and 4.8-fold respectively in the PL@mBiME group compared to controls (Fig. 8g, h; Supplementary Figs. 44b, 46 and 47, Supporting Information), suggesting enhanced antigen presentation. Natural killer (NK) cell numbers were also increased by 3.4-fold (Fig. 8i and Supplementary Fig. 48), while myeloid-derived suppressor cell (MDSC) infiltration was dramatically reduced (Fig. 8j; Supplementary Fig. 49).

Immunofluorescence staining and immunohistochemical (IHC) staining of brain tumor sections confirmed a markedly enhanced infiltration of CD8⁺ T cells in the PL@mBiME group (Supplementary Figs. 50 and 51a, b). Importantly, these infiltrating CD8⁺ T cells were functionally active, as evidenced by a substantial increase in the expression of the cytolytic effector molecule granzyme B (Supplementary Fig. 51c, d).

To delineate the immune effector populations responsible for the anti-tumor efficacy of PL@mBiME, we performed depletion studies using a neutralizing antibody to deplete CD8⁺ T cells and clodronate liposomes to deplete macrophages (Supplementary Fig. 52a–h). The results showed that depletion of CD8⁺ T cells mildly attenuated the tumor-inhibitory effects of PL@mBiME (Fig. 8k–m). More strikingly, macrophage depletion resulted in a more pronounced loss of anti-tumor efficacy (Fig. 8k–m), providing direct functional evidence that macrophages are one of the principal effector populations mediating the anti-tumor response induced by PL@mBiME.

Collectively, these findings demonstrate that PL@mBiME not only reprograms TAMs but also stimulates broad innate and adaptive immune responses, contributing to tumor suppression and immune microenvironment remodeling.

### PL@mBiME Induces Long-Lasting Anti-Tumor Immune Memory

**Fig. 9:**
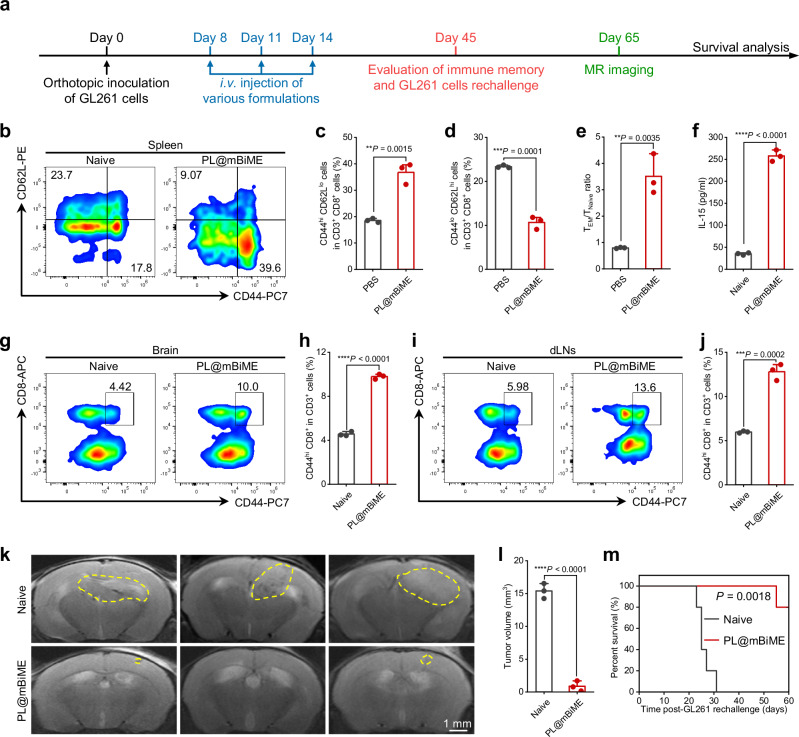
Durable immune memory generated by PL@mBiME in GBM. **a** Illustration of the in vivo research plan. On day 8, 11, and 14 after GL261 cell transplantation, mice received different treatments via intravenous injection. Then, among the mice that survived on day 45 after inoculation, 3 mice were selected for the analysis of effector memory T cells in splenocytes. **b–d** Representative flow cytometry smoothed dot plots of effector memory T cells (T_EM_) with CD44^hi^ expression and low CD62L^lo^ expression and naive T cells (T_Naive_) with CD44^lo^ expression and CD62L^hi^ expression in the spleens of naïve mice and mice treated with PL@mBiME, *n* = 3 independent experiments. **e** Quantitative analysis of the ratio of T_EM_ to T_Naive_ in the spleens of mice after different treatments by flow cytometry, *n* = 3 independent experiments. **f** The cytokine levels of interleukin-15 (IL-15) in tumor tissues after different treatments, *n* = 3 independent experiments. **g–j** Representative flow cytometry smoothed dot plots and quantitative analysis of CD8⁺CD44⁺ memory T cells in the brains and draining lymph nodes (dLNs) of naive mice and mice treated with PL@mBiME, *n* = 3 independent experiments. **k**, **l** Magnetic resonance (MR) images and quantitative analysis on day 65 after re-inoculation of GL261 cells in naive mice and mice treated with PL@mBiME. The yellow dotted line indicates the tumor area, *n* = 3 independent experiments. Scale bar = 1 mm. **m** Survival curves of naïve mice, *n* = 4 independent experiments, and mice treated with PL@mBiME, *n* = 3 independent experiments, after re-inoculation of GL261 cells. Data are presented as mean ± SD, statistical analysis was performed by unpaired two-tailed Student’s t-test for (**c**–**f**, **h**, **j**, **l**). Survival analysis (**m**) was compared using the log-rank test. Source data are provided as a Source Data file.

To evaluate whether PL@mBiME induces durable immune memory capable of preventing tumor recurrence, long-term surviving mice from the PL@mBiME group were re-challenged with GL261 cells on day 45 post-treatment. Age matched naïve mice were used as control (Fig. 9a). Flow cytometry of splenocytes revealed a marked increase in CD44^hi^CD62L^lo^ effector memory T cells (T_EM_), accompanied by a decrease in CD44^lo^CD62L^hi^ naïve T cells (T_Naive_), resulting in a 3.64-fold increase in the T_EM_/T_Naive_ ratio (Fig. 9b, e; Supplementary Fig. 44d). In parallel, IL-15 levels—critical for TEM maintenance—were significantly elevated in the PL@mBiME group (Fig. 9f). Increased CD44⁺CD8⁺ T cell populations were also detected in both the brains and draining lymph nodes of treated mice, with levels 2.65- and 2.28-fold higher than those of naïve mice, respectively (Fig. 9g–j; Supplementary Fig. 44c).

MRI analysis confirmed significantly delayed tumor growth in PL@mBiME-treated mice upon re-challenge, with corresponding extension of survival (Fig. 9k–m). These results demonstrate that PL@mBiME not only achieves tumor eradication but also primes robust and long-lasting immune memory, offering protection against GBM recurrence. This dual action—effective tumor clearance and immune education—underscores the potential of PL@mBiME as a transformative strategy in GBM immunotherapy.

## Discussion

In this study, we developed a multifunctional LNP system, PL@mBiME, to address two central challenges in GBM immunotherapy: efficient delivery across the BBB and modulation of the profoundly immunosuppressive TME. By co-delivering mRNA encoding a bispecific macrophage engager (mBiME) and a GSH-responsive anti–PD-L1 antibody (aPD-L1), this platform induces coordinated activation of innate and adaptive immunity, resulting in robust tumor suppression and long-lasting immune protection.

The emergence of multispecific immune cell engagers (including BiCEs) has provided new therapeutic avenues for tumor targeting and immune activation^14–16^. However, BiTEs, which rely on T cells, face major obstacles in solid tumors—including limited T cell infiltration and the risk of cytokine release syndrome^14,15^. In contrast, TAMs are abundant in most solid tumors, particularly in GBM, where they comprise 30–50% of intratumoral cells^10,11^. Their phenotypic plasticity and deep infiltration into the TME make them attractive effector cells for immunotherapy. The RP-182 peptide, which reprograms CD206⁺ M2-like macrophages into a pro-inflammatory M1 phenotype via lysosomal activation^20^, combined with an anti-ErbB2 scFv to engage glioma cells^21^, forms the core of our mBiME construct. This design not only reprograms TAMs but also bridges macrophages with tumor cells, enhancing selective phagocytosis and antigen presentation. ErbB2 expression in GBM is heterogeneous across cohorts and assays, which has fueled debate over its general importance; however, multiple studies support ErbB2 as a clinically actionable tumor-associated antigen in a subset of GBMs and demonstrate the feasibility of ErbB2-directed immune recognition^22,34^. GL261 is known to exhibit relatively strong immune activation compared with other syngeneic glioma models, and immune composition varies across models^35,36^. In this context, evaluation in CT-2A provides a more immunosuppressive setting in which anti-tumor activity is still observed^37^, indicating that the platform is not restricted to a single GBM model, while the magnitude of response may depend on antigen expression and immune context.

Traditional protein-format BiCE therapeutics suffer from poor serum half-life, manufacturing complexity, and low BBB permeability, limiting their applicability in GBM. Notably, prior studies have shown that mRNA-encoded bispecific antibodies can overcome protein manufacturing and short half-life limitations by enabling in vivo expression, but these efforts have largely focused on non-CNS contexts and did not address BBB-restricted delivery^38–41^. By leveraging the advantages of mRNA—transient expression, scalable synthesis, and lack of genomic integration—we enabled localized production of the BiME in vivo, overcoming challenges associated with protein-based delivery. However, systemically administered mRNA–LNPs typically exhibit limited tumor targeting and poor BBB penetration, representing a central barrier for translating mRNA-encoded engagers to GBM^42^.

To address this, we incorporated multiple design features into the LNP: (1) Angiopep-2 (A2), which facilitates transcytosis across the BBB via LRP-1-mediated transport^26,27^; (2) DCPA, a pH-responsive charge-reversal lipid, which enhances cellular uptake and lysosomal escape in the acidic tumor environment^29^; and (3) aPD-L1 conjugated through a GSH-cleavable disulfide linker to allow tumor-selective release in the reductive microenvironment. These features acted synergistically to ensure tumor-selective delivery, efficient mRNA expression, and immune activation with minimal off-target effects.

Unlike CSF1R tyrosine kinase inhibitors, which primarily deplete immunosuppressive TAMs, our mRNA-based bispecific macrophage engager (BiME) aims to reprogram and engage TAMs to directly phagocytose tumor cells and initiate adaptive anti-tumor immunity. This approach may circumvent issues like compensatory mechanisms or loss of antigen-presenting function associated with depletion strategies^43,44^. Furthermore, local BiME expression via nanoparticle delivery ensures high intratumoral concentration and sustained activity, potentially overcoming pharmacokinetic limitations seen with systemic CSF1R inhibition.

Our results confirm that PL@mBiME effectively penetrated the BBB and accumulated within GBM lesions. The DCPA component enabled rapid charge reversal in acidic conditions, facilitating enhanced cellular uptake and lysosomal escape, while A2 decoration significantly improved brain accumulation. Upon internalization, mBiME expression led to TAM polarization toward an M1 phenotype, suppressed the CD47–SIRPα “don’t eat me” axis, and increased MHCII expression—thus enhancing antigen presentation and pro-inflammatory cytokine production. These effects were accompanied by increased infiltration and activation of CD8⁺ T cells, a reduction in Tregs, and a higher CD8⁺/Treg ratio. Crucially, Granzyme B expression in CD8⁺ T cells was significantly upregulated, demonstrating the activation of their potent cytotoxic function. Additionally, DCs exhibited enhanced maturation (CD86⁺MHCII⁺), NK cell numbers increased, and MDSC infiltration was suppressed—collectively indicating effective TME remodeling.

Importantly, the therapeutic effects of PL@mBiME were durable. Mice treated with PL@mBiME showed strong protection against tumor rechallenge, characterized by increased CD44^hi^CD62L^lo^ effector memory T cells, elevated IL-15 levels, and enhanced CD44⁺CD8⁺ T cell populations in the brain and lymph nodes. These findings highlight the ability of our system not only to eliminate tumors but also to establish lasting immune memory, a key feature of successful immunotherapy.

Nonetheless, some limitations remain, including the inherent differences between simplified in vitro systems and the complex in vivo tumor microenvironment, which may influence the magnitude and mechanisms of immune responses observed^19,45,46^. Second, our current BiME construct only targets murine ErbB2, which restricts potential clinical translation. In future studies, we will focus on humanizing both the target and the antibody component to validate translational potential. In parallel, we will employ patient-derived xenograft or humanized mouse models expressing human HER2 to further confirm target specificity, toxicity, and therapeutic relevance. Further, the use of DSPE-PEG-NH₂ as an anchor lipid for post-formulation conjugation of aPD-L1 enables modular surface modification, but may result in random antibody orientation or partial denaturation, potentially affecting biodistribution or eliciting unintended immune responses. Alternative strategies, such as site-specific conjugation or scFv-based constructs, will be considered to improve antibody orientation and preserve functional activity. Finally, more comprehensive comparisons between our LNP and traditional formulations—specifically regarding tumor accumulation, biodistribution, and immunogenicity—are necessary to fully characterize the translational potential of this delivery system.

In conclusion, PL@mBiME represents a rationally designed mRNA nanoplatform that achieves targeted delivery, selective activation of innate and adaptive immunity, and durable tumor control in GBM. By integrating brain-targeted delivery, environmental responsiveness, and immune synergism, this system demonstrates strong translational promise not only for GBM but also for other ErbB2⁺ and macrophage-enriched malignancies.

## Methods

### Ethics statements

C57BL/6 mice (6–8 weeks old) were purchased from Jiangsu Huachuang Sino Pharma Tech Co., Ltd. The mice were co-housed in the specific pathogen-free (SPF) care facility with free access to food and water under a 12 h light/dark cycle. All mice were maintained at 22–26 °C with humidity levels between 30% and 50%. All animal experiments were performed in compliance with the relevant laws and approved by the Institutional Animal Care and Use Committee of Southeast University School of Medicine (NO. 20220316049). None of the tumors reached a volume of 100 mm^3^ (the maximum tumor size).

### Bioinformatics analysis

ErbB2 gene expression in GBM patients and healthy populations retrieved from the Cancer Genome Atlas was analyzed through interactive gene expression profiling (GEPIA; http://gepia.cancer-pku.cn/detail.php?gene=&clicktag=boxplot). Survival analysis of GBM patients with high or low ErbB2 expression using http://gepia.cancer-pku.cn/detail.php?gene=&clicktag=boxplot.

### Synthesis of GSH-responsive crosslinker

Dissolve 2,2’-dithiodiethanol (1.54 g, 10 mmol) and pyridine (5 mL) in dichloromethane (DCM, 50 mL). Stir the solution in an ice bath for 10 minutes. Then, slowly add dropwise a DCM solution of 4-nitrophenyl chloromethyl carbonate (4.22 g, 22 mmol). After the addition, allow the reaction mixture to return to room temperature and stir for 6 h. Wash the mixture with sodium bicarbonate solution and brine successively, dry over anhydrous sodium sulfate, and remove the solvent by rotary evaporation. Purify the product by silica gel column chromatography.

### Synthesis of Blood-Brain Barrier Penetrating Lipid DSPE-PEG-A2

Equimolar amounts of DSPE-PEG-COOH and N-Hydroxysuccinimide (NHS) were dissolved in dichloromethane (DCM). One equivalent of 1,3-dicyclohexylcarbodiimide (DCC) was added under an ice-bath, and the mixture was stirred at room temperature overnight. After filtering the precipitate, the solvent was removed by rotary evaporation to obtain DSPE-PEG-NHS.

The targeting conjugate DSPE-PEG-A2 was synthesized by coupling the Angiopep-2 (A2) peptide to the terminal of DSPE-PEG2000-NHS via amine-reactive chemistry.

Previously obtained DSPE-PEG2000-NHS (5 mg) was dissolved in 5 mL 0.01 M PBS (pH 7.4). To this solution, 5 mg Angiopep-2 peptide was added. The reaction mixture was stirred at 4 °C overnight to facilitate the conjugation between the NHS ester group of the lipid and the primary amines of the peptide. Following the conjugation, the reaction was quenched by adding an excess of Tris-HCl buffer (containing free amine groups) and incubating for 1 h at room temperature to hydrolyze any unreacted NHS esters. The crude mixture was then transferred to a dialysis bag (cut-off 500 Da) and dialyzed extensively against ultrapure water. The dialyzed solution was subsequently concentrated by rotary evaporation and purified by reverse-phase chromatography using a CombiFlash EZ Prep system (ISCO). The separation was monitored in real-time using both an Evaporative Light Scattering Detector (ELSD) and a UV detector. Fractions containing the desired product, DSPE-PEG-A2, were collected based on the characteristic elution profile. Finally, the pooled fractions were lyophilized to obtain DSPE-PEG-A2 as a white, hygroscopic powder.

The structure of the synthesized DSPE-PEG-A2 conjugate was verified using matrix-assisted laser desorption/ionization time-of-flight mass spectrometry (MALDI-TOF-MS).

### Synthesis of DCPA

N-Boc-L-glutamic acid (compound 1, 2.47 g, 10 mmol), 1-octadecanol (5.95 g, 22 mmol), and 4-dimethylaminopyridine (DMAP, 61.1 mg, 0.5 mmol) were dissolved in 40 mL of dichloromethane (DCM). After stirring the mixed solution under ice bath for 30 minutes, 1,3-dicyclohexylcarbodiimide (DCC, 5.16 g, 25 mmol) dissolved in 20 mL of DCM was slowly added. The solution was washed twice with saturated sodium chloride solution (50 mL), and the organic layer was collected, dried over anhydrous sodium sulfate, and filtered to remove the drying agent. Subsequently, dichloromethane was removed by distillation under vacuum to obtain the crude product of compound 2. Purification was performed via silica gel column chromatography using a gradient of petroleum ether:ethyl acetate (50:1), yielding 6.80 g of a white powder with a yield of 90.5%.

Under nitrogen protection, compound 2 (1.52 g, 2 mmol) was dissolved in a mixture of dichloromethane (DCM) and trifluoroacetic acid (TFA) (volume ratio 2:1) under ice bath to remove the Boc protecting group, yielding glutamic acid distearyl ester trifluoroacetate (compound 3). The mixture was then stirred at room temperature for 2 h. The reaction solution was added dropwise to ice-cold NaOH solution, extracted with ethyl acetate, dried over anhydrous sodium sulfate, and concentrated by rotary evaporation to obtain compound 3 as a white solid (1.30 g, yield 97.0%).

To prepare compound 4, isonicotinic acid (123.1 mg, 1 mmol), HATU (380.2 mg, 1 mmol), and diisopropylethylamine (DIEA, 0.330 mL, 2 mmol) were added to a solution of compound 3 (652 mg, 1 mmol) in dry dichloromethane (DCM, 25 mL) under nitrogen protection and ice bath. The mixture was stirred at room temperature for 6 h. Subsequently, ethyl acetate (50 mL) was added, and the solution was washed with 1% NaOH solution, 1% HCl solution, and saturated sodium chloride solution (50 mL). The organic layer was dried over sodium sulfate and filtered. Ethyl acetate was removed by distillation under vacuum to obtain the crude product of distearyl isonicotinoyl glutamate (compound 4). The crude compound 4 was purified by reprecipitation with ethyl acetate, yielding compound 4 as a white powder (740 mg, yield 97.7%).

Finally, the nitrogen atom in the pyridine ring of the compound was quaternized to prepare DCPA. To this end, bromoacetic acid (556 mg, 4 mmol) was added to a solution of compound 4 (328 mg, 0.5 mmol) in acetonitrile. After stirring and heating at 80 °C for 24 h, the solution was cooled, and the solvent was removed by rotary evaporation under reduced pressure to obtain crude DCPA as a pale-yellow powder. The crude DCPA was purified by reprecipitation with acetonitrile, yielding a white powder (325 mg, 79.3% yield).

### Preparation of LNPs

DCPA was synthesized in Supplementary Fig. 2. MC3, SM102, DSPC and Cholesterol were purchased from AVT (shanghai) Pharmaceutical Tech Co., Ltd. A lipid mixture composed of MC3 or SM102, DCPA or DSPC, cholesterol, and DSPE-PEG2000-NH_2_ (MedChemExpress) was prepared in ethanol as a 10 mg mL^−^^1^ stock solution, with a molar ratio of 50:10:38.5:1.5. The diluted lipid solution (4.5 mg mL^−1^) was mixed with an aqueous mRNA solution (0.1 mg mL^−1^) in 10 mM citrate buffer (pH 4) at a 1:3 organic-to-aqueous volume ratio using a microfluidic mixing device (INano X, Micronanobiologics) at a total flow rate of 2.4 mL/min at room temperature. The resulting LNPs were immediately diluted twofold with Tris-HCl buffer (pH 7.4, 0.01 M), followed by three rounds of ultrafiltration at 2000 × g using 10-kDa Amicon Ultra centrifuge filters (Millipore, Burlington, MA) at 4 °C, each time diluted with Tris-HCl buffer (pH 7.4, 0.01 M). Subsequently, the LNPs were filtered through a 0.22 μm membrane.

### Preparation of L@mBiME and PL@mBiME

Roughly 0.1% M/M of DSPE-PEG2000-A2 mixture was added to the formulated LNPs. The reaction was incubated at 4 °C for 48 h. The post-inserted LNP was then concentrated by an ultrafiltration system and slowly applied to a 90-cm-bed-length gravity-flow size exclusion column prepared with Sepharose-CL4B gel. The mobile phase was PBS. The fractions that contained LNP were collected and concentrated by the ultrafiltration to obtain L@mBiME. To release the encapsulated or surface-conjugated A2 peptide, the nanoparticles were treated with 0.5% (v/v) Triton X-100 in PBS for 30 minutes at 37 °C. The A2 peptide concentration in the lysate was determined using a Fluorometric Peptide Quantitation Assay Kit (Beyotime Biotechnology) according to the manufacturer’s protocol.

To construct the final formulation, the obtained L@mBiME was conjugated with an anti-programmed cell death ligand 1 (aPD-L1) antibody via a redox-responsive linker. Specifically, the heterobifunctional crosslinker NPC-SS-NPC (where NPC denotes a nitro phenyl carbonate ester group reactive toward amines, and SS denotes a cleavable disulfide bond) was employed. The crosslinker was added at a molar ratio of 1.2:1 (crosslinker to aPD-L1) to ensure efficient bridging. This mixture was then reacted with L@mBiME at a fixed molar ratio of aPD-L1 to L@mBiME at 0.5%. The conjugation reaction was conducted in PBS buffer (0.01 M PBS, pH 7.4) at 4 °C for 12–16 h with gentle stirring.

Following the reaction, the crude mixture was purified to remove unreacted crosslinker and antibody. The solution was transferred to a Vivaspin ultrafiltration device (Sartorius) with a molecular weight cut-off (MWCO) of 300 kDa and centrifuged. This process was repeated with multiple cycles of dilution and concentration (diafiltration) using the reaction buffer to thoroughly exchange the medium. The final retentate, containing the purified antibody-conjugated nanoparticles, was collected and designated as PL@mBiME. For the quantification of conjugated aPD-L1, the PL@mBiME nanoparticles were treated with 0.5% (v/v) Triton X-100 to disrupt the lipid bilayer and release the antibody. The aPD-L1 concentration in the supernatant was quantified using a commercial Rat IgG ELISA Kit (Beyotime Biotechnology) following the standard protocol. This assay measures the total IgG content, which directly corresponds to the amount of aPD-L1 present in the nanoparticle formulation.

### Quantification of Targeting Ligand and Conjugated Antibody

To quantify the efficiency of targeting ligand insertion, nanoparticles post-inserted with DSPE-PEG-A2 were lysed with 0.5% (v/v) Triton X-100. The concentration of the released A2 peptide in the lysate was determined using a Fluorometric Peptide Quantitation Assay Kit (Beyotime Biotechnology) according to the manufacturer’s protocol.

Subsequently, the antibody-conjugated prodrug nanoparticles (PL@mBiME) were obtained by conjugating aPD-L1 via a crosslinker, followed by purification using ultrafiltration (MWCO: 300 kDa). To quantify the amount of aPD-L1 conjugated to PL@mBiME, the nanoparticles were similarly treated with 0.5% (v/v) Triton X-100 to release the antibody. Subsequently, the aPD-L1 concentration in the supernatant was quantified using a Rat IgG ELISA Kit (Beyotime Biotechnology) as per the standard protocol, which measures total IgG content corresponding to the conjugated aPD-L1.

The LNP concentration was determined by nanoparticle tracking analysis (NTA) using a NanoSight Pro (Malvern Panalytical). The experimental procedure was performed according to a previously published method^47^.

$${Number}\,{of}\,A2\,{per}\,{LNP}\,=\,\frac{{\rho }_{A2}\times {N}_{A}}{{c}_{{LNP}}\times {M}_{A2}}$$

ρ_A2_: mass concentration of the A2 peptide

N_A_: Avogadro constant

C_LNP_: particle concentration of LNPs

M_A2_: molar mass of the A2 peptide

Similarly, the number of aPD-L1 antibodies per LNP was calculated using the same method.

### Fluorescent Labeling of aPD-L1 Antibody (FITC, Cy5, and Cy7)

The aPD-L1 antibody was fluorescently labeled with FITC, Cy5 or Cy7 using NHS ester chemistry. The following protocol was applied to all dyes with minimal adjustments for their specific molecular weights when calculating molar ratios.

1. Antibody Preparation:
  The aPD-L1 antibody was dialyzed into 0.01 M phosphate-buffered saline (PBS, pH 7.4) using a suitable dialysis membrane or desalting column. The antibody concentration was adjusted to 1 mg/mL in the same buffer prior to conjugation.
2. Dye Solution Preparation:
  The NHS-ester derivative of the fluorescent dye was dissolved in anhydrous dimethyl sulfoxide (DMSO) to prepare a 10 mM stock solution. The solution was used immediately or aliquoted and stored desiccated at −20 °C, protected from light and moisture.
3. Conjugation Reaction:
  The dye stock solution was added dropwise to the prepared antibody solution with gentle vortexing or stirring. The reaction was performed at a molar ratio of dye:antibody = 30:1. The reaction mixture was incubated at 4 °C overnight with continuous gentle agitation and protection from light.
4. Reaction Quenching:
  To quenche any unreacted NHS esters and terminate the reaction, an equal volume of 0.01 M Tris-HCl buffer (pH 7.4) was added. The mixture was incubated for 30 minutes at room temperature in the dark.
5. Purification:
  The crude conjugation mixture was transferred to an ultrafiltration device (MWCO: 10 kDa). Free dye and reaction byproducts were removed by performing three cycles of concentration and dilution with ice-cold PBS (pH 7.4). The final retentate was collected.
6. Characterization and Storage:
  The purified labeled antibody solution was filtered through a 0.22 µm membrane filter. A small aliquot was taken for characterization.

The final labeled antibody was stored at 4 °C in PBS, protected from light.

### Fluorescent Labeling of Lipid Nanoparticles (LNPs)

Two distinct fluorescent labeling strategies were employed to enable the tracking of LNPs and their mRNA cargo.

1. Lipid-Labeled LNPs (Cy7 Conjugation):
  LNPs bearing a near-infrared fluorescent label on their lipid component were prepared by incorporating DSPE-PEG2000-Cy7 into the standard lipid mixture during formulation. The fluorescent lipid was added at a molar ratio of 0.1% (M/M, relative to total lipid content). All other formulation parameters, including the microfluidic mixing procedure described in the general method, remained unchanged.
2. mRNA-Labeled LNPs (Fluorescent mRNA Cargo):
  LNPs encapsulating fluorescently labeled mRNA were formulated using the standard LNP synthesis protocol. The cargo consisted of mRNA that had been pre-labeled using a fluorescently modified nucleotide (FITC-UTP or Cy5-UTP) via in vitro transcription. The labeled mRNA was purified and then used directly in place of unmodified mRNA during the LNP assembly process, with no other alterations to the lipid composition or mixing procedure.

### Antibody

Specific antibody information, including catalog numbers and clone numbers, has been provided in Supplementary Table 4.

### The construction of mRNA

All mRNA sequences used in this study were codon-optimized and chemically synthesized by Suzhou NOVOPROTEIN Biotech Corporation.

The encoded BiME structure, from N- to C-terminus, is: CD8α signal peptide-anti-ErbB2 scFv-(G4S)3 linker-RP182. The construct of mBiME-T2A-EGFP co-expresses the BiME and enhanced green fluorescent protein (EGFP), separated by a self-cleaving T2A peptide sequence (BiME-T2A-EGFP), to monitor transfection efficiency and for cell tracing. The mRP182-T2A-Luc encodes a control immunopeptide, with the structure: CD8α signal peptide-RP182-T2A-Luciferase (Luc).

All synthesized mRNAs were co-transcriptionally capped at the 5’ end and incorporated with modified nucleotides (N1-methylpseudouridine) to enhance stability and translational efficiency, followed by a 3’ polyadenylate poly(A) tail.

To generate Fluorescein- or Cy5-labeled mBiME, mRNAs were synthesized by in vitro transcription using Fluorescein-UTP or Cy5-UTP, respectively.

### Characterization of PL@mBiME

Measure the size, polydispersity, and zeta potential using a Malvern Zeta sizer. Briefly, dilute the PL@mBiME solution 100× in 1× Tris-HCl to a final volume of 1 mL in a disposable polystyrene cuvette to evaluate the particle size and polydispersity. For the zeta potential measurement, dilute the PL@mBiME solution 100× in 0.1× Tris-HCl and measure it in a disposable folded capillary cell. Use the Ribogreen kit to measure the encapsulation efficiency. According to the manufacturer’s protocol, measure the fluorescence with a microplate reader and compare it with the calibration curve of the encapsulated mRNA cargo. The size particle number per millilitre was measured using NanoSight Pro (Malvern Panalytical).

### pKa Determination by TNS Assay

The apparent pKa of PL@mBiME (DSPC) and PL@mBiME (DCPA) was determined using the hydrophobic fluorescent probe 2-(p-Toluidino)-6-naphthalenesulfonic acid (TNS). Nanoparticle dispersions were diluted to a constant lipid concentration in a citrate-sodium citrate buffer (20 mM) adjusted to pH values from 3.0 to 10.0 at 0.5-unit intervals. TNS was added to each sample at a final concentration of 10 µM. After incubation for 10 min at 25 °C, the TNS fluorescence intensity was measured (excitation 321 nm, emission 445 nm) using a fluorescence spectrophotometer. The background signal from buffer containing TNS was subtracted. The apparent pKa value was determined as the inflection point of the sigmoidal curve obtained by plotting the net fluorescence intensity versus pH. Data were fitted using the Boltzmann sigmoidal function.

### Cell culture

The murine glioma cell line GL261 (Cat# KGG2236-1) and the murine macrophage cell line RAW 264.7 (Cat# KGG2201-1) were obtained from KeyGEN Biotech Co., Ltd. (Jiangsu, China). The mouse brain endothelial cell line bEnd.3 (Cat# CL-0598) was obtained from Wuhan Procell Biotechnology Co., Ltd. (Wuhan, Hubei, China). The murine glioma cell line CT-2A (Cat# JY-Y1455) was obtained from Shanghai Jinyuan Biotechnology Co., Ltd. (Shanghai, China). GL261-Luc and CT-2A-Luc cells were generated by infecting cells with Lenti-EF1α-Luc-T2A-Puro virus (Banma Biotechnology Co., Ltd., Cat# BM2024012201) and selecting with 2 μg/mL puromycin (Beyotime, Cat# ST551-10mg). All cells were cultured in DMEM (HyClone) supplemented with 10% (v/v) fetal bovine serum (FBS; Gibco) and 100 U/mL penicillin/streptomycin (Invitrogen). Cell lines were authenticated by the vendors and were routinely tested for mycoplasma contamination. All cells used in this study were confirmed to be mycoplasma-negative.

### Acquisition of BMDM cells

C57 mice were killed by cervical dislocation to ensure that the animals died quickly and painlessly. Then the executed animals are placed in a clean workbench, and the femur and tibia of the limbs are carefully separated with sterile instruments to maintain bone integrity and remove impurities such as attached muscles and connective tissues. During the operation, attention should be paid to avoid contamination. Next, use a syringe to draw an appropriate amount of sterile PBS, carefully insert the needle into the marrow cavity opening at one end of the bone, slowly and steadily inject the buffer into the marrow cavity, allowing the marrow cells to be flushed out, and collect the buffer containing the marrow cells flowing from the other end into a sterile centrifuge tube. It can be rinsed several times to obtain as many bone marrow cells as possible.

### In vitro cellular uptake and mBiME transfection

For in vitro mBiME transfection assays, RAW 264.7 cells, BMDM cells, or GL261 cells were incubated with PBS, mBiME-T2A-EGFP, or PL@mBiME-T2A-EGFP, respectively. After 24 h of incubation, the percentage of EGFP-positive cells was analyzed using a flow cytometer (CytoFLEX, Beckman Coulter Life Sciences, Indianapolis, USA), and the data were processed with FlowJo software (version 10.8.1).

### Cell Cytotoxicity

GL261 and BMDM cells were inoculated into 96-well plates, respectively at a density of 1 × 10^4^ cells per well and incubated for 24 h with different concentrations of PBS, mBiME, L@mLuc, L@mBiME and PL@mBiME. Cell counting was performed using the EOS1 Cell Imaging System (Hiscore Inc). 20 μL of CCK-8 Assay Kit (CCK-8 Reagent: medium = 1:10, v/v) was added to each well and incubated in the dark for 1–4 h. Absorbance at 450 nm was measured using an enzyme marker (Multiskan Go, Thermo Fisher Scientific, Massachusetts, USA). Untreated control cells were considered 100% viable.

### Construction of Engineered GL261-Luc Cell Lines

GL261-OE-Luc Cell Line: The GL261-OE-Luc cell line was generated to stably overexpress mouse ErbB2 (mErbB2). GL261-Luc cells were transduced with the pLV3-CMV-mErbB2-3xFLAG-NEO lentiviral vector. Following transduction, stable polyclonal populations were selected and maintained in medium containing an appropriate concentration of G418 (1 mg/mL).

GL261-KO-Luc (Knockout) Cell Line: The GL261-KO-Luc cell line was generated using CRISPR-Cas9-mediated gene knockout. GL261 cells were transduced with the Lenti-CRISPRv2-NEO lentiviral vector, which expresses both the Cas9 nuclease and a single-guide RNA (sgRNA) targeting the gene of interest. The vector also carries a neomycin (NEO) resistance gene. After transduction, cells were selected with G418 (1 mg/mL) to obtain a polyclonal population harboring the knockout construct. The knockout efficiency was subsequently validated by immunoblotting analysis.

sgLdha#1: AGCTGGTCATTATCACCGCG

sgLdha#2: CTGCTGATCGTCTCCAATCC

### BMDM cells migration and polarization assay

The promotional effects of nanoparticals on BMDM cells migration were assessed by Transwell assay. 1 × 10^6^ BMDM cells were inoculated onto 8 μm polycarbonate membranes, GL261 tumor cells were spread on the lower layer, and the BMDM cells on the upper layer were treated with different preparations (PBS, mBiME, L@mLuc, L@mBiME, and PL@mBiME) treatment. Cells from the lower chamber were collected for flow analysis. After 12 h of incubation, BMDM cells migrating to the lower chamber were analyzed by flow cytometry and fluorescence microscopy, results are tabulated as the percentage of the total number of BMDM cells added. (BMDM cells were labeled with F4/80 flow antibody)

BMDM cells were laid in six-well plates, M-CSF was added to the cultures at a concentration of 20 ng/mL, followed by pre-stimulation with 10 ng/mL IL-4 to induce polarization into the M2 phenotype, then different preparations were added. Incubated for 24 h, cells were harvested, stained with F4/80, CD86, CD206 antibodies, and then analyzing BMDM cells polarization by flow cytometry.

### Conjugation and phagocytosis assay in vitro

BMDMs were co‑cultured with CT‑2A-Luc, GL261-Luc, or GL261‑OE-Luc cells and treated with PBS, PL@mRP182‑Luc, or PL@mBiME. To visualize the interaction between tumor cells and macrophages, tumor cells (CT‑2A-Luc, GL261-Luc, or GL261‑OE-Luc) were pre‑labeled with the green fluorescent DiO dye (Beyotime, Shanghai), and BMDMs were pre‑labeled with the red fluorescent DiI dye (Beyotime, Shanghai). After 12 h of co‑culture, macrophage‑tumor cell interactions were observed and imaged under a fluorescence microscope. Quantitative statistical analysis was performed using ImageJ.

For phagocytosis analysis, BMDMs were co-cultured with CT-2A-Luc cells, GL261-Luc cells and GL261-OE-Luc cells, treated with different preparations of PBS, PL@mRP182-Luc and PL@mBiME. After co-cultured for 24 h, the cells were harvested, stained with PE anti-mouse/human CD11b antibody (Biolegend, 101208), and then analyzed by flow cytometry. The percentage of phagocytic events in the CD11b^+^ population is plotted as a percentage of phagocytosis. For qualitative analysis of phagocytosis, tumor cells (CT‑2A-Luc, GL261-Luc, and GL261‑OE-Luc) were labeled with DiO dye (Green) (Shanghai Beyotime Company). Tumor cells and BMDMs were incubated at a ratio of 1:1 for 24 h at 37 °C.

For apoptosis analysis, cells were collected and analyzed using the Annexin V-APC/PI staining kit (Elabscience, E-CK-A217). Cells were resuspended in 500 μL PBS prior to examination using a CytoFLEX flow cytometer (Beckman Coulter, Inc., California, USA), and then data were analyzed using Flowjo. All antibodies used in this study are listed in Supplementary Table 4.

### Permeation study of brain targeting ability on BBB model

Mouse brain endothelial bEnd.3 and GL261 cells were inoculated into transwell upper and lower chambers (Corning, NY, USA), respectively, at a density of 2 × 10^5^ cells per well/mL in 6-well plates used for monolayer cell cultures. Then PBS, aPD-L1, mBiME, L@mBiME and PL@mBiME (Cy5-aPD-L1, Cy5-mBiME, L@Cy5-mBiME and PL@Cy5-mBiME) different treatments were added to the upper chamber and incubated for 4 h. The solution in the lower chamber was collected, and the fluorescence intensity was measured with an enzyme marker (Varioska LUX, Thermo Fisher Scientific, Massachusetts, USA).

### Biological distribution

To investigate the biological distribution, PBS, aPD-L1, mLuc, L@mLuc, and PL@mLuc were prepared using mLuc, Cy7-aPD-L1 and DSPE-PEG-Cy7 (L@mLuc, and PL@mLuc), and then C57BL/6 mice carrying GL261 tumors were injected intravenously with different formulations (mRNA equivalent dose of 1 mg kg^−^^1^), and near-infrared fluorescence imaging were performed at 0, 1, 2, 4, 8, 12, 24, 48 and 72 h after injection. Mice were sacrificed 12 h after injection to extract the heart, liver, spleen, lung, kidney, and brain for in vitro fluorescence imaging (Tanon ABL-X5, Shanghai, China).

To investigate mRNA expression in vivo, the following formulations were prepared: PL@mLuc without A2 and PL@mLuc with A2 in tumor-free mice, and PL@mLuc without A2, PL@mLuc (DSPC) with A2, and PL@mLuc with A2 in GL261-bearing mice. The mice were then administered the respective formulations intravenously at an mRNA-equivalent dose of 1 mg kg^−1^. Near-infrared bioluminescence imaging was performed at 0, 2, 4, 6, 8, 12, 24, 48, 72, 96, 120, and 144 h post-injection. At 12 h after injection, the mice were euthanized, and the heart, liver, spleen, lungs, kidneys, and brain were harvested for ex vivo bioluminescence imaging (IVIS Spectrum In Vivo Imaging System, Revvity). Luciferase mRNA was purchased from Novoprotein (Cat. No.: MR009; Novoprotein, Shanghai, China).

### Blood compatibility and biosafety of various treatments

To assess the impact of various preparations on mice, healthy mice were randomized into five groups and administered caudal vein injections of PBS, aPD-L1, mLuc, L@mLuc and PL@mLuc (mRNA equivalent dose of 1 mg kg^−1^), respectively, on day 7 and 14. The mice were anesthetized, and the whole blood of each group was collected through orbital blood collection, placed in an EP tube containing anticoagulant (EDTA), centrifuged at 3000 rpm at 4 °C for 15 min, and the upper serum was collected to detect the four biochemical indexes of ALT, AST, BUN and CREA. Then the mice were sacrificed, and the hearts, liver, spleen, lung and kidney of the mice were separated by perfusion with ice PBS and paraformaldehyde. The paraformaldehyde is fixed overnight. H&E staining was then performed according to the operation procedures. Images were obtained by ECLIPSE Ti2-U microscope (Nikon Corporation, Tokyo, Japan).

### Assessment of liver Kupffer cell flow cytometry

For evaluation of Kupffer cells in the liver, the following procedure was performed following three administrations of PL@mBiME. The mouse liver was harvested and digested in 750 µg/ml Collagenase IV (Gibco) at 37 °C for 1 h. The resulting cell suspension was treated with red blood cell lysis buffer for 5 minutes at room temperature, followed by centrifugation (360 × g, 5 min, 4 °C) to obtain a single-cell suspension. Cells were blocked with anti-CD16/32 for 15 min according to the manufacturer’s protocol, followed by staining with Zombie NIR™ Fixable Viability Kit for 20 minutes. Surface staining was performed using antibodies against CD45, CD11b, F4/80, CD86, and CD163 for 30 minutes in the dark. In addition, CD206 staining required an additional nuclear permeabilization step. Stained cells were analyzed on a flow cytometer (CytoFLEX, Beckman Coulter Life Sciences, Indianapolis, USA) to identify the Kupffer cell population. To assess mRNA expression in liver Kupffer cells, mice were injected with PL@mBiME-T2A-EGFP. Twelve hours later, livers were carefully harvested and processed into single-cell suspensions using the same digestion and lysis protocol described above. Cells were similarly blocked with anti-CD16/32 and stained with Zombie NIR™ viability dye. They were then incubated with antibodies against CD45, CD11b, and F4/80 for 30 min in the dark. Flow cytometric analysis was performed on the CytoFLEX to identify and analyze Kupffer cells for reporter expression.

### Evaluation of anti-tumor efficacy in vivo

An intracranial GBM mouse model was established by stereotactic inoculation of GL261 or GL261-Luc cells (1.5 × 10^5^ cells per mouse in 5 μL PBS) into the brains of mice (1 mm to the right, 2 mm forward, and 3 mm downward) anesthetized with inhaled 1 to 5% isoflurane mixed with oxygen. A 1:1 mixture by volume of GL261 or GL261-Luc tumor cells and Matrigel was injected gradually into the right hemisphere of the brain and the date of inoculation was designated as day 0. On day 8, mice were randomly assigned to receive the treatment through a tail vein. PBS (200 μL), aPD-L1 (5 mg kg^−^^1^), L@mLuc (1 mg kg^−^^1^ on an mLuc basis), PL@mRP182-Luc (1 or 5 mg kg^−^^1^ on an mRP182-Luc or aPD-L1 basis), L@mBiME (1 mg kg^−^^1^ on an mBiME basis), and PL@mBiME (1 or 5 mg kg^−^^1^ on an mBiME or aPD-L1 basis) were administered three times on day 8, day 11, and day 14. MR and IVIS are used to monitor tumor growth. The survival rate and body weight of mice were recorded, and the statistical significance of survival extension was analyzed by Log-rank test. Survival was defined as the time from orthotopic tumor inoculation until natural death of the animals.

After perfusing the brain tissue with 4% paraformaldehyde (PFA) solution and cold PBS, remove the tissue using ophthalmic scissors and forceps and soak it in PFA solution overnight. Then the paraffin-embedded sections were performed, and the brain sections were stained with H&E, Ki67, and TUNEL according to the manufacturer’s protocol to detect tumor size, tumor cell proliferation and tumor apoptosis. Then photos were taken using a fluorescence microscope and positive cells were counted by ImageJ.

An intracranial GBM mouse model was established by stereotaxically inoculating luciferase-expressing GL261-Luc, GL261-OE-Luc, GL261-KO-Luc, or CT-2A-Luc cells into the right brain hemisphere of anesthetized mice. Briefly, a 1:1 mixture of tumor cells (1.5 × 10⁵ cells in 5 μL PBS) and Matrigel was gradually injected at coordinates 1 mm lateral (right), 2 mm anterior, and 3 mm ventral to the bregma. The day of inoculation was designated as day 0. On day 8, mice were randomly assigned to receive the drug through a tail vein. PBS (200 μL), PL@mRP182-Luc (1 or 5 mg kg^−^^1^ on an mRP182-Luc or aPD-L1 basis) and PL@mBiME (1 or 5 mg kg^−^^1^ on an mBiME or aPD-L1 basis) were administered three times on day 8, day 11, and day 14 in GL261, GL261-OE-Luc or GL261-KO-Luc bearing-mice. PBS (200 μL), aPD-L1 (5 mg kg^−^^1^), L@mLuc (1 mg kg^−^^1^ on an mLuc basis), PL@mRP182-Luc (1 or 5 mg kg^−^^1^ on an mRP182-Luc or aPD-L1 basis), L@mBiME (1 mg kg^−^^1^ on an mBiME basis), and PL@mBiME (1 or 5 mg kg^−^^1^ on an mBiME or aPD-L1 basis) were administered three times on day 8, day 11, and day 14 in CT-2A-Luc bearing-mice. Tumor growth was monitored in vivo using IVIS imaging. The survival rate and body weight of mice were recorded, and the statistical significance of survival extension was analyzed by Log-rank test. Survival was defined as the time from orthotopic tumor inoculation until natural death of the animals.

### In vivo bioluminescence imaging

The intensity of bioluminescence in GL261-Luc, GL261-OE-Luc, GL261-KO-Luc, and CT-2A-Luc tumor-bearing mice was quantitatively measured with an IVIS (Tanon ABL-X5, Shanghai, China). After administering 100 μL of D-Luciferin solution (15 mg/ml in PBS) via intraperitoneal injection, images were captured 5 to 10 min later and analyzed using Tanon software (Tanon, Shanghai, China). D-Luciferin potassium salt was kindly provided by Nanjing Starleaf Biological Technology Co., Ltd (Jiangsu, China).

### Assessment of cognitive function by Morris water maze test

To assess the potential impact of PL@mBiME on higher-order brain function, spatial learning and memory were evaluated in GL261 glioma-bearing mice using the Morris water maze (MWM) test. Each experimental group consisted of five animals, including PL@mBiME, Control (with tumor volume matched to the PL@mBiME group via pre-test imaging to control for tumor burden effects), PBS and other relevant treatment control groups. The MWM was performed in a circular pool (120 cm diameter) filled with opaque water maintained at 25 ± 1 °C, containing a hidden escape platform positioned in a constant quadrant. Mouse behavior was recorded and analyzed using the Supermaze behavioral tracking system. During the six-day acquisition phase, all groups underwent daily training trials in the maze. On day 7, a probe trial was conducted with the platform removed, during which the time spent in the target quadrant and the number of platform crossings were recorded.

### Cell conjugation assay in vivo

To evaluate the in vivo conjugation between tumor cells and macrophages, tumor-bearing mice (CT-2A-Luc, GL261-Luc, and GL261-OE-Luc) were treated with PBS, PL@mRP182-Luc, or PL@mBiME. Brain tissues were carefully harvested for immunofluorescence staining. Following euthanasia, tumor tissues were collected, fixed in 4% paraformaldehyde, paraffin-embedded, and sectioned. Tissue sections were stained for F4/80 and ErbB2 (blue: DAPI; red: SpOrange-F4/80; green: SpGreen-ErbB2). Images were acquired using fluorescence microscopy, and quantitative analysis was performed with ImageJ.

### Assessment of changes in the immune microenvironment in vivo

For flow tissue testing, the brain tissue is carefully removed and cleaned with PBS. Collagenase D (0.5 μg/mL), DNase1 (0.5 μg/mL from Vazyme Biotec Co., Ltd, China; 3 μg/mL from Sigma Aldrich, USA), and a lymphocyte isolation solution (17-5442-03, GE, USA) were used to isolate brain-infiltrating immune cells. Afterwards, ACK Lysis Buffer (#R1013, Beijing Solarbio Science & Technology Co., Ltd.) was added for centrifugation to remove red blood cells. The mouse tumor-infiltrating tissue lymphocyte extraction kit was then used to obtain a total of 1 ×106 cells in each tube according to the instructions, and then closed with CD16/32 for 15 minutes. Then stain using Zombie NIR™ Fixable Viability Kit for 20 min, after which CD45, CD3, CD4, CD8, CD11b, CD11c, F4/80, CD25, CD86, MHCII, Gr-1, and NK1.1 were added to cells and incubated in the dark for half an hour. In order to stain CD206 and Foxp3, additional nucleoclast staining is required. Thereafter, stained cells were analyzed by flow cytometry (FACS Celesta, Becton, Dickinson and Company, New Jersey, USA) for macrophages, CD8^+^ T cells, Tregs, mature DC, NK, and MDSC populations.

For immunofluorescence staining, mice were sacrificed and tumor tissue was removed, further fixed in 4% paraformaldehyde, embedded in paraffin, and then cut into slides. To evaluate M1 macrophages in GBM, tumor sections were labeled as F4/80 and CD86 (blue: DAPI; red: CY3; green: Alexa fluor 488). To evaluate M2 macrophages in GBM, tumor sections were labeled as F4/80 and CD206 (blue: DAPI; red: CY3; green: Alexa fluor 488). To evaluate CD8^+^ T cells in GBM, tumor sections were labeled as CD4, and CD8 (blue: DAPI; green: SpGreen-CD4; purple: Cy5-CD8). Images were captured using a fluorescence microscope and subsequently analyzed for fluorescence intensity with ImageJ software.

To detect the expression of relevant inflammatory factors in brain tissue, tumor tissues were collected, homogenized, and centrifuged. The secretion of IFN-γ,, IL-10, IL-1-β and TNF-α in brain tissue were measured using an ELISA kit according to manufacturer’s instructions.

### Detection of mBiME and aPD-L1 in liver and brain tissues

To detect mBiME-T2A-EGFP expression, liver and brain tissues were harvested 12 h post-injection of PL@mBiME-T2A-EGFP, fixed in 4% paraformaldehyde, and embedded in paraffin. Section (5 μm) were subjected to antigen retrieval in citrate buffer (pH 6.0). After blocking with 5% BSA, sections were incubated overnight at 4 °C with a primary anti-GFP antibody (Abcam, #ab290, 1:500), followed by an Alexa Fluor 488-conjugated secondary antibody (Invitrogen, 1:1000) for 1 h at room temperature. Nuclei were counterstained with DAPI. Slides were scanned using a whole-slide scanner, and images were analyzed with ImageJ software.

To assess the aPD-L1 antibody, liver and brain tissues were harvested from mice at 12 h post-injection of PL@mBiME. Tissues were perfused with PBS, fixed in 4% paraformaldehyde, and paraffin-embedded. Section (5 μm) underwent antigen retrieval in EDTA buffer (pH 9.0). After blocking, sections were incubated with Alexa Fluor 594-conjugated anti-rat IgG for 1 h at room temperature. Nuclei were stained with DAPI. Images were captured using a fluorescence microscope and subsequently analyzed for fluorescence intensity with ImageJ software.

### Macrophage antigen presentation and SIRPα evaluation

Pre-stimulated M2-type bone marrow-derived macrophages (BMDM) were co-cultured with labeled GL261 cells in a 1:1 ratio in six-well plates and treated with different formulations (PBS, mBiME, L@mLuc, L@mBiME, and PL@mBiME). After 12 h of co-culture, cells were collected and stained with Zombie NIR™ Fixable Viability Kit, anti-CD45 antibody, anti-CD11b antibody, anti-F4/80 antibody, anti-MHCII antibody, and anti-SIRPα antibody. Cells stained for MHCII and SIRPα were analyzed on a flow cytometer (CytoFLEX, Beckman Coulter Life Sciences, Indianapolis, USA), and the data was processed using FlowJo software (version 10.8.1).

### Macrophage and CD8 T cell depletion assay in vivo

For macrophage depletion, clodronate liposomes (Clone HY-172202, MedChemExpress) were administered intravenously (200 μL/mouse), with initial treatment on day 7 followed by subsequent doses every 3 days (day 10, 13, and 16) to ensure continuous depletion of newly recruited macrophages. All depletion efficacies were validated by flow cytometry (CytoFLEX, Beckman Coulter Life Sciences, Indianapolis, USA).

To deplete CD8 T cells, mice in the treatment group were injected with anti-mouse CD8α antibody (Clone 2.43, BioXCell; 100 μg/mouse) two days before the first administration of formulations. Additional doses were administered on days 5 and 12 post-initial formulations to ensure effective depletion throughout the treatment period. This approach allowed for the assessment of the role of CD8 T cells in the anti-tumor immune response induced by the formulations.

### Evaluation of long-term immune memory in vivo

To analyze long-term memory in vivo, mice treated with PL@mBiME were selected on day 45 of inoculation for further tumor rechallenge studies. On day 65 after tumor inoculation, MR monitoring was performed to record the survival time, and the statistical significance of prolonged survival time was analyzed by Log-rank test. Afterwards, three PL@mBiME-treated mice were selected for flow cytometry analysis of central memory T cells and effector memory T cells on day 65, and the brain, dLNs, and spleen were removed and stained with anti-CD3, anti-CD8, anti-CD44, and anti-CD62L antibodies. Subsequently, the stained cells were analyzed by flow cytometry (CytoFLEX, Beckman Coulter Life Sciences, Indianapolis, USA) and the data was processed with FlowJo software (version 10.8.1). All antibodies are used according to manufacturer’s instructions. The fluorescent dye conjugated on the antibody is an exact match to the same fluorescent dye channel.

### Magnetic resonance imaging (MRI)

7.0 Tesla Small Animal MRI (Bruker Pharmascan, Ettlingen, Germany) was used to detect tumor growth in tumor-bearing mice. The mice were anesthetized with 1% isoflurane. Coil parameters are the spin-echo sequence (repetition time/echo time, 2000/50 ms; matrix, 256×  256; the field of view, 20 × 20 mm; slice thickness, 1.0 mm). The parameters of T2-weighted images were as follows: TR = 3000 ms, TE = 36 ms; matrix size = 256 × 256; field of view = 2.0 × 2.0 cm; and slice thickness = 1 mm. Then RadiAnt DICOM Viewer was used to analysis the images. Tumor volume was measured by MRI. Specifically, the tumor volume was calculated by summing the tumor area measured on each scanned slice and multiplying by the slice interval (thickness) to obtain the total volume.

### Statistics & Reproducibility

All statistical analyses were performed by Origin software version 2026 (OriginLab Corporation, Northampton, Massachusetts, USA). Statistical evaluation was performed with the Student’s t test and one-way or two-way analysis of variance (ANOVA). Survival was analyzed using log-rank tests. All values were described as the means ± standard deviation (SD), and differences were considered as statistically significant at *P* < 0.05. All experiments were repeated for at least three times and experimental findings were reproducible.

### Reporting summary

Further information on research design is available in the Nature Portfolio Reporting Summary linked to this article.

## Supplementary information

Supplementary Information

Reporting Summary

Transparent Peer Review file

## Source data

Source Data

## Data Availability

The data that support the findings of this study are available in the supplementary material of this article. Source data are provided with this paper.

## References

[1] Jacob, F. et al. A Patient-Derived Glioblastoma Organoid Model and Biobank Recapitulates Inter- and Intra-tumoral Heterogeneity. *Cell* **180**, 188–204.e122 (2020). 31883794 10.1016/j.cell.2019.11.036 PMC7556703

[2] Rong, L., Li, N. & Zhang, Z. Emerging therapies for glioblastoma: current state and future directions. *J. Exp. Clin. Cancer Res.* **41**, 142 (2022). 35428347 10.1186/s13046-022-02349-7 PMC9013078

[3] Stupp, R. et al. Radiotherapy plus Concomitant and Adjuvant Temozolomide for Glioblastoma. *N. Engl. J. Med.* **352**, 987–996 (2005). 15758009 10.1056/NEJMoa043330

[4] Mellman, I., Coukos, G. & Dranoff, G. Cancer immunotherapy comes of age. *Nature* **480**, 480–489 (2011). 22193102 10.1038/nature10673 PMC3967235

[5] Banks, W. A. From blood–brain barrier to blood–brain interface: new opportunities for CNS drug delivery. *Nat. Rev. Drug Discov.* **15**, 275–292 (2016). 26794270 10.1038/nrd.2015.21

[6] Bechmann, I., Galea, I. & Perry, V. H. What is the blood–brain barrier (not)?. *Trends Immunol.* **28**, 5–11 (2007). 17140851 10.1016/j.it.2006.11.007

[7] DeCordova, S. et al. Molecular Heterogeneity and Immunosuppressive Microenvironment in Glioblastoma. *Front. Immunol.* **11**, 1402 (2020). 32765498 10.3389/fimmu.2020.01402 PMC7379131

[8] Jackson, C. M., Lim, M. & Drake, C. G. Immunotherapy for Brain Cancer: Recent Progress and Future Promise. *Clin. Cancer Res.* **20**, 3651–3659 (2014). 24771646 10.1158/1078-0432.CCR-13-2057 PMC4729210

[9] Arvanitis, C. D., Ferraro, G. B. & Jain, R. K. The blood–brain barrier and blood–tumour barrier in brain tumours and metastases. *Nat. Rev. Cancer* **20**, 26–41 (2019). 31601988 10.1038/s41568-019-0205-x PMC8246629

[10] Quail, D. F. & Joyce, J. A. The Microenvironmental Landscape of Brain Tumors. *Cancer Cell* **31**, 326–341 (2017). 28292436 10.1016/j.ccell.2017.02.009 PMC5424263

[11] Chen, C. et al. Intracavity generation of glioma stem cell–specific CAR macrophages primes locoregional immunity for postoperative glioblastoma therapy. *Sci. Transl. Med.* **14**, eabn1128 (2022). 35921473 10.1126/scitranslmed.abn1128

[12] Xuan, W., Lesniak, M. S., James, C. D., Heimberger, A. B. & Chen, P. Context-Dependent Glioblastoma–Macrophage/Microglia Symbiosis and Associated Mechanisms. *Trends Immunol.* **42**, 280–292 (2021). 33663953 10.1016/j.it.2021.02.004 PMC8005482

[13] Simonds, E. F. et al. Deep immune profiling reveals targetable mechanisms of immune evasion in immune checkpoint inhibitor-refractory glioblastoma. *J. Immunother. Cancer* **9**, e002181 (2021). 34083417 10.1136/jitc-2020-002181 PMC8183210

[14] Rolin, C., Zimmer, J. & Seguin-Devaux, C. Bridging the gap with multispecific immune cell engagers in cancer and infectious diseases. *Cell Mol. Immunol.* **21**, 643–661 (2024). 38789528 10.1038/s41423-024-01176-4 PMC11214628

[15] Fenis, A., Demaria, O., Gauthier, L., Vivier, E. & Narni-Mancinelli, E. New immune cell engagers for cancer immunotherapy. *Nat. Rev. Immunol.* **24**, 471–486 (2024). 38273127 10.1038/s41577-023-00982-7

[16] Bucci, L. et al. Bispecific T cell engager therapy for refractory rheumatoid arthritis. *Nat. Med.* **30**, 1593–1601 (2024). 38671240 10.1038/s41591-024-02964-1

[17] Xu, L. et al. Targeting CD89 on tumor-associated macrophages overcomes resistance to immune checkpoint blockade. *J. Immunother. Cancer* **10**, e005447 (2022). 10.1136/jitc-2022-005447 PMC9723960 36460336

[18] Li, B. et al. CD89-mediated recruitment of macrophages via a bispecific antibody enhances anti-tumor efficacy. *Oncoimmunology* **7**, e1380142 (2017). 29296544 10.1080/2162402X.2017.1380142 PMC5739557

[19] Pombo Antunes, A. R. et al. Single-cell profiling of myeloid cells in glioblastoma across species and disease stage reveals macrophage competition and specialization. *Nat. Neurosci.* **24**, 595–610 (2021). 33782623 10.1038/s41593-020-00789-y

[20] Jaynes, J. M. et al. Mannose receptor (CD206) activation in tumor-associated macrophages enhances adaptive and innate antitumor immune responses. *Sci. Transl. Med.* **12**, eaax6337 (2020). 10.1126/scitranslmed.aax6337 PMC7832040 32051227

[21] Liu, G. et al. HER-2, gp100, and MAGE-1 are expressed in human glioblastoma and recognized by cytotoxic T cells. *Cancer Res* **64**, 4980–4986 (2004). 15256472 10.1158/0008-5472.CAN-03-3504

[22] Ahmed, N. et al. HER2-specific T cells target primary glioblastoma stem cells and induce regression of autologous experimental tumors. *Clin. Cancer Res* **16**, 474–485 (2010). 20068073 10.1158/1078-0432.CCR-09-1322 PMC3682507

[23] Schlake, T. et al. mRNA: A Novel Avenue to Antibody Therapy?. *Mol. Ther.* **27**, 773–784 (2019). 30885573 10.1016/j.ymthe.2019.03.002 PMC6453519

[24] Van Hoecke, L. & Roose, K. How mRNA therapeutics are entering the monoclonal antibody field. *J. Transl. Med* **17**, 54 (2019). 30795778 10.1186/s12967-019-1804-8 PMC6387507

[25] Zhao, Y. et al. Polymer-locking fusogenic liposomes for glioblastoma-targeted siRNA delivery and CRISPR-Cas gene editing. *Nat. Nanotechnol.* **19**, 1869–1879 (2024). 39209994 10.1038/s41565-024-01769-0

[26] Zhang, Z. et al. Redox-responsive polymer micelles co-encapsulating immune checkpoint inhibitors and chemotherapeutic agents for glioblastoma therapy. *Nat. Commun.* **15**, 1118 (2024). 38320994 10.1038/s41467-024-44963-3 PMC10847518

[27] Xu, X. et al. Recruiting T-Cells toward the Brain for Enhanced Glioblastoma Immunotherapeutic Efficacy by Co-Delivery of Cytokines and Immune Checkpoint Antibodies with Macrophage-Membrane-Camouflaged Nanovesicles. *Adv. Mater.* **35**, e2209785 (2023). 37101060 10.1002/adma.202209785

[28] Zhao, S. et al. Acid-degradable lipid nanoparticles enhance the delivery of mRNA. *Nat. Nanotechnol.* **19**, 1702–1711 (2024). 39179796 10.1038/s41565-024-01765-4 PMC12479011

[29] Wang, D. Y. et al. Liposomes with Water as a pH-Responsive Functionality for Targeting of Acidic Tumor and Infection Sites. *Angew. Chem. Int Ed. Engl.* **60**, 17714–17719 (2021). 34028150 10.1002/anie.202106329 PMC8362074

[30] Elda Valenti, G., Tasso, B., Traverso, N., Domenicotti, C. & Marengo, B. Glutathione in cancer progression and chemoresistance: an update. *Redox Exp. Med.* **2023**, e220023 (2023).

[31] Niu, B. et al. Application of glutathione depletion in cancer therapy: Enhanced ROS-based therapy, ferroptosis, and chemotherapy. *Biomaterials* **277**, 121110 (2021). 34482088 10.1016/j.biomaterials.2021.121110

[32] Semple, S. C. et al. Rational design of cationic lipids for siRNA delivery. *Nat. Biotechnol.* **28**, 172–176 (2010). 20081866 10.1038/nbt.1602

[33] Alabi, C. A. et al. Multiparametric approach for the evaluation of lipid nanoparticles for siRNA delivery. *Proc. Natl. Acad. Sci. USA* **110**, 12881–12886 (2013). 23882076 10.1073/pnas.1306529110 PMC3740846

[34] Li, X., Zhao, L., Li, W., Gao, P. & Zhang, N. HER2-targeting CAR-T cells show highly efficient anti-tumor activity against glioblastoma both in vitro and in vivo. *Genes Immun.* **25**, 201–208 (2024). 38702509 10.1038/s41435-024-00275-6 PMC11178492

[35] Khalsa, J. K. et al. Immune phenotyping of diverse syngeneic murine brain tumors identifies immunologically distinct types. *Nat. Commun.* **11**, 3912 (2020). 32764562 10.1038/s41467-020-17704-5 PMC7411074

[36] Noffsinger, B. et al. Technical choices significantly alter the adaptive immune response against immunocompetent murine gliomas in a model-dependent manner. *J. Neurooncol* **154**, 145–157 (2021). 34432197 10.1007/s11060-021-03822-7 PMC9277914

[37] Wouters, R., Bevers, S., Riva, M., De Smet, F. & Coosemans, A. Immunocompetent Mouse Models in the Search for Effective Immunotherapy in Glioblastoma. *Cancers (Basel)* **13**, 19 (2020). 10.3390/cancers13010019 PMC7793150 33374542

[38] Stadler, C. R. et al. Preclinical efficacy and pharmacokinetics of an RNA-encoded T cell-engaging bispecific antibody targeting human claudin 6. *Sci. Transl. Med* **16**, eadl2720 (2024). 38776391 10.1126/scitranslmed.adl2720

[39] Stadler, C. R. et al. Elimination of large tumors in mice by mRNA-encoded bispecific antibodies. *Nat. Med* **23**, 815–817 (2017). 28604701 10.1038/nm.4356

[40] Huang, C. et al. Lipid Nanoparticle Delivery System for mRNA Encoding B7H3-redirected Bispecific Antibody Displays Potent Antitumor Effects on Malignant Tumors. *Adv. Sci. (Weinh.)* **10**, e2205532 (2023). 36403209 10.1002/advs.202205532 PMC9875623

[41] Huang, Y. et al. Organ-specific delivery of an mRNA-encoded bispecific T cell engager targeting glypican-3 in hepatocellular carcinoma. *Nat. Commun.* **16**, 11111 (2025). 41397962 10.1038/s41467-025-66087-y PMC12705655

[42] Hou, X., Zaks, T., Langer, R. & Dong, Y. Lipid nanoparticles for mRNA delivery. *Nat. Rev. Mater.* **6**, 1078–1094 (2021). 34394960 10.1038/s41578-021-00358-0 PMC8353930

[43] Voissière, A. et al. The CSF-1R inhibitor pexidartinib affects FLT3-dependent DC differentiation and may antagonize durvalumab effect in patients with advanced cancers. *Sci. Transl. Med.* **16**, eadd1834 (2024). 38266104 10.1126/scitranslmed.add1834

[44] Quail, D. F. et al. The tumor microenvironment underlies acquired resistance to CSF-1R inhibition in gliomas. *Science* **352**, aad3018 (2016). 27199435 10.1126/science.aad3018 PMC5450629

[45] Bowman, R. L. et al. Macrophage Ontogeny Underlies Differences in Tumor-Specific Education in Brain Malignancies. *Cell Rep.* **17**, 2445–2459 (2016). 27840052 10.1016/j.celrep.2016.10.052 PMC5450644

[46] Muller, S. et al. Single-cell profiling of human gliomas reveals macrophage ontogeny as a basis for regional differences in macrophage activation in the tumor microenvironment. *Genome Biol.* **18**, 234 (2017). 29262845 10.1186/s13059-017-1362-4 PMC5738907

[47] Chen, M. Z. et al. A versatile antibody capture system drives specific in vivo delivery of mRNA-loaded lipid nanoparticles. *Nat. Nanotechnol.* **20**, 1273–1284 (2025). 40759744 10.1038/s41565-025-01954-9 PMC12443633

