# Supplementary material for: Co-delivering macrophage engager mRNA and PD-L1 antibody via tumor-responsive nanoparticles for glioblastoma immunotherapy
Source: Nat Commun. 2026 Apr 11;17:5127. doi: 10.1038/s41467-026-71646-y (PMC13247073; doi:10.1038/s41467-026-71646-y)
Supplement: Supplementary file 1 — Supplementary Information [file 41467_2026_71646_MOESM1_ESM.pdf]

SUPPLEMENTARY INFORMATION

Co-delivering macrophage engager mRNA and PD-L1 antibody via tumor-responsive nanoparticles for glioblastoma immunotherapy

Haoge Zhang[1,2,6], Jia Miao[2,6], Lin Gao[2,6], Xuhong Yang[2], Zhengcheng Yun[2], Lei Dong[2], Wanqing Cheng[2], Yuqi Wang[2], Hui Yang[3], Ying Zhou[4], Yini Zhu[4,5] and Jinbing Xie[1,2*]*

H. Zhang, J. Xie

[1] Department of Radiology, Nantong First People's Hospital, School of Medicine, Southeast University, Nantong 226001, China

H. Zhang, J. Miao, L. Gao, X. Yang, Z. Yun, L. Dong, W. Cheng, Y. Wang, J. Xie

[2] Nurturing Center of Jiangsu Province for State Laboratory of AI Imaging & Interventional Radiology, Zhongda Hospital, Southeast University, Nanjing 210009, China

H. Yang

[3] Department of Biochemistry and Molecular Biology, School of Medicine, Southeast University, Nanjing 210009, China

Y. Zhou, Y. Zhu

[4] Department of Microbiology and Immunology, School of Medicine, Southeast University, Nanjing 210009, China

Y. Zhu

[5] Department of Urology, Nantong First People's Hospital, School of Medicine, Southeast University, Nantong 226001, China

[6]These authors contributed equally: Haoge Zhang, Jia Miao and Lin Gao

*Correspondence to: xiejb@seu.edu.cn (J. Xie); yzhu15@seu.edu.cn (Y. Zhu)

Table of contents

1. Supplementary Figures

2. Supplementary Tables

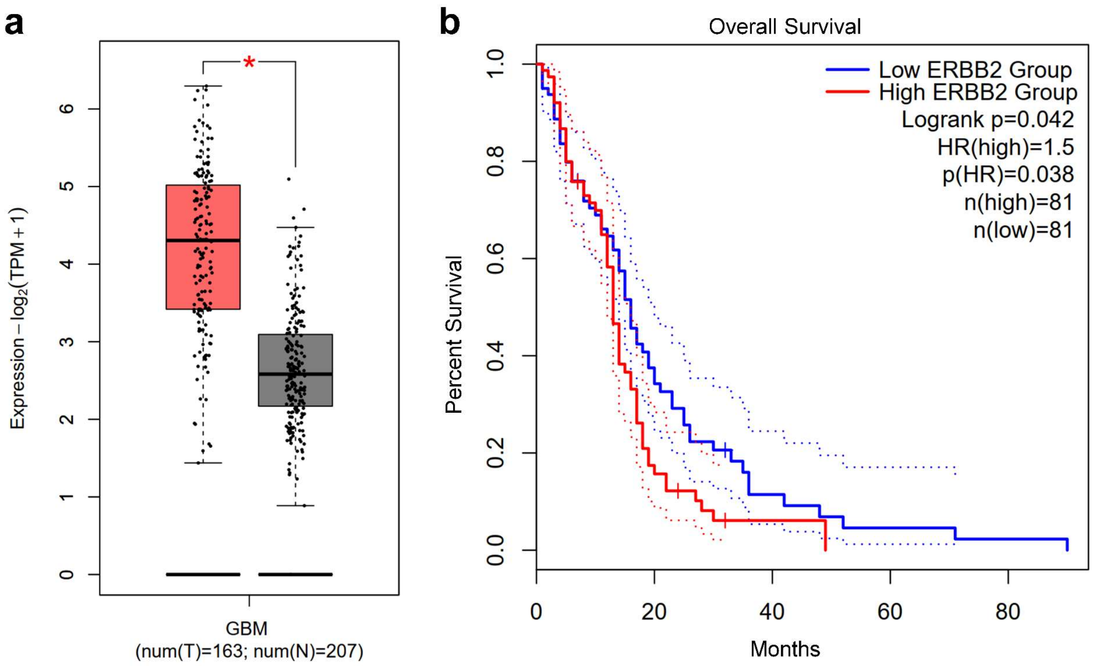

Supplementary Fig. 1. Association between ErbB2 and prognosis in patients with GBM. a Expression of ErbB2 gene was significantly increased (**P* < 0.05) in GBM subtypes (Left) compared to normal subjects (Right). **b** In GBM, patients with low expression of ErbB2 had significantly higher recurrence survival and overall survival than patients with high expression of ErbB2. Mining for information in the TCGA databases via http://gepia2.cancer-pku.cn/.

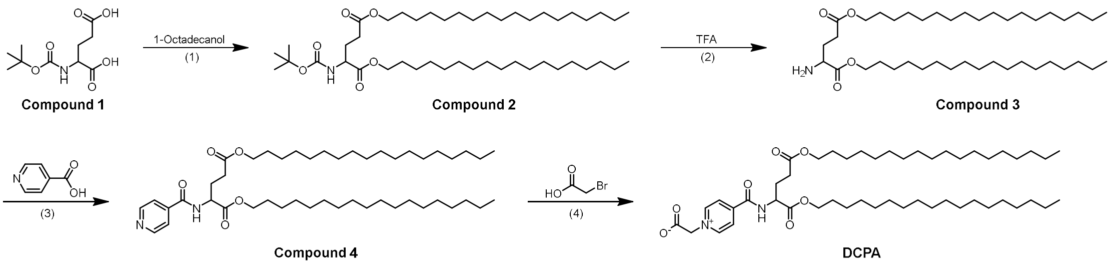

Supplementary Fig. 2. Synthetic route of DCPA. (1) 1-Octadecanol, DMAP, DCC, DCM, 0-25 °C, overnight, yield 90.5%; (2) TFA, 0-25 °C, for 1h, yield 97.0%; (3) p-pyridinecarboxylic acid, HATU, DIEA, DMF, 0-65 °C, for 2 h, yield 97.7%; (4) bromoacetic acid, acetonitrile, reflux for 24 h, yield 79.3%.

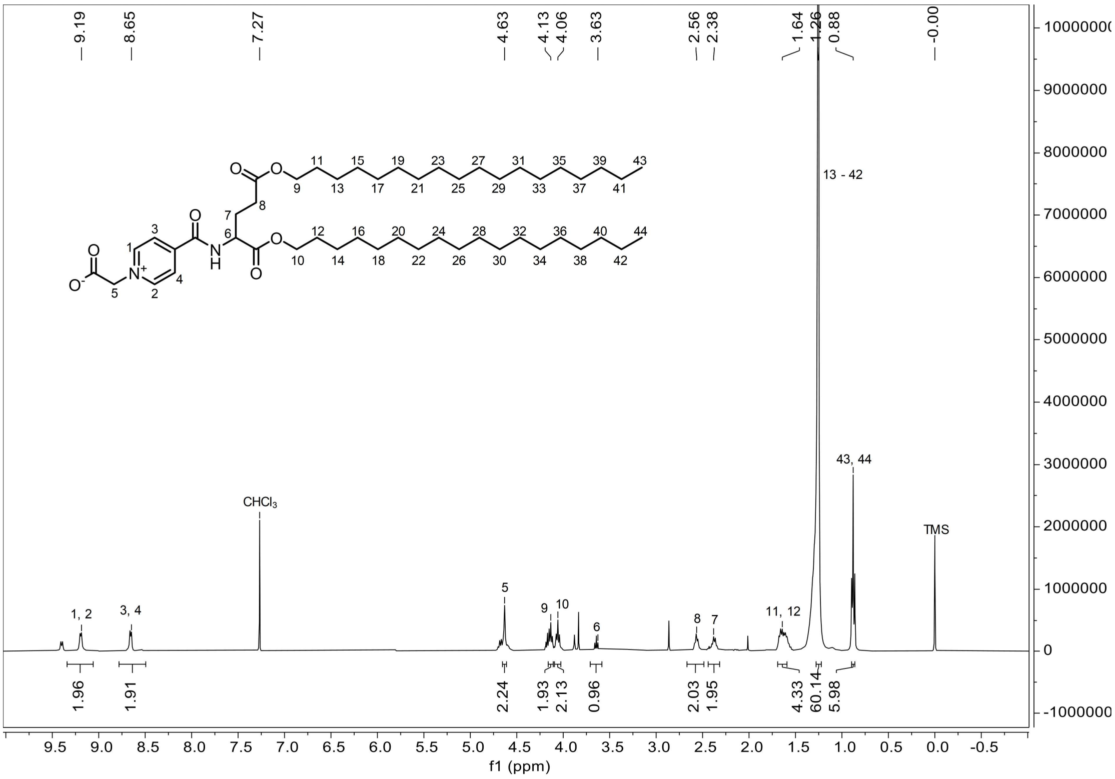

Supplementary Fig. 3. Structural characterization of DCPA. The ^{1}H nuclear magnetic resonance (NMR) spectrum of DCPA in $CDCl_3$, confirming its chemical structure.

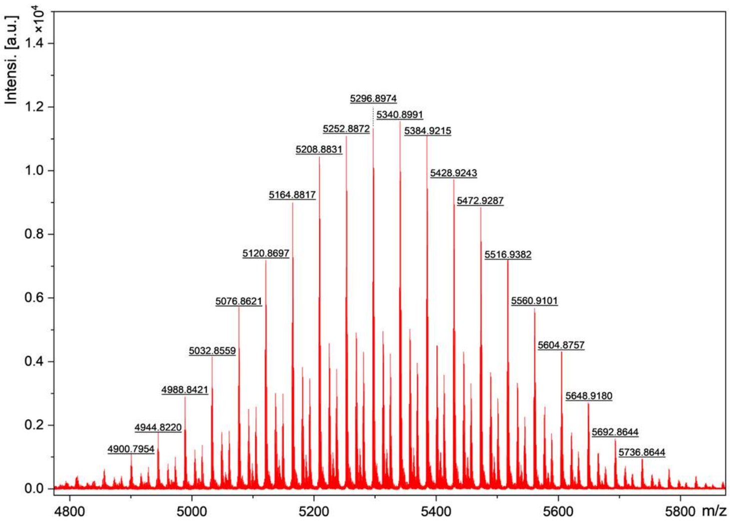

Supplementary Fig. 4. Verification of the covalent conjugation of DSPE-PEG-A2. The matrix assisted laser desorption ionization time of flight mass spectrometry (MALDI-TOF-MS) spectrum of DSPE-PEG-A2.

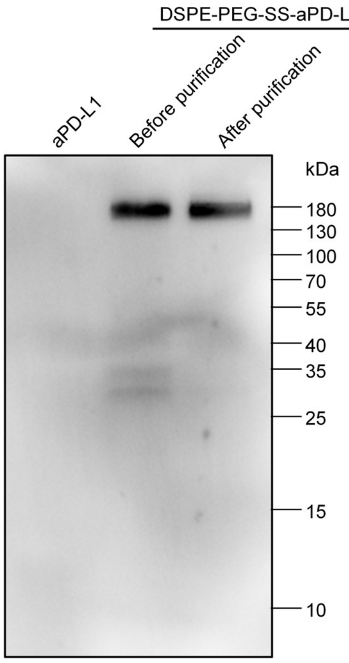

Supplementary Fig. 5. Successful PEGylation of aPD-L1 confirmed by Western blot. Following SDS-PAGE separation, protein blotting was performed using an anti-PEG antibody (PEG-B-47, Abcam), which specifically detected the aPD-L1-PEG conjugate.

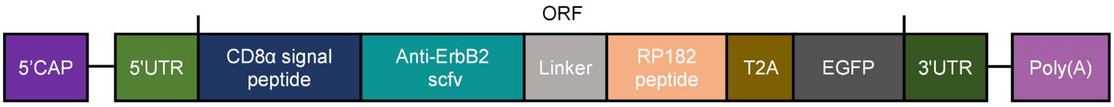

Supplementary Fig. 6. Diagram of a mBiME-T2A-EGFP. The mRNA sequence encodes a construct incorporating the CD8α signal peptide, an anti-ErbB2 single-chain variable fragment (scFv), the RP182 peptide, and enhanced green fluorescent protein (EGFP).

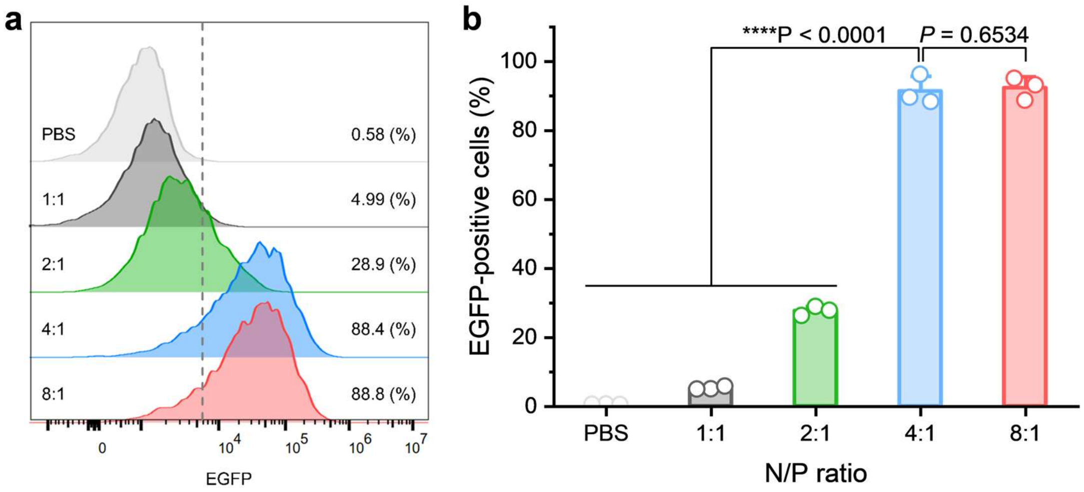

Supplementary Fig. 7. Transfection efficiency of PL@mBiME-T2A-EGFP in RAW 264.7 cells at different N/P ratios. a Representative flow cytometry histograms showing EGFP expression. **b** Quantitative analysis of the percentage of EGFP-positive cells from (a). Data are presented as mean ± SD, n = 3 independent experiments; one-way ANOVA with Fisher's LSD post-hoc test.

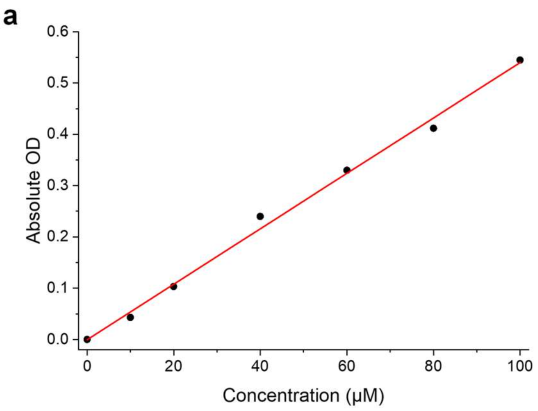

b

Sample	1	2	3
OD	0.253	0.274	0.243
Dilute ratio	1:100	1:100	1:100
Concentration (mM)	4.68	5.07	4.50

Supplementary Fig. 8. Quantification of glutathione (GSH) concentration. a Standard curve for the GSH assay kit. Serial dilutions of a known GSH standard were used to generate the curve for calculating sample concentrations. **b** Quantified GSH concentrations in brain with tumor, as determined using the standard curve from panel (a).

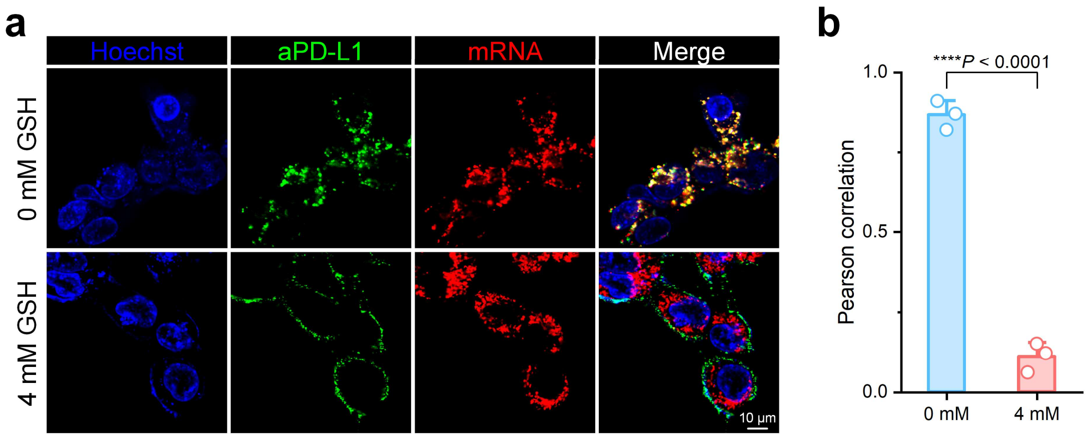

Supplementary Fig. 9. GSH-triggered aPD-L1 release of PL@mBiME. a Intracellular distribution of FITC-labeled aPD-L1 (green) and Cy5-labeled mBiME (red) in GL261 cells after 4 h, with 0 mM or 4 mM GSH. Scale bar, 10 μm. **b** Pearson's correlation analysis confirms GSH-responsive carrier disassembly (reduced co-localization) at high GSH. Data are presented as mean ± SD, n = 3 independent experiments; unpaired two-tailed Student's t-test.

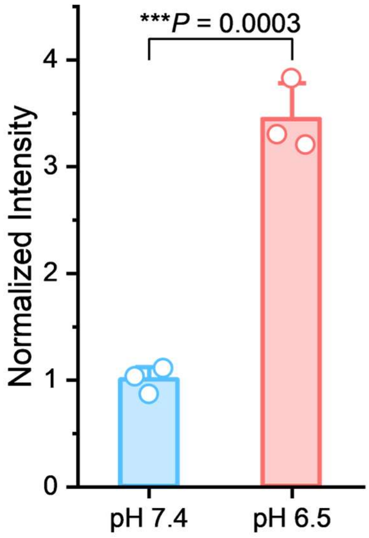

Supplementary Fig. 10. Quantification of PL@mBiME uptake by RAW264.7 cells at pH 7.4 and 6.5. Corresponding quantitative analysis of the PL@mBiME fluorescence intensity from the images in Fig. 2a. Data are presented as mean ± SD, n = 3 independent experiments; unpaired two-tailed Student's t-test.

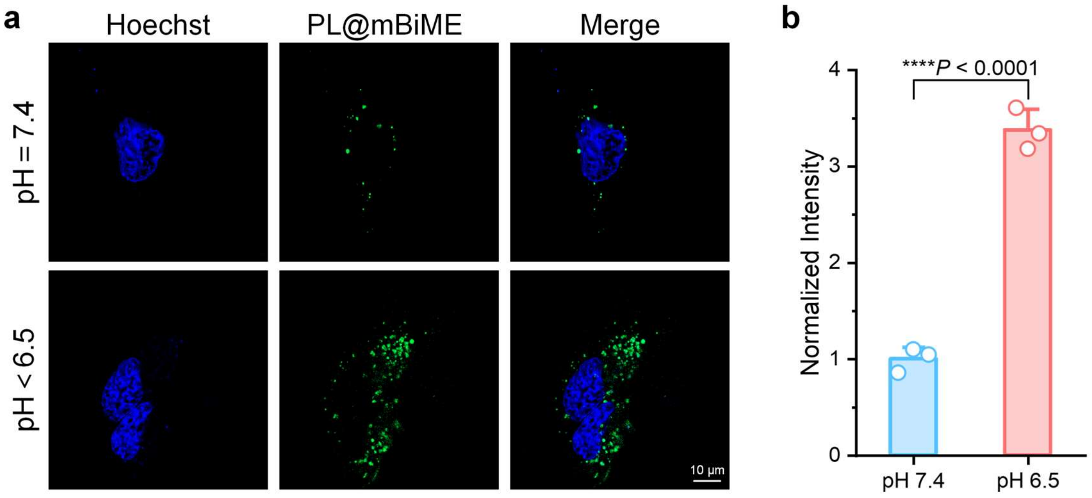

Supplementary Fig. 11. Cellular uptake of PL@mBiME by GL261 cells under different pH conditions. a Representative fluorescence microscopy images showing cellular uptake at pH 7.4 and 6.5. PL@mBiME (green) and nuclei (blue, Hoechst 33342). Scale bar, 10 μm. **b** Corresponding quantitative analysis of the PL@mBiME fluorescence intensity from the images in (a). Data are presented as mean ± SD, n = 3 independent experiments; unpaired two-tailed Student's t-test.

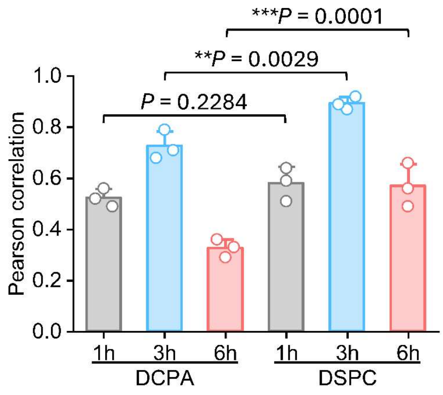

Supplementary Fig. 12. Quantification analysis of Pearson correlation coefficients. Pearson correlation of Lyso-Track green and Cy5-mBiME in DCPA-PL@mBiME group and DSPC-PL@mBiME group at 1h, 3h, and 6h, n = 3 independent experiments. Data are presented as mean ± SD, statistical analysis was performed by two-way ANOVA with Fisher's LSD post-hoc test.

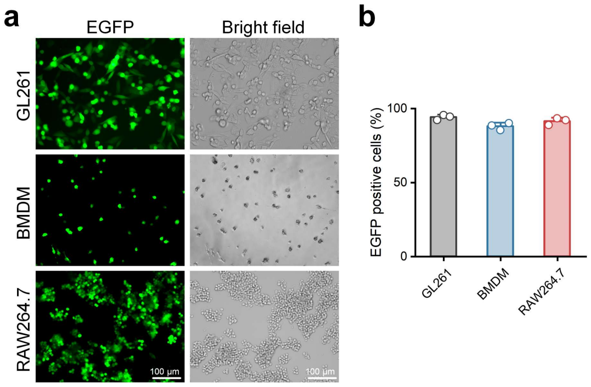

Supplementary Fig. 13. Determination of cellular EGFP transfection efficiency. a Results of GL261, BMDM and RAW264.7 transfection using PL@mBiME-T2A-EGFP at pH 6.5, images representative of 3 experiments. Scale bar = 100 μm. **b** Quantification of EGFP-positive GL261, BMDMs, and RAW264.7 cells by Image J at 24 h. Data are presented as mean ± SD, n = 3 independent experiments.

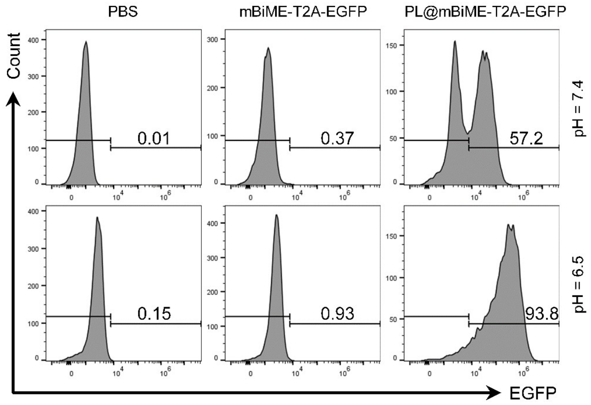

Supplementary Fig. 14. Flow cytometric analysis of EGFP transfection efficiency. Flow cytometry histogram of PL@mBiME-T2A-EGFP transfection in GL261 cells.

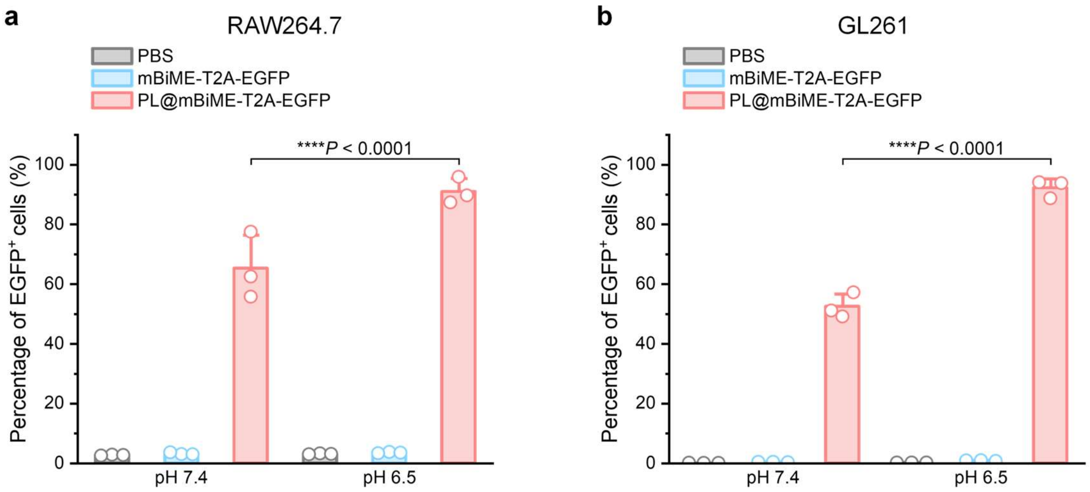

Supplementary Fig. 15. Statistical analysis of EGFP transfection efficiency by flow cytometry. a, b Quantification of EGFP-positive RAW264.7 (Fig. 2g) and GL261 cells (Supplementary Fig. 14.) by flow cytometry 24 hours after treatment with PBS, mBiME-T2A-EGFP, or PL@mBiME-T2A-EGFP. Data are presented as mean ± SD, n = 3 independent experiments; two-way ANOVA with Fisher's LSD post-hoc test.

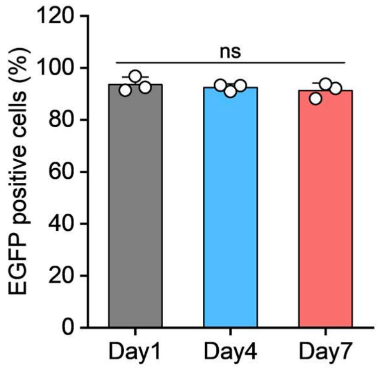

Supplementary Fig. 16. Stability assessment of PL@mBiME stored in 8% sucrose/Tris solution. Transfection efficiency of PL@mBiME-T2A-EGFP in RAW 264.7 cells was evaluated over a seven-day period. Data are presented as mean ± SD, n = 3 independent experiments; one-way ANOVA with Fisher's LSD post-hoc test.

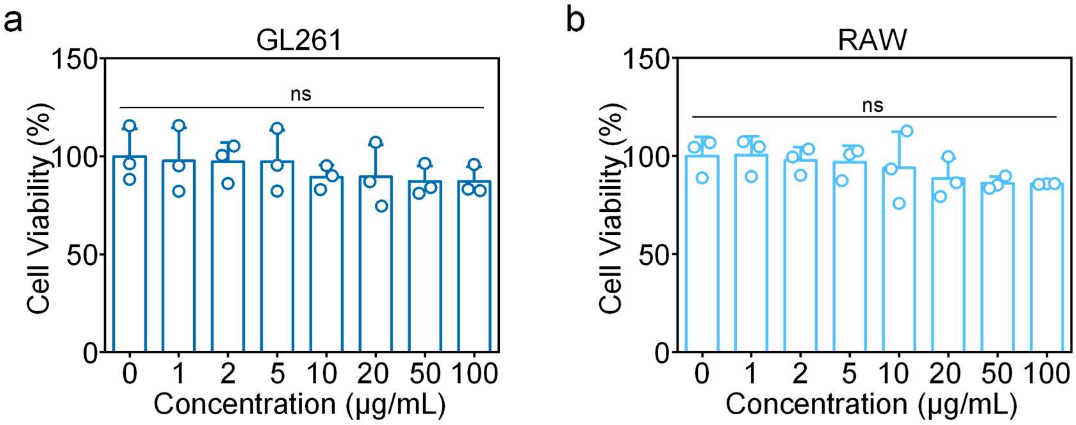

Supplementary Fig. 17. Cytotoxicity of nanoparticles. Cytotoxicity assay of GL261 and RAW264.7 cells by CCK8 after use of different concentrations of PL@mBiME. Data are presented as mean ± SD, n = 3 independent experiments; one-way ANOVA with Fisher's LSD post-hoc test.

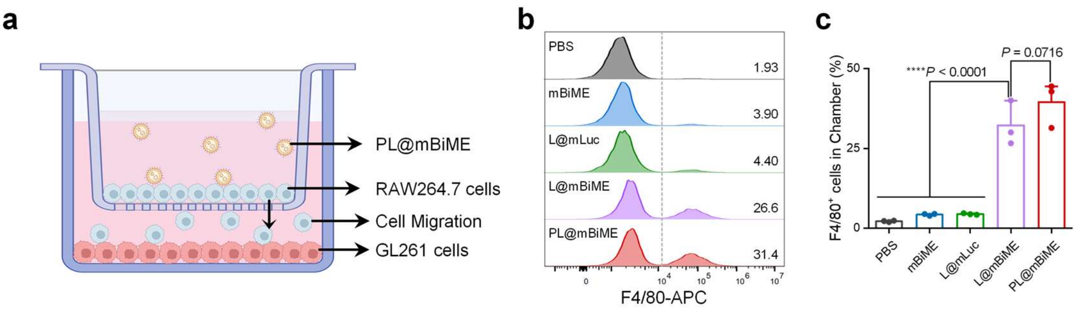

Supplementary Fig. 18. PL@mBiME treatment promote the macrophage migration towards tumor. a-c Schematic diagram of macrophage migration. RAW264.7 cells were plated in the upper layer, and GL261 tumor cells were plated in the lower layer. The migration of RAW264.7 cells was observed after the addition of PL@mBiME. RAW264.7 cells were labeled with F4/80, and flow cytometric histograms and quantitative graphs were obtained, n = 3 independent experiments. Data are presented as mean ± SD, statistical analysis was performed by one-way ANOVA with Fisher's LSD post-hoc test. Created in BioRender. Fei, G. (2026) https://BioRender.com/bq1g0ow.

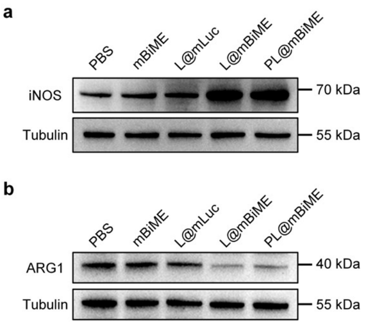

Supplementary Fig. 19. Analysis of M1 and M2 phenotypic markers in BMDMs by western blot. a, b BMDMs were treated with different formulations, and the expression levels of iNOS (a, M1 marker) and Arg-1 (b, M2 marker) were assessed. Uncropped blots in Source Data.

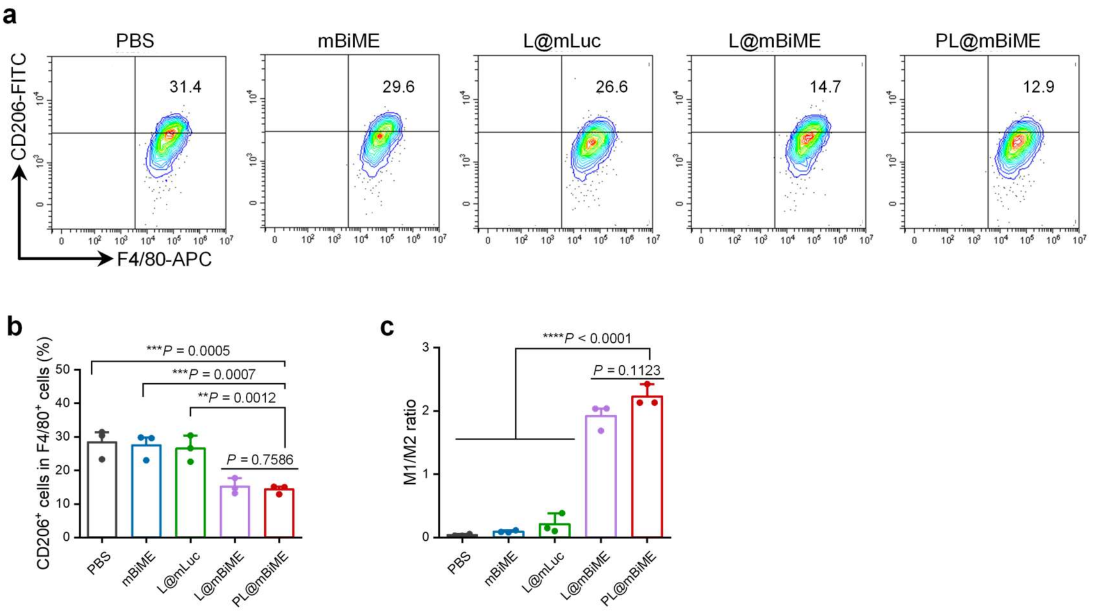

Supplementary Fig. 20. Flow cytometry analysis of macrophage polarization. a Representative flow cytometry dot plots showing the propotion of M2 macrophages ($F4/80^+$ $CD206^+$) after treatment with different formulations. **b** Quantitative analysis of the M2 macrophage polarization ratio. **c** M1/M2 ratio of BMDMs treated with various formulations, n = 3 independent experiments. Data are presented as mean ± SD, statistical analysis was performed by one-way ANOVA with Fisher's LSD post-hoc test.

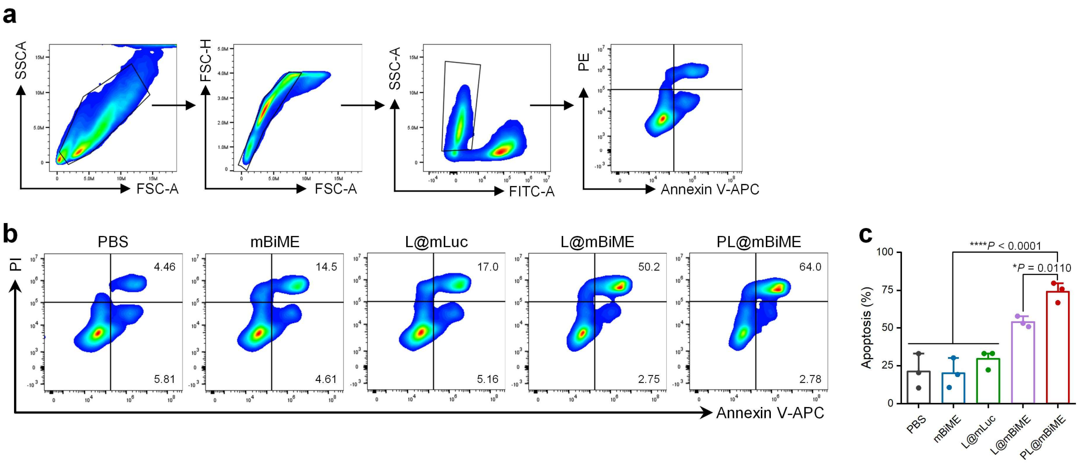

Supplementary Fig. 21. PL@mBiME enhance macrophage triggered tumor cell apoptosis. a Gating diagram for verifying the apoptosis of tumor cells by flow cytometry. Gated on DiO$^-$ Annexin$^+$ cells. (Pre-treat BMDMs with the membrane dye DiO.) **b, c** Cell apoptosis assays were performed 24 hours after treatment with each formulation, and the numbers of apoptotic and dead GL261 cells in each treatment group were determined, n = 3 independent experiments. Data are presented as mean ± SD, statistical analysis was performed by one-way ANOVA with Fisher's LSD post-hoc test.

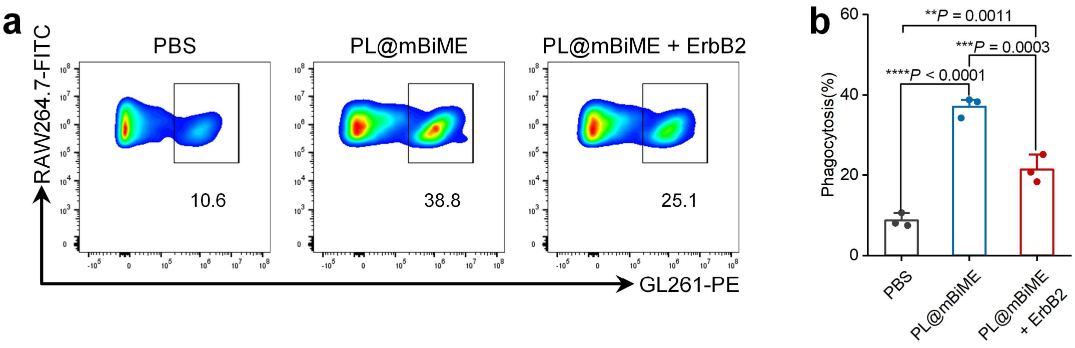

Supplementary Fig. 22. Competitive inhibition by ErbB2 reduces BiME-mediated phagocytosis. RAW264.7 cells and GL261 cells were treated with PBS, PL@mBiME and PL@mBiME + ErbB2 for 24 h, then phagocytosis was analyzed by flow cytometry and quantified, RAW264.7 cells were labeled with CD11b-FITC, and GL261 was labeled with DiI, n = 3 independent experiments. Data are presented as mean ± SD,

statistical analysis was performed by one-way ANOVA with Fisher's LSD post-hoc test.

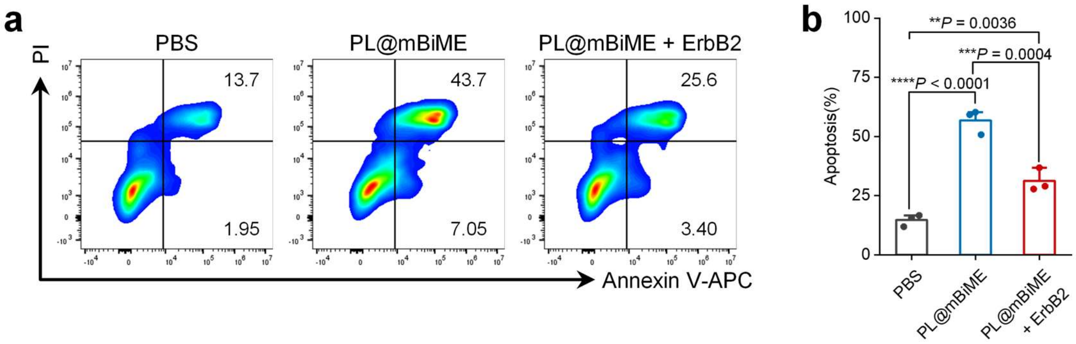

Supplementary Fig. 23. ErbB2 competition diminishes BiME-induced tumor cell apoptosis. RAW264.7 cells and GL261 cells were treated with PBS, PL@mBiME and PL@mBiME + ErbB2 for 24 h, then apoptosis was analyzed by flow cytometry and quantified, n = 3 independent experiments. Data are presented as mean ± SD, statistical analysis was performed by one-way ANOVA with Fisher's LSD post-hoc test.

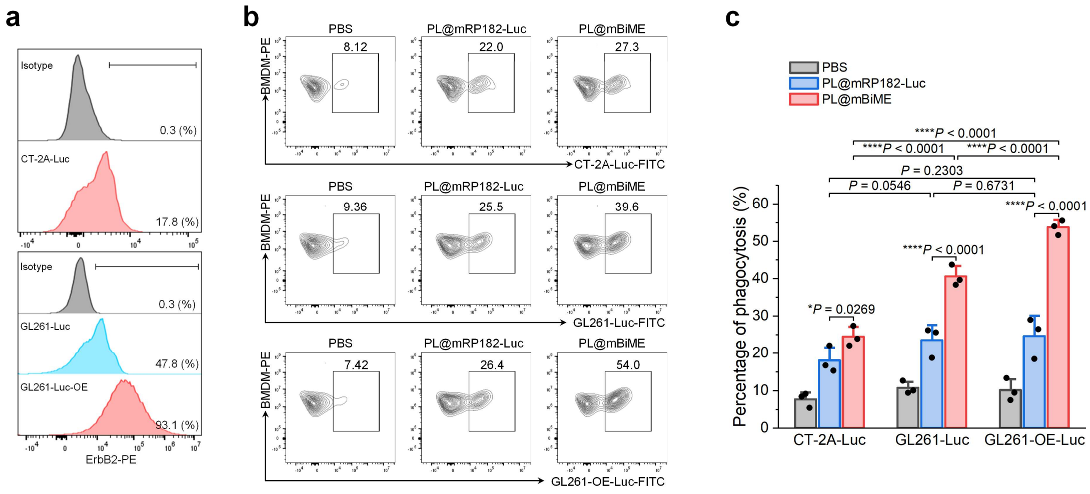

Supplementary Fig. 24. Analysis of ErbB2 expression across different cell lines and flow cytometric assessment of macrophage-mediated tumor phagocytosis. a Representative flow cytometry histograms showing ErbB2 expression in CT-2A, GL261, and GL261-OE cell lines. **b** Flow cytometry plots showing the phagocytic activity of BMDMs after co-incubation with CT2A, GL261, or GL261-OE cells under

different treatment conditions (PBS, PL@mRP182-Luc, or PL@mBiME). **c** Quantification of phagocytic activity based on the flow cytometry plots shown in (b), n = 3 independent experiments. Data are presented as mean ± SD. Statistical analysis was performed by two-way ANOVA with Fisher's LSD post-hoc test.

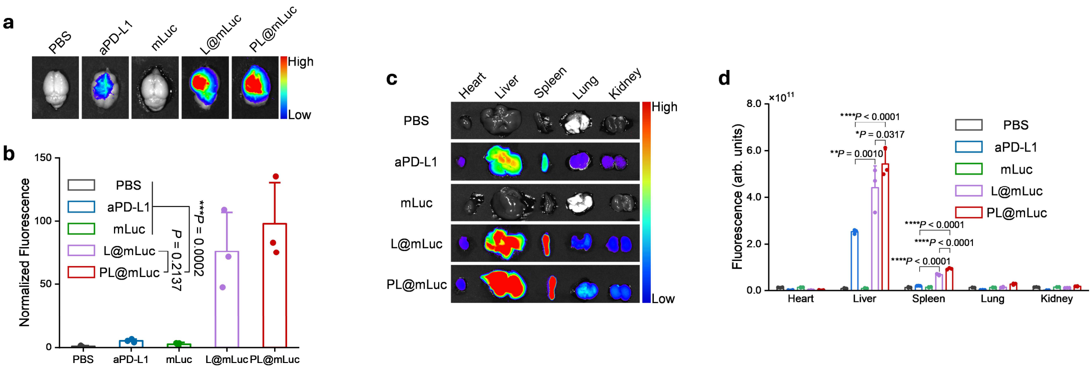

Supplementary Fig. 25. Biodistribution of fluorescently labeled formulations in major organs. a Representative *ex vivo* fluorescence images of brains collected from mice 12 hours after systemic administration. **b** Quantitative analysis of the fluorescence intensity in brain tissues from panel (a), n = 3 independent experiments. **c** Representative *ex vivo* fluorescence images of the heart, liver, spleen, lungs, and kidneys collected from mice 12 hours after systemic administration. **d** Quantitative analysis of the fluorecence in the organs shown in panel (c), n = 3 independent experiments. Data are presented as mean ± SD, statistical analysis was performed by one-way ANOVA with Fisher's LSD post-hoc test.

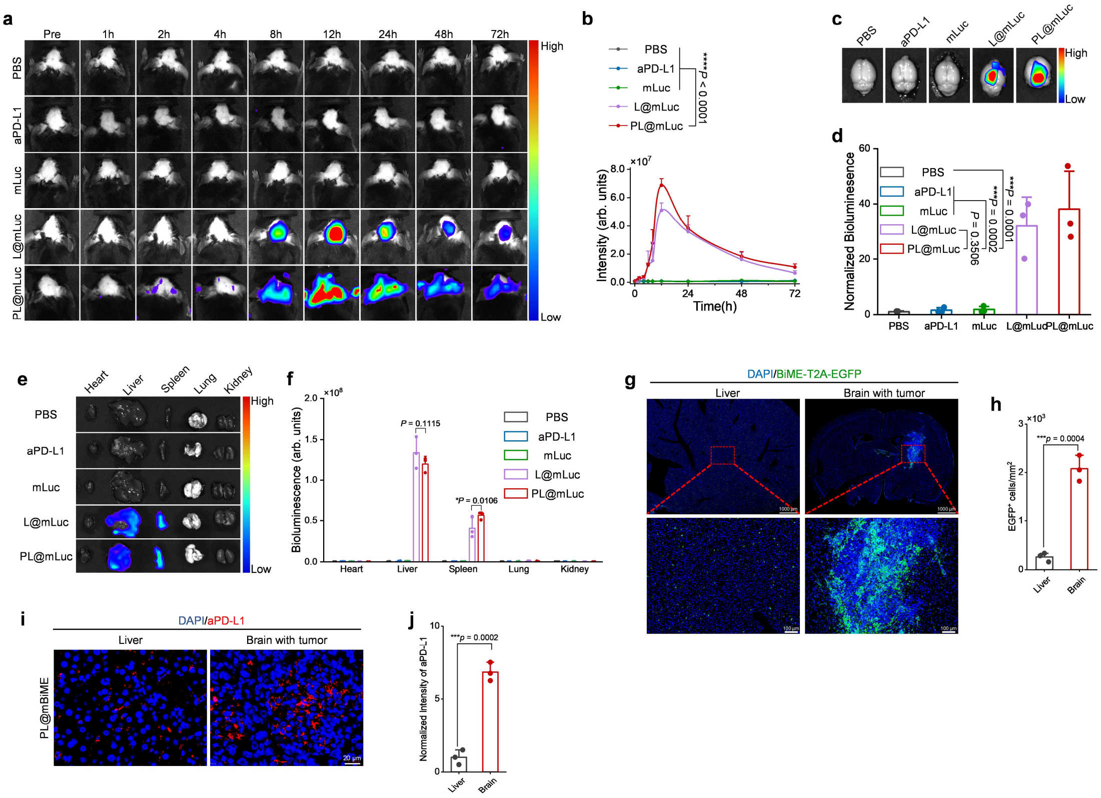

Supplementary Fig. 26. The expression biodistribution of PL@BiME. **a, b** *In vivo* bioluminescence imaging (a) and corresponding intensity quantification (b) at the tumor site over time after *i.v.* injection of the indicated formulations, n = 3 independent experiments. **c, d** *Ex vivo* bioluminescence imaging (c) and quantification (d) of the tumor-bearing brains harvested 12 hours after *i.v.* injection of the indicated formulations, n = 3 independent experiments. Statistical analysis was performed by one-way ANOVA with Fisher's LSD post-hoc test. **e, f** Bioluminescence imaging and quantification of heart, liver, spleen, lung and kidney 12 hours after *i.v.* injection of GL261 tumor-bearing mice with indicated formulations, n = 3 independent experiments. Statistical analysis was performed by one-way ANOVA with Fisher's LSD post-hoc test. **g, h** Representative fluorescence images showing the expression of mBiME-T2A-EGFP (green) in liver tissues and tumor-bearing brain tissues. Nuclei were counterstained with DAPI (blue), n = 3 independent experiments. Scale bar = 1000 μm, 100μm. Data are presented as mean ± SD. Statistical analysis was performed by unpaired two-tailed t-test. **i** Representative fluorescence images showing the release of aPD-L1 (red) in liver tissues and tumor-bearing brain tissues. Nuclei were

counterstained with DAPI (blue). Scale bar = 20 μm. **j** Quantitative analysis of aPD-L1 fluorescence intensity in liver and tumor-bearing brain tissues, n = 3 independent experiments. Data are presented as mean ± SD. Statistical analysis was performed by unpaired two-tailed t-test.

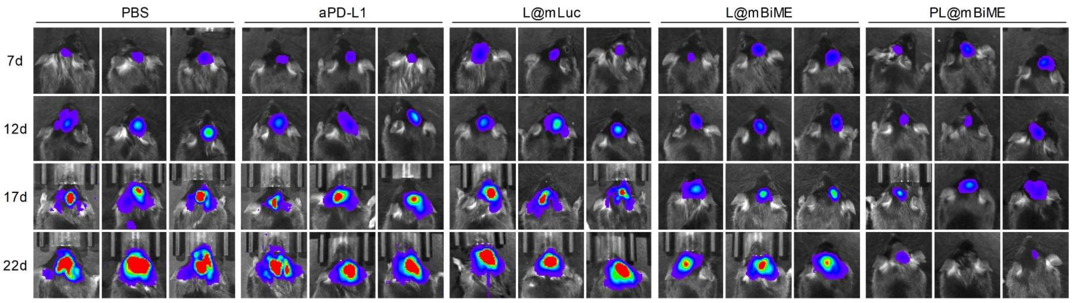

Supplementary Fig. 27. *In vivo* treatment efficacy of PL@mBiME with GL261-luc tumor-baring mice. Antitumor efficacy of various treatments was assessed by an *in vivo* imaging system on day 7, 12, 17, and 22 of constructing the GL261-Luciferase in situ mouse model of GBM, n = 5 independent experiments.

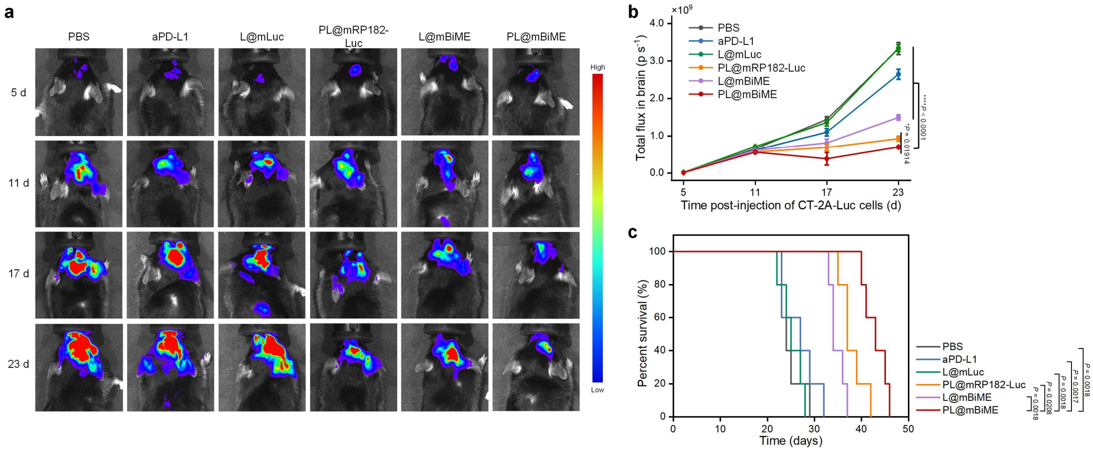

Supplementary Fig. 28. *In vivo* treatment efficacy of PL@mBiME with CT-2A-Luc tumor-baring mice. a Representative IVIS images and quantified signal intensities of CT-2A-Luc tumor-bearing mice treated with PBS, aPD-L1, L@mLuc, PL@mRP182-Luc, L@mBiME, and PL@mBiME, n = 3 independent experiments. **c** Survival curves of CT-2A-Luc tumor-bearing mice following treatment with different formulations, n = 5 independent experiments. Data are presented as mean ± SD,

statistical analysis was performed by one-way ANOVA with Fisher's LSD post-hoc test for b. Survival analysis (c) was compared using the log-rank test.

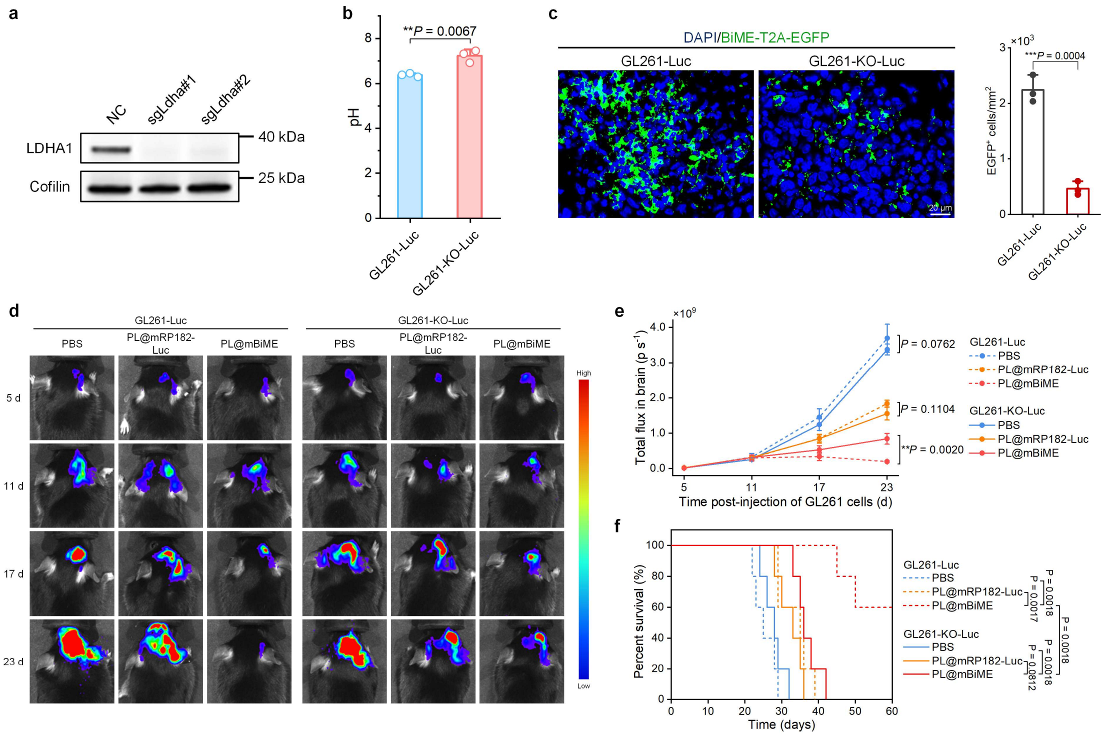

Supplementary Fig. 29. *In vivo* treatment efficacy of PL@mBiME with GL-261-Luc and GL261-KO-Luc tumor-baring mice. a Western Blot verification of LDHA Knockout efficiency in GL261 cells (GL261- KO-Luc). **b** Measurement of intratumoral acidity in GL261-Luc and GL261-KO-Luc brain tissues, n = 3 independent experiments. **c** Representative images and quantification of BiME-T2A-EGFP expression in GL261-Luc and GL261-KO-Luc brain tissues, n = 3 independent experiments. **d, e** Representative IVIS images and quantified signal intensities of GL261-Luc and GL261-KO-Luc tumor-bearing mice treated with PBS, PL@mRP182-Luc and PL@mBiME, n = 3 independent experiments. **f** Survival curves of GL261-Luc and GL261-OE-Luc tumor-bearing mice following treatment with different formulations, n = 5 independent experiments. Data are presented as mean ± SD. Statistical analysis was performed by unpaired two-tailed Student's t-test for b and c. Statistical analysis was performed by two-way ANOVA with Fisher's LSD post-hoc test for e. Survival analysis (f) was compared using the log-rank test.

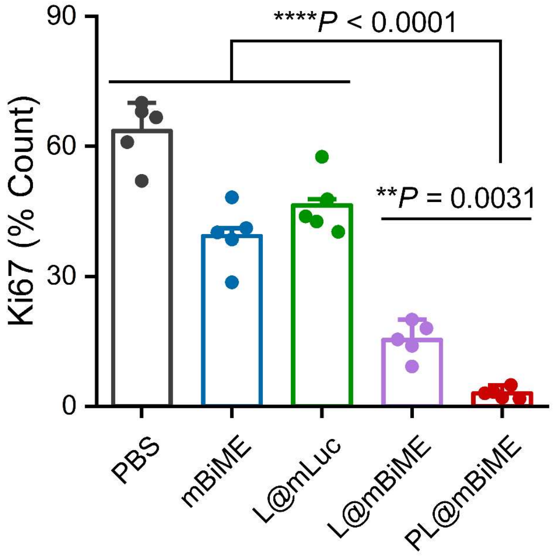

Supplementary Fig. 30. Detection of Ki67 following various treatments. Immunohistochemical Ki-67statistics of tumor sections with different treatments at 22 days after tumor cell inoculation, n = 5 independent experiments. Data are presented as mean ± SD, statistical analysis was performed by one-way ANOVA with Fisher's LSD post-hoc test.

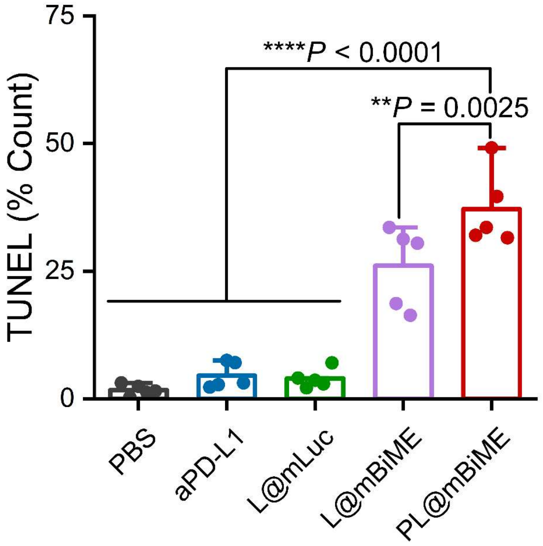

Supplementary Fig. 31. Detection of TUNEL following various treatments. TUNEL statistics of tumor sections with different treatments at 22 days after tumor cell inoculation, n = 5 independent experiments. Data are presented as mean ± SD, statistical analysis was performed by one-way ANOVA with Fisher's LSD post-hoc test.

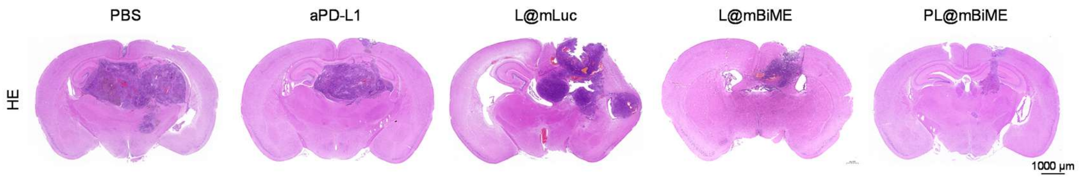

Supplementary Fig. 32. H&E images of the tumor tissues after different treatments. Representative H&E staining images of GL261 tumor-bearing mice from different treatment groups 30 days after tumor cell inoculation, n = 3 independent experiments. Scale bar = 1000 μm.

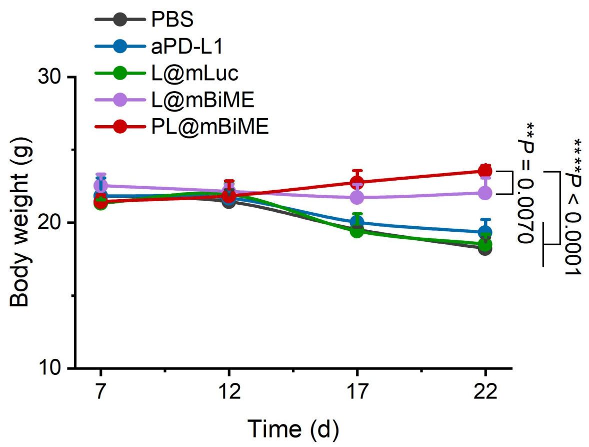

Supplementary Fig. 33. Body weight monitoring following treatment with different formulations. Body weight changes in GL26l tumor-bearing mice over 22 days treated with different formulas, n = 5 independent experiments. Data are presented as mean ± SD, statistical analysis was performed by one-way ANOVA with Fisher's LSD post-hoc test.

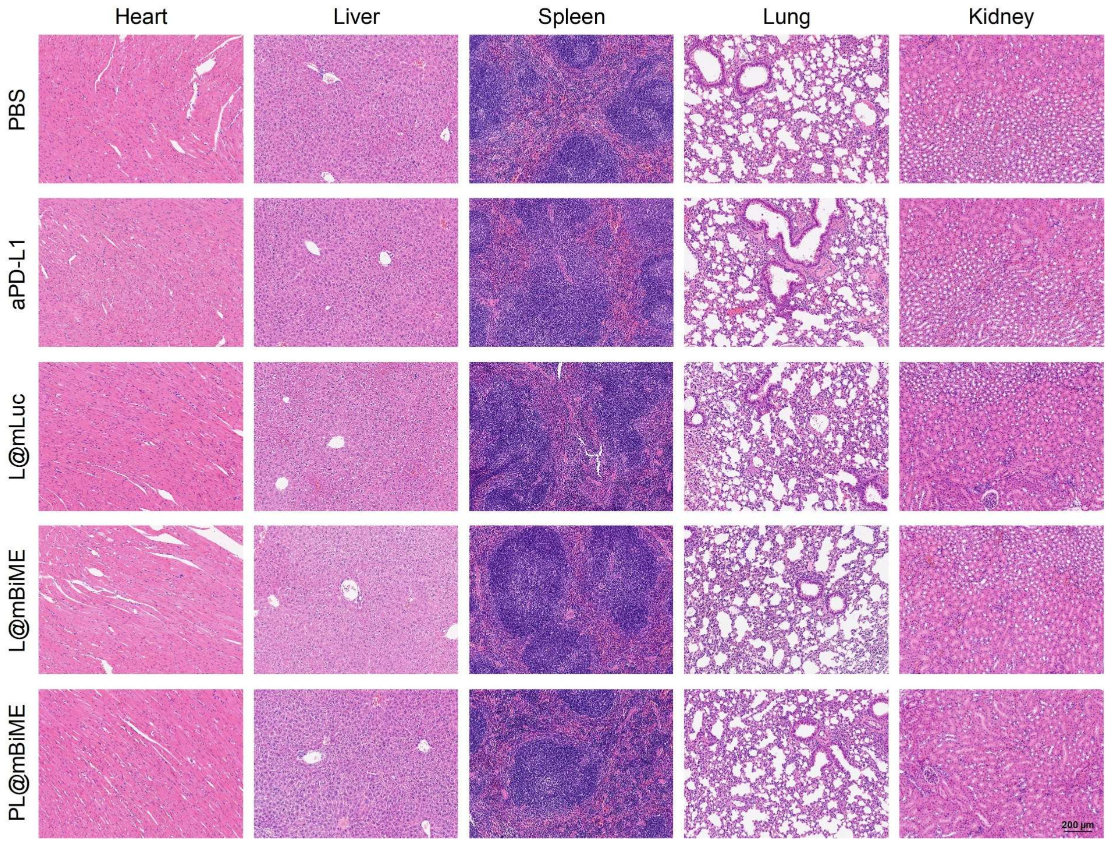

Supplementary Fig. 34. HE staining of heart, liver, spleen, lung, and kidney tissue sections was performed after various treatments. Seven days after three treatments with various formulations, mice were euthanized and vital organs (heart, liver, spleen, lung, and kidney) were excised, followed by HE staining to detect potential damage, n = 3 independent experiments. Scale bar = 200 μm.

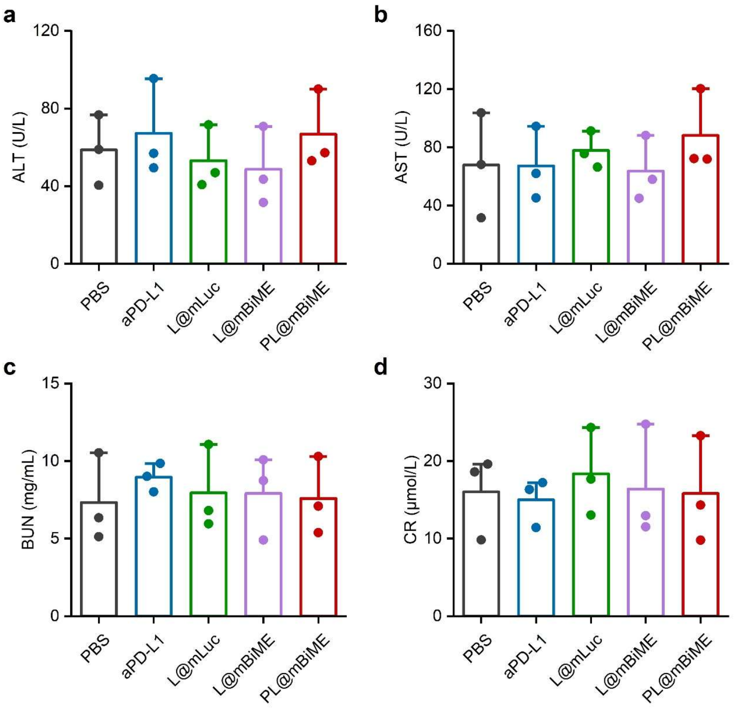

Supplementary Fig. 35. Hematological indicators of the biosafety of various treatments in healthy mice. Hematological indexes of healthy mice seven days after three treatments with various formulations. **a** AST levels after treatments. **b** ALT levels aftertreatments. **c** BUN levels after treatments. **d** Creatinine levels after treatments. Data are presented as mean ± SD, n = 3 independent experiments.

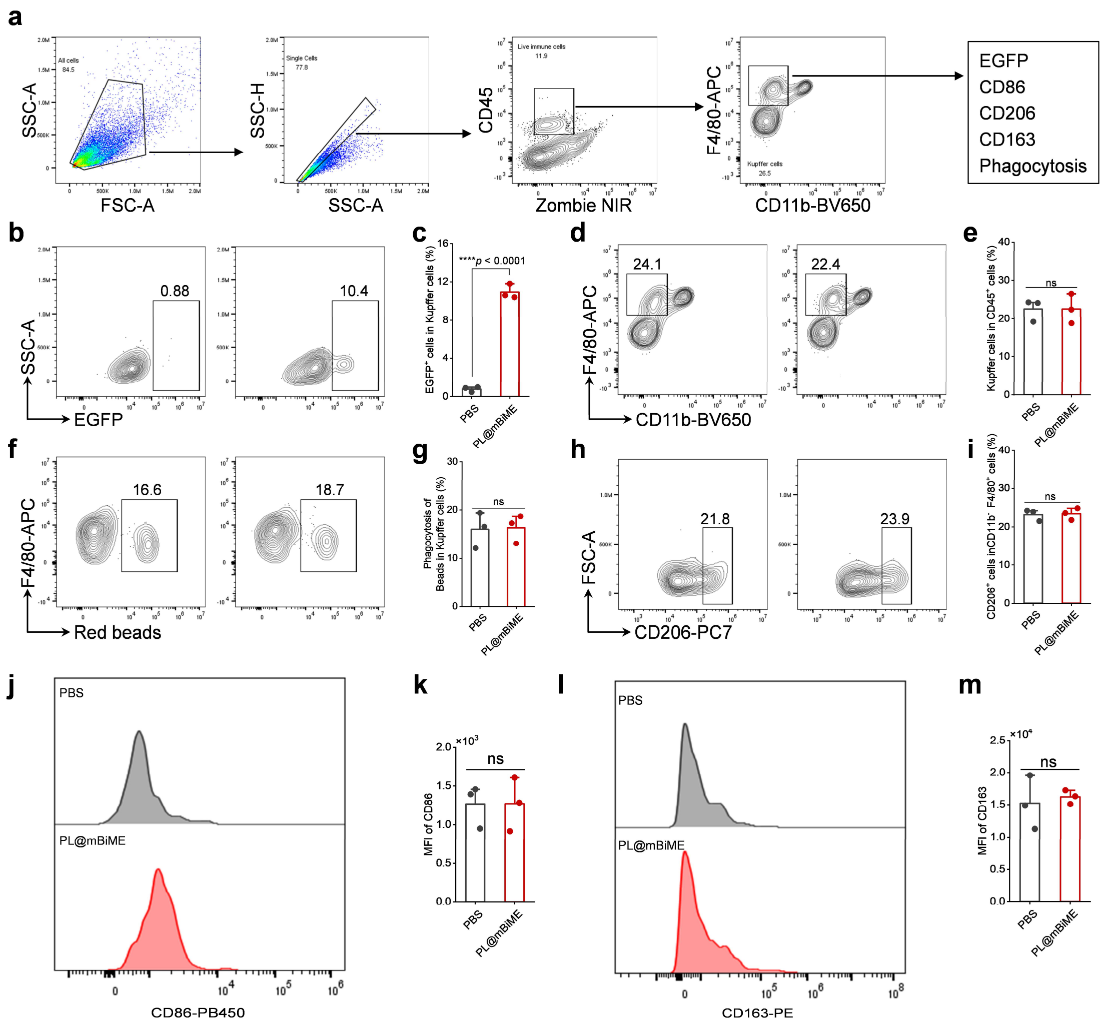

Supplementary Fig. 36. Phenotypic and functional modulation of Kupffer cells by PL@mBiME *in vivo*. **a** Gating strategy for liver Kupffer cell analysis. **b** Dot plots of EGFP expression in Kupffer cells post PL@mBiME-T2A-EGFP treatment. **c** Quantification of EGFP$^+$ Kupffer cell percentage (from b). **d** Dot plots showing Kupffer cell proportion among liver leukocytes. **e** Quantification of Kupffer cell percentage (from d). **f** Histogram of phagocytosis (latex beads uptake) by Kupffer cells. **g** Quantification of phagocytic activity (from f). **h** Histogram of CD206 expression on Kupffer cells. **i** Quantification of CD206$^+$ cell percentage (from h). **j** Histogram of CD86 expression on Kupffer cells. **k** Quantification of CD86 mean fluorescence intensity (MFI, from j). **l** Histogram of CD163 expression on Kupffer cells. **m** Quantification of CD163 median fluorescence intensity (MedFI, from l). Data are presented as mean ± SD, n = 3 independent experiments; unpaired two-tailed t-test.

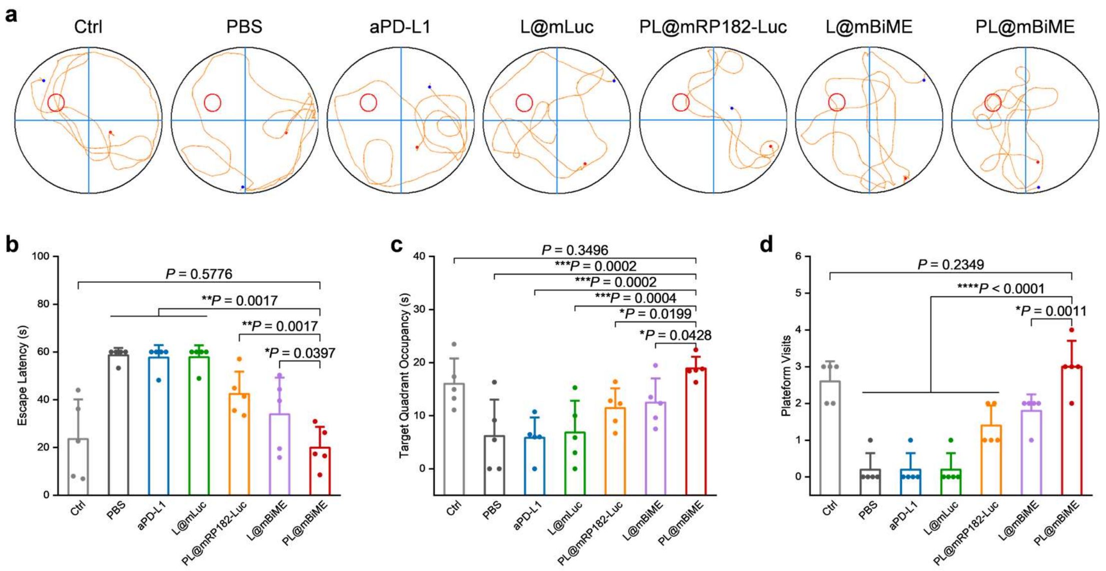

Supplementary Fig. 37. Assessment of murine cognitive function via behavioral testing. a Representative Morris water maze trajectories treated with various formulations. **b-d** Statistical analysis of escape latency, target quadrant occupancy, and platform visits in various formulation groups, n = 5 independent experiments. Data are presented as mean ± SD, statistical analysis was performed by one-way ANOVA with Fisher's LSD post-hoc test.

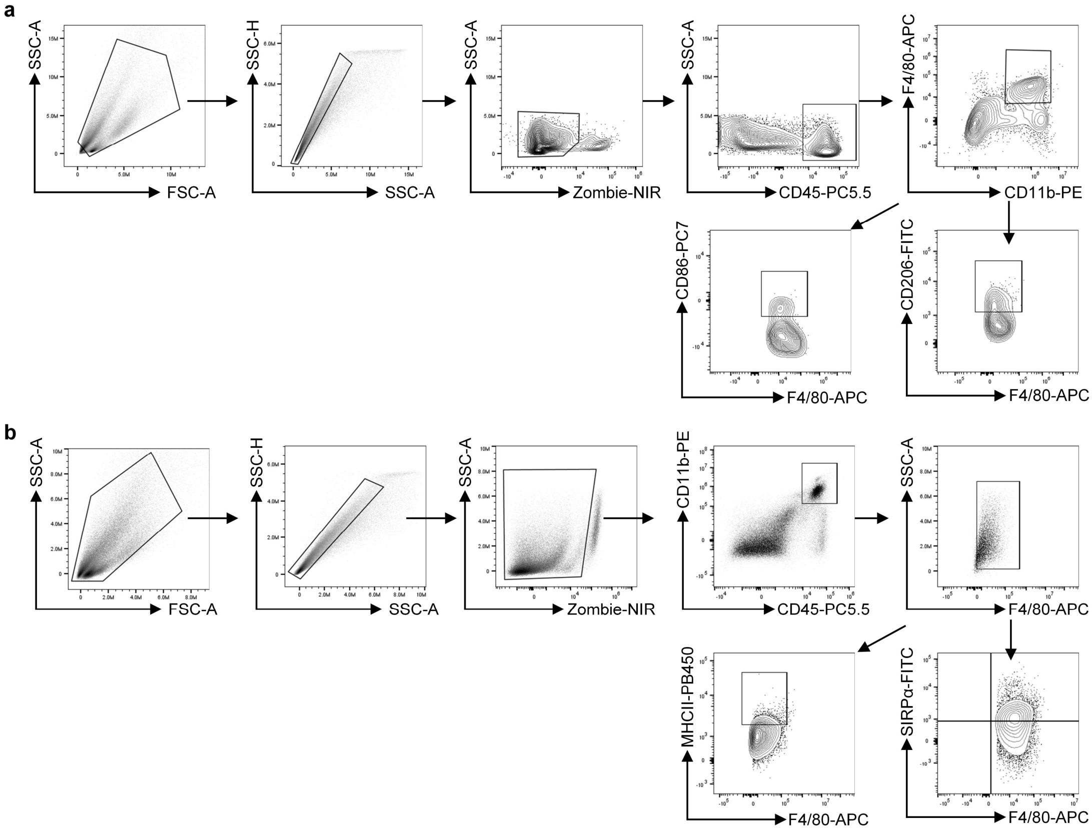

Supplementary Fig. 38. Flow cytometry gating strategies of Macrophages. a Gating strategy for identifying M1-type (CD86⁺) and M2-type (CD206⁺) macrophages within tumor tissues, gated on Live⁺ CD45⁺ CD11b⁺ F4/80⁺ cells. **b** Gating strategy for identifying MHCII⁺ and SIRPα⁺ macrophages within tumor tissues, gated on Live⁺ CD45⁺ CD11b⁺ F4/80⁺ cells.

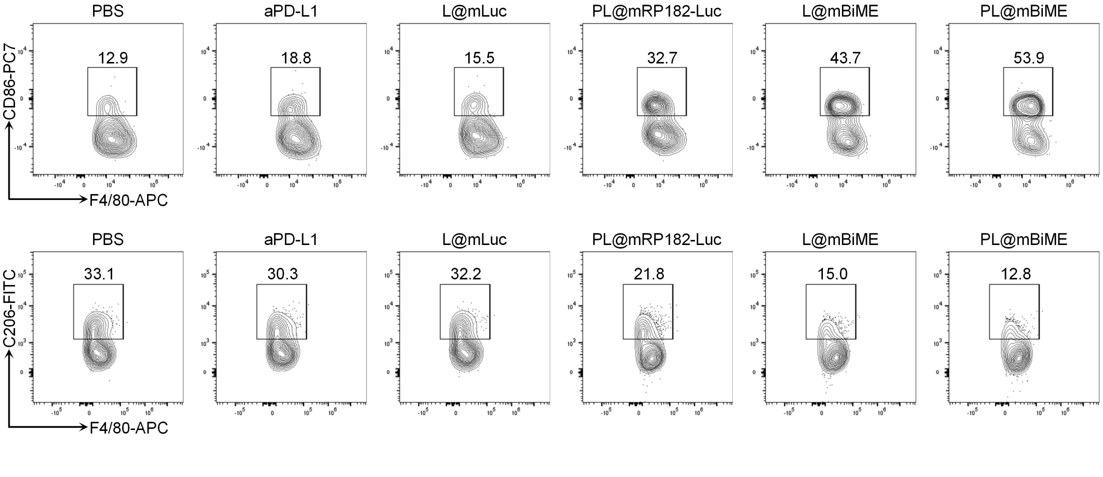

Supplementary Fig. 39. PL@mBiME alteres macrophage polarization in tumor tissues, as demonstrated by flow cytometry analysis. Representative flow cytometry contour plots of tumor-infiltrating TAM polarization (M1 marker CD86, M2 marker CD206) cells three days after three treatments with PBS, aPD-L1, L@mLuc, PL@mRP182-Luc, L@mBiME, and PL@mBiME, n = 3 independent experiments.

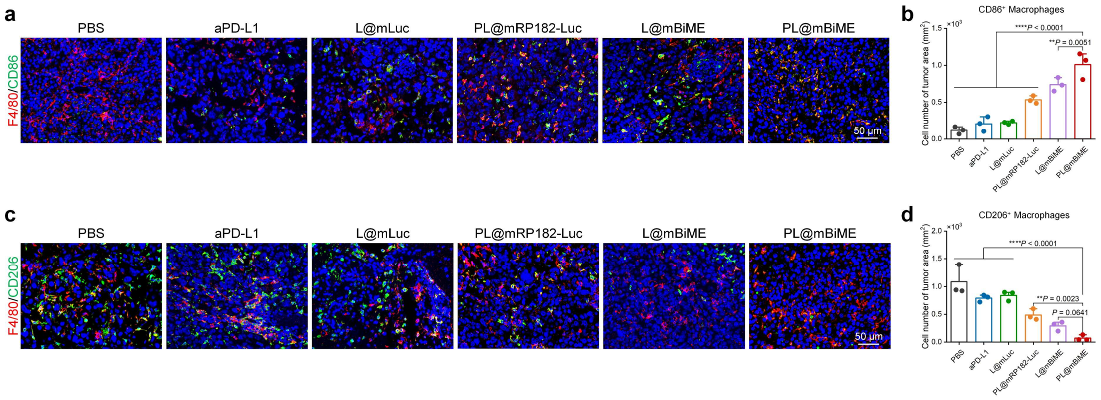

Supplementary Fig. 40. PL@mBiME alteres macrophage polarization in tumor tissues, as demonstrated by immunofluorescence analysis. a Representative immunofluorescence images of macrophages in tumor tissue sections three days after injections of different treatments. Blue: DAPl; red: F4/80- Cy3; green: CD86-FITC. Scale bar = 50 μm. **b** Statistical charts of $CD86^+$ Macrophage in immunofluorescence sections, n = 3 independent experiments. **c** Representative immunofluorescence images of macrophages in tumor tissue sections three days after injections of different treatments. Blue:DAPl; red: Cy3-F4/80; green: FITC-CD206. Scale bar = 50 μm. **d** Statistical charts of $CD206^+$ Macrophage in immunofluorescence sections, n = 3 independent experiments. Data are presented as mean ± SD, statistical analysis was performed by one-way ANOVA with Fisher's LSD post-hoc test.

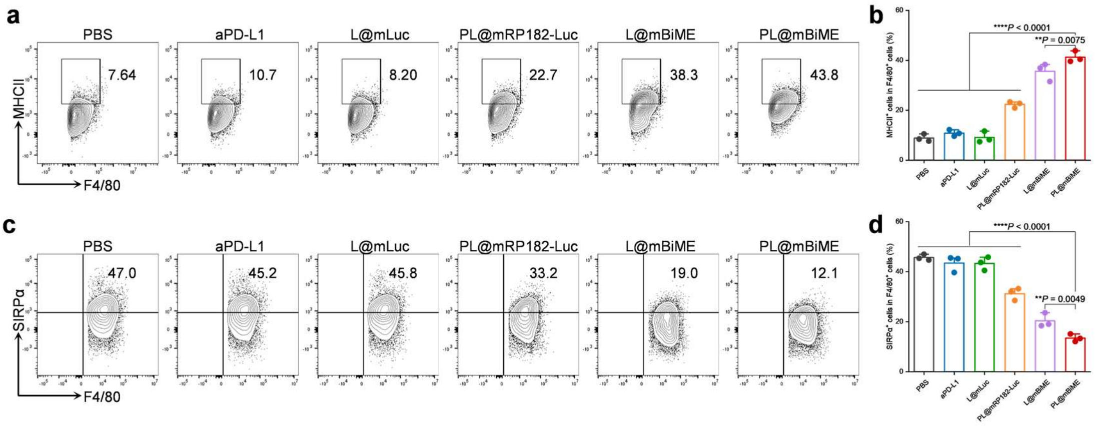

Supplementary Fig. 41. Effect of PL@mBiME on MHCII and SIRPα of tumor-associated macrophages (TAMs) *in vivo*. a, b Representative flow cytometric images and quantification of MHCII$^+$ antigen presentation in TAMs following treatment with different formulations, n = 3 independent experiments. **c, d** Representative flow cytometric images of SIRPα$^+$ in TAMs following treatment with different formulations, n = 3 independent experiments. Data are presented as mean ± SD, statistical analysis was performed by one-way ANOVA with Fisher's LSD post-hoc test.

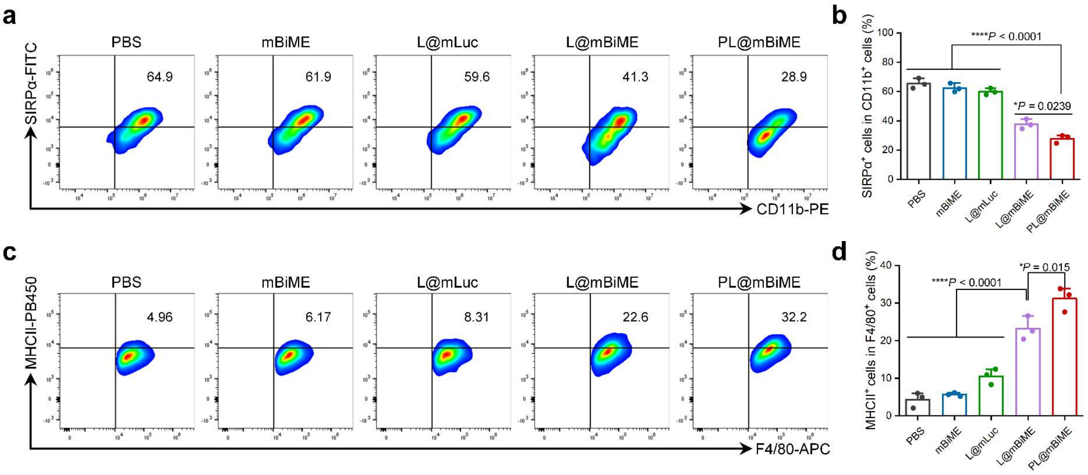

Supplementary Fig. 42. Inhibitory effect of PL@mBiME on SIRPα of tumor-associated macrophages (TAMs) and antigen presentation effect *in vitro*. a, b Representative flow cytometric images and quantification of SIRPα$^+$ following treatment with different formulations, n = 3 independent experiments. **c, d** Representative flow cytometric images of MHCII$^+$ antigen presentation following

treatment with different formulations, n = 3 independent experiments. Data are presented as mean ± SD, statistical analysis was performed by one-way ANOVA with Fisher's LSD post-hoc test.

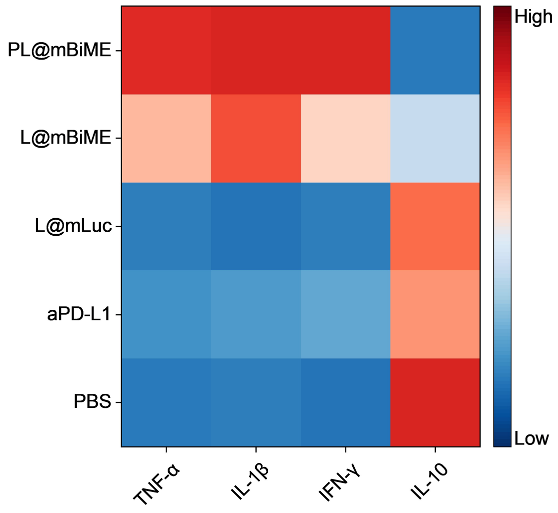

Supplementary Fig. 43. Determination of inflammatory cytokines following treatment with different formulations. Pro-inflammatory factor (TNF-α, IL-1β, IFN-γ) and anti-inflammatory factor (IL-10) levels and in tumor tissues treated with PBS, aPD-L1, L@mLuc, L@mBiME and PL@mBiME for three times, respectively, n = 3 independent experiments.

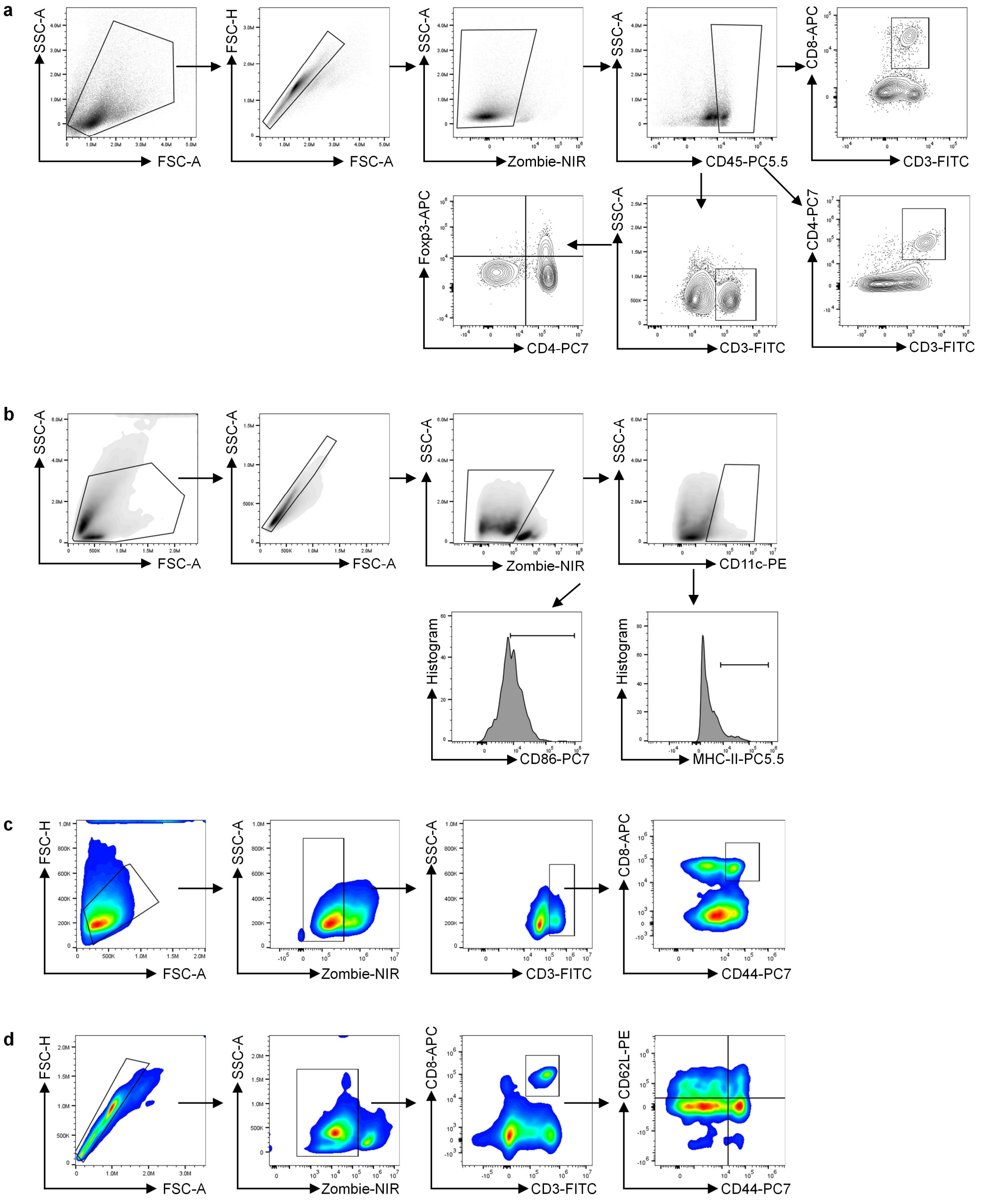

Supplementary Fig. 44. Graphical account for flow cytometry gating strategies of CD8$^+$ T cells, CD4$^+$ T cells, Tregs, DCs and T memory cells. a Gating strategy to sort CD8$^+$ T cells and CD4$^+$ T cells in tumor tissues gating on Live$^+$ CD45$^+$ CD3$^+$ cells and Treg cells in tumor tissues gating on Live$^+$ CD45$^+$ CD3$^+$ CD4$^+$ Foxp3$^+$ cells. **b** Gating strategy to access the level of mDC (MHCII$^+$ and CD86$^+$) in tumor tissues gating on Live$^+$ CD11c$^+$ cells. **c** Gating strategy to sort CD44^{hi} CD8$^+$ memory T cells in tumor

tissues gating on Live$^+$ CD3$^+$ cells. **d** Gating strategy to sort CD44^{hi} CD62^{Lo} effector memory T cells in spleen tissues.

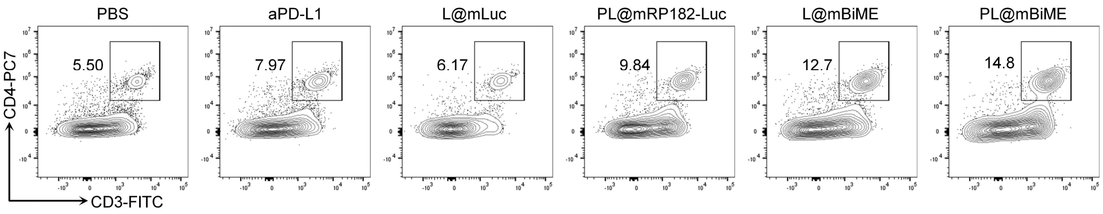

Supplementary Fig. 45. PL@mBiME treatment increases the proportion of tumor-infiltrating CD4$^+$ T cells. Representative flow cytometry plots of tumor-infiltrating CD4$^+$ T cells three days after three injections of treatments with PBS, aPD-L1, L@mLuc, PL@mRP182-Luc, L@mBiME and PL@mBiME, respectively, n = 3 independent experiments.

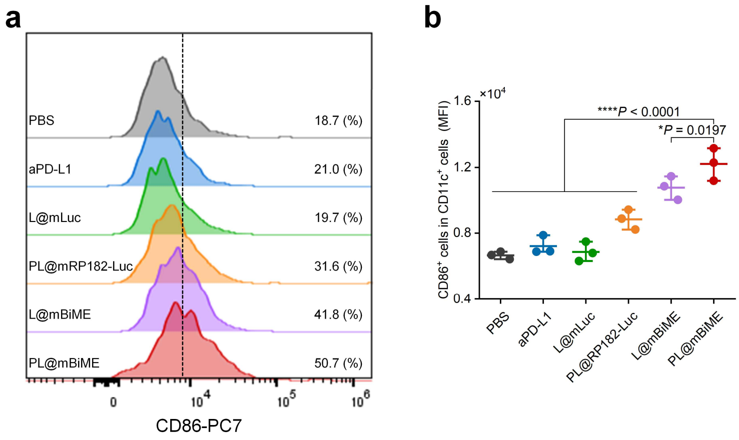

Supplementary Fig. 46. PL@mBiME treatment promotes DC (CD86) maturation. a, b Flow cytometric histograms and statistical quantitative graphs of the mean fluorescence intensity (MFI) of tumor-infiltrating dendritic cells (DCs, CD86) three days after being treated with PBS, aPD-L1, L@mLuc, PL@mRP182-Luc, L@mBiME and PL@mBiME respectively, n = 3 independent experiments. Data are presented as mean ± SD, statistical analysis was performed by one-way ANOVA with Fisher's LSD post-hoc test.

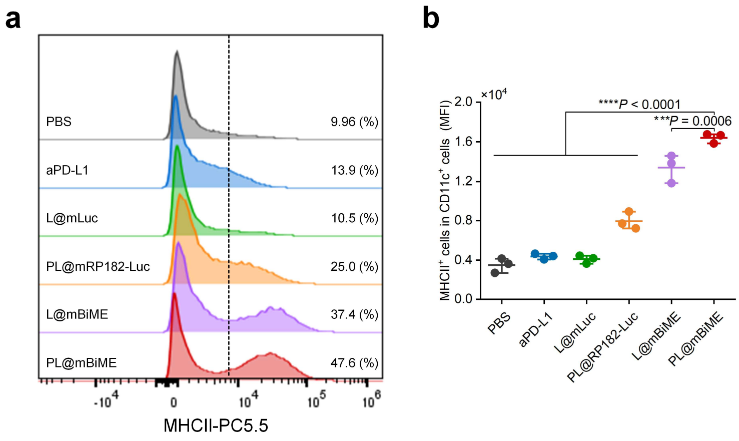

Supplementary Fig. 47. PL@mBiME treatment promotes DC (MHCII) maturation. a, b Flow cytometric histograms and statistical quantitative graphs of the mean fluorescence intensity (MFI) of tumor-infiltrating dendritic cells (DCs, major histocompatibility complex class II molecules (MHCII)) three days after being treated with PBS, aPD-L1, L@mLuc, PL@mRP182-Luc, L@mBiME and PL@mBiME respectively, n = 3 independent experiments. Data are presented as mean ± SD, statistical analysis was performed by one-way ANOVA with Fisher's LSD post-hoc test.

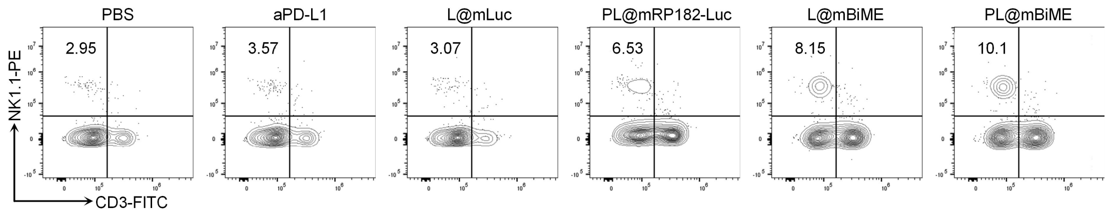

Supplementary Fig. 48. PL@mBiME treatment increases the proportion of tumor-infiltrating natural killer cells (NK cells). Representative flow cytometry plots of tumor-infiltrating NK cells three days after being treated with PBS, aPD-L1, L@mLuc, PL@mRP182-Luc, L@mBiME and PL@mBiME, respectively, n = 3 independent experiments.

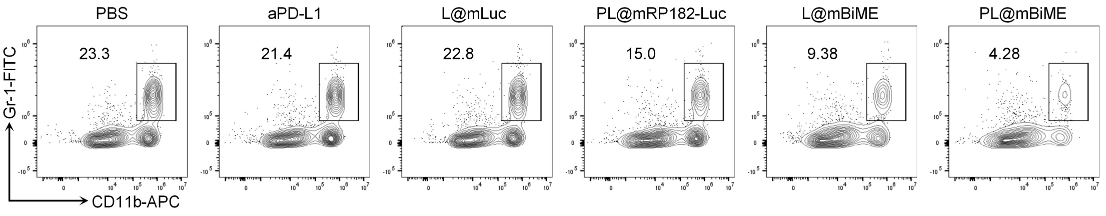

Supplementary Fig. 49. PL@mBiME treatment decreases the proportion of tumor-infiltrating myeloid-derived suppressor cells (MDSCs). Representative flow cytometry plots of tumor-infiltrating MDSCs three days after being treated with PBS, aPD-L1, L@mLuc, PL@mRP182-Luc, L@mBiME and PL@mBiME, respectively, n = 3 independent experiments.

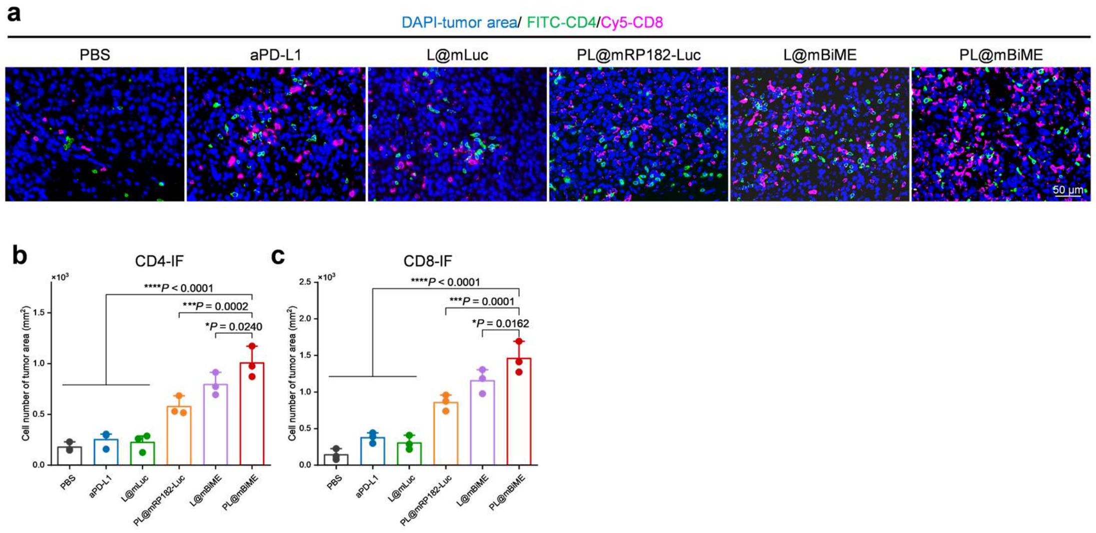

Supplementary Fig. 50. Immunofluorescence staining of lymphocytes in tumor sample from different treatment group. a-c Immunofluorescence (IF) images and quantification of tumor-infiltrating T cells three days after three injections of PBS, aPD-L1, L@mLuc, PL@mRP182-Luc, L@mBiME, and PL@mBiME treatments (blue: DAPI; green: Spgreen-CD4; purple: Cy5-CD8; n = 3 independent experiments, scale bar = 50 μm). Data are presented as mean ± SD, statistical analysis was performed by one-way ANOVA with Fisher's LSD post-hoc test.

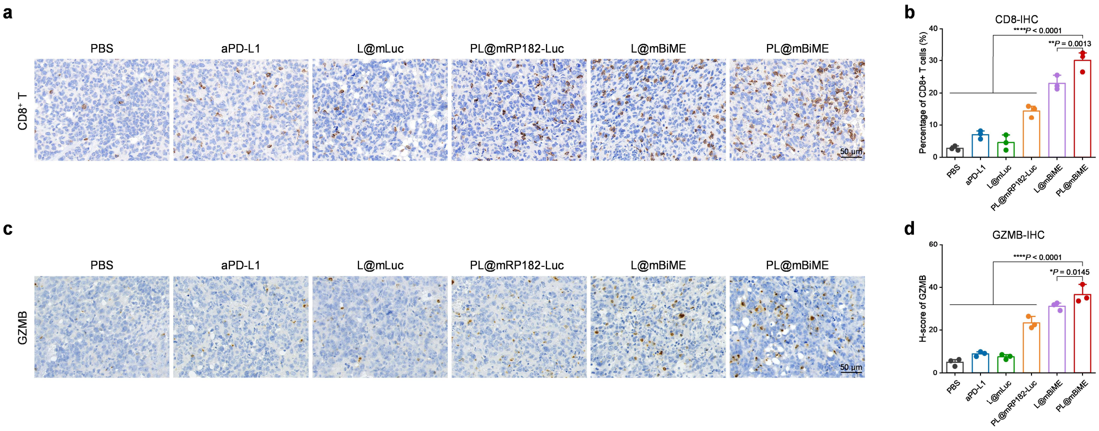

Supplementary Fig. 51. Quantitative immunohistochemical profiling of cytotoxic immune response in treated tumors. a Representative immunohistochemical (IHC) staining for $CD8^+$ cytotoxic T lymphocytes within tumor sections from mice receiving the indicated treatments. **b** Quantification of $CD8^+$ T cell positivity rate (percentage of $CD8^+$ cells among total nucleated cells) from images in panel (a). **c** Representative IHC staining for the cytolytic effector protein Granzyme B (GZMB). **d** Quantification of GZMB expression level using the H-Score (Histochemical Score) method, which integrates both the intensity and proportion of positive staining, from images in panel (c). Scale bar = 50 μm Quantitative data (b, d) are presented as mean ± SD, n = 3 independent experiments. Data are presented as mean ± SD, statistical analysis was performed by one-way ANOVA with Fisher's LSD post-hoc test.

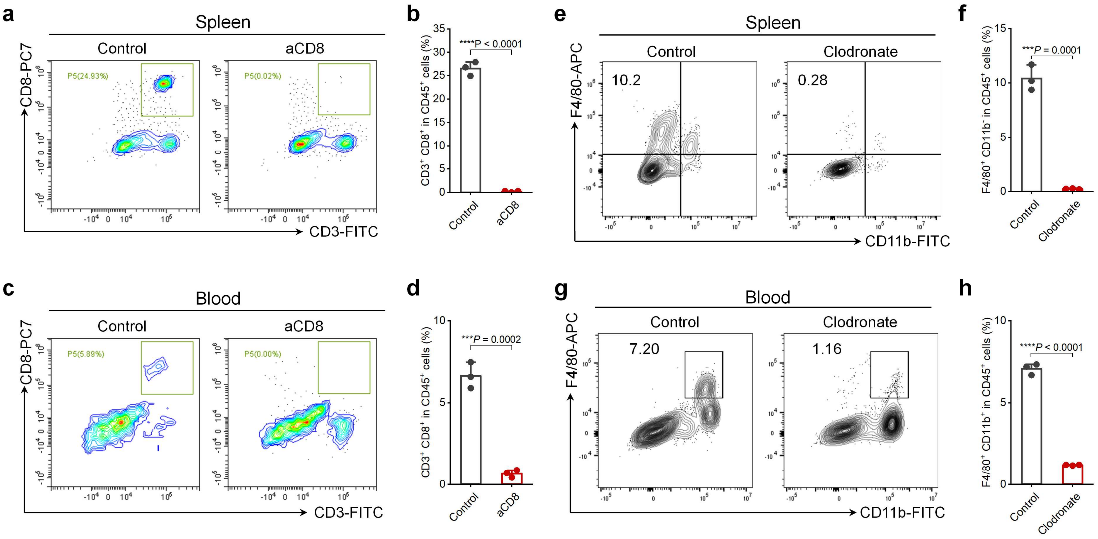

Supplementary Fig. 52. ***In vivo*** **depletion of specific immune subsets and its impact on PL@mBiME efficacy. a-d** Validation of $CD8^+$ T cell depletion. Representative flow cytometry dot plots (a, c) and corresponding quantification (b, d) show the frequency of $CD8^+$ T cells among $CD45^+$ leukocytes in the spleen (a, b) and peripheral blood (c, d) of mice treated with anti-CD8α depleting antibody. **e-h** Validation of macrophage depletion. Representative plots (e, g) and quantification (f, h) show the frequency of $F4/80^+$ macrophages among $CD45^+$ leukocytes in the spleen (e, f) and blood (g, h) of mice treated with clodronate liposomes. Data in b, d, f, h are presented as mean ± SD, n = 3 independent experiments. Statistical significance was determined by unpaired t-test (b, d, f, h).

Supplementary Tables

Supplementary Table 1. Application of reporter mRNA and its variants.

Reporter mRNA and variations	Figure	Application
mBiME	Fig. 4 and Fig. 6-9	Treatment
mBiME-T2A-EGFP	Fig. 3e-g, Supplementary Fig. 7, Supplementary Fig. 13-16, Supplementary Fig. 26 and Supplementary Fig. 29.	Transfection efficiency and tissue tracing
mRP182-Luc	Fig. 4e-f, Fig. 7h-l and Supplementary Fig. 24 and Supplementary Fig. 28-29	The correlation between therapeutic efficacy and ErbB2 expression level
mLuc	Fig. 5f-j and Supplementary Fig. 26.	In vivo delivery
FITC-mBiME	Fig. 3a	Cellular uptake
Cy5-mBiME	Fig. 3b-d, Fig. 5a-c and Supplementary Fig. 9.	GSH-responsive release, lysosomal escape and in vitro blood-brain barrier penetration

Supplementary Table 2. Number of Angiopep-2 and aPD-L1 per nanoparticle.

Nanoparticle	Particle number per mL	Angiopep-2	aPD-L1
L@mLuc	7.86E+11	37 ± 5	N/A
L@mBiME	8.79E+11	39 ± 6	N/A
PL@mRP182-Luc	6.97E+11	34 ± 6	116 ± 15
PL@mBiME	7.11E+11	35 ± 5	120 ± 15

Supplementary Table 3. Characterization of various nano-formulations.

Nano-formulations	Figure	Size (nm)	Zeta (mV)	Encapsulation (%)
L@mBiME	Fig. 2f,h	76.8 ± 5.8	−8.31 ± 0.34	94.75 ± 6.02
PL@mBiME	Fig.2g,i	86.9 ± 6.3	−8.15 ± 0.82	94.77 ± 2.28
PL@FITC-mBiME	Fig. 3a	85.6 ± 3.3	−8.52 ± 0.74	92.68 ± 3.71
PL@mBiME-T2A-EGFP	Fig. 3e-g, Supplementary Fig. 13-14	87.9 ± 8.8	−8.01 ± 0.58	92.88 ± 4.78
PL@mBiME-T2A-EGFP (DSPC)	Fig. 3e-g	86.5 ± 6.6	−11.19 ± 0.47	94.93 ± 3.05
PL@mRP182-Luc	Fig. 4e	85.6 ± 4.3	−8.48 ± 0.22	93.04 ± 3.76
L@Cy5-mBiME	Fig. 5a-c	76.3 ± 3.7	−8.82 ± 0.95	95.24 ± 2.14
PL@Cy5-mBiME	Fig. 5a-c	86.1 ± 2.3	−8.24 ± 0.40	94.69 ± 2.56
Cy7-L@mLuc	Fig. 5d-e	76.5 ± 6.5	−8.54 ± 0.39	93.90 ± 3.36
Cy7-PL@mLuc	Fig. 5d-e	86.6 ± 5.4	−8.41 ± 0.56	94.59 ± 3.84
L@mLuc	Supplementary Fig. 26	77.3 ± 7.1	−8.55 ± 0.63	93.12 ± 2.15
PL@mLuc	Supplementary Fig. 26	85.4 ± 3.7	−8.10 ± 0.97	95.61 ± 5.64
PL@mLuc (DSPC)	Fig. 5f-j	89.5 ± 4.2	−11.3 ± 1.35	93.69 ± 3.96
PL@mLuc w/o A2	Fig. 5f-j	87.3 ± 4.4	−8.05 ± 0.59	93.92 ± 4.80

Supplementary Table 4. The catalogue numbers of the antibodies used in this paper

Antibody	Catalogue numbers	Clone numbers
Anti-mouse PD-L1 (B7-H1)	Bio X Cell, #BE0101	10F.9G2
Purified anti-mouse CD16/32	Biolegend, #101302	93
Zombie NIR Fixable Viability Kit	Biolegend, #423106	n/a
PerCP anti-mouse CD45	Biolegend, #103163	30-F11
PE anti-mouse CD11b	Biolegend, #101208	M1/70
APC anti-mouse F4/80	Biolegend, #123116	BM8
Pacific Blue anti-mouse MHCII	Biolegend, #107620	M5/114.15.2
PE/Cyanine7 anti-mouse CD86	Biolegend, #105014	GL-1
FITC anti-mouse CD206	Biolegend, #141704	C068C2
FITC anti-mouse CD172a (SIRPα)	Biolegend, #144006	P84
FITC anti-mouse CD3	Biolegend, #100203	17A2
PE/Cyanine7 anti-mouse CD4	Biolegend, #100421	GK1.5
APC anti-mouse CD8a	Biolegend, #100711	53-6.7
APC anti-mouse CD11c	Biolegend, #117310	N418
PE/Cyanine7 anti-mouse/human CD44	Biolegend, #163607	QA19A43
PE anti-mouse CD62L	Biolegend, #161203	W18021D
PE anti-mouse NK-1.1	Biolegend, #156503	S17016D
APC anti-mouse CD11b	Biolegend, #101212	M1/70
FITC anti-mouse Ly-6G/Ly-6C (Gr-1)	Biolegend, #108405	RB6-8C5
FITC anti-mouse/human CD11b	Biolegend, #101206	M1/70
PerCP/Cyanine5.5 anti-mouse CD45	Biolegend, #103132	30-F11
Brilliant Violet 650 anti-mouse/human CD11b	Biolegend, #101259	M1/70
Brilliant Violet 421 anti-mouse CD86	Biolegend, #105032	GL-1
PE/Cyanine7 anti-mouse CD206	Biolegend, #141720	C068C2
PE anti-mouse CD163	Biolegend, #155307	S15049I
Latex beads, fluorescent red	Sigma, #L2778-1ML	n/a
PE Anti-Mouse HER2/ErbB2	Proteintech, #PE-FcA98153	241366E6
PE Rabbit IgG Isotype Control	Proteintech, #PE-FcA98136	240953C9
Anti-Polyethylene glycol	Abcam, #ab51257	PEG-B-47
Anti-PD-L1	Abcam, #ab213480	EPR20529
Anti-ErbB2/HER2	Abcam, #ab134182	EP1045Y
Anti-iNOS	Proteintech, #22226-1-AP	Polyclonal
Anti-Arginase-1	Proteintech, #16001-1-AP	Polyclonal
Beta Tubulin	Proteintech, #HRP-66240	1D4A4
Cofilin	Abcam, #ab42824	Polyclonal
