# Supplementary material for: Co-delivering macrophage engager mRNA and PD-L1 antibody via tumor-responsive nanoparticles for glioblastoma immunotherapy
Source: Nat Commun. 2026 Apr 11;17:5127. doi: 10.1038/s41467-026-71646-y (PMC13247073; doi:10.1038/s41467-026-71646-y)
Supplement: Supplementary file 2 — Reporting Summary [file 41467_2026_71646_MOESM2_ESM.pdf]

Corresponding author(s): Jinbing Xie and Yini Zhu

Last updated by author(s): Mar 18, 2026

Reporting Summary

Nature Portfolio wishes to improve the reproducibility of the work that we publish. This form provides structure for consistency and transparency in reporting. For further information on Nature Portfolio policies, see our Editorial Policies and the Editorial Policy Checklist.

Statistics

For all statistical analyses, confirm that the following items are present in the figure legend, table legend, main text, or Methods section.

n/a	Confirmed	
☐	☒	The exact sample size (*n*) for each experimental group/condition, given as a discrete number and unit of measurement
☐	☒	A statement on whether measurements were taken from distinct samples or whether the same sample was measured repeatedly
☐	☒	The statistical test(s) used AND whether they are one- or two-sided<br>*Only common tests should be described solely by name; describe more complex techniques in the Methods section.*
☐	☒	A description of all covariates tested
☐	☒	A description of any assumptions or corrections, such as tests of normality and adjustment for multiple comparisons
☐	☒	A full description of the statistical parameters including central tendency (e.g. means) or other basic estimates (e.g. regression coefficient) AND variation (e.g. standard deviation) or associated estimates of uncertainty (e.g. confidence intervals)
☐	☒	For null hypothesis testing, the test statistic (e.g. *F*, *t*, *r*) with confidence intervals, effect sizes, degrees of freedom and *P* value noted<br>*Give P values as exact values whenever suitable.*
☒	☐	For Bayesian analysis, information on the choice of priors and Markov chain Monte Carlo settings
☒	☐	For hierarchical and complex designs, identification of the appropriate level for tests and full reporting of outcomes
☐	☒	Estimates of effect sizes (e.g. Cohen's *d*, Pearson's *r*), indicating how they were calculated

Our web collection on statistics for biologists contains articles on many of the points above.

Software and code

Policy information about availability of computer code

Data collection	The size distribution and zeta potential was evaluated by dynamic light scattering measurements using a ZetaPlus (Brookhaven Instruments, New York, USA).The morphology was observed by transmission electron microscopy (TEM) (Hitachi H7650, Tokyo,Japan). The gel image was taken by Molecular Imager Gel Doc XR+ System (BioRad, California, USA). The flow-cytometry data were obtained by FACS Celesta (Becton, Dickson and Company, USA) or CytoFLEX LX (Beckman Coulter Life Sciences, Indianapolis, USA); A microplate reader (Multiskan Go, Thermo Fisher Scientific, Massachusetts, USA) was used to measure the absorbance at different nm. Fluorescence images were detected by Laser Scanning Confocal Microscopy (Olympus FV3000, Tokyo, Japan) ; IVIS luminescence image was captured by in vivo imaging machine(Tanon ABL-X5, Shanghai, China); In vivo MR was performed using a 7.0-Tesla small animal MR scanner (Bruker Pharmascan, Ettlingen, Germany)
Data analysis	The data are processed by Origin software version 2026 (OriginLab Corperation, Northampton, Massachusetts, USA).

For manuscripts utilizing custom algorithms or software that are central to the research but not yet described in published literature, software must be made available to editors and reviewers. We strongly encourage code deposition in a community repository (e.g. GitHub). See the Nature Portfolio guidelines for submitting code & software for further information.

Data

Policy information about availability of data

All manuscripts must include a data availability statement. This statement should provide the following information, where applicable:

- Accession codes, unique identifiers, or web links for publicly available datasets
- A description of any restrictions on data availability
- For clinical datasets or third party data, please ensure that the statement adheres to our policy

All data supporting the findings of this study are available within the paper and its Supplementary Information files or from the corresponding author upon reasonable request. Source data are provided within this paper.

Research involving human participants, their data, or biological material

Policy information about studies with human participants or human data. See also policy information about sex, gender (identity/presentation), and sexual orientation and race, ethnicity and racism.

Reporting on sex and gender	*Use the terms sex (biological attribute) and gender (shaped by social and cultural circumstances) carefully in order to avoid confusing both terms. Indicate if findings apply to only one sex or gender; describe whether sex and gender were considered in study design; whether sex and/or gender was determined based on self-reporting or assigned and methods used.*<br>*Provide in the source data disaggregated sex and gender data, where this information has been collected, and if consent has been obtained for sharing of individual-level data; provide overall numbers in this Reporting Summary. Please state if this information has not been collected.*<br>*Report sex- and gender-based analyses where performed, justify reasons for lack of sex- and gender-based analysis.*
Reporting on race, ethnicity, or other socially relevant groupings	*Please specify the socially constructed or socially relevant categorization variable(s) used in your manuscript and explain why they were used. Please note that such variables should not be used as proxies for other socially constructed/relevant variables (for example, race or ethnicity should not be used as a proxy for socioeconomic status).*<br>*Provide clear definitions of the relevant terms used, how they were provided (by the participants/respondents, the researchers, or third parties), and the method(s) used to classify people into the different categories (e.g. self-report, census or administrative data, social media data, etc.)*<br>*Please provide details about how you controlled for confounding variables in your analyses.*
Population characteristics	*Describe the covariate-relevant population characteristics of the human research participants (e.g. age, genotypic information, past and current diagnosis and treatment categories). If you filled out the behavioural & social sciences study design questions and have nothing to add here, write "See above."*
Recruitment	*Describe how participants were recruited. Outline any potential self-selection bias or other biases that may be present and how these are likely to impact results.*
Ethics oversight	*Identify the organization(s) that approved the study protocol.*

Note that full information on the approval of the study protocol must also be provided in the manuscript.

Field-specific reporting

Please select the one below that is the best fit for your research. If you are not sure, read the appropriate sections before making your selection.

☒ Life sciences ☐ Behavioural & social sciences ☐ Ecological, evolutionary & environmental sciences

For a reference copy of the document with all sections, see nature.com/documents/nr-reporting-summary-flat.pdf

Life sciences study design

All studies must disclose on these points even when the disclosure is negative.

Sample size	The sample size for each experiment is specified in the figure legend.
Data exclusions	No data was excluded.
Replication	All experiments were repeated for at least three times and experimental findings were reproducible.
Randomization	All samples and organisms were randomly allocated into the experimental groups.
Blinding	The investigators were blinded to group allocation during data collection and/or analysis.

Reporting for specific materials, systems and methods

We require information from authors about some types of materials, experimental systems and methods used in many studies. Here, indicate whether each material, system or method listed is relevant to your study. If you are not sure if a list item applies to your research, read the appropriate section before selecting a response.

Materials & experimental systems

n/a	Involved in the study
☐	☒ Antibodies
☐	☒ Eukaryotic cell lines
☒	☐ Palaeontology and archaeology
☐	☒ Animals and other organisms
☒	☐ Clinical data
☒	☐ Dual use research of concern
☒	☐ Plants

Methods

n/a	Involved in the study
☒	☐ ChIP-seq
☐	☒ Flow cytometry
☒	☐ MRI-based neuroimaging

Antibodies

Antibodies used	Anti-mouse PD-L1 was purchased from Bio X Cell (Cat. No. BE0101, New Hampshire, USA).<br>CD16/CD32 monoclonal antibody (Cat. No. 101302) and the antibodies used for flow cytometry were specific for Zombie NIR™ Fixable Viability Kit (Cat. No. 423106), CD45 (Cat. No. 103163), CD11b (Cat. No. 101208, 101212, 101206), F4/80 (Cat. No. 123116), MHC Ⅱ (Cat. No. 107620), CD86 (Cat. No. 105014, 105032), CD206 (Cat. No. 141704, 141720), SIRPα (Cat. No. 144006), CD3 (Cat. No. 100203), CD4 (Cat. No. 100421), CD8 (Cat. No. 100711), CD11c (Cat. No. 117310), CD44 (Cat. No. 163607), CD62L (Cat. No. 161203), NK (Cat. No.156503), Gr-1 (Cat. No. 108405) and CD163 (Cat. No. 155307) were purchased from BioLegend (California, USA). Anti-Mouse HER2/ErbB2 (Cat. No. PE-FcA98153) and Rabbit IgG Isotype Control (Cat. No. PE-FcA98136) were purchased from Proteintech for flow cytometry. Anti-Polyethylene glycol (Cat. No. ab51257), Anti-PD-L1 (Cat. No. ab213480) and Cofilin (Cat. No. ab42824) were purchased from Abcam for Western blot. Anti-iNOS (Cat. No. 22226-1-AP), Anti-Arginase-1(Cat. No. 16001-1-AP) and Beta Tubulin (Cat. No. HRP-66240) were purchased from Proteintech for Western blot.
Validation	All the antibodies used are from commercial sources and have been validated by the vendors.

Eukaryotic cell lines

Policy information about cell lines and Sex and Gender in Research

Cell line source(s)	The murine glioma cell line GL261 (Cat# KGG2236-1) and the murine macrophage cell line RAW 264.7 (Cat# KGG2201-1) were obtained from KeyGEN Biotech Co., Ltd. (Jiangsu, China). The mouse brain endothelial cell line bEnd.3 (Cat# CL-0598) was obtained from Wuhan Procell Biotechnology Co., Ltd. (Wuhan, Hubei, China). The murine glioma cell line CT-2A (Cat# JY-Y1455) was obtained from Shanghai Jinyuan Biotechnology Co., Ltd. (Shanghai, China). GL261-Luc and CT-2A-Luc cells were generated by infecting cells with Lenti-EF1α-Luc-T2A-Puro virus (Banma Biotechnology Co., Ltd., Cat# BM2024012201) and selecting with 2 μg/mL puromycin (Beyotime, Cat# ST551-10mg). All cells were cultured in DMEM (HyClone) supplemented with 10% (v/v) fetal bovine serum (FBS; Gibco) and 100 U/mL penicillin/streptomycin (Invitrogen). Cell lines were authenticated by the vendors and were routinely tested for mycoplasma contamination. All cells used in this study were confirmed to be mycoplasma-negative.
Authentication	The cell lines used were authenticated.
Mycoplasma contamination	All the cell line are mycoplasma negative.
Commonly misidentified lines (See ICLAC register)	No misidentified lines were used.

Animals and other research organisms

Policy information about studies involving animals; ARRIVE guidelines recommended for reporting animal research, and Sex and Gender in Research

Laboratory animals	C57BL/6 mice (6–8 weeks old) were purchased from Jiangsu Huachuang Sino Pharma Tech Co., Ltd.
Wild animals	The study did not involve wild animals.
Reporting on sex	Gender preference analysis was not performed for ethical reasons and the study is gender-neutral.
Field-collected samples	The study did not involve samples collected from the field
Ethics oversight	All animal experiments were conducted according to the ethical guidelines of the Animal Care & Welfare Committee of Southeast University, Jiangsu, China (NO. 20220316049).

Note that full information on the approval of the study protocol must also be provided in the manuscript.

Plants

Seed stocks

Report on the source of all seed stocks or other plant material used. If applicable, state the seed stock centre and catalogue number. If plant specimens were collected from the field, describe the collection location, date and sampling procedures.

Novel plant genotypes

Describe the methods by which all novel plant genotypes were produced. This includes those generated by transgenic approaches, gene editing, chemical/radiation-based mutagenesis and hybridization. For transgenic lines, describe the transformation method, the number of independent lines analyzed and the generation upon which experiments were performed. For gene-edited lines, describe the editor used, the endogenous sequence targeted for editing, the targeting guide RNA sequence (if applicable) and how the editor was applied.

Authentication

Describe any authentication procedures for each seed stock used or novel genotype generated. Describe any experiments used to assess the effect of a mutation and, where applicable, how potential secondary effects (e.g. second site T-DNA insertions, mosiacism, off-target gene editing) were examined.

Flow Cytometry

Plots

Confirm that:

- [x] The axis labels state the marker and fluorochrome used (e.g. CD4-FITC).
- [x] The axis scales are clearly visible. Include numbers along axes only for bottom left plot of group (a 'group' is an analysis of identical markers).
- [x] All plots are contour plots with outliers or pseudocolor plots.
- [x] A numerical value for number of cells or percentage (with statistics) is provided.

Methodology

Sample preparation

For flow tissue testing, the brain tissue is carefully removed and cleaned with PBS. Collagenase D (0.5 μg/mL), DNase1 (0.5 μg/mL from Vazyme Biotec Co., Ltd, China; 3 μg/mL from Sigma Aldrich, USA), and a lymphocyte isolation solution (17-5442-03, GE, USA) were used to isolate brain-infiltrating immune cells. Afterwards, red blood cell lysate was added for centrifugation to remove red blood cells. The mouse tumor-infiltrating tissue lymphocyte extraction kit was then used to obtain a total of 1 x 106 cells in each tube according to the instructions, and then closed with CD16/32 for 15 minutes. Then stain using Zombie NIR ™ Fixable Viability Kit for 20 min, after which CD45, CD3, CD4, CD8, CD11b, CD11c, F4/80, CD25, CD86, MHCII, Gr-1, and NK1.1 were added to cells and incubated in the dark for half an hour. In order to stain CD206 and Foxp3, additional nucleoclast staining is required. Thereafter, stained cells were analyzed by flow cytometry (CytoFLEX, Beckman Coulter Life Sciences, Indianapolis, USA and FACS Celesta, Becton, Dickinson and Company, New Jersey, USA) for macrophages, CD8+ T cells, Tregs, mature DC, NK, and MDSC populations.

For evaluation of Kupffer cells in the liver, the following procedure was performed following three administrations of PL@mBiME. The mouse liver was harvested and digested in 750 μg/ml Collagenase IV (Gibco) at 37°C for 1 hour. The resulting cell suspension was treated with red blood cell lysis buffer for 5 minutes at room temperature, followed by centrifugation (360 × g, 5 minutes, 4°C) to obtain a single-cell suspension. Cells were blocked with anti-CD16/32 for 15 minutes according to the manufacturer's protocol, followed by staining with Zombie NIR™ Fixable Viability Kit for 20 minutes. Surface staining was performed using antibodies against CD45, CD11b, F4/80, CD86, and CD163 for 30 minutes in the dark. In addition, CD206 staining required an additional nuclear permeabilization step. Stained cells were analyzed on a flow cytometer (CytoFLEX, Beckman Coulter Life Sciences, Indianapolis, USA) to identify the Kupffer cell population. To assess mRNA expression in liver Kupffer cells, mice were injected with PL@mBiME-T2A-EGFP. Twelve hours later, livers were carefully harvested and processed into single-cell suspensions using the same digestion and lysis protocol described above. Cells were similarly blocked with anti-CD16/32 and stained with Zombie NIR™ viability dye. They were then incubated with antibodies against CD45, CD11b, and F4/80 for 30 minutes in the dark. Flow cytometric analysis was performed on the CytoFLEX to identify and analyze Kupffer cells for reporter expression.

For immunofluorescence staining, mice were sacrificed and tumor tissue was removed, further fixed in 4% paraformaldehyde, embedded in paraffin, and then cut into slides. To evaluate M1 macrophages in GBM, tumor sections were labeled as F4/80 and CD86 (blue: DAPI; red: CY3; green: Alexa fluor 488). To evaluate M2 macrophages in GBM, tumor sections were labeled as F4/80 and CD206 (blue: DAPI; red: CY3; green: Alexa fluor 488). To evaluate CD8+ T cells in GBM, tumor sections were labeled as CD4, and CD8 (blue: DAPI; green: SpGreen-CD4; purple: Cy5-CD8).

To detect the expression of relevant inflammatory factors in brain tissue, tumor tissues were collected, homogenized, and centrifuged. The secretion of IFN-γ, , IL-10, IL-1-β and TNF-α in brain tissue were measured using an ELISA kit according to manufacturer's instructions.

Instrument

FACS Celesta, Becton, Dickinson and Company, New Jersey, USA and CytoFLEX, Beckman Coulter Life Sciences, Indianapolis, USA

Software

FlowJo_v10.8.1

Cell population abundance

mouse tumor infiltrating tissue lymphocyte extraction kit was used during sample preparation, no sorting was performed

Gating strategy

As illustrated in Supplementary Fig. 38, we first utilized FSC-A and SSC-A to gate the cells, followed by SSC-A and SSC-H for single-cell gating. Next, we applied live-dead gating to identify viable cells, followed by CD45 positive staining. For markers without obvious positive and negative population boundaries, a Fluorescence Minus One (FMO) control was employed to establish the boundaries between positive and negative populations.

☒ Tick this box to confirm that a figure exemplifying the gating strategy is provided in the Supplementary Information.
