# Supplementary material for: Co-delivering macrophage engager mRNA and PD-L1 antibody via tumor-responsive nanoparticles for glioblastoma immunotherapy
Source: Nat Commun. 2026 Apr 11;17:5127. doi: 10.1038/s41467-026-71646-y (PMC13247073; doi:10.1038/s41467-026-71646-y)
Supplement: Supplementary file 3 — Transparent Peer Review file [file 41467_2026_71646_MOESM3_ESM.pdf]

nature portfolio

Peer Review File

Co-delivering macrophage engager mRNA and PD-L1 antibody via tumor-responsive nanoparticles for glioblastoma immunotherapy

Corresponding Author: Professor Jinbing Xie

This file contains all reviewer reports in order by version, followed by all author rebuttals in order by version.

Version 0:

Reviewer comments:

Reviewer #1

(Remarks to the Author)
The manuscript by Zhang et al describes generation and therapeutic efficacy of a macrophage engager in combination with anti-PD-L1 blocker, both encapsulated in a lipid nanoparticle, named PL@mBiME. The particles were optimized by application of angiopep-2 peptide decoration (to promote blood-brain-barrier transport) and pH- and glutathione-responsiveness (both to promote tumor-selective targeting). The lipid nanoparticles were in average 80 nm in diameter and were validated in vitro using several quality control assays. Proper controls were taken along in all assays including luciferase encoding mRNA for in vivo work. Therapeutic efficacy and tumor-microenvironment analysis demonstrate dependence on the macrophage engager and nanoparticle encapsulation. The PL@BiME compound alters the immune landscape of tumors for macrophage type (more M1) and T cell infiltrate (less Treg and more CD8 T cells). The data are comprehensive and well presented and the paper is well written.
A few comments:
• Fig 3 Evidence for reprogramming of bone marrow derived macrophages in vitro from M2 to M1 is limited. Authors do not show CD206 expression or other M2 markers.
• Fig 4i indicates fluorescence on the y-axis, but bioluminescence in the legend. Please correct.
• Fig S18b depict biodistribution of the pharmaceutical in organs after i.v. injection using luciferase mRNA containing particles. But the graph does not include measurements in the tumors and therefore the values of liver can not be related to tumor. Include the quantification of PL@mLuc in tumors in the graph.
• Lipid nanoparticles also accumulate in livers that is populated with many CD206+ Kupffer cells. Are these cells affected by treatment?
• Fig 5e shows survival of orthotopic glioblastoma-bearing mice. What is the criterium of survival? M&M states that none of the mice reached maximum tumor size of 100 mm3, so on what criterium is survival based?
• Fig 6. What is the frequency and number of total F4/80+ cells of the immune infiltrate of the tumors?
• Glioblastoma-bearing brain vasculature is different from that of healthy brains. So the lipid nanoparticles might cross the blood-brain barrier independent of the angiopep-2 peptide decoration. The additional benefit of the angiopep-2 peptide might be tested in in tumor-free brains.
• The impact of the nanoparticles on the immune infiltrate is well performed, but does not reveal the ultimate effector population responsible for tumor control. Authors may perform subset depletion studies, removing immune cell populations (e.g. macrophages, CD8 T cells, etc).
• The discussion section lacks comparison with recently published literature on tyrosine kinase inhibitors to M-CSF receptor that have been successfully applied in glioblastomas.
• Row 204 contains a typo ‘helpher’.

Reviewer #2

(Remarks to the Author)
This manuscript reports the development of an acid-responsive lipid nanoparticle (PL@mBiME) for co-delivery of bispecific macrophage engager mRNA and PD-L1 antibody in glioblastoma therapy. The study is rigorous, well executed, and

addresses critical barriers such as BBB penetration and TAM-driven immunosuppression. Notably, the work introduces a macrophage engager, an area far less explored than T-cell engagers, demonstrates the efficiency of in vivo engager expression via mRNA to bypass complex protein production, and employs a novel CD206-targeting peptide. These advances, together with the elegant nanoparticle design, position this study as an original and impactful contribution to mRNA-based immunotherapy.

I have a few suggestions that could further strengthen the manuscript:
1. While PL@mBiME is well characterized and shows impressive therapeutic outcomes, it would be valuable to directly compare its delivery efficacy and biodistribution with conventional DSPC-based LNPs in vivo. Such data would better highlight the translational advantage of this acid-responsive LNP design over existing platforms.
2. In Figure 4j, luciferase signals are still present at 72 hours, suggesting sustained protein expression. Since persistence of mRNA-derived protein expression is an important parameter for translational application, it would be interesting to examine how long the protein expression lasts in the brain and whether this correlates with therapeutic benefit.
3. The in vivo data (Figure 6l–n) and enhanced macrophage engulfment (Figure 3g) indirectly support that BiME connects tumor cells with macrophages. To provide more direct validation, I recommend including an in vitro co-culture experiment of tumor cells and macrophages, assessing whether the frequency of physical macrophage–tumor cell conjugates increases upon BiME expression.
4. Since BiME bridges macrophages and tumor cells via CD206 and HER2, it would be informative to test whether phagocytosis efficiency depends on HER2 expression levels. Specifically, assessing BiME-mediated macrophage activity against HER2^high versus HER2^low tumor cells could clarify the selectivity and potential scope of this strategy.

Reviewer #3

(Remarks to the Author)
This study presents a multifaceted approach to overcoming the challenges of treating solid tumors like glioblastoma. The PL@mBiME combines several strategies into a single system. By shifting the focus from T-cells to engaging macrophages with a bispecific mRNA construct, the authors circumvented critical barriers such as poor T-cell infiltration and systemic toxicity. The dual targeting of ErbB2 on glioma cells and CD206 on M2 macrophages is a promising strategy that not only directs the immune attack but also reprograms the immunosuppressive tumor microenvironment, enhancing phagocytosis and antigen presentation. Furthermore, the incorporation of pH-responsive charge reversal for improved tumor accumulation and lysosomal escape ensures efficient mRNA expression. The addition of a glutathione-triggered, surface-conjugated aPD-L1 antibody provides a localized and potent immune checkpoint blockade to counteract suppression and sustain anti-tumor immunity. This work offers a therapeutic strategy that could improve outcomes for patients with GBM and potentially other solid malignancies. However, following issues should be addressed.
1. In Figure 1j, what is the in vivo GSH concentration? Does it significantly exceed 10 mM? When is the PD-L1 antibody released—before or after cellular uptake?
2. "Furthermore, enzyme-linked immunosorbent assay (ELISA) analysis revealed that treatment with BiME significantly upregulated the secretion of pro-inflammatory cytokines IL-1β, TNF-α, and IFN-γ, while simultaneously downregulating the anti-inflammatory cytokine IL-10 (Figure 3j-l). " There are no IFN-γ data presented here. Additionally, based on the sequence of the results description, the IL-10 data should be placed at the end rather than in the middle.
3. The authors found that PL@mBiME enhances the physical interaction between macrophages and tumor cells and increases phagocytic activity. While this represents a major innovative aspect of the study, the experimental evidence provided is insufficient. Further in vitro and in vivo evidence is required to substantiate this conclusion.
4. In Fiugre 5，the effects of PL@mBiME on the levels of CD8+ T cells and Granzyme B should be determined by IHC.
5. A single GBM model is insufficient. It is necessary to validate the efficacy of PL@mBiME in at least one additional model. Since PL@mBiME is more effective under acidic pH conditions, it would be interesting to examine its efficacy in glycolytic tumors compared to non-glycolytic tumors, or in control versus LDHA-knockout tumors.
6. What is the mechanism for the durable immune memory generated by PL@mBiME in GBM?
7. As PL@mBiME targets GBM in the brain, its potential effects on cognitive function should be evaluated in mouse models.

Reviewer #4

(Remarks to the Author)
The authors report a new and highly complex nanoparticle platform for targeted delivery to the brain to treat glioblastoma. The authors make several claims about their nanodelivery system, which features 1) peptide targeting to cross the blood-brain barrier, 2) a glutathione cleavable linker to release anti-PDL1 extracellularly, 3) an acidity-sensitive lipid that supports lysosomal escape and delivery of mRNA cargo into tumor and non-tumor cells. The mRNA cargo encodes a bispecific macrophage engager targeting ErbB2 on tumor cells and CD206 on macrophages.
The study is timely and addresses an unmet need in cancer using a new nanoparticle platform, however characterization of this platform is not adequate to support the authors' claims. The in vivo data showing efficacy of their system is impressive and encouraging; however, there are major concerns that may confound the interpretation of these results and subsequently diminishes the impact of this work.

Major Concerns
1. The nanoparticle system is exceptionally complex, and requires more robust characterization. The authors are encouraged to review the 2018 paper in Nature Nanotechnology “Minimum information reporting in bio-nano experimental literature,” PMID: 30190620. Given the complexity of this system, Figure 1 is not adequate. The encapsulation of efficiency of mRNA is addressed, but characterization of A2 and aPDL1 are lacking. The conjugation conditions are not provided, nor

any assays to show that the peptide and antibody are stably covalently conjugated. Similarly, multiple assays use modifications of the platform, with fluorescent labels, reporter mRNA, and variations on their mBiME. These modifications can alter the structure and function of lipid nanoparticles, and it was challenging to follow exactly which LNP variation was being tested throughout the paper. The methods do not report how fluorescent dyes were conjugated to lipids or antibodies or show whether these conjugation strategies impacted nanoparticle size, charge, encapsulation, etc.

2. The choice of tumor model and mBiME target are incongruent and likely confound the interpretation of the study results. While commonly used and well-characterized for immunotherapy studies in glioblastoma, GL261 is not representative of most human GBM tumors, and does not natively express ErbB2/Her2. The authors do not appear to have genetically engineered GL261 to express human ERBB2/HER2 (as is common when this model is used to study HER2-directed agents), and do not present characterization data that either the murine or human target is expressed in their model. Figure 3 implies that HER2 is present on the GL261 but there is no data presented to support this. Figure 6i is the only figure staining for ERBB2 and no isotype control is shown; methods do not include antibody information for this antibody. Experiments in which exogenous ErbB2 are added in vitro do not validate that the target is present. In this context, it is not possible to interpret their results showing exceptional responses to PL@mBiME in figure 5. While several appropriate controls were included, the authors did not include any LNP controls without the anti-HER2 component of the mBiME (other than mLuc). It is possible that in this system the other components of the mBiME (RP182 peptide, CD8 signal peptide, EGFP) and anti-PDL1 are responsible for these responses as opposed to HER2-specific interactions. Indeed, expression of luciferase alone has been shown to elicit immune responses in this model (PMID 32620877). Further testing of this system in a glioma model in which the target is present and validated, and with appropriate controls to better understand the importance of each element of the mBiME would significantly increase the impact of this work. This reviewer acknowledges that the authors have attempted to characterize the immune microenvironment in Figures 7 and 8, but these experiments do not confirm a specific mechanism of immune activation that is specific to the cargo delivered in the LNP.

Minor Concerns
1. Benchmarking to existing literature: The manuscript does not clearly articulate its incremental advance over prior studies. Explicitly state how this work differs from and improves upon existing literature.

2. Experimental Design & Quantification:
- Methods for nanoparticle synthesis are not sufficient to allow researchers to replicate your methods. Equipment, mixing method and speed, temperature, concentration, etc were not described.
- Characterization of DSPE-PEG-A2 is incomplete – how was conjugation confirmed? How many molecules of A2 are present, on average, per molecule. Similarly, how many molecules of aPDL1 are on each nanoparticle?
- Figures 3 and 4 both attempt to show mechanistically how the nanoparticles deliver their cargo to specific cells, but ultimately it is not clear how and when the GSH-mediated cleavage and charge reversal are occurring. In the in vitro setting at least, this could be further probed through pre-incubation of LNPs with GSH to isolate the role of this cleavage as was explored for pH in Fig 2
- The use of 'docking studies' in 3m does not prove mechanism, and inclusion is questionable in this paper
- Quantitative analyses (e.g., Pearson/Manders coefficients, permeability metrics) would strengthen the conclusions of imaging data.
- Clearly report the number of biological and technical replicates.
- The authors only include male mice, which is not appropriate for a preclinical evaluation in a tumor that impacts both sexes.
- While toxicity was not the focus of this paper, it would be expected based on the biodistribution data reported that the mBiME is expressed in the liver. Staining for mBiME and anti-PDL1 should be evaluated using tissue in S24.

3. Data Presentation
- Several figures lack scale bars, error bars, or statistical details.
- Some conclusions over-interpret correlation as causation. E.g. "Further experiments confirmed that DCPA-based LNPs mediated efficient EGFP expression in…" refers to a supplemental micrograph without replicates or quantification.

4. Discussion & Context
- Place results within broader literature, acknowledging supporting and conflicting reports. This is especially important given controversy over the importance of ErbB2 in GBM, and also regarding the choice of GL261 which is known to have multiple mechanisms of immune activation in vivo.
- Explicitly state study limitations, including in vitro vs. in vivo relevance and choice of models.

Specific Figure Comments
Figure 1
- Given that the formulation contains an ionizable lipid (SM-201, ~50%) with a pKa of ~6.5, it is unclear why pH-responsive charge reversal appears strongly dependent on DCPA (~10%). Clarify whether a zeta potential of –5 or +5 mV is considered high for nanoparticles, especially in low ionic strength media.
- TEM images lack scale bars, appear very small, and are of low quality.
- The effect of adding aPD-L1 on nanoparticle charge is not clearly demonstrated; only size changes are reported. Please include supporting data on charge measurements.
- Stability studies in 10% FBS, DMEM, and PBS at 4 °C may not reflect physiologically relevant conditions. Justify this choice or consider repeating at room temperature or 37 °C.

Figure 2
- Reduced colocalization of green nanoparticle signal with red LysoTracker is presented as evidence of lysosomal escape. However, if true cytosolic release occurs, the green fluorescence should appear diffuse rather than punctate. In the DCPA group at 4 h, the green signal remains vesicular, raising questions about the extent of lysosomal escape and requiring clarification.

Figure S13
- Macrophage polarization should produce distinct populations (M1 vs. M2), yet no clear separation is visible. Gating strategy and data interpretation need clarification.

Figure 4 (B–C)
- Comparison groups are insufficient. Using mRNA alone or antibody alone does not convincingly demonstrate the role of Angiopep-2 (A2). Direct comparison between nanoparticles with and without A2 is recommended to assess BBB transport.
Figure 4 (D–K)
- Separating brain from other organs makes relative biodistribution difficult to interpret. Data should be reported as percent injected dose if possible, or at a minimum the reporting the proportion of nanoparticles reaching the brain relative to other organs would be more informative.

Additional Point
- Figure 5g is referenced in the text but is missing from the manuscript. This inconsistency should be addressed.

Reviewer #5

(Remarks to the Author)
I co-reviewed this manuscript with one of the reviewers who provided the listed reports. This is part of the Nature Communications initiative to facilitate training in peer review and to provide appropriate recognition for Early Career Researchers who co-review manuscripts.

Version 1:

Reviewer comments:

Reviewer #1

(Remarks to the Author)
the authors have comprehensively attended my concerns by performing critical new experiments. results are incorporated in the new version and clearly improved the scientific rigor.

Reviewer #2

(Remarks to the Author)
The authors have addressed all my concerns. The manuscript is now suitable for publication in Nature Communications.

Reviewer #3

(Remarks to the Author)
The authors addressed all my concerns.

Reviewer #4

(Remarks to the Author)
The authors have done a substantial amount of work to address nearly all of our concerns. Specifically, ensuring that their model systems are accurately characterized for expression of the antigens of interest and that appropriate controls are included in key in vivo studies. The addition of a second murine model confirming their results is noted and appreciated.

Our other major concern regarding nanoparticle characterization was also well-addressed by the addition of comprehensive methods and supporting characterization data that meets or exceeds the minimum reporting requirements accepted in the nanoparticle field.

In summary, the revised manuscript addresses our concerns and is appropriate for publication in Nature Communications.

Reviewer #5

(Remarks to the Author)
I co-reviewed this manuscript with one of the reviewers who provided the listed reports. This is part of the Nature Communications initiative to facilitate training in peer review and to provide appropriate recognition for Early Career

Researchers who co-review manuscripts.

Open Access This Peer Review File is licensed under a Creative Commons Attribution 4.0 International License, which permits use, sharing, adaptation, distribution and reproduction in any medium or format, as long as you give appropriate credit to the original author(s) and the source, provide a link to the Creative Commons license, and indicate if changes were made.
In cases where reviewers are anonymous, credit should be given to 'Anonymous Referee' and the source.
The images or other third party material in this Peer Review File are included in the article’s Creative Commons license, unless indicated otherwise in a credit line to the material. If material is not included in the article’s Creative Commons license and your intended use is not permitted by statutory regulation or exceeds the permitted use, you will need to obtain permission directly from the copyright holder.
To view a copy of this license, visit https://creativecommons.org/licenses/by/4.0/

Point to Point Response Letter

Dear Editor and Reviewers,
Please note that Reviewer comments are shown in black, our responses in blue.

REVIEWER COMMENTS

Reviewer #1 (Remarks to the Author):

The manuscript by Zhang et al describes generation and therapeutic efficacy of a macrophage engager in combination with anti-PD-L1 blocker, both encapsulated in a lipid nanoparticle, named PL@mBiME. The particles were optimized by application of angiopep-2 peptide decoration (to promote blood-brain-barrier transport) and pH- and glutathione-responsiveness (both to promote tumor-selective targeting). The lipid nanoparticles were in average 80 nm in diameter and were validated in vitro using several quality control assays. Proper controls were taken along in all assays including luciferase encoding mRNA for in vivo work. Therapeutic efficacy and tumor-microenvironment analysis demonstrate dependence on the macrophage engager and nanoparticle encapsulation. The PL@BiME compound alters the immune landscape of tumors for macrophage type (more M1) and T cell infiltrate (less Treg and more CD8 T cells). The data are comprehensive and well presented and the paper is well written.

Response：
We sincerely appreciate your thorough review, positive evaluation, and constructive feedback on our manuscript. Your recognition of the comprehensiveness of our data, clarity of presentation, and the scientific significance of our work is greatly encouraging. All the points you highlighted have been carefully noted. We will maintain the clarity and completeness of the data presentation and manuscript writing in the revised version. For any minor adjustments required by the editorial process, we will address them promptly and meticulously.

A few comments:
• Fig 3 Evidence for reprogramming of bone marrow derived macrophages in vitro from M2 to M1 is limited. Authors do not show CD206 expression or other M2 markers.
Response: We fully acknowledge that the original version of our manuscript lacked direct evidence for M2 macrophage markers, leading to an insufficiently supported

conclusion regarding the limited reprogramming of bone marrow-derived macrophages (BMDMs) from M2 to M1 phenotype in vitro. To address this critical gap, we have supplemented key experimental data and revised the corresponding description of Figure 3 as detailed below:

1. We performed flow cytometry analysis of CD206 expression (a canonical M2 marker) in BMDMs treated with various formulations. Representative flow plots and quantitative statistical results are now included in **Figure R1a-b**, which directly reflect the proportion of M2-polarized cells in each group.
2. Based on the flow cytometry data of M1 markers (CD86) and M2 markers (CD206), we calculated the M1/M2 ratio for each experimental group and incorporated these quantitative data into **Figure R1c**. This ratio provides a more intuitive and objective indicator of macrophage polarization trends.
3. We added Western blot assays for Arg-1 (a classic M2 functional marker) and iNOS (a core M1 functional marker) at the protein level. The results, presented in **Figure R1d-e**, clearly show the dynamic changes of M1/M2 marker expression upon treatment, complementing the flow data.

The data has been updated in **Supplementary Fig. 19 and Supplementary Fig. 20.** The corresponding description is updated in our revised manuscript at **Lines 264-273**.

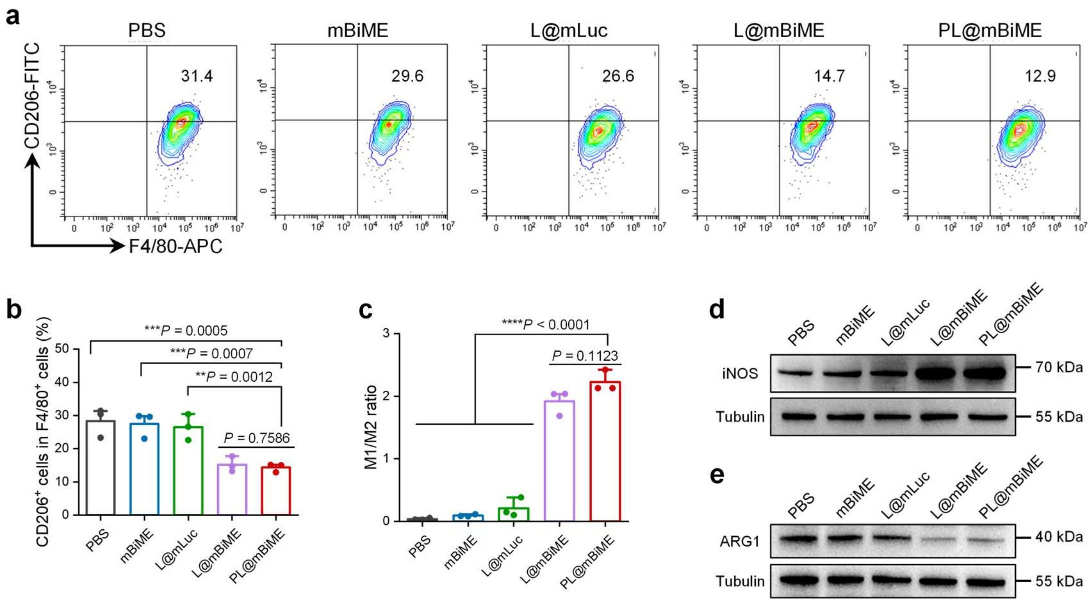

Figure R1. Flow cytometry and western blot analysis of macrophage polarization. **a** Representative flow cytometry dot plots showing the proportion of M2 macrophages ($F4/80^+$ $CD206^+$) after treatment with different formulations. **b** Quantitative analysis of the M2 macrophage polarization ratio. **c** M1/M2 ratio of BMDMs treated with various formulations, n = 3 biologically independent replicates. **d, e** Analysis of M1 and M2 phenotypic markers in BMDMs by western blot. BMDMs were treated with different

formulations, and the expression levels of iNOS (a, M1 marker) and Arg-1 (b, M2 marker) were assessed. Uncropped blots in Source Data. Data are presented as mean ± SD, statistical analysis was performed by one-way ANOVA with Fisher's LSD post-hoc test.

• Fig 4i indicates fluorescence on the y-axis, but bioluminescence in the legend. Please correct.

Response: Thank you for pointing this out. We apologize for the inconsistency and have revised the figure legend from "bioluminescence" to "fluorescence" to align with the y-axis label and the actual experimental data (now shown in **Supplementary Fig. 25c, d**). Furthermore, we have conducted a thorough check of all figures and their corresponding legends throughout the manuscript to ensure that similar inconsistencies are not present elsewhere.

• Fig S18b depict biodistribution of the pharmaceutical in organs after i.v. injection using luciferase mRNA containing particles. But the graph does not include measurements in the tumors and therefore the values of liver can not be related to tumor. Include the quantification of PL@mLuc in tumors in the graph.

Response:

We sincerely thank the reviewer for this constructive suggestion, which is important for strengthening the translational relevance of our biodistribution analysis. We agree that the absence of tumor tissue quantification limits direct comparison of nanoparticle accumulation between tumors and other organs.

To address this concern, we performed additional experiments in which tumor tissue (brain tumor), along with the heart, liver, spleen, lung, and kidney, was subjected to ex vivo bioluminescence imaging and quantitative analysis following intravenous administration of PL@mLuc (Figure R2). The results showed that luciferase expression was highest in the liver, followed by the brain tumor and spleen, enabling a direct comparison of nanoparticle accumulation across tumors and major organs.

Moreover, we also further explored the biodistribution of PL@mLuc formulations with or without the A2 peptide in both tumor-free and GBM-bearing mice. In tumor-free mice, PL@mLuc without A2 exhibited negligible bioluminescence in the brain (Fig. R2a), consistent with an intact blood-brain barrier (BBB) and minimal brain accumulation. In GBM-bearing mice, PL@mLuc without A2 showed a low but detectable brain signal (Fig. R2a), likely resulting from local BBB disruption induced by the tumor. Notably, the inclusion of the A2 peptide in PL@mLuc increased brain-associated bioluminescence by approximately 9.8-fold compared with the non-targeted formulation (Fig. R2b), highlighting the essential role of A2 in promoting efficient BBB penetration and brain delivery. Moreover, in GBM-bearing mice, the

bioluminescence signal generated by the standard PL@mBiME formulation was approximately 1.7-fold higher in the brain compared to the PL@mBiME (DSPC) variant, while being reduced in the spleen, (Fig. R2b), indicating that the incorporation of DCPA markedly enhanced mRNA expression specifically within the tumor region. The corresponding description has been incorporated into the manuscript (**lines 402-413**), and the results have been updated in **Figure 4h–j**.

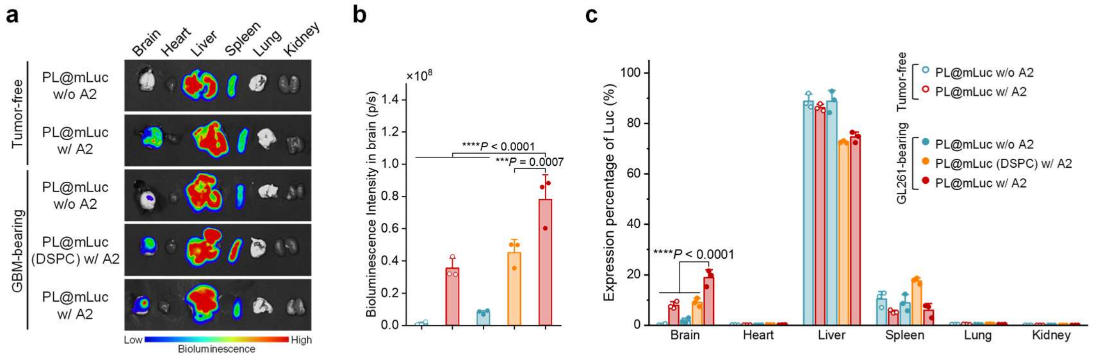

Figure R2. **a** Representative bioluminescence imaging of isolated brains from tumor-free versus GBM-bearing mice at 12 h post-administration of various formulations. **b** Quantitative analysis of brain bioluminescence intensity in tumor-free versus GBM-bearing mice at 12 h post-administration of various formulations, n = 3 biologically independent replicates. **c** Quantification analysis of the percentage of total bioluminescence signal attributed to major organs (brain, heart, liver, spleen, lung, kidney), n = 3 biologically independent replicates. Data are presented as mean ± SD, statistical analysis was performed by one-way ANOVA with Fisher's LSD post-hoc test.

• Lipid nanoparticles also accumulate in livers that is populated with many $CD206^+$ Kupffer cells. Are these cells affected by treatment?

Response:

We sincerely thank you for this insightful question, which prompted us to carefully examine the interaction between our lipid nanoparticles (LNPs) and liver-resident $CD206^+$ Kupffer cells, as well as the potential impact of our treatment on these cells. To address this point, we first injected L@ mBiME-EGFP into mice (Figure R3a). To further determine whether Kupffer cells were affected by the treatment, we analyzed EGFP expression and phenotypic changes in this population. The results showed that approximately 10% of Kupffer cells were EGFP-positive following treatment, yet no significant change in the overall proportion of Kupffer cells within the immune cell population was observed (Figure R3a–e). Moreover, the expression of CD206, CD86, and CD163, as well as the phagocytic capacity of Kupffer cells, remained unchanged after treatment (Figure R3f–m).

These observations are likely attributable to the unique biology of Kupffer cells, which

are developmentally distinct, tissue-resident macrophages with a stable, liver-imprinted transcriptional and functional program. Unlike BMDMs or tumor-associated macrophages, Kupffer cells exhibit limited plasticity in classical M1/M2 markers, and binding of RP182 to CD206 on Kupffer cells is insufficient to drive functional reprogramming. Consequently, while PL@mBiME effectively reprograms tumor-associated macrophages, it does not measurably alter Kupffer cell phenotype or function.

The corresponding description has been incorporated into revised manuscript (**lines 467-477**), and the results have been included as **Supplementary Fig. 36**.

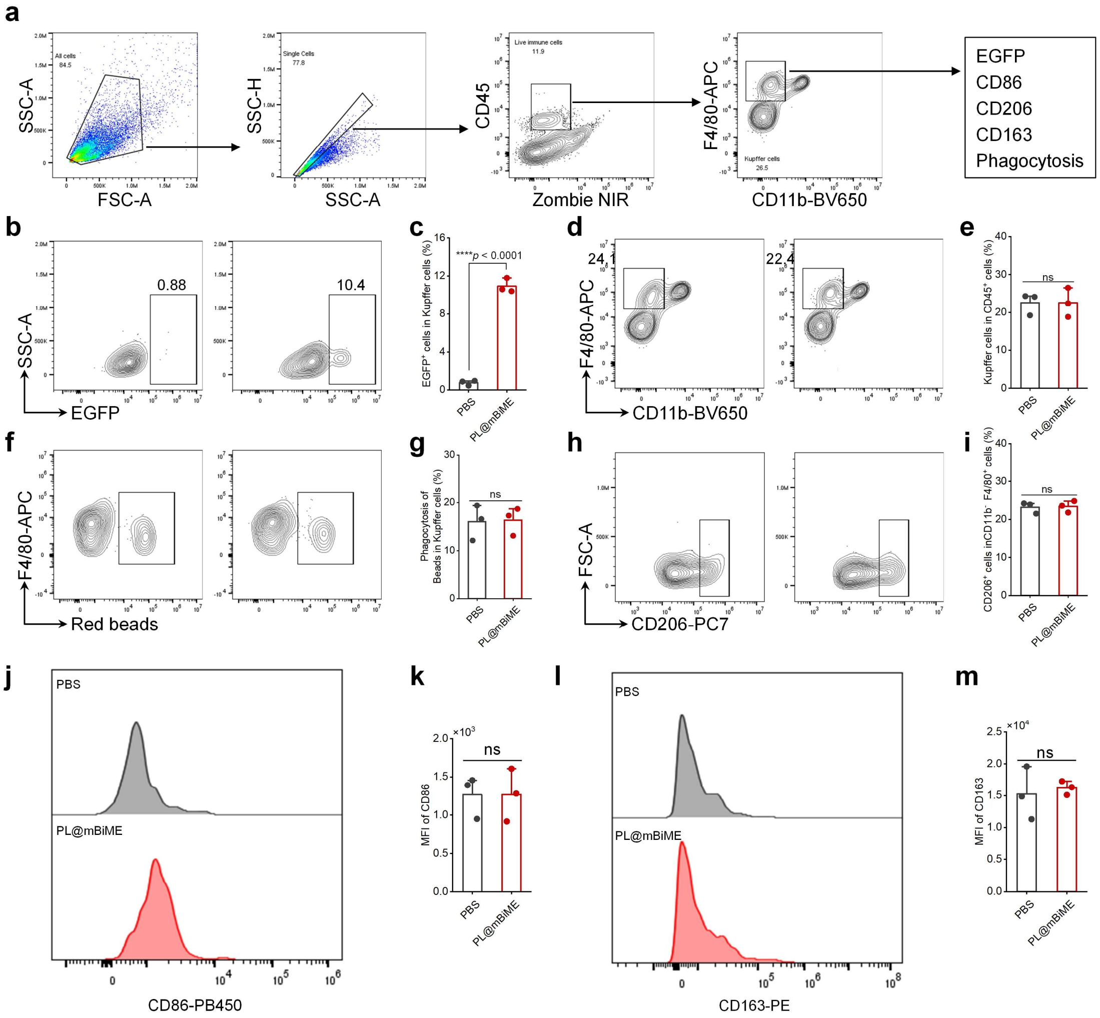

Figure R3. **Phenotypic and functional modulation of Kupffer cells by PL@mBiME *in vivo*.** **a** Gating strategy for liver Kupffer cell analysis. **b** Dot plots of EGFP expression in Kupffer cells post PL@mBiME-T2A-EGFP treatment. **c** Quantification of $EGFP^{+}$ Kupffer cell percentage (from b). **d** Dot plots showing Kupffer cell proportion among liver leukocytes. **e** Quantification of Kupffer cell percentage (from d). **f** Histogram of phagocytosis (latex beads uptake) by Kupffer cells. **g** Quantification of phagocytic activity (from f). **h** Histogram of CD206 expression on Kupffer cells. **i** Quantification

of CD206$^+$ cell percentage (from h). **j** Histogram of CD86 expression on Kupffer cells. **k** Quantification of CD86 mean fluorescence intensity (MFI, from j). **l** Histogram of CD163 expression on Kupffer cells. **m** Quantification of CD163 median fluorescence intensity (MedFI, from l). Data are presented as mean ± SD, n = 3 biologically independent replicates; unpaired two-tailed t-test.

• Fig 5e shows survival of orthotopic glioblastoma-bearing mice. What is the criterium of survival? M&M states that none of the mice reached maximum tumor size of 100 mm3, so on what criterium is survival based?

Response:

We apologize for the confusion and thank the reviewer for raising this important methodological point. In this study, survival was defined as the time from orthotopic tumor inoculation until natural death of the animals. For the orthotopic glioblastoma model shown in Fig. 5d-e, tumors were monitored by MRI to confirm successful tumor establishment and intracranial growth; however, survival was not determined by reaching a predefined tumor volume threshold. Instead, death was used as the primary survival endpoint. All animal experiments were conducted in accordance with approved protocols, which strictly require humane endpoints—including a maximal tumor burden not exceeding 100 mm^3 in brain. In our study, natural mortality occurred before any animal reached this volume threshold, thus all deaths were counted as spontaneous endpoints.

To avoid confusion, we have removed the description of a maximum tumor size from the M&M section and clarified that survival in the orthotopic model is based solely on mouse death. The corresponding description has been incorporated into revised manuscript (**lines 807-809**)

• Fig 6. What is the frequency and number of total F4/80$^+$ cells of the immune infiltrate of the tumors?

Response:

We analyzed the proportion of F4/80$^+$ cells among CD45$^+$ cells within the tumor immune infiltrate by flow cytometry. The results indicated that the macrophage ratio remained consistently around 18%, with no significant difference observed across all experimental groups. Additionally, quantification of IF staining revealed that the infiltration density of F4/80$^+$ cells was approximately 1500 cells/mm^2 across all groups (Figure R4).

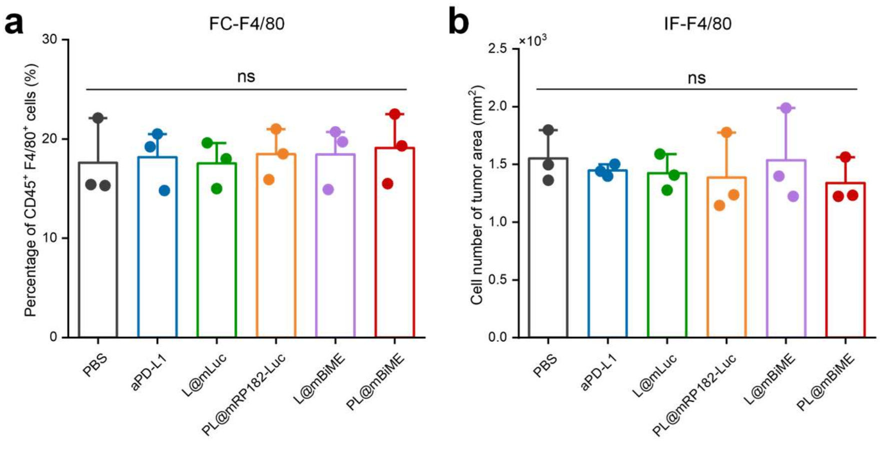

Figure R4 Quantification of tumor-infiltrating F4/80$^+$ macrophages following various treatments. a Flow cytometric analysis of the percentage of F4/80$^+$ macrophages within the CD45$^+$ immune cell infiltrate of tumors. Mice were treated three times with PBS, free aPD-L1, L@mLuc, PL@mRP182-Luc, L@mBiME, or PL@mBiME as indicated. **b** Immunofluorescence (IF) analysis of F4/80$^+$ cells in tumor sections. Representative images (Supplementary Fig. 40) were quantified using ImageJ software to determine the number of F4/80$^+$ cells per field of view. Data are presented as mean ± SD, n = 3 biologically independent replicates; one-way ANOVA with Fisher's LSD post-hoc test.

• Glioblastoma-bearing brain vasculature is different from that of healthy brains. So the lipid nanoparticles might cross the blood-brain barrier independent of the angiopep-2 peptide decoration. The additional benefit of the angiopep-2 peptide might be tested in in tumor-free brains.

Response:

We sincerely appreciate your insightful comment regarding the insufficiency of comparison groups and fully agree that direct comparison between nanoparticles with and without Angiopep-2 (A2) is essential to convincingly demonstrate the role of A2 in blood-brain barrier (BBB) transport. Notably, this concern has also been raised by another reviewer, which has further emphasized the critical importance of supplementing such comparative data to strengthen the rigor of our study.

To address this key issue comprehensively, we have performed additional in vivo experiments to monitor and compare the bio-luminescence signals of A2-conjugated PL@mLuc and non-conjugated PL@mLuc in both tumor-free brains and glioblastoma (GBM)-bearing brains. Moreover, we have extended the monitoring duration from the original time window to 144 h to capture the long-term BBB transport efficiency and intratumoral retention characteristics of the two LNP formulations.

The results clearly demonstrate that A2-conjugated PL@mLuc exhibit significantly

higher bio-luminescence signals in both tumor-free brain tissues and GBM lesions compared with non-conjugated controls at all time points tested, with the most prominent difference observed at 12 h post-injection (Figure R5a-b). The ex-vivo tissue imaging also confirm this (Figure R5c-e) This finding directly confirms that the A2 peptide mediates efficient BBB crossing and enhances the accumulation of LNPs in brain tissues, especially in the GBM microenvironment.

The data has been updated in the revised manuscript **Figure 4f-j** and the corresponding text description is added in manuscript (**lines 402-413**).

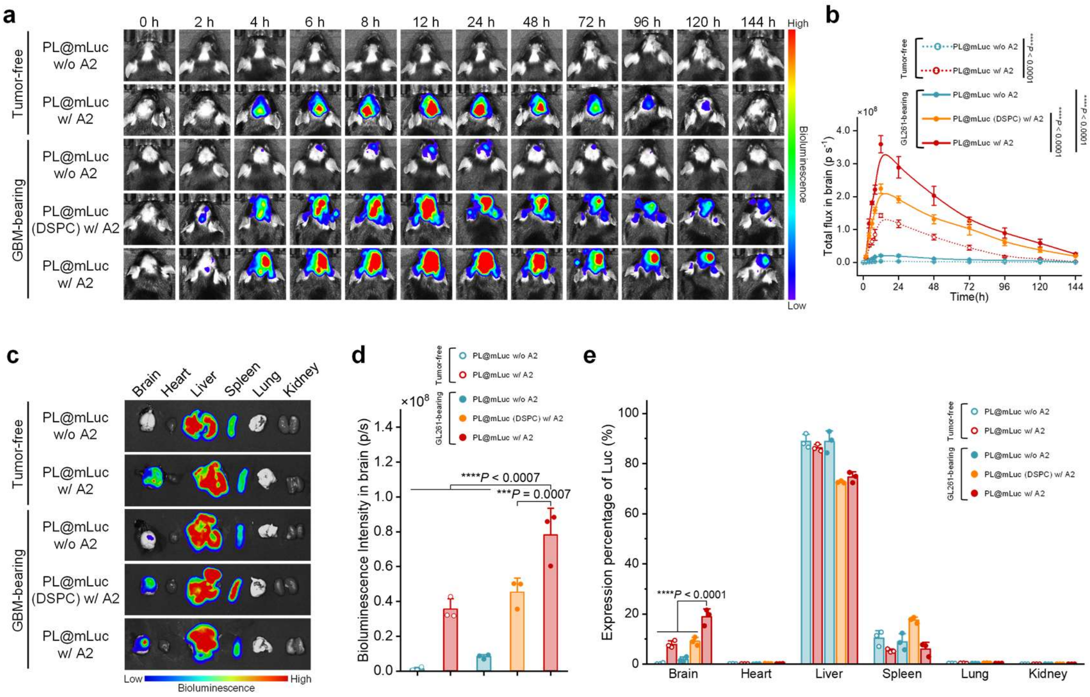

Figure R5 a, b Bioluminescence imaging of brain in tumor-free and GBM-bearing mice at 0, 2, 4, 6, 8, 12, 24, 48, 72 h, 96 h, 120h, and 144 h post-administration of various formulations, n = 3 biologically independent replicates. **c** Representative bioluminescence imaging of isolated brains from tumor-free versus GBM-bearing mice at 12 h post-administration of various formulations. **d** Quantitative analysis of brain bioluminescence intensity in tumor-free versus GBM-bearing mice at 12 h post-administration of various formulations, n = 3 biologically independent replicates. **e** Quantification analysis of the percentage of total bioluminescence signal attributed to major organs (brain, heart, liver, spleen, lung, kidney), n = 3 biologically independent replicates. Data are presented as mean ± SD, statistical analysis was performed by one-way ANOVA with Fisher's LSD post-hoc test.

• The impact of the nanoparticles on the immune infiltrate is well performed, but does not reveal the ultimate effector population responsible for tumor control. Authors may perform subset depletion studies, removing immune cell populations (e.g. macrophages,

CD8 T cells, etc).

Response：

We sincerely thank you for the excellent suggestion to perform subset depletion studies to identify the key effector populations responsible for the anti-tumor efficacy of PL@mBiME. We agree that mechanistic validation of the immune populations involved is crucial.

In response to your recommendation, we conducted depletion experiments using anti-CD8 antibodies to deplete $CD8^+$ T cells and clodronate liposomes to deplete macrophages (Figure R6a-h). The results showed that depletion of $CD8^+$ T cells only mildly attenuated the tumor inhibition effects of PL@mBiME, suggesting that $CD8^+$ T cells partially contribute to the overall response (Figure R6i-k). More strikingly, macrophage depletion significantly abrogated the anti-tumor effects of PL@mBiME (Figure R6i-k), providing direct functional evidence that macrophages are one of the principal effector cells in mediating the anti-tumor response. Overall, these results demonstrate that PL@mBiME activates both innate and adaptive immune responses to suppress tumor growth.

We have integrated these important depletion studies results into the revised manuscript, in **Fig 7k-m and Supplementary Fig. 52a-h** and the corresponding text in **line 596-603** in the Results section.

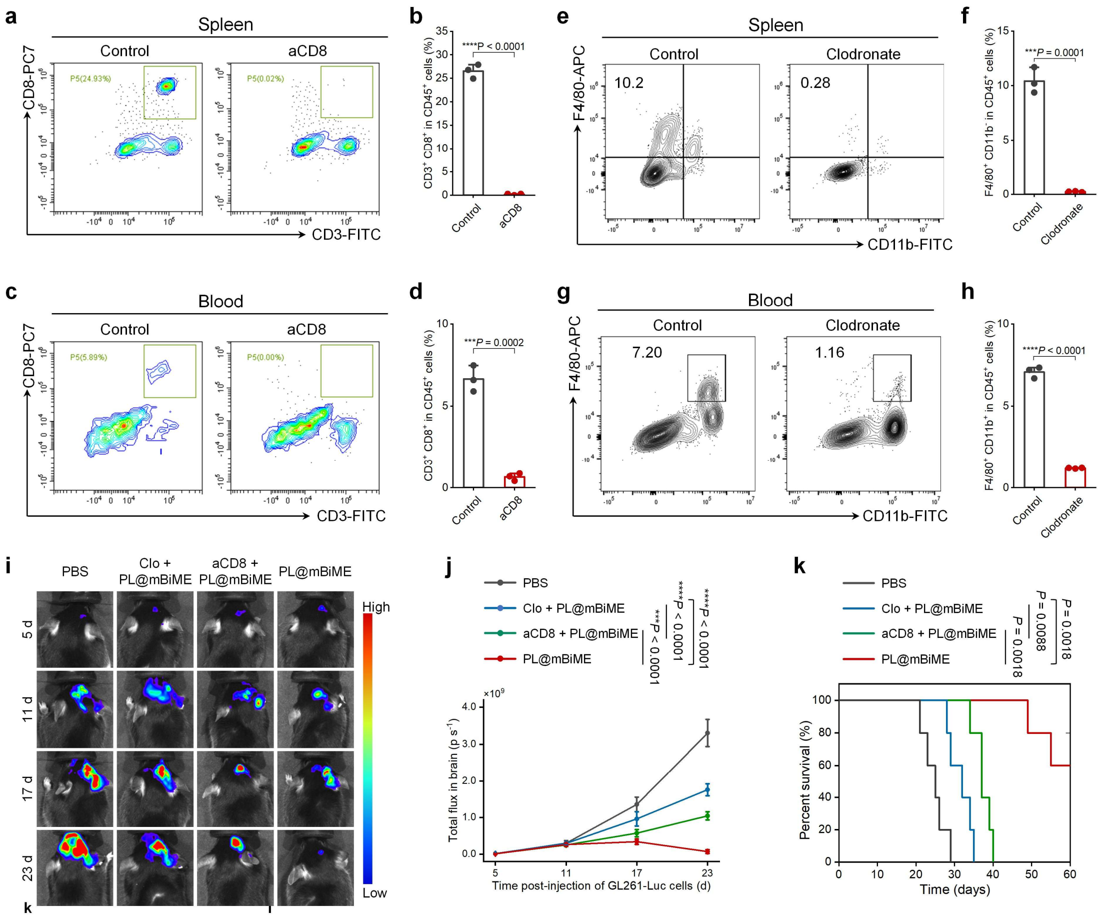

Figure R6. **a-d** Validation of $CD8^+$ T cell depletion. Representative flow cytometry dot plots (a, c) and corresponding quantification (b, d) show the frequency of $CD8^+$ T cells among $CD45^+$ leukocytes in the spleen (a, b) and peripheral blood (c, d) of mice treated with anti-CD8α depleting antibody. **e-h** Validation of macrophage depletion. Representative plots (e, g) and quantification (f, h) show the frequency of $F4/80^+$ macrophages among $CD45^+$ leukocytes in the spleen (e, f) and blood (g, h) of mice treated with clodronate liposomes. Data in b, d, f, h are presented as mean ± SD, n = 3 biologically independent replicates. Statistical significance was determined by unpaired t-test (b, d, f, h). **i, j** Serial *in vivo* bioluminescence images (**i**) and quantification of tumor flux (**j**) show tumor growth in GL261 tumor-bearing mice treated with PL@mBiME following depletion of the indicated immune cell populations, n = 3 biologically independent replicates. **k** Kaplan-Meier survival curves of the corresponding treatment groups, n = 5 biologically independent replicates. Data are presented as mean ± SD, statistical analysis was performed by one-way ANOVA with Fisher's LSD post-hoc test for j. Survival analysis (k) was compared using the log-rank test.

• The discussion section lacks comparison with recently published literature on tyrosine kinase inhibitors to M-CSF receptor that have been successfully applied in glioblastomas.

Response:

Thank you for raising this important point. We agree that comparing our macrophage-engaging strategy with existing pharmacological approaches targeting similar pathways would significantly strengthen the discussion.

We added the following discussion in the revised manuscript in **line 705-712**:

"Unlike CSF1R tyrosine kinase inhibitors which primarily deplete immunosuppressive TAMs, our mRNA-based bispecific macrophage engager (BiME) aims to reprogram and engage TAMs to directly phagocytose tumor cells and initiate adaptive anti-tumor immunity. This approach may circumvent issues like compensatory mechanisms or loss of antigen-presenting function associated with depletion strategies[1, 2, 3]. Furthermore, local BiME expression via nanoparticle delivery ensures high intratumoral concentration and sustained activity, potentially overcoming pharmacokinetic limitations seen with systemic CSF1R inhibition."

This comparative analysis, supported by relevant recent literature, now provides a clearer contextualization of our work within the current therapeutic landscape and better underscores the complementary rationale and potential advantages of our strategy. We thank you again for this valuable suggestion.

• Row 204 contains a typo ‘helpher’.

Response：

We apologize for the oversight and thank you for your careful attention to detail. The term has been corrected to "helper" in the revised manuscript in **line 233**.

We appreciate your meticulous review, which has helped us improve the accuracy of the text.

Reviewer #2 (Remarks to the Author):

This manuscript reports the development of an acid-responsive lipid nanoparticle (PL@mBiME) for co-delivery of bispecific macrophage engager mRNA and PD-L1 antibody in glioblastoma therapy. The study is rigorous, well executed, and addresses critical barriers such as BBB penetration and TAM-driven immunosuppression. Notably, the work introduces a macrophage engager, an area far less explored than T-cell engagers, demonstrates the efficiency of in vivo engager expression via mRNA to bypass complex protein production, and employs a novel CD206-targeting peptide. These advances, together with the elegant nanoparticle design, position this study as an original and impactful contribution to mRNA-based immunotherapy.

Response:
Thank you for the positive feedback. We have carefully considered your suggestions below and have prepared the following responses and planned revisions to further strengthen the manuscript as recommended.
We believe that addressing these points will further enhance the clarity, depth, and impact of the manuscript. Thank you again for your time and valuable guidance.

I have a few suggestions that could further strengthen the manuscript:
1. While PL@mBiME is well characterized and shows impressive therapeutic outcomes, it would be valuable to directly compare its delivery efficacy and biodistribution with conventional DSPC-based LNPs in vivo. Such data would better highlight the translational advantage of this acid-responsive LNP design over existing platforms.

Response:
We sincerely thank you for this excellent suggestion to provide a direct comparative benchmark. We agree that demonstrating the advantage of our pH-responsive LNP design over conventional platforms is essential for highlighting its translational potential.
In response to the reviewer's recommendation, we performed a head-to-head comparison in GBM-bearing mice, directly evaluating the delivery efficacy and biodistribution of our DCPA-based PL@mBiME against a conventional LNP formulated with DSPC. The results showed that luciferase-mRNA expression delivered by the DCPA-based LNP was approximately 1.7-fold higher than that achieved by the DSPC-based LNP in the brain tumor, but not in other organs (Figure R7a-e). This

finding further supports that the incorporation of DCPA strengthens the acid-responsive properties of the LNP, enhancing target gene expression specifically in the tumor area.

These new comparative data were included in revised manuscript **Figure 4f-j**, with corresponding text added in **line 402-413**.

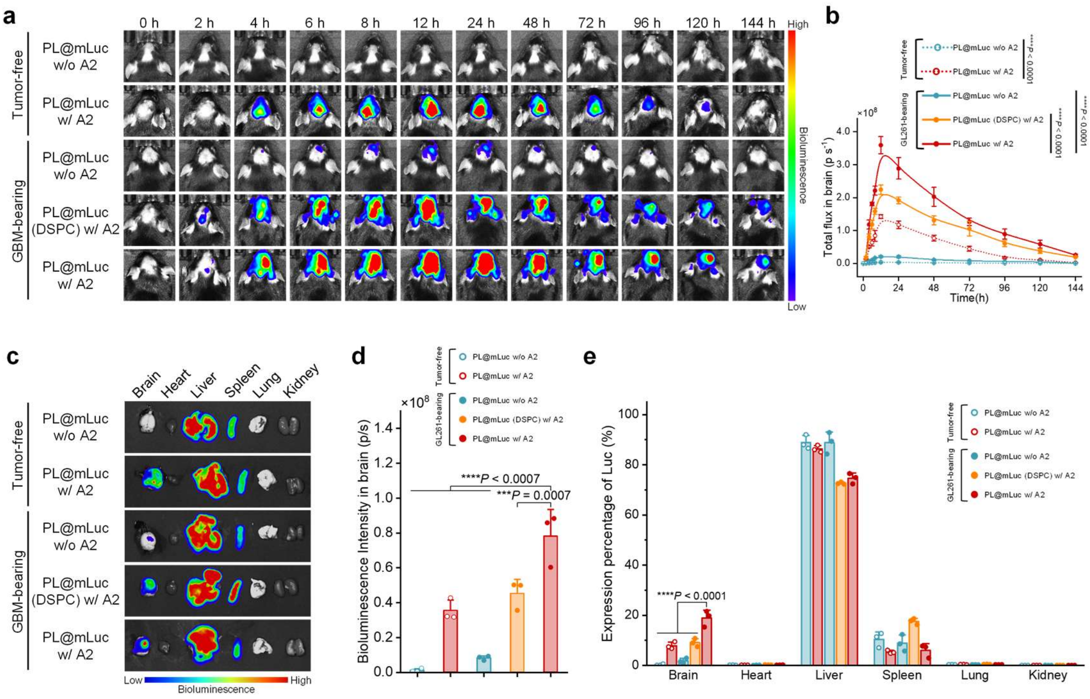

Figure R7. a, b Bioluminescence imaging of brain in tumor-free and GBM-bearing mice at 0, 2, 4, 6, 8, 12, 24, 48, 72 h, 96 h, 120h, and 144 h post-administration of various formulations, n = 3 biologically independent replicates. **c** Representative bioluminescence imaging of isolated brains from tumor-free versus GBM-bearing mice at 12 h post-administration of various formulations. **d** Quantitative analysis of brain bioluminescence intensity in tumor-free versus GBM-bearing mice at 12 h post-administration of various formulations, n = 3 biologically independent replicates. **e** Quantification analysis of the percentage of total bioluminescence signal attributed to major organs (brain, heart, liver, spleen, lung, kidney), n = 3 biologically independent replicates. Data are presented as mean ± SD, statistical analysis was performed by one-way ANOVA with Fisher's LSD post-hoc test.

2. In Figure 4j, luciferase signals are still present at 72 hours, suggesting sustained protein expression. Since persistence of mRNA-derived protein expression is an important parameter for translational application, it would be interesting to examine how long the protein expression lasts in the brain and whether this correlates with

therapeutic benefit.

Response:

Thank you for raising this important translational point regarding the persistence of protein expression. We agree that understanding the duration of in vivo protein expression is crucial for evaluating the therapeutic potential and dosing regimen of mRNA-based therapies.

In response to your suggestion, we extended the longitudinal in vivo bioluminescence imaging time course to explicitly examine how long luciferase protein expression persists in the brains of tumor-bearing mice following a single administration of PL@mBiME (carrying luciferase mRNA). The results clearly showed that protein expression persists for up to 144 hours (6 days) post-injection, with a peak observed around 12 hours (Figure R8a-b).

These updated data have been incorporated into **Figure 4f-g** of revised manuscript.

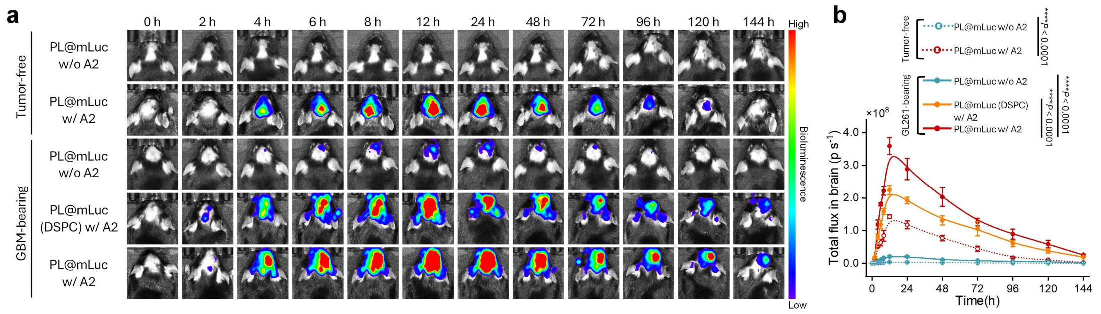

Figure R8. a, b Bioluminescence imaging of brain in tumor-free and GBM-bearing mice at 0, 2, 4, 6, 8, 12, 24, 48, 72 h, 96 h, 120h, and 144 h post-administration of various formulations, n = 3 biologically independent replicates. Data are presented as mean ± SD, statistical analysis was performed by one-way ANOVA with Fisher's LSD post-hoc test.

3. The in vivo data (Figure 6l–n) and enhanced macrophage engulfment (Figure 3g) indirectly support that BiME connects tumor cells with macrophages. To provide more direct validation, I recommend including an in vitro co-culture experiment of tumor cells and macrophages, assessing whether the frequency of physical macrophage–tumor cell conjugates increases upon BiME expression.

Response:

Thank you for raising this crucial point. We agree that a direct, visual demonstration of the "bridging" function provided by BiME is important to complement our functional evidence.

In response to your recommendation, we performed the suggested in vitro co-culture assay to directly validate the BiME-mediated interaction. The details are as follows:

First, we measured the surface ErbB2 expression on CT-2A-Luc (another GBM model),

GL261-Luc, and GL261-OE-Luc (exogenously overexpressing ErbB2) via flow cytometry. The results showed that GL261-Luc cells (47.8%) exhibit higher ErbB2 expression compared to CT-2A-Luc cells (17.8%), while GL261-OE-Luc cells (93.1%) exhibited the highest ErbB2 expression (Figure R9a).

Next, we conducted a co-culture assay using Bone Marrow-Derived Macrophages (BMDMs) and CT-2A-Luc, GL261-Luc, or GL261-OE-Luc cells, and treated the cultures with PBS, PL@mRP182-Luc, or PL@BiME. By systematically quantifying the frequency of macrophage-tumor cell physical conjugates using fluorescence microscopy, we found that, compared to PBS, PL@BiME significantly increased the formation of physical "bridges" between macrophages and tumor cells in all cell groups. Furthermore, adding the ErbB2 antibody sequence to the mRNA construct further promoted these conjugates compared to PL@mRP182-Luc. This effect was correlated with ErbB2 expression, with the highest conjugate formation observed in the GL261-OE-Luc cells, which exhibited the highest ErbB2 expression in the PL@BiME treatmaent group but not in the PL@mRP182-Luc or PBS treatment group. This directly links enhanced target engagement to increased conjugate formation, explaining the observed boost in phagocytic activity (Figure R9b-c).

These data confirm that BiME expression enhances the physical interaction between tumor cells and macrophages, with RP-182 binding to CD206 on macrophages and the ErbB2 antibody targeting ErbB2 on cancer cells.

These new findings have been added to **Fig. 3e-g and Supplementary Fig. 24** in the revised manuscript, and the corresponding text has been updated in **line 285-300** of the Results section.

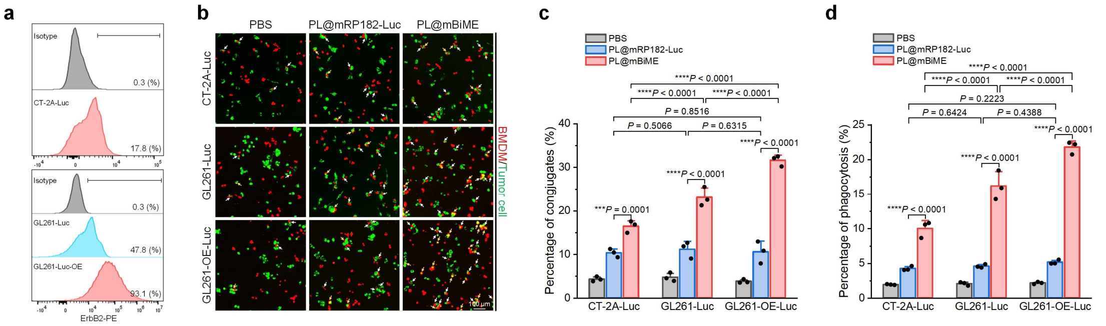

Figure R9. a Representative flow cytometry histograms showing ErbB2 expression in CT-2A-Luc, GL261-Luc, and GL261-OE-Luc cell lines. **b-d** Representative fluorescence microscopy images and quantification of conjugates and phagocytosis between tumor cells (DiO, green) and BMDMs (DiI, red). White arrows indicate conjugate formation between BMDMs and tumor cells, while yellow arrows denote

phagocytosis of tumor cells by BMDMs. Scale bar = 100 μm, n = 3 biologically independent replicates. Data are presented as mean ± SD, statistical analysis was performed by two-way ANOVA with Fisher's LSD post-hoc test.

4. Since BiME bridges macrophages and tumor cells via CD206 and HER2, it would be informative to test whether phagocytosis efficiency depends on HER2 expression levels. Specifically, assessing BiME-mediated macrophage activity against HER2^high versus HER2^low tumor cells could clarify the selectivity and potential scope of this strategy.

Response:

Thank you for this insightful suggestion. Investigating the correlation between target antigen expression and therapeutic response is crucial for defining the selectivity and applicability of our bispecific engager strategy.

As mentioned in response to the previous comment, we have demonstrated that the physical bridging between cancer cells and macrophages induced by BiME depends on ErbB2 expression. Following your recommendation, we next directly examined whether BiME-mediated phagocytic activity depends on HER2 (ErbB2) expression levels on tumor cells.

To address this, we performed an in vitro co-culture phagocytosis assay using BMDMs and CT-2A-Luc, GL261-Luc, or GL261-OE-Luc cells, and treated them with either a control mRNA construct (PL@mRP182-Luc) or PL@mBiME. The results were striking. Treatment with PL@mRP182-Luc showed no significant difference in phagocytic efficiency across the three tumor cell lines, regardless of their ErbB2 expression. In contrast, treatment with PL@mBiME resulted in a strong, ErbB2-dependent enhancement of phagocytosis. Specifically, the phagocytic efficiency for GL261-Luc and GL261-OE-Luc cells was 1.45-fold and 1.98-fold higher, respectively, compared to that for CT-2A-Luc cells (Figure R10).

These data provide direct and quantitative evidence that the efficacy of BiME-mediated macrophage phagocytosis is critically dependent on the expression level of the target antigen (ErbB2) on tumor cells. This confirms the selectivity of our approach and clarifies its potential scope: tumors with higher ErbB2 expression are likely to respond more robustly.

These new data have been incorporated into the revised manuscript **Fig. 3g and Supplementary Fig. 24**, and the result description has been added in **line 285-300.**

We thank you again for prompting this important analysis, which significantly strengthens the mechanistic understanding and translational rationale of our study.

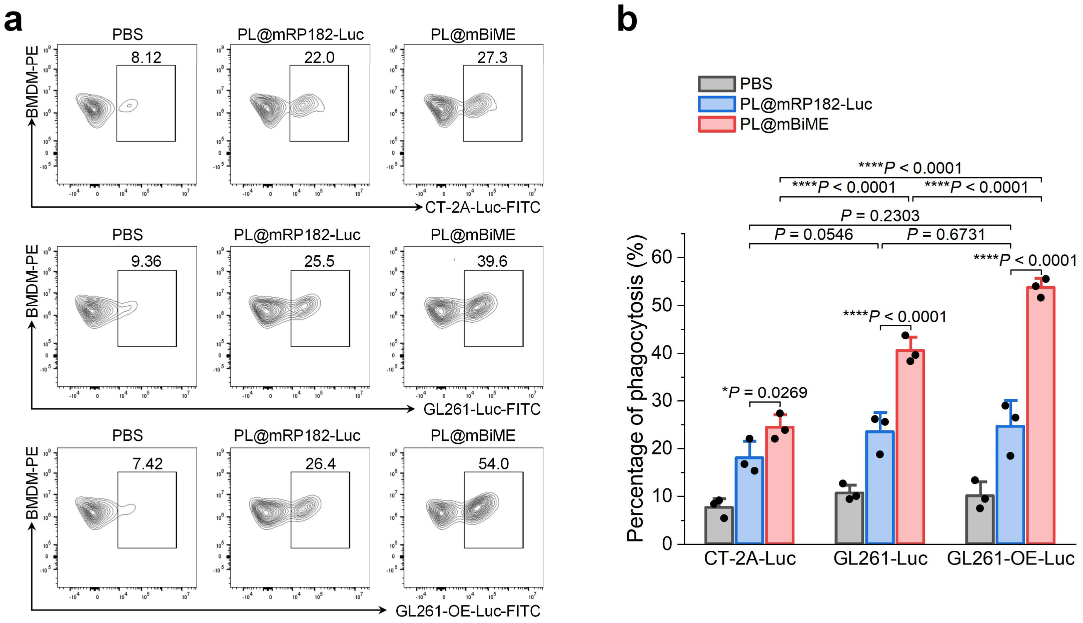

Figure R10. a Flow cytometry plots showing the phagocytic activity of BMDMs after co-incubation with CT2A-Luc, GL261-Luc, or GL261-OE-Luc cells under different treatment conditions (PBS, PL@mRP182-Luc, or PL@mBiME). **b** Quantification of phagocytic activity based on the flow cytometry plots shown in (a), n = 3 biologically independent replicates. Data are presented as mean ± SD, statistical analysis was performed by two-way ANOVA with Fisher's LSD post-hoc test.

Reviewer #3 (Remarks to the Author):

This study presents a multifaceted approach to overcoming the challenges of treating solid tumors like glioblastoma. The PL@mBiME combines several strategies into a single system. By shifting the focus from T-cells to engaging macrophages with a bispecific mRNA construct, the authors circumvented critical barriers such as poor T-cell infiltration and systemic toxicity. The dual targeting of ErbB2 on glioma cells and CD206 on M2 macrophages is a promising strategy that not only directs the immune attack but also reprograms the immunosuppressive tumor microenvironment, enhancing phagocytosis and antigen presentation. Furthermore, the incorporation of pH-responsive charge reversal for improved tumor accumulation and lysosomal escape ensures efficient mRNA expression. The addition of a glutathione-triggered, surface-conjugated aPD-L1 antibody provides a localized and potent immune checkpoint blockade to counteract suppression and sustain anti-tumor immunity. This work offers a therapeutic strategy that could improve outcomes for patients with GBM and potentially other solid malignancies. However, following issues should be addressed.

Response:
Thank you for this insightful and constructive summary of our study's core strategy and potential. Regarding the points raised for further consideration, we have prepared detailed responses and performed experiments below. We believe addressing these will significantly strengthen the manuscript.

1. In Figure 1j, what is the in vivo GSH concentration? Does it significantly exceed 10 mM? When is the PD-L1 antibody released—before or after cellular uptake?

Response:
We sincerely thank the reviewer for raising these insightful questions, which help clarify how PL@mBiME responds to the tumor microenvironment (TME). The in vivo glutathione (GSH) concentration in the TME was quantified using a commercial GSH assay kit. The results show that the GSH concentration in the TME is approximately 4.75 mM (Figure R11a-b).
Based on this observation, we further evaluated PD-L1 antibody release in vitro under both 4 mM and 10 mM GSH conditions. The results showed that aPD-L1 was gradually released in the presence of GSH, reaching a plateau of approximately 50% and 75% release at 12 hours under 4 mM and 10 mM GSH, respectively. In contrast, negligible release was observed in the absence of GSH (Figure R11c).
To further investigate the timing of PD-L1 antibody release, we labeled aPD-L1 with FITC and mBiME with Cy5 dye. We then co-incubated PL@mBiME with GL261 cells

in medium containing either 0 mM or 4 mM GSH. Confocal microscopy images revealed that, under the 0 mM GSH condition, the PD-L1 antibody co-localized with mBiME inside the cells. In contrast, under the 4 mM GSH condition, most of the PD-L1 antibody was released extracellularly and targeted PD-L1 expression on cell membrane of GL261 cells, with minimal co-localization observed with Cy5-mBiME. (Figure R11d-e). This result indicates that PD-L1 antibody undergoes extracellular release in response to GSH in the tumor microenvironment prior to cellular uptake.
These data have been incorporated into the revised manuscript in **Figure 1m and Supplementary Figure 8 and 9**, with the corresponding text updated in **line 176-192**.

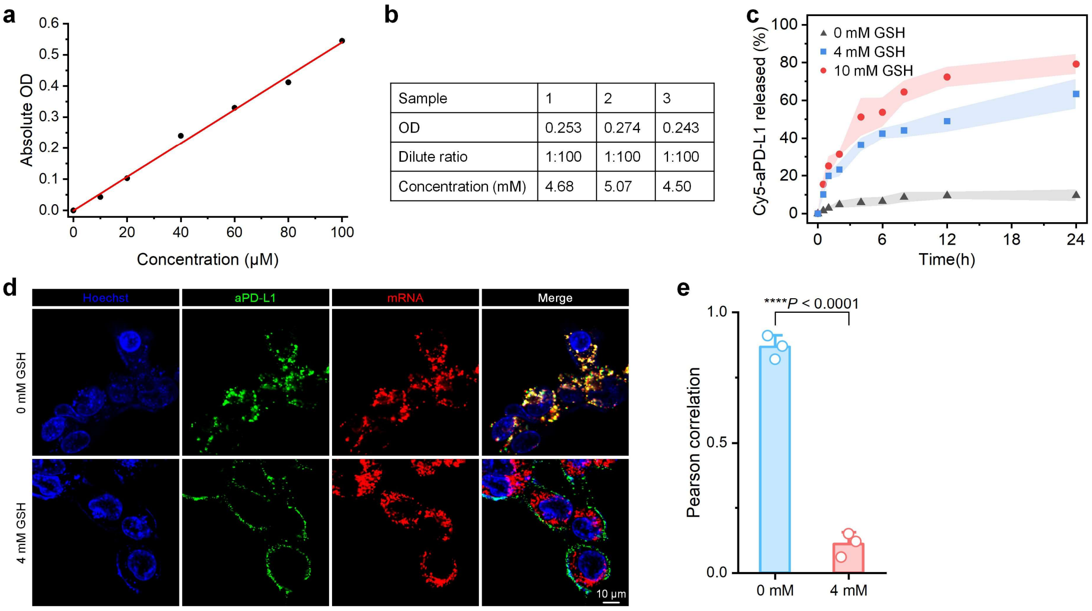

Sample	1	2	3
OD	0.253	0.274	0.243
Dilute ratio	1:100	1:100	1:100
Concentration (mM)	4.68	5.07	4.50

Figure R11.a Standard curve for the GSH assay kit. Serial dilutions of a known GSH standard were used to generate the curve for calculating sample concentrations. **b** Quantified GSH concentrations in brain with tumor, as determined using the standard curve from panel (a), n = 3 independent replicates. **c** Responsive release of the Cy5-aPD-L1 in 0, 4, or 10 mM GSH (n = 3 independent replicates). **d** Intracellular distribution of FITC-labeled aPD-L1 (green) and Cy5-labeled mBiME (red) in GL261 cells after 4 h, with 0 mM or 4 mM GSH. Scale bar = 25 μm. **e** Pearson's correlation analysis confirms GSH-responsive carrier disassembly (reduced co-localization) at high GSH. Data are presented as mean ± SD, n = 3 biologically independent replicates; unpaired two-tailed Student's t-test.

2. "Furthermore, enzyme-linked immunosorbent assay (ELISA) analysis revealed that treatment with BiME significantly upregulated the secretion of pro-inflammatory cytokines IL-1β, TNF-α, and IFN-γ, while simultaneously downregulating the anti-inflammatory cytokine IL-10 (Figure 3j-l). " There are no IFN- γ data presented here.

Additionally, based on the sequence of the results description, the IL-10 data should be placed at the end rather than in the middle.

Response:

Thank you for this careful and accurate observation regarding the cytokine data presentation in organically manuscript Figure 3j-l. We sincerely apologize for the oversight and any confusion caused.

1. **Regarding the order of IL-10 data:** We have revised the manuscript text to reorder the description of the cytokine results. The sentence now reads: "Furthermore, enzyme-linked immunosorbent assay (ELISA) analysis revealed that treatment with BiME significantly upregulated the secretion of pro-inflammatory cytokines IL-1β and TNF-α, while simultaneously downregulating the anti-inflammatory cytokine IL-10 (**Fig 3h-j**)." in revised manuscript **line 301-305**. The IL-10 data was placed at the end of the figure (Figure R12a-c). Upon reviewing the manuscript, we also discovered that the same issue regarding the order of IL-10 data presented in **Supplementary Fig. 43**. We have accordingly corrected the sequence there as well (Figure R12d).
2. **Regarding the IFN-γ data:** We apologize for the error. We have carefully reviewed the data, we only measured IFN-γ in vivo (**Supplementary Fig. 43**) but not in vitro . Therefore, we have corrected this by removing the IFN-γ from this sentence.

Thank you again for your meticulous review, which has helped us improve the precision of our data presentation.

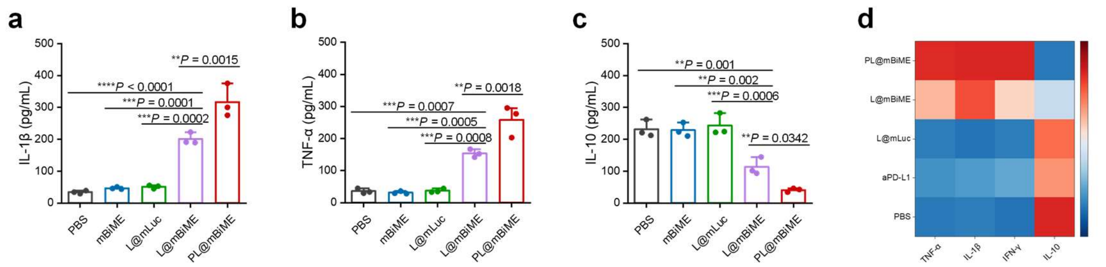

Figure R12. **a-c** Changes in the secretion of IL-1β, TNF-α and IL-10 after treatment with different nano formulations, n = 3 biologically independent replicates. **d** Pro-inflammatory factor (TNF-α, IL-1β, IFN-γ) and anti-inflammatory factor (IL-10) levels and in tumor tissues treated with PBS, aPD-L1, L@mLuc, L@mBiME and PL@mBiME for three times, respectively, n = 3 biologically independent replicates. Data are presented as mean ± SD, statistical analysis was performed by one-way ANOVA with Fisher's LSD post-hoc test.

3. The authors found that PL@mBiME enhances the physical interaction between macrophages and tumor cells and increases phagocytic activity. While this represents a

major innovative aspect of the study, the experimental evidence provided is insufficient. Further in vitro and in vivo evidence is required to substantiate this conclusion.

Response:

We thank the reviewer for this important comment and fully agree that rigorous in vitro and in vivo validation is essential to substantiate the conclusion that PL@mBiME enhances macrophage–tumor cell interactions and phagocytic activity. In response, we have performed additional direct and quantitative in vitro and in vivo experiments that provide mechanistic evidence supporting this key innovation.

First, to establish a system with graded target antigen expression, we quantified surface ErbB2 levels on three glioblastoma models—CT-2A-Luc, GL261-Luc, and GL261-OE-Luc (exogenous overexpressing Erbb2)—by flow cytometry. The results showed that GL261-Luc cells (47.8%) express higher ErbB2 levels than CT-2A-Luc cells (17.8%), while GL261-OE-Luc cells exhibit the highest ErbB2 expression (93.1%) (Figure R13a).

Using these models, we next performed an in vitro co-culture assay with Bone Marrow-Derived Macrophages (BMDMs) to directly visualize and quantify BiME-mediated physical interactions. Compared to PBS treatment, PL@BiME significantly increased the formation of physical macrophage–tumor cell conjugates across all tumor cell lines. Importantly, PL@BiME, which incorporated the ErbB2 antibody sequence in the mRNA construct, exhibited enhanced conjugate formation compared to PL@mRP182-Luc. This effect was ErbB2-dependent, as higher ErbB2 expression was associated with increased macrophage–tumor cell "bridge" formation only in the PL@BiME-treated group, whereas no such correlation with ErbB2 expression was observed in the PL@mRP182-Luc–treated or PBS treated group. These results provide direct visual evidence that BiME promotes physical bridging between macrophages and tumor cells via dual engagement of CD206 and ErbB2 (Figure R13b-c).

To further determine whether this enhanced physical interaction translates into functional activity, we conducted quantitative phagocytosis assays using the same co-culture system. While PL@mRP182-Luc treatment resulted in comparable phagocytic efficiency across all tumor cell lines regardless of ErbB2 expression, PL@mBiME induced a strong, ErbB2-dependent enhancement of macrophage phagocytosis. Specifically, phagocytic efficiency increased by 1.45-fold and 1.98-fold for GL261 and GL261-OE-Luc cells, respectively, compared to CT-2A-Luc cells (Figure R13d-e). These data demonstrate that BiME-mediated macrophage activity is critically dependent on tumor antigen expression level.

2. Expanded In Vivo Evidence:

To determine the role of ErbB2 in mediating the *in vivo* activity of PL@mBiME, we

established orthotopic tumors using GL261-Luc and GL261-OE-Luc cells and treated mice with PBS, PL@mRP182-Luc, or PL@mBiME. Consistent with the oncogenic function of ErbB2, GL261-OE-Luc tumors exhibited significantly accelerated growth compared to GL261-Luc tumors in both the PBS and PL@mRP182-Luc treatment groups (Figure R13f-g). In contrast, PL@mBiME treatment resulted in pronounced tumor growth inhibition and significantly prolonged survival in both GL261-Luc and GL261-OE-Luc models, with a greater degree of tumor suppression observed in the GL261-OE-Luc model and a higher proportion of tumor-free mice achieved in this group (Figure R13f-h).

Representative tumors from CT-2A-Luc, GL261-Luc, and GL261-OE-Luc models were analyzed by immunofluorescence. Markedly increased colocalization of ErbB2 (green) and the macrophage marker F4/80 (red) was observed in PL@mBiME-treated tumors across all three models, indicating enhanced macrophage－tumor cell bridging events compared to both PBS- and PL@mRP182-Luc-treated groups. Notably, the extent of macrophage－tumor cell conjugate formation increased with tumor cell ErbB2 expression, progressing from CT-2A-Luc (low) to GL261-Luc (intermediate) and GL261-OE-Luc (high) (Figure R13i-j). Together, these findings demonstrate that PL@mBiME promotes macrophage-tumor cell physical interactions in an ErbB2-dependent manner in vivo, with higher tumor ErbB2 expression associated with enhanced therapeutic efficacy.

Together, these new experiments provide direct, quantitative, and mechanistic evidence that PL@mBiME enhances macrophage–tumor cell physical interactions and selectively amplifies phagocytic activity in an ErbB2-dependent manner. These findings substantiate our central conclusion and define the mechanistic basis and selectivity of this bispecific engager strategy.

All new data have been incorporated into the revised manuscript (**Fig. 3e-g, Fig. 6e-i and Supplementary Fig. 24**), with corresponding text added in **line 285-300** and **Line 530-548** of the Results section.

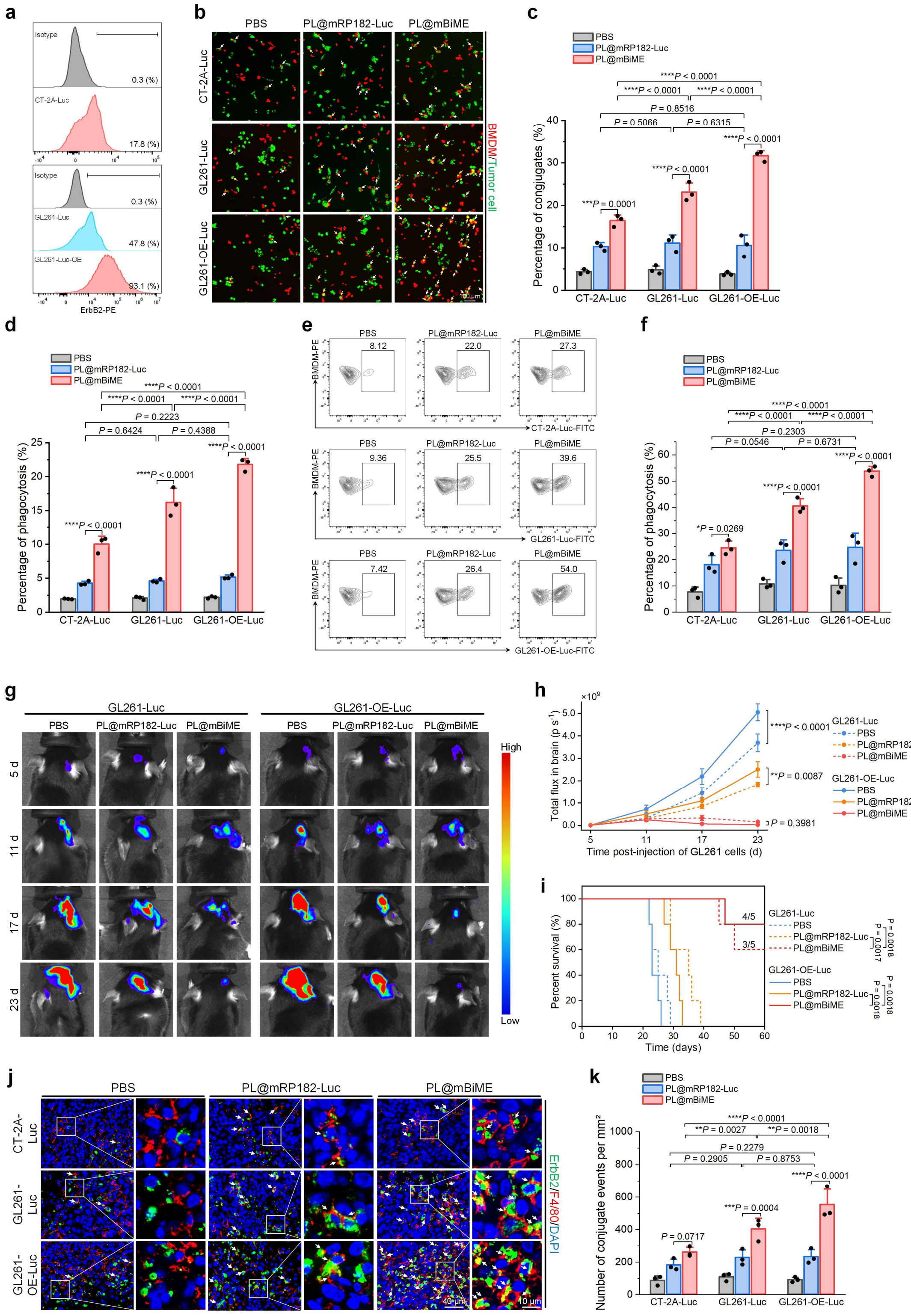

Figure R13. a Representative flow cytometry histograms showing ErbB2 expression in CT-2A-Luc, GL261-Luc, and GL261-OE-Luc cell lines. **b-d** Representative fluorescence microscopy images and quantification of conjugates and phagocytosis between tumor cells (DiO, green) and BMDMs (DiI, red). White arrows indicate conjugate formation between BMDMs and tumor cells, while yellow arrows denote

phagocytosis of tumor cells by BMDMs. Scale bar = 100 μm, n = 3 biologically independent replicates. **e** Flow cytometry plots showing the phagocytic activity of BMDMs after co-incubation with tumor cells under different treatment conditions (PBS, PL@mRP182-Luc, or PL@mBiME). **f** Quantification of phagocytic activity based on the flow cytometry plots shown in (e), n = 3 biologically independent replicates. **g, h** Representative IVIS images and quantified signal intensities of GL261-Luc and GL261-OE-Luc tumor-bearing mice treated with PBS, PL@mRP182-Luc and PL@mBiME, n = 3 biologically independent replicates. **i** Survival curves of GL261-Luc and GL261-OE-Luc tumor-bearing mice following treatment with different formulations, n = 5 biologically independent replicates. **j, k** Representative immunofluorescence (IF) images and statistical analysis of macrophage-tumor cell conjugation (green: ErbB2, red: F4/80, white arrows indicate macrophage-tumor cell conjugates). Scale bar = 50 μm, 20μm, n = 3 biologically independent replicates. Data are presented as mean ± SD, statistical analysis was performed by two-way ANOVA with Fisher's LSD post-hoc test for c, d, f, h and k. Survival analysis (i) was compared using the log-rank test.

4. In Fiugre 5， the effects of PL@mBiME on the levels of CD8+ T cells and Granzyme B should be determined by IHC.

Response:

Thank you for this constructive suggestion. We agree that quantifying tumor-infiltrating $CD8^+$ T cells and Granzyme B levels via immunohistochemistry (IHC) would provide valuable spatial and histological context to complement our flow cytometry data.

Following your recommendation, we have performed additional IHC analyses on tumor sections from the relevant treatment groups. The results, now included in the revised manuscript **Supplementary Fig. 51** (**line 591-595**), which clearly demonstrate that treatment with PL@mBiME leads to a significant increase in the infiltration of $CD8^+$ T cells and an elevated expression of Granzyme B within the tumor microenvironment, compared to control groups (Figure R14). This histological evidence directly and visually supports our conclusion that PL@mBiME increased cytotoxic T cell number and activity in tumor, thereby reinforcing the adaptive anti-tumor immune response induced by our therapy.

Thank you again for the suggestion, which has strengthened the evidential support for the immunomodulatory effects of PL@mBiME.

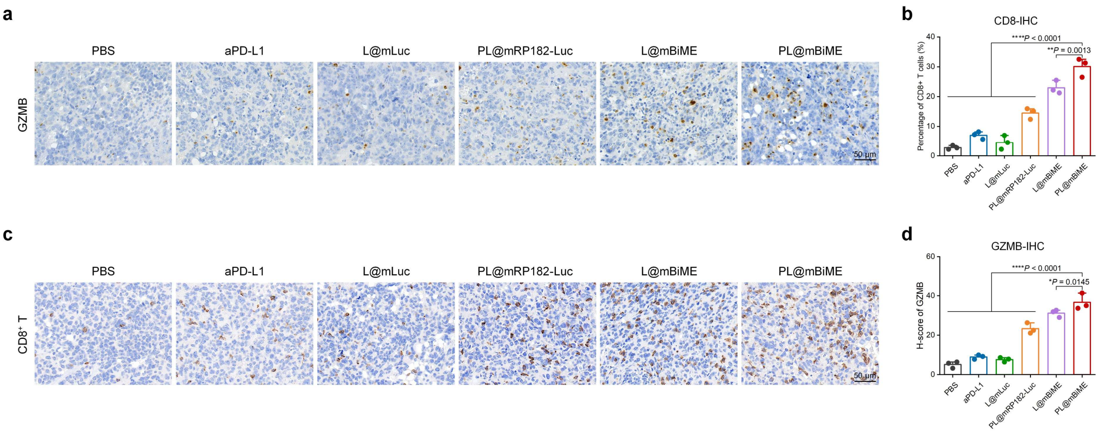

Figure R14. Quantitative immunohistochemical profiling of cytotoxic immune response in treated tumors. a Representative immunohistochemical (IHC) staining for $CD8^+$ cytotoxic T lymphocytes within tumor sections from mice receiving the indicated treatments. **b** Quantification of $CD8^+$ T cell positivity rate (percentage of $CD8^+$ cells among total nucleated cells) from images in panel (a). **c** Representative IHC staining for the cytolytic effector protein Granzyme B (GZMB). **d** Quantification of GZMB expression level using the H-Score (Histochemical Score) method, which integrates both the intensity and proportion of positive staining, from images in panel (c). Scale bars are indicated in the images. Quantitative data (b, d) are presented as mean ± SD, n = 3 biologically independent replicates. Data are presented as mean ± SD, statistical analysis was performed by one-way ANOVA with Fisher's LSD post-hoc test.

5. A single GBM model is insufficient. It is necessary to validate the efficacy of PL@mBiME in at least one additional model. Since PL@mBiME is more effective under acidic pH conditions, it would be interesting to examine its efficacy in glycolytic tumors compared to non-glycolytic tumors, or in control versus LDHA-knockout tumors.

Response:

Thank you for this insightful and constructive suggestion. We agree that validating the therapeutic efficacy in an additional GBM model is crucial, and we appreciate your specific proposal to explore the pH-dependent mechanism in relation to tumor glycolysis.

1. Validation in an Additional GBM Model:

In direct response to your primary concern regarding model robustness, we have conducted a comprehensive efficacy study using the syngeneic CT-2A luciferase-expressing GBM model (CT-2A-Luc). Both L@mBiME and PL@mBiME demonstrated significant anti-tumor effects, with PL@mBiME treatment resulting in

the most potent tumor suppression (Figure R15a-c). However, in contrast to the GL261-Luc model, no tumors in the PL@mBiME treatment group reached completed regression in the CT-2A-Luc model. This is likely due to the lower expression of ErbB2 on CT-2A-Luc cells compared to GL261 cells-Luc (Figure R13a).

2. Investigation of pH-Dependent Efficacy in a Glycolysis-Modulated Model:

PL@mBiME shows preferential accumulation in acidic environments; however, whether tumor acidity is critical for its anti-tumor efficacy remained unclear. To investigate this, we knocked out the Ldha1 gene in GL261-Luc cells, which catalyzes the conversion of pyruvate to lactate during glycolysis (Figure R15). Ldha1 knockout significantly increased the intratumoral pH and concomitantly reduced BiME expression within the tumor area (Figure R15d-f). As a result, the tumor-suppressive efficacy of PL@mBiME was also attenuated in Ldha1-knockout tumors (Figure R15g-i).

These data indicate that the anti-tumor efficacy of PL@mBiME is not limited to a specific model, but rather depends on the expression of ErbB2. Furthermore, these findings provide compelling in vivo evidence linking the therapeutic superiority of PL@mBiME to its pH-responsive design and the acidic tumor microenvironment. We sincerely thank you for this excellent suggestion, which has significantly enhanced our mechanistic understanding of the nanoplatform

These new experiments have been incorporated into the revised manuscript (**Supplementary Fig. 28 and 29**) with corresponding text added in **Line 439-455**.

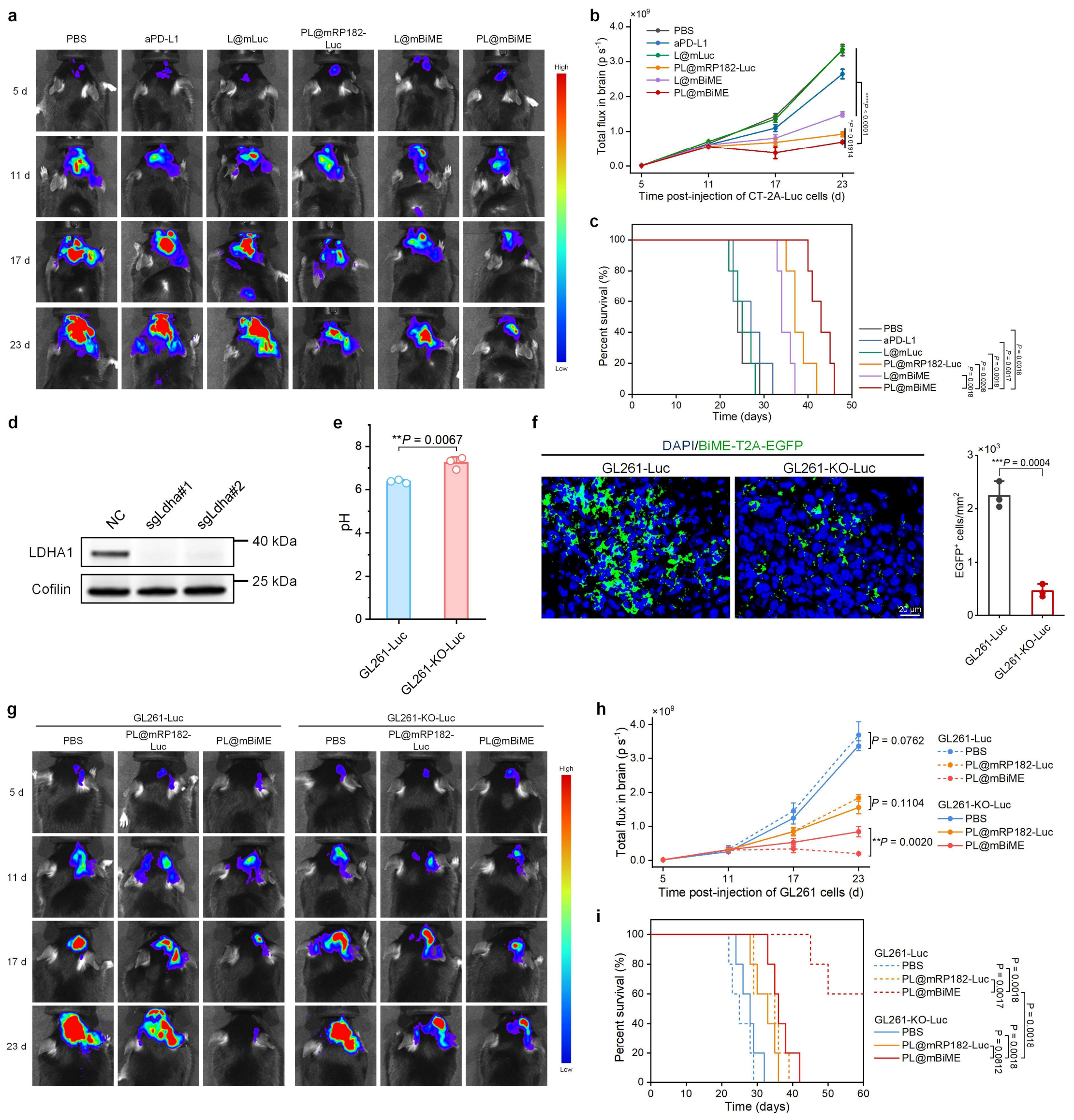

Figure R15. **a, b** Representative IVIS images and quantified signal intensities of CT-2A-Luc tumor-bearing mice treated with PBS, aPD-L1, L@mLuc, PL@mRP182-Luc, L@mBiME, and PL@mBiME, n = 3 biologically independent replicates. **c** Survival curves of CT-2A-Luc tumor-bearing mice following treatment with different formulations, n = 5 biologically independent replicates. Data are presented as mean ± SD, statistical analysis was performed by one-way ANOVA with Fisher's LSD post-hoc test for b. Survival analysis (c) was compared using the log-rank test. **d** Western Blot verification of LDHA Knockout efficiency in GL261 cells (GL261- KO-Luc). **e** Measurement of intratumoral acidity in GL261-Luc and GL261-KO-Luc brain tissues, n = 3 biologically independent replicates. **f** Representative images and quantification of BiME-T2A-EGFP expression in GL261-Luc and GL261-KO-Luc brain tissues, n = 3 biologically independent replicates. **g, h** Representative IVIS images and quantified signal intensities of GL261-Luc and GL261-KO-Luc tumor-bearing

mice treated with PBS, PL@mRP182-Luc and PL@mBiME, n = 3 biologically independent replicates. **i** Survival curves of GL261-Luc and GL261-OE-Luc tumor-bearing mice following treatment with different formulations, n = 5 biologically independent replicates. Data are presented as mean ± SD, statistical analysis was performed by one-way ANOVA with Fisher's LSD post-hoc test for e, f and h. Survival analysis (i) was compared using the log-rank test.

6. What is the mechanism for the durable immune memory generated by PL@mBiME in GBM?

Response:

Thank you for this insightful question regarding the mechanism underlying the durable immune memory induced by PL@mBiME. We propose a multi-step mechanism that leverages the central role of macrophages in initiating and sustaining adaptive anti-tumor immunity:

The establishment of long-term, protective anti-tumor immunity represents a central goal of immunotherapy, particularly for cancers like glioblastoma (GBM) characterized by a highly immunosuppressive microenvironment. Our PL@mBiME nanoparticle platform is designed to overcome this barrier by orchestrating a sequential, multi-component immune response that culminates in durable immunological memory. The proposed mechanism unfolds in three critical and synergistic phases: initiation, facilitation, and consolidation.

1) The process is initiated at the level of innate immunity. PL@mBiME delivers mRNA encoding a bispecific macrophage engager (BiME) directly into the tumor milieu. The mRNA is translated in situ, producing BiME proteins that simultaneously bind to CD206 on immunosuppressive, M2-like tumor-associated macrophages (TAMs) and to ErbB2 on glioma cells. This dual binding serves two key functions: it physically bridges the macrophage to the tumor cell, facilitating direct cytotoxic engagement and phagocytosis, and it triggers an intracellular signaling cascade that repolarizes the macrophage toward a pro-inflammatory, antigen-presenting M1-like phenotype, with increased expression of CD86, a co-stimulatory molecule essential for T cell activation (Figure R16a-b). This phenotypic switch is critical. Reactivated macrophages enhance their phagocytic ability and efficiently process engulfed tumor-associated antigens (TAAs) and tumor-specific antigens (TSAs) for presentation to T cells (Figure R16 c-d). Consequently, they transform into potent, tumor-localized antigen-presenting cells (APCs), capable of cross-presenting TAAs to prime naïve $CD8^+$ cytotoxic T cells and activating $CD4^+$ helper T cells—the essential first step in generating a de novo adaptive immune response against the tumor.

2) Second, the supportive phase ensures the survival and functionality of newly primed T cells. PL@mBiME co-encapsulates and enable glutathione-responsive release of an anti-PD-L1 antibody (aPD-L1) within the same tumor niche. This localized checkpoint blockade disrupts the PD-1/PD-L1 inhibitory axis, a dominant pathway of T-cell exhaustion in solid tumors. By preventing the early functional impairment or deletion of tumor-specific T cells, this sustained local inhibition of this pathway creates a permissive window that allows the primed T-cell clones to undergo robust expansion and differentiate into effector cells, thereby amplifying the adaptive immune attack.

3) The final, the memory phase ensures the longevity of the response. The T-cell activation and proliferation, supported by the pro-inflammatory cytokines from M1 macrophages and the relief from checkpoint-mediated inhibition, drives a subset of the effector T cells to differentiate into long-lived memory T cells. These cells provide systemic surveillance and rapid recall capability. Our in vivo rechallenge experiments, wherein cured mice completely resisted a secondary tumor inoculation, provide direct functional validation of this established, systemic immune memory (Figure R16e-m).

In summary, PL@mBiME generates durable immune memory by transforming TAMs into effective APCs that cross-prime and activate tumor-specific T cells, while simultaneously employing localized checkpoint blockade to prevent T-cell exhaustion. This coordinated strategy ensures the initiation, expansion, and long-term maintenance of a protective adaptive immune response against GBM.

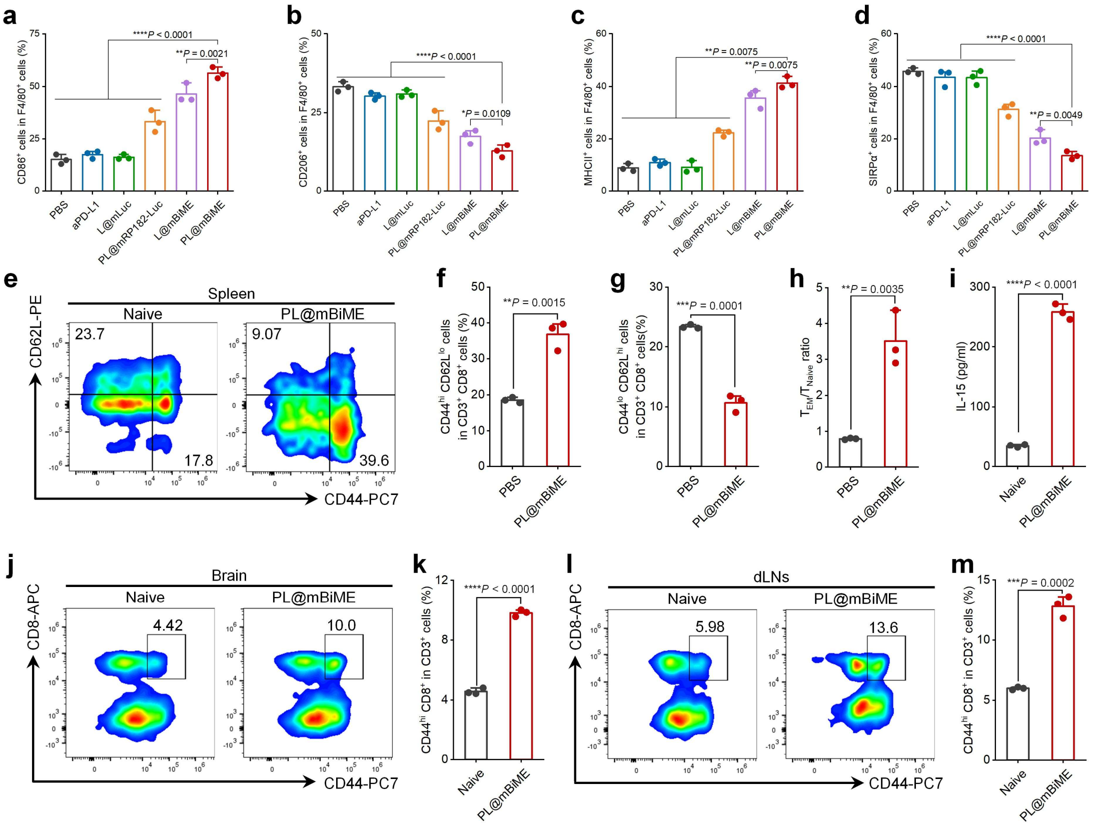

Figure R16. **a, b** Quantification of tumor-infiltrating TAM polarization (M1 marker CD86, M2 marker CD206) cells three days after three treatments with PBS, aPD-L1, L@mLuc, PL@mRP182-Luc, L@mBiME, and PL@mBiME, n = 3 biologically independent replicates. **c, d** Representative flow cytometric images of SIRPα^{+} in TAMs following treatment with different formulations, n = 3 biologically independent replicates. Data are presented as mean ± SD, statistical analysis was performed by one-way ANOVA with Fisher's LSD post-hoc test for a-d. **e-g** Representative flow cytometry smoothed dot plots of effector memory T cells (T_{EM}) with CD44^{hi} expression and low CD62L^{lo} expression and naive T cells (T_{Naive}) with CD44^{lo} expression and CD62L^{hi} expression in the spleens of naïve mice and mice treated with PL@mBiME, n = 3 biologically independent replicates. **h** Quantitative analysis of the ratio of T_{EM} to T_{Naive} in the spleens of mice after different treatments by flow cytometry, n = 3 biologically independent replicates. **i** The cytokine levels of interleukin-15 (IL-15) in tumor tissues after different treatments, n = 3 biologically independent replicates. **j-m** Representative flow cytometry smoothed dot plots and quantitative analysis of CD8^{+}CD44^{+} memory T cells in the brains and draining lymph nodes (dLNs) of naive mice and mice treated with PL@mBiME, n = 3 biologically independent replicates. Data are presented as mean ± SD, statistical analysis was performed by unpaired two-tailed Student's t-test for f-i, k and m. Survival analysis (m) was compared using the log-rank test.

7. As PL@mBiME targets GBM in the brain, its potential effects on cognitive function should be evaluated in mouse models.

Response:

Thank you for this valuable comment. We fully agree with your suggestion that evaluating the potential effects of PL@mBiME on cognitive function is crucial for its potential clinical translation, especially given its targeting of glioblastoma (GBM) in the brain—where therapeutic interventions may inadvertently impact central nervous system (CNS) function.

In response to your comment, we evaluated the potential impact of PL@mBiME on higher-order brain function, a critical consideration for any intracranial therapy. Cognitive function, particularly spatial learning and memory which is highly dependent on hippocampal integrity, was assessed using the Morris water maze test in GL261 tumor-bearing mice. Notably, to minimize confounding effects of tumor burden on cognitive performance, a tumor-bearing control group with tumor sizes comparable to those of the PL@mBiME-treated group, but receiving no treatment, was included.

Compared to the tumor-bearing control group, PL@mBiME-treated mice exhibited similar escape latency during training (Figure R17b), comparable time spent in the target quadrant during the probe trial (Figure R17c), and an equivalent number of platform crossings (Figure R17d). In contrast, mice in the PBS and other treatment groups showed significantly impaired performance. These differences are likely attributable to the reduced tumor burden achieved by PL@mBiME treatment relative to the other treatment groups.

These results demonstrate that the potent anti-tumor immunity elicited by PL@mBiME does not come at the cost of cognitive impairment. The preservation of complex neural functions underscores the neurological safety of our approach and suggests that the localized immune activation and tumor clearance do not induce widespread damage to cognition-critical neural circuits.

The data were added in revised manuscripts (**Supplementary Fig. 37**), the corresponding text was added in **Line 478-496**.

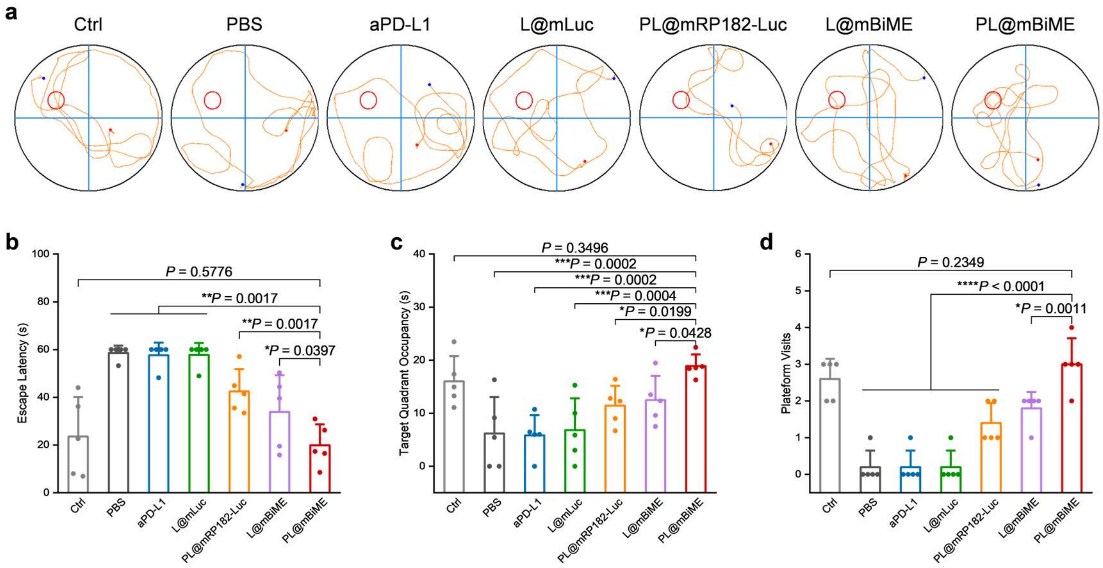

Figure R17. Assessment of murine cognitive function via behavioral testing. a Representative Morris water maze trajectories treated with various formulations. **b-d** Statistical analysis of escape latency, target quadrant occupancy, and platform visits in various formulation groups, n = 5 biologically independent replicates. Data are presented as mean ± SD, statistical analysis was performed by one-way ANOVA with Fisher's LSD post-hoc test.

Reviewer #4 (Remarks to the Author):

The authors report a new and highly complex nanoparticle platform for targeted delivery to the brain to treat glioblastoma. The authors make several claims about their nano delivery system, which features 1) peptide targeting to cross the blood-brain barrier, 2) a glutathione cleavable linker to release anti-PDL1 extracellularly, 3) an acidity-sensitive lipid that supports lysosomal escape and delivery of mRNA cargo into tumor and non-tumor cells. The mRNA cargo encodes a bispecific macrophage engager targeting ErbB2 on tumor cells and CD206 on macrophages.

The study is timely and addresses an unmet need in cancer using a new nanoparticle platform, however characterization of this platform is not adequate to support the authors' claims. The in vivo data showing efficacy of their system is impressive and encouraging; however, there are major concerns that may confound the interpretation of these results and subsequently diminishes the impact of this work.

Response:

We sincerely thank the reviewer for the thoughtful and constructive assessment of our work, as well as for recognizing the timeliness and potential impact of our nanoparticle platform for glioblastoma treatment. We fully agree that a rigorous and comprehensive characterization of such a multifunctional delivery system is essential to substantiate our mechanistic claims and to ensure the correct interpretation of the therapeutic outcomes.

In direct response to the overarching concern regarding the adequacy of characterization, we have undertaken a comprehensive series of supplementary in vitro and in vivo experiments. We believe these additions strengthen the mechanistic foundation of the study, ensuring that the observed in vivo efficacy can be confidently attributed to the intended functionalities of our delivery system.

Thank you again for this constructive and valuable feedback, which has been instrumental in enhancing the rigor and impact of our work.

Major Concerns

1. The nanoparticle system is exceptionally complex, and requires more robust characterization. The authors are encouraged to review the 2018 paper in Nature Nanotechnology "Minimum information reporting in bio-nano experimental literature," PMID: 30190620IF: 34.9 Q1. Given the complexity of this system, Figure 1 is not adequate. The encapsulation of efficiency of mRNA is addressed, but characterization of A2 and aPDL1 are lacking. The conjugation conditions are not provided, nor any assays to show that the peptide and antibody are stably covalently conjugated. Similarly, multiple assays use modifications of the platform, with

fluorescent labels, reporter mRNA, and variations on their mBiME. These modifications can alter the structure and function of lipid nanoparticles, and it was challenging to follow exactly which LNP variation was being tested throughout the paper. The methods do not report how fluorescent dyes were conjugated to lipids or antibodies or show whether these conjugation strategies impacted nanoparticle size, charge, encapsulation, etc.

Response:

We sincerely thank you for your insightful and constructive comments. Your feedback has been invaluable in helping us improve the rigor and completeness of our nanoparticle characterization. We fully recognize the complexity of the PL@mBiME lipid nanoparticle (LNP) system, and we agree that the initial characterization (Figure 1) and method descriptions were insufficient for fully validating the system's integrity. Following your suggestion, we have reviewed the 2018 paper from Nature Nanotechnology ("Minimum Information Reporting in Bio-Nano Experimental Literature," PMID: 30190620) and have performed additional experiments to address the gaps you identified. Below are our detailed responses to the specific concerns you raised:

1. **Characterization of A2 and aPD-L1 Conjugation**

Comment: "The encapsulation efficiency of mRNA is addressed, but characterization of A2 and aPD-L1 is lacking. The conjugation conditions are not provided, nor any assays to show that the peptide and antibody are stably covalently conjugated."

We greatly appreciate your comment regarding the incomplete characterization of DSPE-PEG-A2 and the lack of quantitative data on ligand valency (A2 and aPD-L1 molecules per nanoparticle). In response, we have performed and included the following comprehensive characterization data in the revised manuscript

Conjugation Conditions: Detailed conjugation conditions for A2 peptide and aPD-L1 antibody have been added to the section "Synthesis of Blood-Brain Barrier Penetrating Lipid DSPE-PEG-A2" and "Preparation of L@mBiME and PL@mBiME" in the Methods. The full details are available in the supplementary information (**Line 53-114**).

Conjugation of Angiopep-2 to DSPE-PEG-NHS

The targeting conjugate DSPE-PEG-A2 was synthesized by coupling the Angiopep-2 (A2) peptide to the terminal of DSPE-PEG2000-NHS via amine-reactive chemistry. Previously obtained DSPE-PEG2000-NHS (5 mg) was dissolved in 5 mL 0.01 M PBS (pH 7.4). To this solution, 5 mg Angiopep-2 peptide was added. The reaction mixture was stirred at 4 ° C overnight to facilitate the conjugation between the NHS ester group of the lipid and the primary amines of the peptide. Following the conjugation, the reaction was quenched by adding an excess of Tris-HCl buffer (containing free amine groups) and incubating for

1 hour at room temperature to hydrolyze any unreacted NHS esters. The crude mixture was then transferred to a dialysis bag (cut-off 500 Da) and dialyzed extensively against ultrapure water. The dialyzed solution was subsequently concentrated by rotary evaporation and purified by reverse-phase chromatography using a CombiFlash EZ Prep system (ISCO). The separation was monitored in real-time using both an Evaporative Light Scattering Detector (ELSD) and a UV detector. Fractions containing the desired product, DSPE-PEG-A2, were collected based on the characteristic elution profile. Finally, the pooled fractions were lyophilized to obtain DSPE-PEG-A2 as a white, hygroscopic powder.

DSPE-PEG-A2 Incorporation and aPD-L1 Conjugation

Roughly 0.1% M/M of DSPE-PEG2000-A2 mixture was added to the formulated LNPs. The reaction was incubated at 4 °C for 48 h. The post-inserted LNP was then concentrated by an ultrafiltration system and slowly applied to a 90-cm-bed-length gravity-flow size exclusion column prepared with Sepharose-CL4B gel. The mobile phase was PBS. The fractions that contained LNP were collected and concentrated by the ultrafiltration to obtain L@mBiME. To release the encapsulated or surface-conjugated A2 peptide, the nanoparticles were treated with 0.5% (v/v) Triton X-100 in PBS for 30 minutes at 37 ° C. After centrifugation to remove insoluble debris, the supernatant was collected, and the A2 peptide concentration in the lysate was determined using a Fluorometric Peptide Quantitation Assay Kit (Beyotime Biotechnology) according to the manufacturer's protocol.

To construct the final formulation, the obtained L@mBiME was conjugated with an anti-programmed cell death ligand 1 (aPD-L1) antibody via a redox-responsive linker. Specifically, the heterobifunctional crosslinker NPC-SS-NPC (where NPC denotes a nitro phenyl carbonate ester group reactive toward amines, and SS denotes a cleavable disulfide bond) was employed. The crosslinker was added at a molar ratio of 1.2:1 (crosslinker to aPD-L1) to ensure efficient bridging. This mixture was then reacted with L@mBiME at a fixed molar ratio of aPD-L1 to L@mBiME at 0.5%. The conjugation reaction was conducted in PBS buffer (0.01 M PBS, pH 7.4) at 4 ° C for 12 – 16 hours with gentle stirring.

Following the reaction, the crude mixture was purified to remove unreacted crosslinker and antibody. The solution was transferred to a Vivaspin ultrafiltration device (Sartorius) with a molecular weight cut-off (MWCO) of 300 kDa and centrifuged. This process was repeated with multiple cycles of dilution and concentration (diafiltration) using the reaction buffer to thoroughly exchange the medium. The final retentate, containing the purified antibody-conjugated nanoparticles, was collected and designated as

PL@mBiME. For the quantification of conjugated aPD-L1, the PL@mBiME nanoparticles were treated with 0.5% (v/v) Triton X-100 to disrupt the lipid bilayer and release the antibody. The aPD-L1 concentration in the supernatant was quantified using a commercial Rat IgG ELISA Kit (Beyotime Biotechnology) following the standard protocol. This assay measures the total IgG content, which directly corresponds to the amount of aPD-L1 present in the nanoparticle formulation.

Confirmation of DSPE-PEG-A2 Conjugation via MALDI-TOF-MS:

We synthesized DSPE-PEG-A2 through the Michael addition reaction between maleimide-functionalized DSPE-PEG2000 and Angiopep-2. To confirm the successful covalent conjugation, we used MALDI-TOF-MS. The expected molecular weight of DSPE-PEG-A2 is approximately 5300 Da, and MALDI-TOF-MS analysis revealed characteristic peaks in the 4900–5736 Da range, consistent with the theoretical molecular weight of the conjugate (Figure R18a). The minor deviation from the theoretical value is attributed to the polydispersity of the PEG segment.

Detection of PEGylation of aPD-L1 by Western Blot:

We performed Western blot analysis to confirm the PEGylation of aPD-L1. Proteins were probed with an anti-PEG antibody and detected by enhanced chemiluminescence. The anti-PEG antibody showed no signal in native aPD-L1 but clear bands in the reaction mixture and purified product, confirming successful covalent attachment of PEG to aPD-L1 (Figure R18b).

Regarding the A2 peptide and aPD-L1 characterization, we have provided a detailed supplementary explanation and supporting data in response to your later comment: "How many molecules of A2 are present, on average, per molecule. Similarly, how many molecules of aPDL1 are on each nanoparticle?" in the section of 2. Experimental Design & Quantification of minor concern part. Please kindly refer to there for details. These additional data provide evidence for the stable conjugation of A2 peptide and aPD-L1 antibody, the results are included in the revised manuscript (**Supplementary Fig. 4 and 5**).

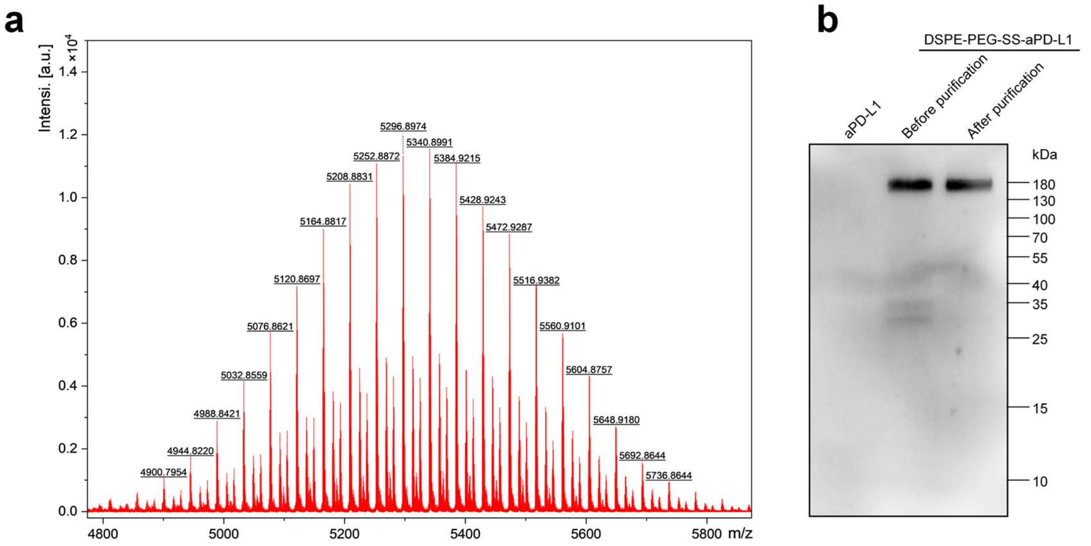

Figure R18. a The matrix assisted laser desorption ionization time of flight mass spectrometry (MALDI-TOF-MS) spectrum of DSPE-PEG-A2. **b** Detection of PEGylation of aPD-L1 by Western blot. Proteins were separated by SDS-PAGE and probed with an anti-PEG antibody (PEG-B-47, abcam) to confirm PEGylation, Lane 1: Native aPD-L1 (non-PEGylated control); Lane 2: Reaction mixture of aPD-L1 and DSPE-PEG-NH_2 conjugated via the NPC-SS-NPC crosslinker before purification; Lane 3: Purified PEGylated aPD-L1.

2. Characterization of LNP Variants

Comment: "Similarly, multiple assays use modifications of the platform, with fluorescent labels, reporter mRNA, and variations on their mBiME. These modifications can alter the structure and function of lipid nanoparticles, and it was challenging to follow exactly which LNP variation was being tested throughout the paper. The methods do not report how fluorescent dyes were conjugated to lipids or antibodies or show whether these conjugation strategies impacted nanoparticle size, charge, encapsulation, etc."

We sincerely thank you for this constructive and important observation. We fully agree that the use of multiple labeled versions of the platform, including fluorescent tags and reporter mRNA, could lead to potential confusion regarding the exact LNP formulations being tested at different stages. We appreciate the opportunity to clarify this and provide the necessary methodological details.

To directly address this point, we have prepared two comprehensive summary tables (Table R1 and Table R2) that list all mRNA and LNP variants used throughout the study. Furthermore, in the revised Methods section, we have now provided detailed protocols for all conjugation strategies. Critically, we also report the full characterization data

(size, zeta potential, encapsulation efficiency) for each key LNP variant both before and after conjugation with dyes or antibodies. This data confirms that our conjugation methods did not significantly alter the fundamental physicochemical properties of the nanoparticles. The tables are included in the revised manuscript (**Supplementary Table 1 and Supplementary Table 3**).

Table R1. Application of reporter mRNA and its variants.

Reporter mRNA and variations	Figure	Application
mBiME	Fig. 3 and Fig. 5-8	Treatment
mBiME-T2A-EGFP	Fig. 2 e-g, Supplementary Fig. 7, Supplementary Fig. 13-16, Supplementary Fig. 26 and Supplementary Fig. 29.	Transfection efficiency and tissue tracing
mRP182-Luc	Fig. 3 e-f, Fig. 6 h-l and Supplementary Fig. 24 and Supplementary Fig. 28-29	The correlation between therapeutic efficacy and ErbB2 expression level
mLuc	Fig. 4 f-j and Supplementary Fig. 26.	In vivo delivery
FITC-mBiME	Fig. 2 a	Cellular uptake
Cy5-mBiME	Fig. 2 b-d, Fig. 4 a-c and Supplementary Fig. 9.	GSH-responsive release, lysosomal escape and in vitro blood-brain barrier penetration

Table R2. Characterization of various nano-formulations.

Nano-formulations	Figure	Size (nm)	Zeta (mV)	Encapsulation (%)
L@mBiME	Fig. 1f,h	76.8 ± 5.8	−8.31 ± 0.34	94.75 ± 6.02
PL@mBiME	Fig.1g,i	86.9 ± 6.3	−8.15 ± 0.82	94.77 ± 2.28
PL@FITC-mBiME	Fig. 2a	85.6 ± 3.3	−8.52 ± 0.74	92.68 ± 3.71
PL@mBiME-T2A-EGFP	Fig. 2 e-g, Supplementary Fig. 13-14	87.9 ± 8.8	−8.01 ± 0.58	92.88 ± 4.78
PL@mBiME-T2A-EGFP (DSPC)	Fig. 2 e-g	86.5 ± 6.6	−11.19 ± 0.47	94.93 ± 3.05
PL@mRP182-Luc	Fig. 3 e	85.6 ± 4.3	−8.48 ± 0.22	93.04 ± 3.76
L@Cy5-mBiME	Fig. 4 a-c	76.3 ± 3.7	−8.82 ± 0.95	95.24 ± 2.14
PL@Cy5-mBiME	Fig. 4 a-c	86.1 ± 2.3	−8.24 ± 0.40	94.69 ± 2.56
Cy7-L@mLuc	Fig. 4 d-e	76.5 ± 6.5	−8.54 ± 0.39	93.90 ± 3.36
Cy7-PL@mLuc	Fig. 4 d-e	86.6 ± 5.4	−8.41 ± 0.56	94.59 ± 3.84
L@mLuc	Supplementary Fig. 26	77.3 ± 7.1	−8.55 ± 0.63	93.12 ± 2.15
PL@mLuc	Supplementary Fig. 26	85.4 ± 3.7	−8.10 ± 0.97	95.61 ± 5.64
PL@mLuc (DSPC)	Fig. 4 f-j	89.5 ± 4.2	−11.3 ± 1.35	93.69 ± 3.96
PL@mLuc w/o A2	Fig. 4 f-j	87.3 ± 4.4	−8.05 ± 0.59	93.92 ± 4.80

Fluorescent Labeling of aPD-L1 Antibody (FITC, Cy5, and Cy7)

The aPD-L1 antibody was fluorescently labeled with FITC, Cy5 or Cy7 using NHS ester chemistry. The following protocol was applied to all dyes with minimal adjustments for their specific molecular weights when calculating molar ratios.

1. Antibody Preparation:

The aPD-L1 antibody was dialyzed into 0.01 M phosphate-buffered saline (PBS, pH 7.4) using a suitable dialysis membrane or desalting column. The antibody concentration was adjusted to 1 mg/mL in the same buffer prior to conjugation.

2. Dye Solution Preparation:

The NHS-ester derivative of the fluorescent dye was dissolved in anhydrous dimethyl sulfoxide (DMSO) to prepare a 10 mM stock solution. The solution was used immediately or aliquoted and stored desiccated at -20° C, protected from light and moisture.

3. Conjugation Reaction:

The dye stock solution was added dropwise to the prepared antibody solution with gentle vortexing or stirring. The reaction was performed at a molar ratio of dye:antibody = 30:1. The reaction mixture was incubated at 4° C overnight with continuous gentle agitation and protection from light.

4. Reaction Quenching:

To quenche any unreacted NHS esters and terminate the reaction, an equal volume of 0.01 M Tris-HCl buffer (pH 7.4) was added. The mixture was incubated for 30 minutes at room temperature in the dark.

5. Purification:

The crude conjugation mixture was transferred to an ultrafiltration device (MWCO: 10 kDa). Free dye and reaction byproducts were removed by performing three cycles of concentration and dilution with ice-cold PBS (pH 7.4). The final retentate was collected.

6. Characterization and Storage:

The purified labeled antibody solution was filtered through a 0.22 μm membrane filter. A small aliquot was taken for characterization.

The final labeled antibody was stored at 4° C in PBS, protected from light.

Fluorescent Labeling of Lipid Nanoparticles (LNPs)

Two distinct fluorescent labeling strategies were employed to enable the tracking of LNPs and their mRNA cargo.

1. Lipid-Labeled LNPs (Cy7 Conjugation):

LNPs bearing a near-infrared fluorescent label on their lipid component were prepared by incorporating DSPE-PEG2000-Cy7 into the standard lipid mixture during formulation. The fluorescent lipid was added at a molar ratio of 0.1% (M/M, relative to total lipid content). All other formulation parameters, including the microfluidic mixing procedure described in the general method, remained unchanged.

2. mRNA-Labeled LNPs (Fluorescent mRNA Cargo):
LNPs encapsulating fluorescently labeled mRNA were formulated using the standard LNP synthesis protocol. The cargo consisted of mRNA that had been pre-labeled using a fluorescently modified nucleotide (FITC-UTP or Cy5-UTP) via in vitro transcription. The labeled mRNA was purified and then used directly in place of unmodified mRNA during the LNP assembly process, with no other alterations to the lipid composition or mixing procedure.

The data were added in revised manuscripts (**Supplementary Table 1 and 3**), the corresponding text was added in Materials and Methods in **Supplementary Information line 140-188**.

2. The choice of tumor model and mBiME target are incongruent and likely confound the interpretation of the study results. While commonly used and well-characterized for immunotherapy studies in glioblastoma, GL261 is not representative of most human GBM tumors, and does not natively express ErbB2/Her2. The authors do not appear to have genetically engineered GL261 to express human ERBB2/HER2 (as is common when this model is used to study HER2-directed agents), and do not present characterization data that either the murine or human target is expressed in their model. Figure 3 implies that HER2 is present on the GL261 but there is no data presented to support this. Figure 6i is the only figure staining for ERBB2 and no isotype control is shown; methods do not include antibody information for this antibody. Experiments in which exogenous ErbB2 are added in vitro do not validate that the target is present. In this context, it is not possible to interpret their results showing exceptional responses to PL@mBiME in figure 5. **While several appropriate controls were included, the authors did not include any LNP controls without the anti-HER2 component of the mBiME (other than mLuc).** It is possible that in this system the other components of the mBiME (RP182 peptide, CD8 signal peptide, EGFP) and anti-PDL1 are responsible for these responses as opposed to HER2-specific interactions. Indeed, expression of luciferase alone has been shown to elicit immune responses in this model (PMID 32620877IF: 3.9 Q1). Further testing of this system in a glioma model in which the target is present and validated, and with appropriate controls to better understand the importance of each element of the mBiME would significantly increase the impact of this work. This reviewer acknowledges that the authors have attempted to characterize the immune microenvironment in Figures 7 and 8, but these experiments do not confirm a specific mechanism of immune activation that is specific to the cargo delivered in the LNP.

Response:

We thank the reviewer for raising this critical point regarding the choice of tumor model and target validation. We fully agree that establishing the relevance and specificity of our mBiME system for ErbB2 (HER2) is essential for interpreting its activity and therapeutic potential. We have addressed these concerns through additional experimental characterization, careful control design, and clarification of mechanisms.

1. ErbB2 expression in tumor models

To directly address your point, we quantified surface ErbB2 expression in GL261-Luc cell, as well as in CT-2A-Luc cells (another GBM model) and GL261-OE-Luc cells that exogenously overexpresses murine ErbB2 through flow cytometry. We did not overexpress human ErbB2 in this study because the ErbB2 antibody encoded by the BiME construct specifically recognizes murine ErbB2 only. We agree that overexpressing human ErbB2 in GBM cells and targeting it with a human ErbB2-specific antibody would further enhance the translational relevance of this strategy, and this will be an important direction for our future work.

Flow cytometry analysis confirmed that:

CT-2A-Luc shows low endogenous ErbB2 expression (17.8% positive), **GL261**-Luc displays moderate endogenous ErbB2 expression (47.8% positive), **GL261-OE**-Luc exhibits high ErbB2 expression (93.1% positive) (Figure R19a).

These data establish a graded ErbB2-expression system and confirm that GL261-Luc does express murine ErbB2 at appreciable levels, providing a relevant target for the mBiME platform.

We have added these data in revised manuscripts (**Supplemental Fig. 24a**), with corresponding text update in **Line 285-289**.

2. Target-dependent activity of BiME

To evaluate the specific contribution of the ErbB2 antibody in the BiME construct, we replaced the ErbB2 antibody sequence with luciferase to generate RP182-Luc mRNA. Co-culture of BMDMs with CT-2A-Luc, GL261-Luc, or GL261-OE-Luc cells revealed that, compared to PBS and PL@mRP182-Luc, PL@mBiME significantly increased the formation of physical "bridges" between macrophages and tumor cells and enhanced phagocytic activity in all cell groups (Figure R19b-f). This effect was ErbB2-dependent, as the higher conjugate formation and phagocytosis were observed only in PL@mBiME-treated group. In contrast, the PL@mRP182-Luc treatment group showed similar levels of "bridges" and phagocytosis across CT-2A-Luc, GL261-Luc, or GL261-OE-Luc cells (Figure R19b-f). These findings support the essential role of ErbB2 in mediating BiME-dependent macrophage engagement and tumor cell killing.

We have added these data in revised manuscripts (**Figure 3e-g, Supplemental Fig. 24b**), with corresponding text update in **Line 289-300**.

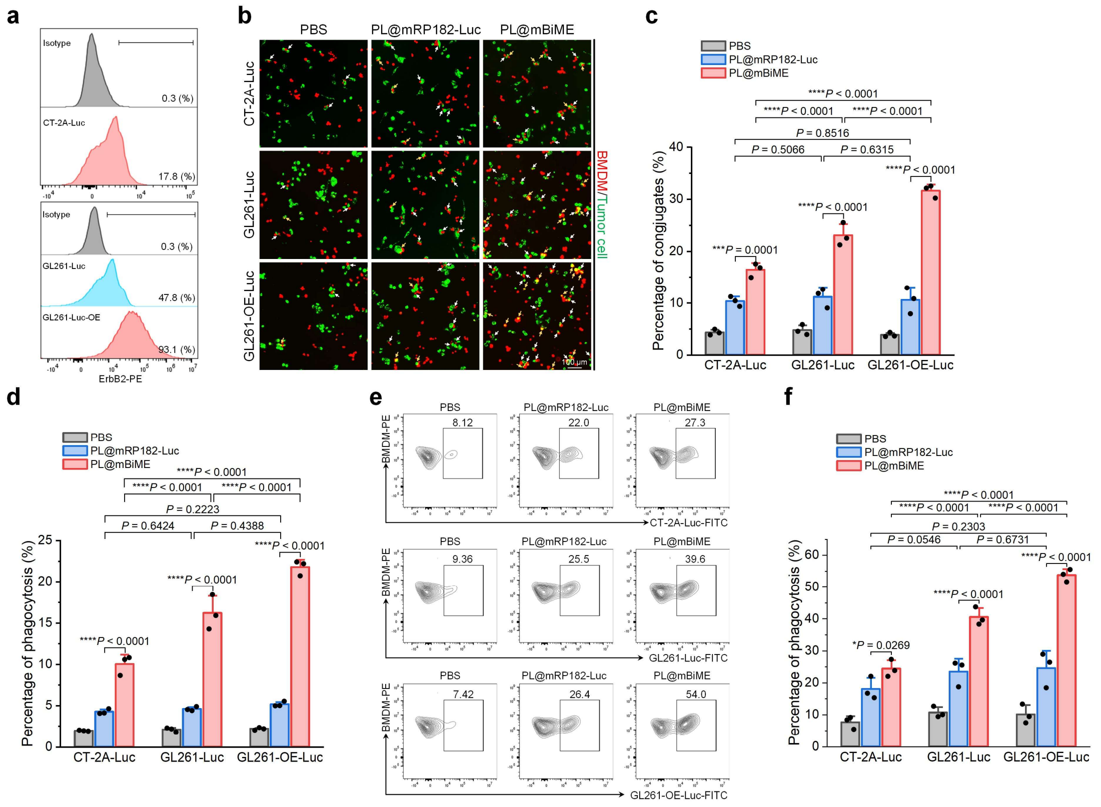

Figure R19. **a** Representative flow cytometry histograms of ErbB2 expression in CT-2A-Luc, GL261-Luc, and GL261-OE-Luc cell lines. **b-d** Representative fluorescence microscopy images and quantification of conjugates and phagocytosis between tumor cells (DiO, green) and BMDMs (DiI, red). White arrows indicate conjugate formation between BMDMs and tumor cells, while yellow arrows denote phagocytosis of tumor cells by BMDMs. Scale bar = 100 µm, n = 3 biologically independent replicates. Data are presented as mean ± SD, statistical analysis was performed by two-way ANOVA with Fisher's LSD post-hoc test. **e** Flow cytometry plots showing the phagocytic activity of BMDMs after co-incubation with CT2A-Luc, GL261-Luc, or GL261-OE-Luc cells under different treatment conditions (PBS, PL@mRP182-Luc, or PL@mBiME), n = 3 biologically independent replicates. **f** Quantification of phagocytosis based on the data presented in panel (e). Data are presented as mean ± SD, statistical analysis was performed by two-way ANOVA with Fisher's LSD post-hoc test.

To determine the role of ErbB2 in mediating the in vivo activity of PL@mBiME, we established orthotopic tumors using CT-2A-Luc (Figure R20a-c), GL261-Luc, and GL261-OE-Luc cells and treated mice with PBS, PL@mRP182-Luc, or PL@mBiME.

Consistent with the oncogenic function of ErbB2, GL261-OE-Luc tumors exhibited significantly accelerated growth compared to GL261-Luc tumors in both the PBS and PL@mRP182-Luc treatment groups (Figure R20d-f). In contrast, PL@mBiME treatment resulted in pronounced tumor growth inhibition and significantly prolonged survival in both GL261-Luc and GL261-OE-Luc models, with a greater degree of tumor suppression observed in the GL261-OE-Luc model and a higher proportion of tumor-free mice achieved in this group.

Representative tumors from CT-2A-Luc, GL261-Luc, and GL261-OE-Luc models were analyzed by immunofluorescence. Markedly increased colocalization of ErbB2 (green) and the macrophage marker F4/80 (red) was observed in PL@mBiME-treated tumors across all three models, indicating enhanced macrophage-tumor cell bridging events compared to both PBS- and PL@mRP182-Luc-treated groups. Notably, the extent of macrophage-tumor cell conjugate formation increased with tumor cell ErbB2 expression, progressing from CT-2A-Luc (low) to GL261-Luc (intermediate) and GL261-OE-Luc (high) (Figure R20g-h). Together, these findings demonstrate that PL@mBiME promotes macrophage-tumor cell physical interactions in an ErbB2-dependent manner in vivo, with higher tumor ErbB2 expression associated with enhanced therapeutic efficacy.

We have added these data in revised manuscripts (**Figure 6e-i, Supplemental Fig. 28**) with corresponding text update in **Line 530-548, 439-448**.

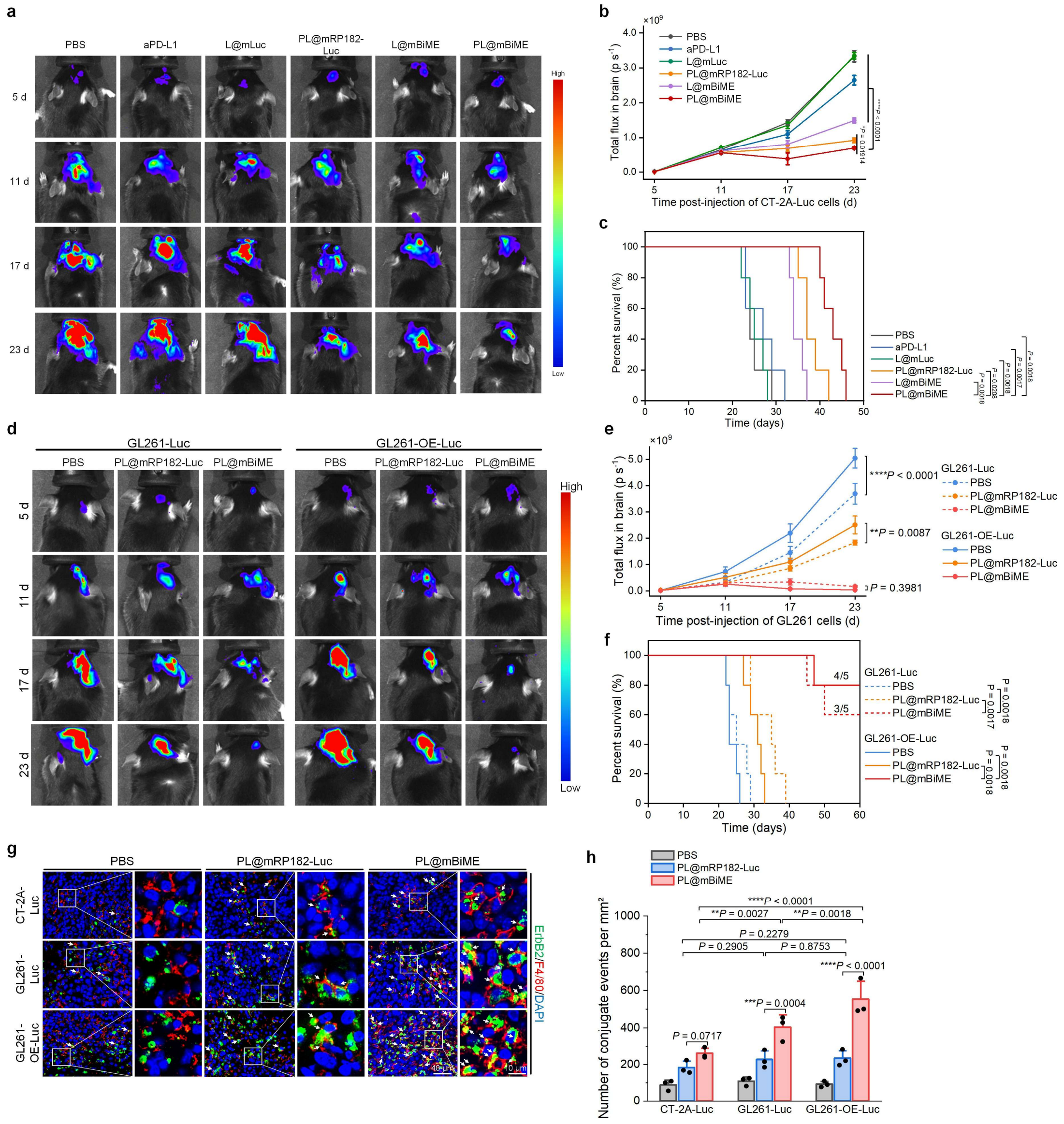

Figure R20. **a, b** Representative IVIS images and quantified signal intensities of CT-2A-Luc tumor-bearing mice treated with PBS, aPD-L1, L@mLuc, PL@mRP182-Luc, L@mBiME, and PL@mBiME, n = 3 biologically independent replicates. **c** Survival curves of CT-2A-Luc tumor-bearing mice following treatment with different formulations, n = 5 biologically independent replicates. **d, e** Representative IVIS images and quantified signal intensities of GL261-Luc and GL261-OE-Luc tumor-bearing mice treated with PBS, PL@mRP182-Luc and PL@mBiME, n = 3 biologically independent replicates. **f** Survival curves of GL261-Luc and GL261-OE-Luc tumor-bearing mice following treatment with different formulations, n = 5 biologically independent replicates. **g, h** Representative immunofluorescence (IF) images and statistical analysis of macrophage-tumor cell conjugation (green: ErbB2, red: F4/80, white arrows indicate macrophage-GL261 tumor cell conjugates). Scale bar = 50 μm, 20μm, n = 3 biologically independent replicates. Data are presented as mean ± SD.

Statistical analysis was performed by one-way ANOVA with Fisher's LSD post-hoc test for b. Statistical analysis was performed by two-way ANOVA with Fisher's LSD post-hoc test for e and h. Survival analysis (c and f) was compared using the log-rank test.

3. Control design and immune activation mechanism

To dissect the contribution of each component especially Anti-ErbB2, we included multiple LNP controls in Figure R21-22:

L@mLuc–lacks both RP182 and anti-ErbB2, without PD-L1 antibody

PL@mRP182-Luc– contains the RP182 peptide but lacks anti-ErbB2, with PD-L1 antibody

L@mBiME-full construct with RP182 and anti-ErbB2 scFv, without PD-L1 antibody

PL@mBiME– full construct with RP182 and anti-ErbB2 scFv, with PD-L1 antibody

Our data delineate a clear, stepwise immune activation mechanism mediated by PL@mBiME:

Enhanced Macrophage Phenotype and Antigen Presentation: Flow cytometric analysis of tumor-infiltrating macrophages revealed a significant increase in the proportions of $CD86^+$ (a co-stimulatory marker) and $MHC\text{-}II^+$ macrophages following PL@mBiME treatment (Figure R21) compared to all other groups. This shift indicates successful polarization of macrophages towards a pro-inflammatory, antigen-presenting state, thereby augmenting their capacity for tumor antigen processing and presentation.

Potentiated T Cell Priming and Cytotoxic Function: The improved antigen presentation might lead to a marked increase in the infiltration of both $CD4^+$ and $CD8^+$ T cells within the tumor microenvironment (Figure R22). Crucially, Granzyme B expression in $CD8^+$ T cells was significantly upregulated, demonstrating the activation of their potent cytotoxic killing function against tumor cells.

Integrated Cascade of Immune Activation: Collectively, these findings support a coherent mechanistic cascade:

BiME-mediated macrophage – tumor cell bridging

→ Enhances macrophage uptake and presentation of tumor antigens

→ Promotes the priming, activation, and proliferation of T cells (particularly $CD8^+$ T cells)

→ Boosts the Granzyme B-mediated cytotoxic activity of effector T cells.

This integrated mechanism underscores that the anti-tumor efficacy of PL@mBiME stems not only from direct macrophage phagocytosis but also from the subsequent activation of adaptive immune response. To confirm this, we conducted depletion

experiments using anti-CD8 antibodies to deplete $CD8^+$ T cells and clodronate liposomes to deplete macrophages (Figure R23a-h). The results showed that depletion of $CD8^+$ T cells mildly attenuated the tumor inhibition effects of PL@mBiME (Figure R23i-k). More strikingly, macrophage depletion significantly abrogated the anti-tumor effects of PL@mBiME (Figure R23i-k), providing direct functional evidence that macrophages are one of the principal effector cells in mediating the anti-tumor response. Overall, these results demonstrate that PL@mBiME activates both innate and adaptive immune responses to suppress tumor growth.

We have integrated these important depletion studies results into the revised manuscript, in **Fig. 6, Fig. 7 and Supplementary Fig. 52a-h** and the corresponding text in **line 511--606** in the Results section.

4. Limitations and future directions

We acknowledge the limitation that our current BiME construct targets murine ErbB2, which restricts immediate clinical translation. We have explicitly discussed this point in the revised manuscript and emphasized that future studies will focus on **humanizing both the target and the antibody component** to validate translational potential. In parallel, we will employ patient-derived xenograft or mouse models expressing human HER2 to further confirm target specificity, toxicity and therapeutic relevance. (In revised manuscript **Line 733-738**)

Summary

Collectively, the added data and controls confirm that:

1. GL261 expresses murine ErbB2 (47.8%).
2. Appropriate controls confirmed that inclusion of the anti-ErbB2 antibody in the BiME construct enhanced immune activity compared with RP182 alone, and this enhancement was dependent on ErbB2 expression.
3. The therapeutic effect integrates RP182-driven macrophage polarization and ErbB2-specific bridging/phagocytosis.
4. The depletion assay confirmed that both innate immunity, particularly macrophage-mediated responses, and adaptive immunity play essential roles in the anti-tumor efficacy of PL@mBiME.

We believe these revisions strengthen the mechanistic rigor of our study and provide a clear foundation for future translational development. Thank you again for the insightful critique, which has significantly improved the manuscript.

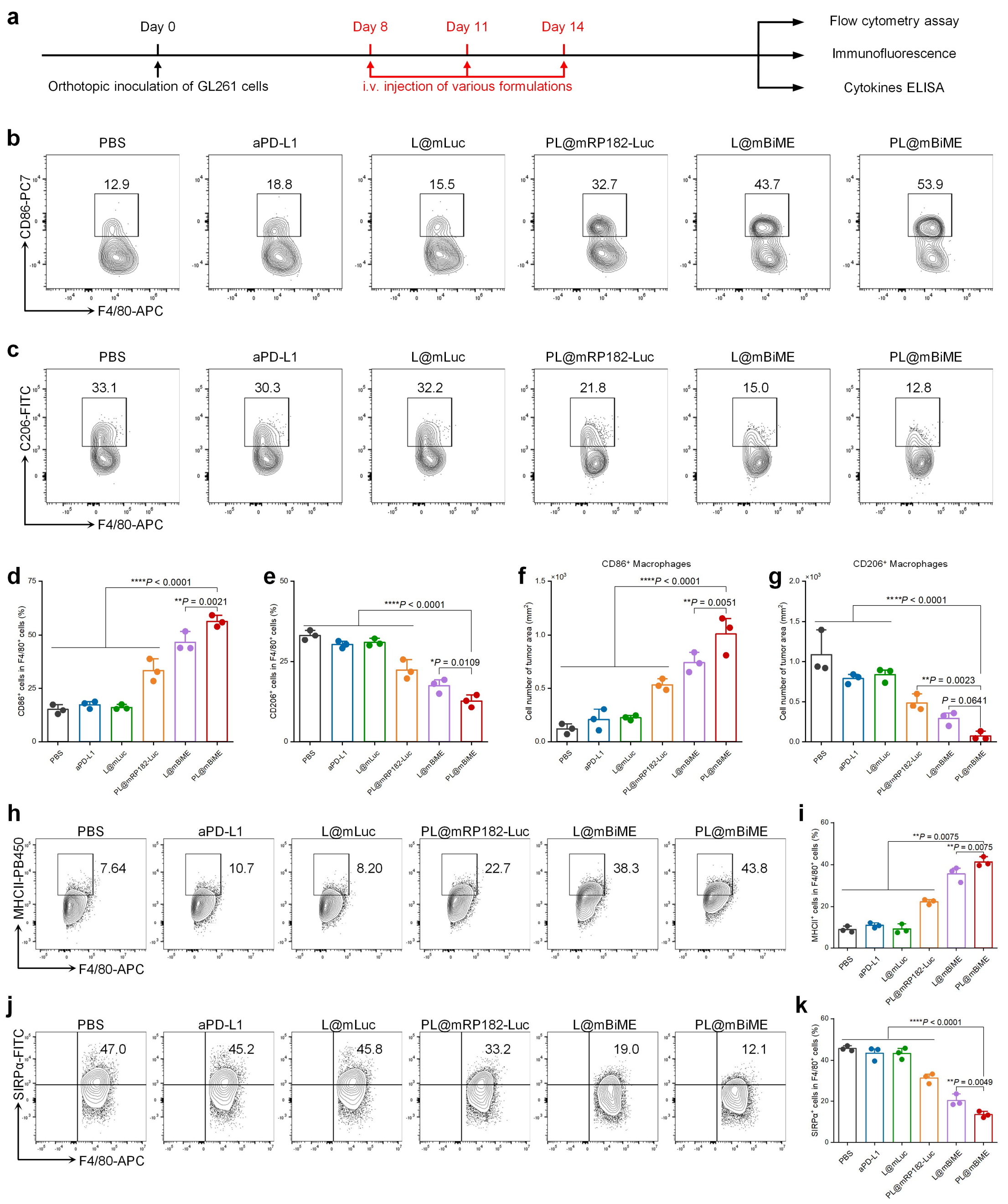

Figure R21. a Timeline for examining the *in vivo* immune response in the GL261 bearing mice induced by PL@mBiME. **b-e** Representative flow cytometry contour plots and quantification of tumor-infiltrating TAM polarization (M1 marker CD86, M2 marker CD206) cells three days after three treatments with PBS, aPD-L1, L@mLuc, PL@mRP182-Luc, L@mBiME, and PL@mBiME, n = 3 biologically independent replicates. **f, g** Statistical charts of CD86$^+$ Macrophage and CD206$^+$ Macrophage in immunofluorescence sections, n = 3 biologically independent replicates. **h, i** Representative flow cytometric images and quantification of MHCII$^+$ antigen presentation in TAMs following treatment with different formulations, n = 3 biologically independent replicates. **j, k** Representative flow cytometric images of SIRPα$^+$ in TAMs following treatment with different formulations, n = 3 biologically

independent replicates. Data are presented as mean ± SD, statistical analysis was performed by one-way ANOVA with Fisher's LSD post-hoc test.

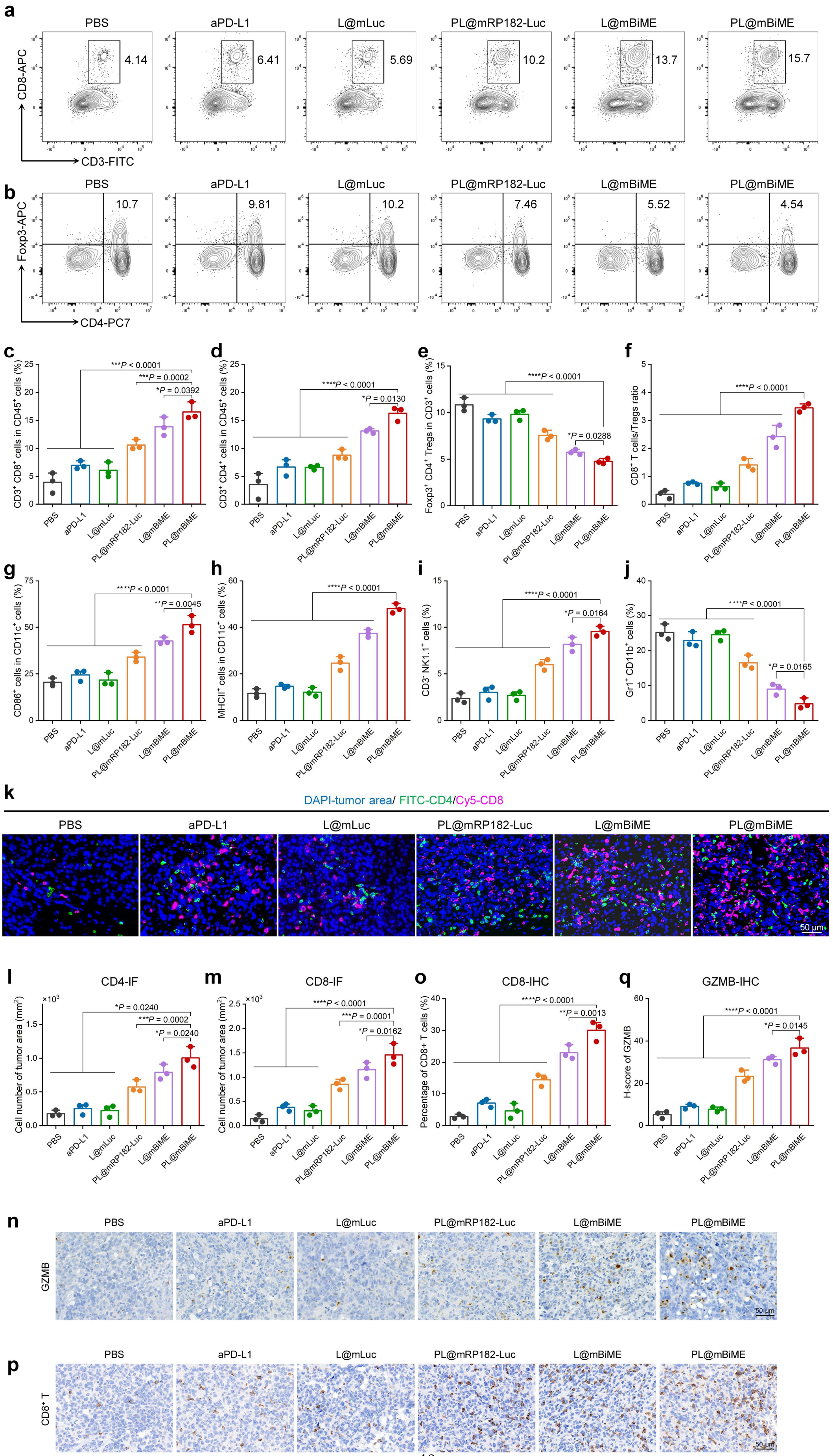

a
PBS
aPD-L1
L@mLuc
PL@mRP182-Luc
L@mBiME
PL@mBiME
CD8-APC
CD3-FITC
4.14
6.41
5.69
10.2
13.7
15.7
b
Foxp3-APC
CD4-PC7
10.7
9.81
10.2
7.46
5.52
4.54
c
d
e
f
g
h
i
j
k
DAPI-tumor area/ FITC-CD4/Cy5-CD8
50 μm
l
CD4-IF
m
CD8-IF
o
CD8-IHC
q
GZMB-IHC
n
GZMB
p
CD8+ T
50 μm

Figure R22. a, b Representative flow cytometry plots of tumor-infiltrating CD8 and Treg cells three days after three injections of PBS, aPD-L1, L@mLuc, PL@mRP182-Luc, L@mBiME, and PL@mBiME treatments. **c-j** Flow cytometry statistical quantification diagrams of tumor-infiltrating CD8, CD4, Treg, CD8/Treg, DC (MHCII, CD86), NK, MDSC three days after three injections of PBS, aPD-L1, L@mLuc, L@mBiME, and PL@mBiME treatments, n = 3 biologically independent replicates. **k-m** Immunofluorescence (IF) images and quantification of tumor-infiltrating T cells three days after three injections of PBS, aPD-L1, L@mLuc, PL@mRP182-Luc, L@mBiME, and PL@mBiME treatments (blue: DAPI; green: Spgreen-CD4; purple: Cy5-CD8; n = 3 biologically independent replicates, scale bar = 50 μm). **n** Representative immunohistochemical (IHC) staining for $CD8^+$ cytotoxic T lymphocytes within tumor sections from mice receiving the indicated treatments. **o** Quantification of $CD8^+$ T cell positivity rate (percentage of $CD8^+$ cells among total nucleated cells) from images in panel (n), n = 3 biologically independent replicates. **p** Representative IHC staining for the cytolytic effector protein Granzyme B (GZMB). **q** Quantification of GZMB expression level using the H-Score (Histochemical Score) method, which integrates both the intensity and proportion of positive staining, from images in panel (p), n = 3 biologically independent replicates, scale bar = 50 μm. Data are presented as mean ± SD, statistical analysis was performed by one-way ANOVA with Fisher's LSD post-hoc test for c-j, l, m o and q.

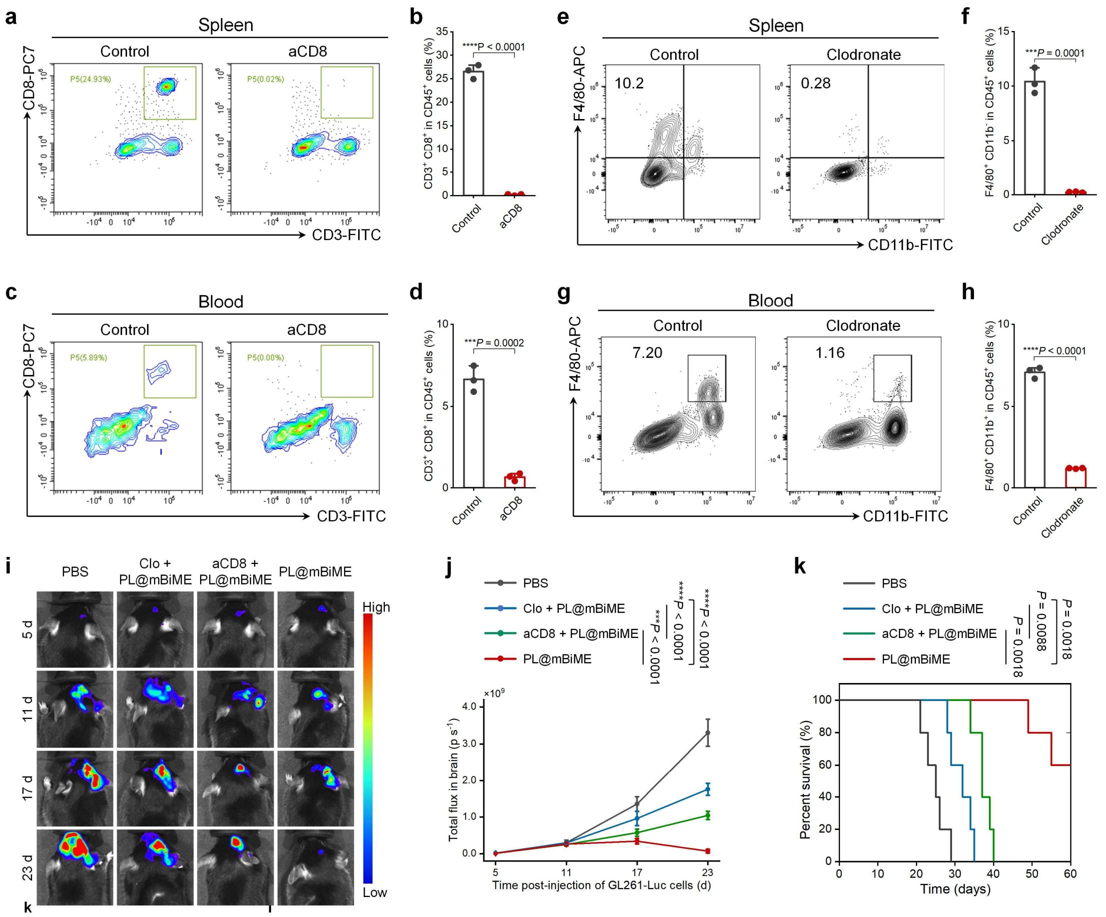

Figure R23. ***In vivo* depletion of specific immune subsets and its impact on PL@mBiME efficacy. a-d** Validation of $CD8^+$ T cell depletion. Representative flow cytometry dot plots (a, c) and corresponding quantification (b, d) show the frequency of $CD8^+$ T cells among $CD45^+$ leukocytes in the spleen (a, b) and peripheral blood (c, d) of mice treated with anti-CD8α depleting antibody. **e-h** Validation of macrophage depletion. Representative plots (e, g) and quantification (f, h) show the frequency of $F4/80^+$ macrophages among $CD45^+$ leukocytes in the spleen (e, f) and blood (g, h) of mice treated with clodronate liposomes. Data in b, d, f, h are presented as mean ± SD, n = 3 biologically independent replicates. Statistical significance was determined by unpaired t-test (b, d, f, h). **i, j** Serial *in vivo* bioluminescence images (k) and quantification of tumor flux (l) show tumor growth in GL261 tumor-bearing mice treated with PL@mBiME following depletion of the indicated immune cell populations, n = 3 biologically independent replicates. **k** Kaplan-Meier survival curves of the corresponding treatment groups, n = 5 biologically independent replicates. Data are presented as mean ± SD, statistical analysis was performed by one-way ANOVA with Fisher's LSD post-hoc test for j. Survival analysis (k) was compared using the log-rank test.

Minor Concerns

1. Benchmarking to existing literature: The manuscript does not clearly articulate its incremental advance over prior studies. Explicitly state how this work differs from and improves upon existing literature.

Response:

We thank the reviewer for this important point and agree that our initial submission did not sufficiently emphasize the incremental advance relative to prior bispecific engager and mRNA/LNP literature. In the revised manuscript, we will revise and add discussion that benchmarks our work against three relevant bodies of prior literature:

1, Protein-format bispecific immune engagers in solid tumors and GBM: Protein BiTE/TCE platforms have shown clinical success in hematologic malignancies but face key barriers in solid tumors, including limited tissue penetration, systemic toxicity, and dependence on high T-cell infiltration[4, 5, 6]. In GBM, these barriers are amplified by the BBB and an immunosuppressive, macrophage-rich microenvironment.

2, mRNA-encoded bispecific antibodies delivered by LNPs—mostly outside the CNS: Recent studies demonstrate the feasibility of in vivo expression of bispecific antibodies from mRNA-LNPs, improving manufacturability and pharmacokinetics relative to protein dosing; however, these efforts have largely focused on peripheral tumors and organ-selective delivery rather than BBB-restricted GBM. For example, LNP-mRNA encoding bispecific antibodies has been used to drive antitumor activity in non-CNS settings (e.g., bispecific antibody-encoding mRNA approaches in solid tumors and organ-selective delivery concepts[7, 8]).

3, Macrophage-engaging bispecifics (BiMEs) for targeted phagocytosis—typically protein-based and not brain-delivered: Bispecific macrophage-engaging antibodies that increase tumor–macrophage conjugates and phagocytosis have been reported (e.g., MerTK/EGFR macrophage-engaging constructs), supporting the general principle of "bridging" to promote phagocytosis, but these platforms are not designed for BBB delivery or tumor-microenvironment-responsive activation in GBM[4].

Our incremental advance over this literature is threefold:

1, Effector choice and mechanism: Unlike T cell–dependent engagers, we implement a macrophage engager designed to repurpose the dominant immune population in GBM (TAMs), combining reprogramming (RP-182/CD206 axis) with physical bridging (ErbB2×CD206) to increase macrophage–tumor conjugates and phagocytosis.

2, Delivery innovation for GBM: We introduce a brain-tumor-targeted and tumor-responsive LNP that integrates BBB transport (A2) with pH-triggered charge reversal/escape and GSH-triggered local release of a checkpoint antibody—addressing the central limitation of insufficient brain delivery seen in many mRNA/LNP immunotherapy approaches.

3, Coordinated dual-therapy in one platform: Co-delivery of mRNA-encoded engager

+ locally released anti–PD-L1 is designed to synchronize innate activation and adaptive support within the same niche, enabling durable responses and immune memory.

In summary, we agree and have revised the existing discussion paragraph (Revised manuscript **Line 688-697**) to explicitly benchmark our work against prior protein-format engagers and prior mRNA-encoded bispecific antibody studies. We now cite key reports demonstrating mRNA-encoded bispecific antibodies in vivo (e.g., Stadler et al., *Nat Med* 2017; Stadler et al., *Sci Transl Med* 2024) and recent mRNA/LNP bispecific platforms in non-CNS contexts (e.g., *Adv Sci* 2023; *Nat Commun* 2025), and we explicitly state the incremental advance of our work: a macrophage engager mechanism combined with a BBB-penetrant and tumor-responsive delivery design tailored for GBM.

To strengthen this point further, we also added a paragraph (in revised manuscript **Line 705-712**) distinguishing our "reprogram-and-engage" strategy from macrophage depletion approaches (e.g., CSF1R inhibition), emphasizing preservation of antigen presentation and avoidance of compensatory effects.

2. Experimental Design & Quantification:

- Methods for nanoparticle synthesis are not sufficient to allow researchers to replicate your methods. Equipment, mixing method and speed, temperature, concentration, etc were not described.

Response:

We sincerely apologize for the inadequacy of the nanoparticle synthesis methods described in the initial manuscript, which failed to provide sufficient details for reproducibility. We fully recognize that precise and comprehensive methodological descriptions are fundamental to ensuring the replicability of experimental results, and we have thoroughly revised and expanded the "Nanoparticle Synthesis" subsection in the Materials and Methods section of the revised manuscript to address this critical issue.

The updated methods now include detailed, step-by-step descriptions of all key parameters, including equipment, mixing conditions, temperature control, reagent concentrations, and post-synthesis processing. A summary of the supplemented core details is as follows:

Preparation of LNPs

DCPA was synthesized in Supplementary Fig. 2. A lipid mixture composed of MC3 or SM102, DCPA or DSPC, cholesterol, and DSPE-PEG2000-NH2 (MedChemExpress) was prepared in ethanol as a 10 mg mL^{-1} stock solution, with a molar ratio of

50:10:38.5:1.5. The diluted lipid solution (4.5 mg mL^{-1}) was mixed with an aqueous mRNA solution (0.1 mg mL^{-1}) in 10 mM citrate buffer (pH 4) at a 1:3 organic-to-aqueous volume ratio using a microfluidic mixing device (INano X, Micronanobiologics) at a total flow rate of 2.4 mL/min at room temperature. The resulting LNPs were immediately diluted twofold with Tris-HCl buffer (pH 7.4, 0.01 M), followed by three rounds of ultrafiltration at 2000 × g using 10-kDa Amicon Ultra centrifuge filters (Millipore, Burlington, MA) at 4° C, each time diluted with Tris-HCl buffer (pH 7.4, 0.01 M). Subsequently, the LNPs were filtered through a 0.22-μm membrane.

Preparation of L@mBiME and PL@mBiME

Roughly 0.1% M/M of DSPE-PEG2000-A2 mixture was added to the formulated LNPs. The reaction was incubated at 4 °C for 48 h. The post-inserted LNP was then concentrated by an ultrafiltration system and slowly applied to a 90-cm-bed-length gravity-flow size exclusion column prepared with Sepharose-CL4B gel. The mobile phase was PBS. The fractions that contained LNP were collected and concentrated by the ultrafiltration to obtain L@mBiME. To release the encapsulated or surface-conjugated A2 peptide, the nanoparticles were treated with 0.5% (v/v) Triton X-100 in PBS for 30 minutes at 37 ° C. After centrifugation to remove insoluble debris, the supernatant was collected, and the A2 peptide concentration in the lysate was determined using a Fluorometric Peptide Quantitation Assay Kit (Beyotime Biotechnology) according to the manufacturer's protocol.

To construct the final formulation, the obtained L@mBiME was conjugated with an anti-programmed cell death ligand 1 (aPD-L1) antibody via a redox-responsive linker. Specifically, the heterobifunctional crosslinker NPC-SS-NPC (where NPC denotes a nitro phenyl carbonate ester group reactive toward amines, and SS denotes a cleavable disulfide bond) was employed. The crosslinker was added at a molar ratio of 1.2:1 (crosslinker to aPD-L1) to ensure efficient bridging. This mixture was then reacted with L@mBiME at a fixed molar ratio of aPD-L1 to L@mBiME at 0.5%. The conjugation reaction was conducted in PBS buffer (0.01 M PBS, pH 7.4) at 4 ° C for 12 – 16 hours with gentle stirring.

Following the reaction, the crude mixture was purified to remove unreacted crosslinker and antibody. The solution was transferred to a Vivaspin ultrafiltration device (Sartorius) with a molecular weight cut-off (MWCO) of 300 kDa and centrifuged. This process was repeated with multiple cycles of dilution and concentration (diafiltration) using the reaction buffer to thoroughly exchange the medium. The final retentate, containing the purified antibody-conjugated nanoparticles, was collected and designated as PL@mBiME. For the quantification of conjugated aPD-L1, the PL@mBiME nanoparticles were treated with 0.5% (v/v) Triton X-100 to disrupt the lipid bilayer and

release the antibody. The aPD-L1 concentration in the supernatant was quantified using a commercial Rat IgG ELISA Kit (Beyotime Biotechnology) following the standard protocol. This assay measures the total IgG content, which directly corresponds to the amount of aPD-L1 present in the nanoparticle formulation.

The descriptions mentioned above have been incorporated into the Materials and Methods section in manuscript text **line 760-770** and supplementary information **line 53-114**.

- Characterization of DSPE-PEG-A2 is incomplete – how was conjugation confirmed? How many molecules of A2 are present, on average, per molecule. Similarly, how many molecules of aPDL1 are on each nanoparticle?

We sincerely appreciate your critical comment pointing out the incomplete characterization of DSPE-PEG-A2 and the lack of quantitative data on ligand valency (A2 and aPDL1 molecules per nanoparticle). We fully recognize that rigorous validation of conjugate formation and precise determination of ligand density are essential to demonstrate the reliability of our targeting LNP platform, and we have supplemented comprehensive characterization data in the Materials and Methods and Supplementary Information (**Supplementary Fig. 4-5**) of the revised manuscript.

To verify the successful and stable conjugation of angiopep-2 (A2) peptide and anti-PD-L1 (aPD-L1) antibody to the LNP surface, we have added the following experiments and data:

1. Confirmation of DSPE-PEG-A2 Covalent Conjugation via MALDI-TOF-MS

DSPE-PEG-A2 was synthesized by the Michael addition reaction between maleimide-functionalized DSPE-PEG_{2000} (DSPE-PEG_{2000}-NHS) and Angiopep-2. The successful covalent conjugation was verified using matrix-assisted laser desorption/ionization time-of-flight mass spectrometry (MALDI-TOF-MS, Bruker ultrafleXtreme). The theoretical molecular weight of DSPE-PEG_{2000}-NHS is ~3000 Da, and that of A2 is 2301.48 Da; the theoretical molecular weight of the DSPE-PEG-A2 conjugate is thus ~5300 Da. MALDI-TOF-MS analysis revealed a series of characteristic m/z peaks in the range of 4900.7954 – 5736.8644 Da, which fully covers the theoretical molecular weight of the DSPE-PEG-A2 conjugate (Figure R24a). The minor deviation from the theoretical value (±10%) is attributed to the polydispersity of the PEG_{2000} segment

We performed Western blot analysis to confirm the PEGylation of aPD-L1. Proteins were probed with an anti-PEG antibody and detected by enhanced chemiluminescence. The anti-PEG antibody showed no signal in native aPD-L1 but clear bands in the reaction mixture and purified product, confirming successful covalent attachment of PEG to aPD-L1 (Figure R24b).

These data have been incorporated into the revised manuscript in **Supplementary Fig.**

4-5.

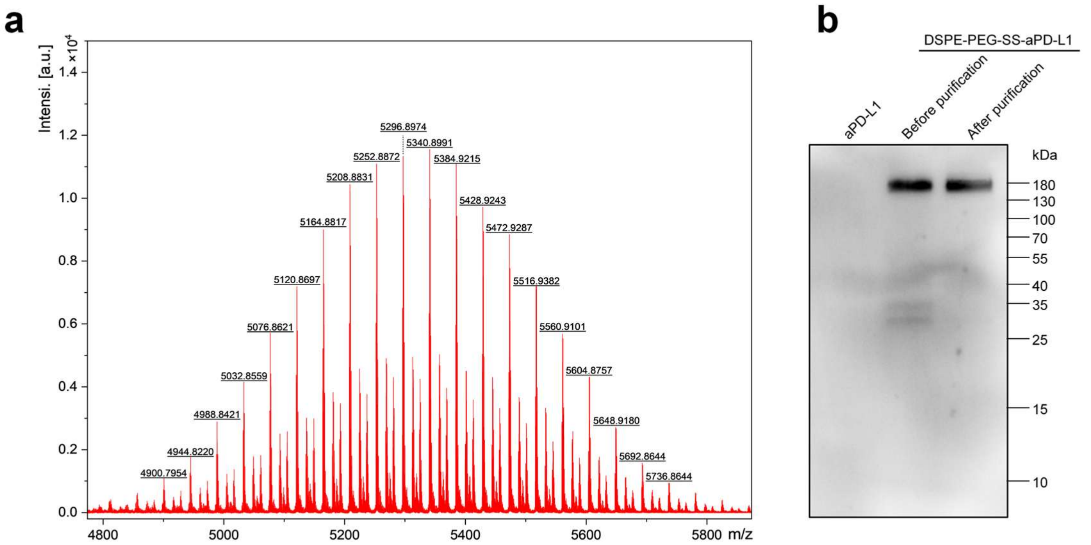

Figure R24. a The matrix assisted laser desorption ionization time of flight mass spectrometry (MALDI-TOF-MS) spectrum of DSPE-PEG-A2. **b** Detection of PEGylation of aPD-L1 by Western blot. Proteins were separated by SDS-PAGE and probed with an anti-PEG antibody (PEG-B-47, abcam) to confirm PEGylation, Lane 1: Native aPD-L1 (non-PEGylated control); Lane 2: Reaction mixture of aPD-L1 and DSPE-PEG-NH_2 conjugated via the NPC-SS-NPC crosslinker before purification; Lane 3: Purified PEGylated aPD-L1.

2. Determination of Ligand Valency (Molecules per Nanoparticle)

To quantify the efficiency of targeting ligand insertion, nanoparticles post-inserted with DSPE-PEG-A2 were lysed with 0.5% (v/v) Triton X-100. The concentration of the released A2 peptide in the lysate was determined using a Fluorometric Peptide Quantitation Assay Kit (Beyotime Biotechnology) according to the manufacturer's protocol.

Subsequently, the antibody-conjugated prodrug nanoparticles (PL@mBiME) were obtained by conjugating aPD-L1 via a crosslinker, followed by purification using ultrafiltration (MWCO: 300 kDa). To quantify the amount of aPD-L1 conjugated to PL@mBiME, the nanoparticles were similarly treated with 0.5% (v/v) Triton X-100 to release the antibody. Subsequently, the aPD-L1 concentration in the supernatant was quantified using a Rat IgG ELISA Kit (Beyotime Biotechnology) as per the standard protocol, which measures total IgG content corresponding to the conjugated aPD-L1.

The LNP concentration was determined by nanoparticle tracking analysis (NTA) using a NanoSight Pro (Malvern Panalytical). The experimental procedure was performed according to a previously published method[9].

$$Number\ of\ A2\ per\ LNP\ =\frac{\rho_{A2}\times N_A}{c_{LNP}\times M_{A2}}$$

ρ_{A2} : mass concentration of the A2 peptide
N_A : Avogadro constant
C_{LNP} : particle concentration of LNPs
M_{A2} : molar mass of the A2 peptide
Similarly, the number of aPD-L1 antibodies per LNP was calculated using the same method. The results showed that the average number of aPD-L1 molecules per LNP is ~120 ± 15, and each LNP carries an average of ~35 ± 5 A2 molecules
These data have been incorporated into the revised manuscript in **Supplementary Table 2**, with the corresponding text updated in supplementary information **line 116-138**.

- Figures 3 and 4 both attempt to show mechanistically how the nanoparticles deliver their cargo to specific cells, but ultimately it is not clear how and when the GSH-mediated cleavage and charge reversal are occurring. In the in vitro setting at least, this could be further probed through pre-incubation of LNPs with GSH to isolate the role of this cleavage as was explored for pH in Fig 2

Response:
We sincerely thank the reviewer for raising these insightful questions, which help clarify how PL@mBiME responds to the tumor microenvironment (TME). The in vivo glutathione (GSH) concentration in the TME was quantified using a commercial GSH assay kit and is presented in Figure R25a. The results show that the GSH concentration in the TME is approximately 4.75 mM (Figure R25a-b).
Based on this observation, we further evaluated PD-L1 antibody release in vitro under 4 mM and 10 mM GSH conditions. The results showed that aPD-L1 was gradually released in the presence of GSH, reaching a plateau of approximately 50% and 75% release at 12 hours under 4 mM and 10 mM GSH, respectively. In contrast, negligible release was observed in the absence of GSH (Figure R25c).
To further elucidate the timing of PD-L1 antibody release, we labeled aPD-L1 with FITC and mBiME with Cy5 dye. We then co-incubated PL@mBiME with GL261 cells for in medium containing either 0 mM or 4 mM GSH. Confocal microscopy images showed that under 4 mM GSH conditions, most of the PD-L1 antibody was released extracellularly (Figure R25d), with no co-localization signal observed with Cy5-mBiME (Figure R25e). This result indicates that PD-L1 antibody undergoes extracellular release in response to GSH in the tumor microenvironment prior to cellular uptake.

Our experimental data support that the charge reversal occurs preferentially and

extracellularly within the tumor microenvironment (TME), thereby facilitating selective uptake by tumor cells.

The underlying mechanism can be summarized as follows:

We measured the extracellular pH of our tumor model to be approximately 6.5 (Figure R25f). The zeta potential data confirm that our LNPs undergo a significant negative-to-positive charge reversal precisely within this acidic pH range (Figure R25g).

Therefore, upon extravasation into the TME, LNPs encounter an acidic milieu (pH ~6.5) and reverse their surface charge to positive before encountering tumor cells. This pre-charge reversal is critical because:

It enhances electrostatic attraction between the now positively charged LNPs and the negatively charged membranes of tumor cells.

This interaction significantly promotes cellular uptake (internalization) specifically within the tumor, as observed in our in vitro and in vivo uptake studies.

In summary, the pH-triggered charge reversal is designed to be a tumor-localized event. It acts as a "switch" that is turned ON in the acidic TME, ensuring that the enhanced, charge-mediated uptake is spatially confined to the tumor site. This mechanism aligns perfectly with our observed results: superior tumor accumulation and gene expression, coupled with reduced distribution and activity in normal organs.

These data have been incorporated into the revised manuscript in **Fig. 1m, Supplementary Fig. 8, Supplementary Fig. 9, and Fig. 1j**.

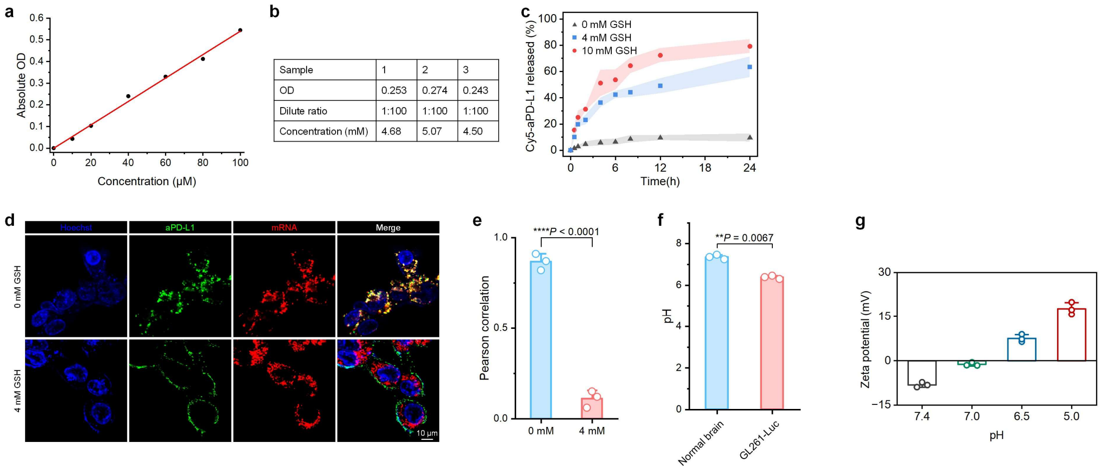

Figure R25. a Quantification of glutathione (GSH) concentration. Standard curve for the GSH assay kit. Serial dilutions of a known GSH standard were used to generate the curve for calculating sample concentrations. **b** Quantified GSH concentrations in brain with tumor, as determined using the standard curve from panel (a). **c** Responsive release of the Cy5-aPD-L1 in 0, 4, or 10 mM GSH (n = 3 independent replicates). Data are presented as mean ± SD. **d** GSH-triggered aPD-L1 release of PL@mBiME. Intracellular distribution of FITC-labeled aPD-L1 (green) and Cy5-labeled mBiME

(red) in GL261 cells after 4 h, with 0 mM or 4 mM GSH. Scale bar = 25 μm. **e** Pearson's correlation analysis confirms GSH-responsive carrier disassembly (reduced co-localization) at high GSH. Data are presented as mean ± SD, n = 3 biologically independent replicates; unpaired two-tailed Student's t-test. **f** Measurement of intratumoral acidity in GL261-Luc tumors compared to normal brain tissue, n = 3 biologically independent replicates. **g** Zeta potentials of PL@mBiME at different pH values in Tris-HCl buffer (n = 3 independent replicates).

- The use of 'docking studies' in 3m does not prove mechanism, and inclusion is questionable in this paper

Response:

We thank the reviewer for this important point and agree that molecular docking alone does not constitute experimental proof of mechanism. Our intention was not to use docking to demonstrate causality, but rather to provide structural rationale supporting the rational design of the BiME construct.

The docking analysis in Figure 3k is therefore presented as supportive, illustrative evidence, complementing our extensive experimental data—including macrophage–tumor conjugate formation, ErbB2-dependence, functional phagocytosis assays, and in vivo validation—which together establish the biological mechanism.

To avoid overinterpretation, we have revised the manuscript text to explicitly state that the docking results are predictive and illustrative, and we have removed any language implying that docking alone proves the mechanism.

The description of this part has been revised in revised manuscript **Line 306-314**.

- Quantitative analyses (e.g., Pearson/Manders coefficients, permeability metrics) would strengthen the conclusions of imaging data.

Response:

Thank you for the valuable suggestion to strengthen the quantitative analysis of our imaging data. We fully agree that applying objective, quantitative metrics will significantly bolster the conclusions drawn from our microscopy and in vivo imaging results.

In direct response to your recommendation, quantitative assessment of Pearson/Manders coefficients and permeability parameters was conducted (Figure R26).

Thank you again for this critical suggestion, which has greatly improved the analytical depth of our study.

These data have been incorporated into the revised manuscript in Supplementary Fig. 9, Supplementary Fig. 12 (Pearson/Manders coefficients) and Fig. 4b (permeability metrics).

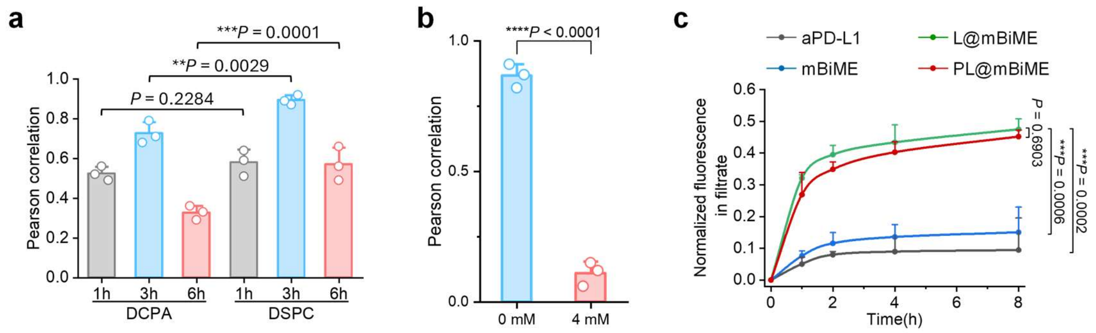

Figure R26. a The Pearson correlation of DCPA-PL@mBiME and DSPC-PL@mBiME at 1h, 3h, and 6h, n = 3 biologically independent replicates. All statistical quantities are presented as mean ± SD. Statistical significance was evaluated by one-way analysis of variance. **b** Pearson’ s correlation analysis confirms GSH-responsive carrier disassembly (reduced co-localization) at high GSH. Data are presented as mean ± SD, n = 3 biologically independent replicates; unpaired two-tailed Student's t-test. **c** Release of the Cy5-PL@mBiME distribution in the chamber after incubation with aPD-L1, mBiME, L@mBiME and PL@mBiME for 1, 2, 4 and 8 h, n = 3 biologically independent replicates.

- Clearly report the number of biological and technical replicates.

Response:

Thank you for raising this important point regarding experimental rigor. We have now clearly specified the number of biological and technical replicates for each relevant experiment throughout the revised Methods and figure legends.

- The authors only include male mice, which is not appropriate for a preclinical evaluation in a tumor that impacts both sexes.

Response：

We thank the reviewer for raising this important point regarding sex as a biological variable. We fully agree that glioblastoma affects both males and females, and that inclusion of both sexes is important for preclinical evaluation.

To address this concern during revision, a balanced cohort of male and female mice (male:female = 1:1) were used in all the additional in vivo experiments during revision.

- While toxicity was not the focus of this paper, it would be expected based on the biodistribution data reported that the mBiME is expressed in the liver. Staining for mBiME and anti-PDL1 should be evaluated using tissue in S24.

Response:

Thank you for this insightful comment. We agree that understanding the expression

pattern is important for a comprehensive safety profile. For visualizing BiME localization within tissues, EGFP expression was examined in brain and liver tissue after injecting PL@mBiME-T2A-EGFP. Strong EGFP fluorescence was observed specifically within the tumor region in brain whereas EGFP expression in liver is relatively even (Figure R27a-b), suggesting successful tumor-targeted expression in brain. Furthermore, we also measured the release of aPD-L1 payload in both brain tumor and liver tissue. As expected, aPD-L1 was significantly lower in the liver compared to the tumor-bearing brain, due to potentially much lower GSH level (Figure R27c-d).

Meanwhile, given that the liver is the primary off-target organ, we conducted a comprehensive evaluation of the potential impact on Kupffer cells (the liver-resident macrophages). Furthermore, to systematically investigate the systemic safety profile of this therapy, behavioral tests were also performed on the experimental animals. The findings from the Kupffer cell assessment, together with the behavioral test results, are presented in **Supplementary Fig. 36 and Supplementary Fig. 37** to further inform the favorable systemic safety profile.

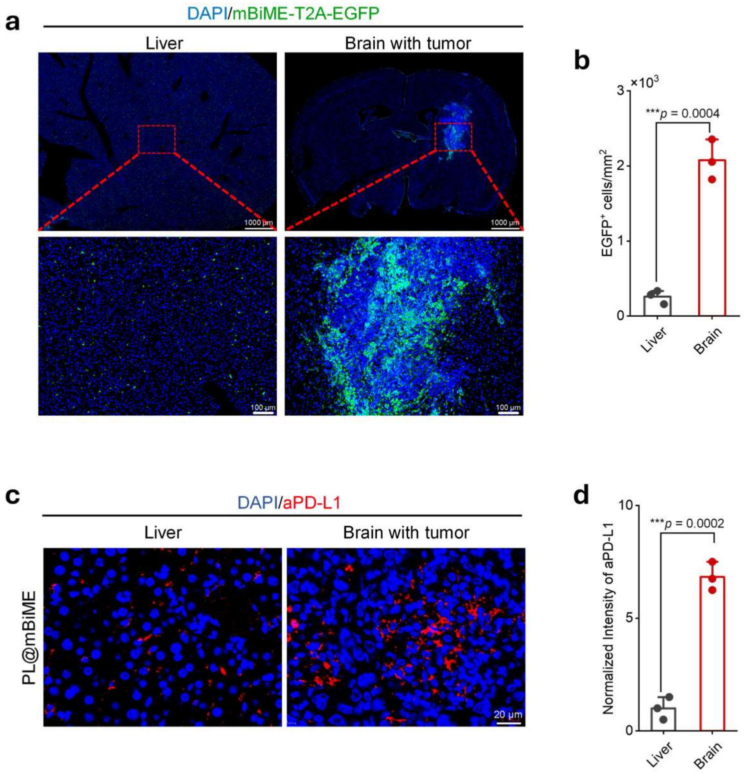

Figure R27. a Representative fluorescence images showing the expression of mBiME-EGFP (green) in liver tissues and tumor-bearing brain tissues. Nuclei were counterstained with DAPI (blue). Scale bar = 1000 μm, 100μm. **b** Quantitative analysis of mBiME-EGFP fluorescence intensity in liver and tumor-bearing brain tissues. The fluorescence intensity was quantified using ImageJ software, and data are presented as

mean ± SD (n = 3 biologically independent replicates). Statistical significance was determined by unpaired two-tailed t-test. **c** Representative fluorescence images showing the release of aPD-L1 (red) in liver tissues and tumor-bearing brain tissues. Nuclei were counterstained with DAPI (blue). Scale bar = 20 μm. **d** Quantitative analysis of aPD-L1 fluorescence intensity in liver and tumor-bearing brain tissues. Data are presented as mean ± SD (n = 3 biologically independent replicates). Statistical significance was determined by unpaired two-tailed t-test.

3. Data Presentation

- Several figures lack scale bars, error bars, or statistical details.

Response:

We sincerely apologize for the omissions of scale bars, error bars, and statistical details in several figures of the initial manuscript. We fully acknowledge that these deficiencies compromise the reproducibility and interpretability of our experimental results, and we highly appreciate your critical feedback to improve the rigor of our study.

To address these issues comprehensively, we have performed systematic revisions on all affected figures, including **Figure 1f-g, Supplementary Fig.7b, Supplementary Fig.10, Supplementary Fig.11b, Supplementary Fig.12, Supplementary Fig.15, Supplementary Fig.16**, etc.

- Some conclusions over-interpret correlation as causation. E.g. “Further experiments confirmed that DCPA-based LNPs mediated efficient EGFP expression in…” refers to a supplemental micrograph without replicates or quantification.

Response:

Thank you for this important methodological critique regarding data interpretation. We agree that conclusions must be supported by rigorous and quantitative evidence.

You are correct to point out that the original statement regarding DCPA-based LNPs and EGFP expression in the referenced supplementary micrograph lacked explicit quantification. We have now addressed this by providing quantitative analysis for all key imaging and flow cytometry data mentioned in that section. Specifically, the EGFP expression mediated by DCPA-based LNPs has been quantified using both fluorescence intensity measurement and flow cytometry across multiple biological replicates (Figure R28). The results confirm a statistically significant increase in EGFP expression compared to control formulations.

All correlative observations throughout the manuscript have been carefully reviewed and, where necessary, amended to ensure conclusions are stated with appropriate statistical support, clearly distinguishing correlation from causation. We appreciate your vigilance in ensuring the statistical rigor of our conclusions.

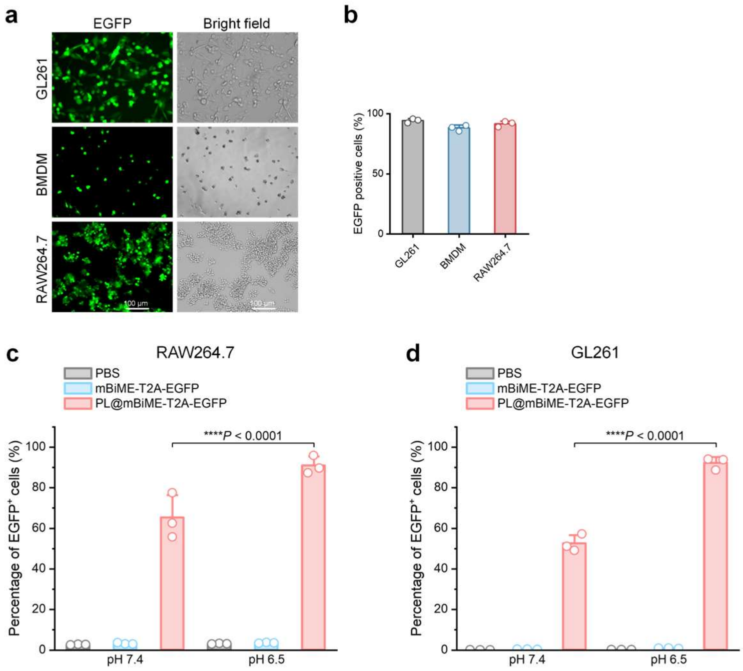

Figure R28. a Results of GL261, BMDM and RAW264.7 transfection using PL@mBiME-T2A-EGFP at pH 6.5. Scale bar = 100 μm. **b** Quantification of EGFP-positive GL261, BMDMs, and RAW264.7 cells by Image J at 24 h. Data are presented as mean ± SD, n = 3 biologically independent replicates; two-way ANOVA with Fisher's LSD post-hoc test. **c, d** Quantification of EGFP-positive RAW264.7 (Figure 2g) and GL261 cells (Supplementary Fig. 14.) by flow cytometry 24 hours after treatment with PBS, mBiME-T2A-EGFP, or PL@mBiME-T2A-EGFP. Data are presented as mean ± SD, n = 3 biologically independent replicates; two-way ANOVA with Fisher's LSD post-hoc test.

4. Discussion & Context

- Place results within broader literature, acknowledging supporting and conflicting reports. This is especially important given controversy over the importance of ErbB2 in GBM, and also regarding the choice of GL261 which is known to have multiple mechanisms of immune activation in vivo.

Response:

We appreciate this guidance and agree that two points require clearer contextualization: (a) ErbB2/HER2 expression heterogeneity and importance in GBM, and (b) immunological caveats of the GL261 model.

a) ErbB2/HER2 in GBM—heterogeneous expression and importance controversy

We agree that HER2/ErbB2 expression in GBM has been reported variably depending on detection method (mRNA vs protein), cohort composition, and scoring criteria; prior reviews explicitly note this variability/controversy[10]. At the same time, multiple

immunotherapy efforts continue to leverage HER2 as a GBM antigen (e.g., HER2-targeted cellular therapies), supporting its relevance in a subset of tumors[11, 12].
Importantly, our design does not aim to inhibit ErbB2 signaling (which would require pathway dependence); rather, ErbB2 functions here as a surface antigen/handle to increase macrophage–tumor proximity and engagement. Therefore, the key translational implication is patient stratification by ErbB2 expression level, not pathway addiction. Consistent with this, our new data directly support an expression–response relationship: higher ErbB2 expression increases macrophage–tumor conjugate formation and improves efficacy (GL261-OE-Luc > GL261-Luc > CT-2A-Luc) (Figure R19 and R20).

2b) GL261 model limitations—immunogenicity and immune activation mechanisms
We agree with the you that GL261 is relatively immunogenic and can overestimate the performance of some immunotherapies compared with human GBM. Published work highlights that GL261 differs from human GBM in immune activation features and that technical choices can substantially alter adaptive immune responses[13, 14].
To address this concern, we have expanded validation beyond GL261 by adding an additional orthotopic GBM model (CT-2A), which has been described as less immunogenic in some comparative analyses, thereby providing a more stringent context[15]. In our CT-2A model, PL@mBiME retains significant antitumor activity, although complete regression is less frequent—consistent with lower ErbB2 expression and a more challenging immune context (Figure R20).
The related description and discussion have been added in revised manuscript **Line 682-687.**

- Explicitly state study limitations, including in vitro vs. in vivo relevance and choice of models.

Response:
We thank the reviewer for this important suggestion. In the revised manuscript, we have now explicitly stated the key limitations of the study in the Discussion section, with particular attention to (i) the relevance of in vitro findings relative to in vivo tumor biology and (ii) the choice and interpretation of preclinical GBM models.
Specifically, we now discuss the immune context of the GL261 model and its relevance to immunogenic GBM, the complementary validation provided by the CT-2A model, the murine specificity of the current ErbB2-targeting BiME construct, and formulation-related considerations such as antibody orientation on the LNP surface. We also outline clear future directions, including humanized targeting strategies and validation in

patient-derived or humanized models, to address these limitations.
These additions are included in the discussion section of revise manuscript **Line 679-684 and Line 731-743** to transparently define the scope, strengths, and boundaries of the current study.

Specific Figure Comments

Figure 1

- Given that the formulation contains an ionizable lipid (SM-201, ~50%) with a pKa of ~6.5, it is unclear why pH-responsive charge reversal appears strongly dependent on DCPA (~10%). Clarify whether a zeta potential of − 5 or +5 mV is considered high for nanoparticles, especially in low ionic strength media.

Response:

We sincerely appreciate your insightful comments on our manuscript. Regarding your questions about the pH-responsive charge reversal mechanism of the formulation and the zeta potential evaluation, we clarify and supplement the relevant details as follows:

1. Effects of DCPA Incorporation on the pKa of SM102-LNPs

We employed the TNS method to determine the pKa of SM102-LNPs doped with either DSPC or DCPA, and the results demonstrated that DCPA doping elevated the pKa of SM102-LNPs from 6.41 to 6.88 (Figure R29a). Owing to the different pKa, SM102-LNPs doped with DSPC exhibit nearly neutral surface charge in the acidic tumor microenvironment (pH ~6.4), whereas DCPA-doped SM102-LNPs acquire positive charge via charge reversal. This phenomenon, consistent with a recent report in *Nat. Biomed. Eng.*[16], further validates our conclusion.

The data has been updated in revised manuscript **Fig 1k**.

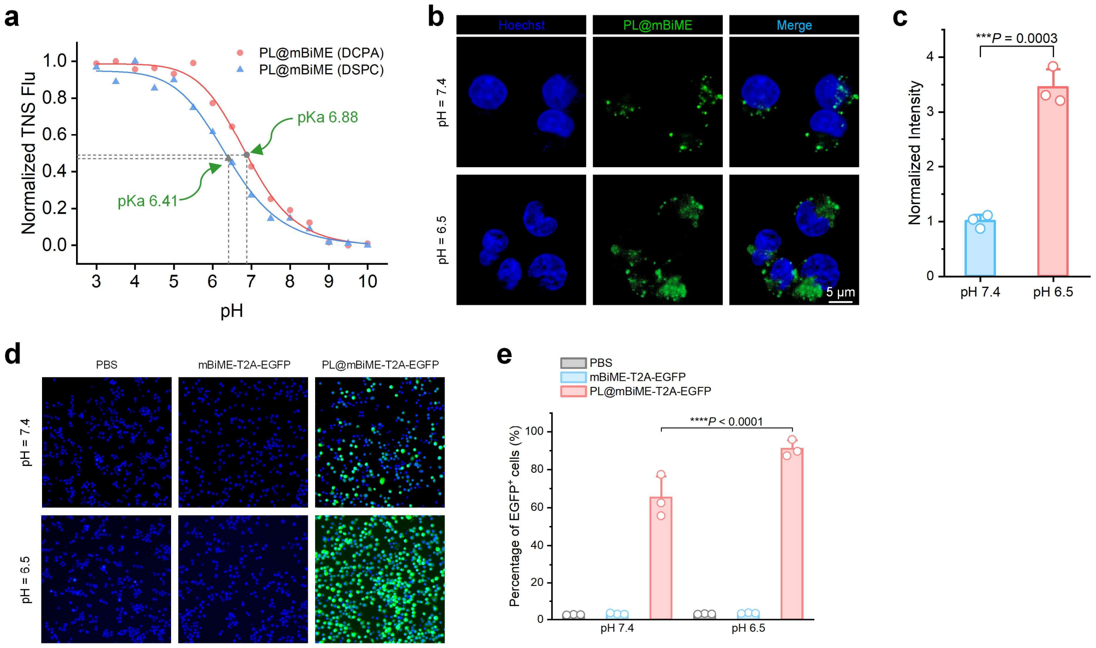

Figure R29. a The pKa determination of PL@BiME formulations containing DCPA or DSPC via the trinitrobenzenesulfonic acid (TNS) method. **b** Uptake of PL@mBiME by RAW264.7 cells at pH 7.4 or 6.5, images representative of 3 experiments. Scale bar = 5 μm. **c** Quantification of PL@mBiME uptake by RAW264.7 cells at pH 7.4 and 6.5. Corresponding quantitative analysis of the PL@mBiME fluorescence intensity from the images in (b). Data are presented as mean ± SD, n = 3 biologically independent replicates; unpaired two-tailed Student's t-test. **d** Expression of EGFP was analyzed by a fluorescence microscope after 24-h transfection of the PL@mBiME-T2A-EGFP to the RAW264.7 cells, images representative of 3 experiments. Scale bar = 100 μm. **e** Quantification of EGFP-positive RAW264.7. Data are presented as mean ± SD, n = 3 biologically independent replicates; two-way ANOVA with Fisher's LSD post-hoc test.

2. Whether the Zeta Potential change is considered high

It has been well documented in the literature that different buffer systems have a significant impact on the surface charge of nanoparticles[17, 18]. The zeta potential data in 10 mM Tris-HCl buffer (a buffer system commonly used in the LNP encapsulation process; previously measured in 10 mM PBS) have been added to the revised manuscript (**Figure 1c&j**), which demonstrates the pH-responsive charge reversal behavior of the nanoparticles.

The zeta potential increase as the pH decreased, shifting from -8.15 ± 0.82 mV at pH 7.4 to +17.65 ± 2.10 mV at pH 5.0, confirming the surface charge reversal under acid condition. This modification further confirms by our functional outcomes.

Supporting evidence:

Enhanced cellular uptake: In low-ionic-strength buffers, the extended Debye length

allows even moderate surface charges to generate meaningful electrostatic interactions with cell membranes, leading to significantly increased nanoparticle internalization (Figure R29b,c).

Higher functional delivery: LNPs with DCPA produced significantly elevated EGFP expression compared to that with DSPC, confirming that this charge magnitude is sufficient to promote productive cellular entry and mRNA release (Figure R29d,e).

The functional data validate that this zeta-potential range is indeed "high enough" to achieve efficient gene delivery.

- TEM images lack scale bars, appear very small, and are of low quality.

Response:

We sincerely apologize for the deficiencies in the transmission electron microscopy (TEM) images presented in the initial manuscript. The revised high-quality TEM images, along with the scale bars, have been updated in **Figure 1f&g** of the revised manuscript. These images clearly demonstrate the spherical morphology and size distribution of L@mBiME and PL@mBiME, which are consistent with the dynamic light scattering (DLS) results (Figure R30).

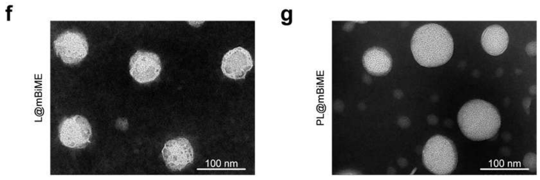

Figure R30. Representative transmission electron microscope (TEM) image of L@mBiME and PL@mBiME with N/P ratio of 4:1. Scale bar = 100nm.

- The effect of adding aPD-L1 on nanoparticle charge is not clearly demonstrated; only size changes are reported. Please include supporting data on charge measurements.

As suggested, we have supplemented the zeta potential (ζ) measurements of PL@mBiME nanoparticles before and after conjugation with aPD-L1, and the results are presented below are summarized in **Supplementary Table 3**.

- Stability studies in 10% FBS, DMEM, and PBS at 4 °C may not reflect physiologically relevant conditions. Justify this choice or consider repeating at room temperature or 37 °C.

We sincerely appreciate your thoughtful comment regarding the relevance of our stability study conditions (10% FBS, DMEM, and PBS at 4 °C) to physiological environments. We fully recognize the importance of distinguishing between storage stability and physiological stability evaluations, and we hereby provide a detailed justification for the selection of 4 °C conditions, which is closely aligned with the core

objectives of this specific stability assay and the practical translational requirements of our lipid nanoparticle (LNP) system.

The primary aim of the 4 °C stability study was to assess **storage stability**—a critical parameter for the pharmaceutical development and clinical translation of LNP formulations. In both laboratory research and industrial production, refrigerated storage (4 °C) is the standard practice for LNPs, as this temperature effectively slows down lipid oxidation, PEG chain degradation, and encapsulated mRNA leakage—all of which are major factors that compromise LNP structural integrity and biological activity over time. Evaluating stability under 4 °C conditions directly addresse the key question of whether our A2-conjugated DCPA-LNPs can maintain their physicochemical properties and functional activity during refrigerated storage, which is a prerequisite for subsequent in vivo applications (Figure R31a). This is consistent with guidelines for nanomedicine stability evaluation, where storage stability is a mandatory assessment item prior to investigating physiological stability.

In parallel, we performed stability studies in 10% FBS, DMEM, and PBS at room temperature, and found that PL@mBiME also maintains good stability under these conditions (Figure R31b).

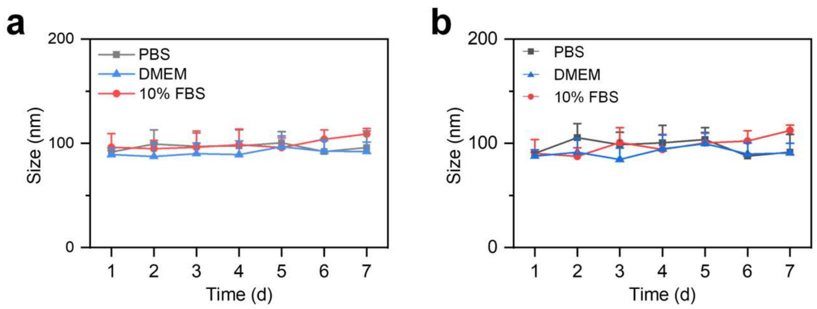

Figure R31. a Stability characteristics of PL@mBiME in PBS, DMEM, and10% FBS for 7 days at 4℃ (n = 3 independent replicates). b Stability characteristics of PL@mBiME in PBS, DMEM, and10% FBS for 7 days at room temperature (n = 3 independent replicates).

Figure 2

- Reduced colocalization of green nanoparticle signal with red LysoTracker is presented as evidence of lysosomal escape. However, if true cytosolic release occurs, the green fluorescence should appear diffuse rather than punctate. In the DCPA group at 4 h, the green signal remains vesicular, raising questions about the extent of lysosomal escape and requiring clarification.

Response:

We sincerely appreciate your critical and insightful comment regarding the interpretation of lysosomal escape evidence based on colocalization analysis and the

morphology of green nanoparticle signals. We clarify the vesicular nature of the green mRNA signal in the DCPA group at 4 h: It should be emphasized that LysoTracker is a pH-responsive probe, and the vesicular mRNA signal observed at 4 h is mainly attributed to the acidification disorder of endosomes/lysosomes induced by the protonation of ionizable lipids and DCPA. Specifically, during the endocytosis process of LNPs, the protonation of ionizable lipids and DCPA in the acidic environment of endosomes/lysosomes leads to the disruption of the original acidic balance of these compartments. This pH disorder affects the specific binding of LysoTracker to lysosomes, resulting in the observed reduced colocalization, while the mRNA still remains in the endosomal/lysosomal-derived vesicular structures at this stage. Notably, true endosomal/lysosomal escape (i.e., complete release of mRNA into the cytosol) occurs at later time points rather than at 4 h. Therefore, we have extended the observation time to 6 h, and the supplemented time-course analysis shows that the green mRNA signal in the DCPA group gradually transitions from punctate (vesicular) to diffuse (cytosolic) at 6 h (Figure R32 and R33). This confirms that the vesicular signal at 4 h is a transient state caused by acidification disorder, rather than persistent entrapment in functional lysosomes, and true endosomal/lysosomal escape is achieved at 6 h.

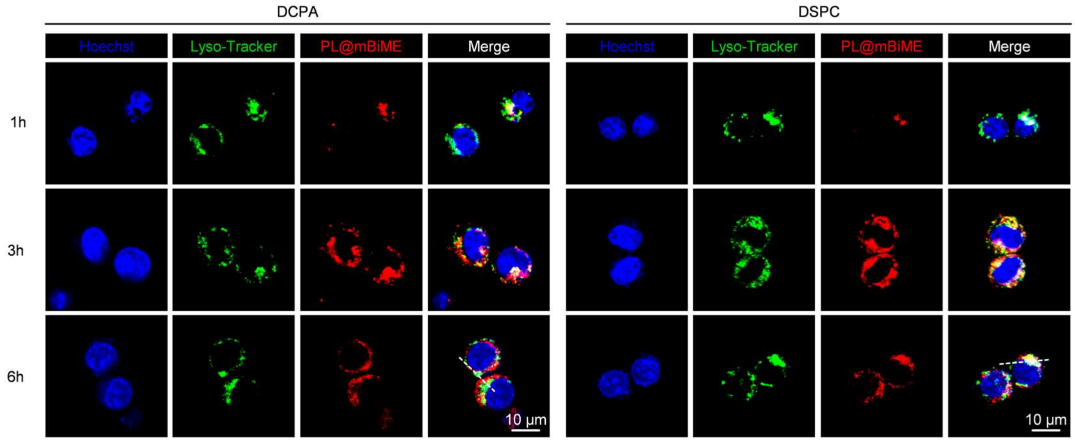

Figure R32. Representative confocal images of RAW264.7 cells incubated with PL@mBiME for 1, 3 or 6 h. Nuclei were stained with Hoechst (blue), endosomes/lysosomes were stained with Lyso-Tracker Green (green), and mRNA was labeled with Cy5 (red), images representative of 3 experiments. Scale bar = 10 μm.

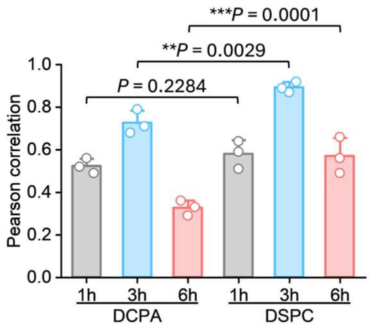

Figure R33. Pearson correlation of Lyso-Track green and Cy5-mBiME in DCPA-PL@mBiME group and DSPC-PL@mBiME group at 1h, 3h, and 6h, n = 3 biologically independent replicates. Data are presented as mean ± SD, statistical analysis was performed by two-way ANOVA with Fisher's LSD post-hoc test.

The data has been updated in revised manuscript in **Fig. 2b and Supplementary Fig. 12**.

Figure S13

- Macrophage polarization should produce distinct populations (M1 vs. M2), yet no clear separation is visible. Gating strategy and data interpretation need clarification.

Thank you for your comment regarding the macrophage polarization data. We acknowledge your observation that the flow cytometry plots do not show distinct, isolated clusters for $CD86^+$ and $CD206^+$ populations. We would like to clarify that this representation accurately reflects the biological reality of macrophage polarization which often manifests as a continuum of activation states rather than two completely discrete populations.

In our *in vitro* assays, bone marrow-derived macrophages (BMDMs) were stimulated under defined polarizing conditions (IL-4 for M2). When analyzed by flow cytometry, the expression of surface markers CD86 and CD206 was assessed on a single-cell level and plotted as a dot plot (CD206 vs. CD86). As shown in our data and supported by established literature[19, 20], macrophages frequently exhibit a spectrum of activation. Cells can co-express markers associated with both classical and alternative activation to varying degrees, resulting in a distribution across the quadrants rather than forming two entirely separate clusters.

Therefore, our gating strategy was designed to quantify the expression of CD86 and CD206 within the total macrophage population ($F4/80^+$), which effectively captures the collective shift in expression profile upon treatment.

Thank you for prompting this clarification, which has improved the precision of our data presentation.

Figure 4 (B – C)

- Comparison groups are insufficient. Using mRNA alone or antibody alone does not convincingly demonstrate the role of Angiopep-2 (A2). Direct comparison between nanoparticles with and without A2 is recommended to assess BBB transport.

Response:

We sincerely appreciate your insightful comment regarding the insufficiency of comparison groups and fully agree that direct comparison between nanoparticles with and without Angiopep-2 (A2) is essential to convincingly demonstrate the role of A2 in blood-brain barrier (BBB) transport. Notably, this concern has also been raised by another reviewer, which has further emphasized the critical importance of supplementing such comparative data to strengthen the rigor of our study.

To address this key issue comprehensively, we have performed additional in vivo experiments to monitor and compare the bio-luminescence signals of **A2-conjugated PL@mLuc** and **non-conjugated PL@mLuc** in both tumor-free brains and glioblastoma (GBM)-bearing brains. Moreover, we have extended the monitoring duration from the original time window to **144 h** to capture the long-term BBB transport efficiency and intratumoral retention characteristics of the two LNP formulations.

These supplementary data are now presented in **Figure 4f&g** of the revised manuscript. The results clearly demonstrate that A2-conjugated PL@mLuc exhibit significantly higher bio-luminescence signals in both tumor-free brain tissues and GBM lesions compared with non-conjugated controls at all time points tested, with the most prominent difference observed at 12 h post-injection (Figure R34). This finding directly confirms that the A2 peptide mediates efficient BBB crossing and enhances the accumulation of LNPs in brain tissues, especially in the GBM microenvironment.

We believe these additional comparative experiments and extended time-course data provide solid evidence to support the critical role of A2 in facilitating BBB transport of our LNP formulation.

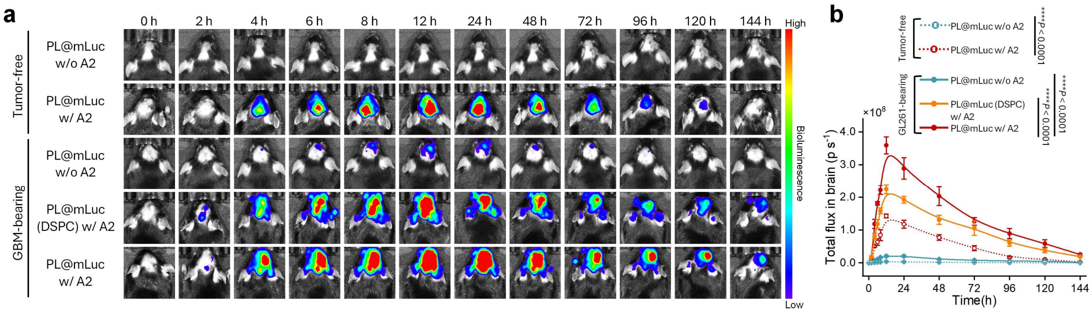

Figure R34. a, b Bioluminescence imaging of brain in tumor-free and GBM-bearing mice at 0, 2, 4, 6, 8, 12, 24, 48, 72 h, 96 h, 120h, and 144 h post-administration of various formulations, n = 3 biologically independent replicates. Data are presented as mean ± SD, statistical analysis was performed by one-way ANOVA with Fisher's LSD post-hoc test.

Figure 4 (D – K)

- Separating brain from other organs makes relative biodistribution difficult to interpret. Data should be reported as percent injected dose if possible, or at a minimum the reporting the proportion of nanoparticles reaching the brain relative to other organs would be more informative.

Response:

Regarding your comment on the interpretation of relative biodistribution data due to separating the brain from other organs: We sincerely appreciate this valuable suggestion, as we fully recognize that reporting biodistribution data without proper normalization makes it difficult to accurately evaluate the targeting efficiency of nanoparticles to the brain relative to other organs. To address this issue, we have re-analyzed our in vivo biodistribution data and supplemented normalized results in the revised manuscript (Figure R35). Specifically, we have re-calculated the biodistribution data as the **percent injected dose per gram of tissue (%ID/g)** for all tested organs (including brain, heart, liver, spleen, lungs, kidneys).

The updated results show that the A2-conjugated DCPA-LNPs exhibit a %ID/g value of 38.25 in the GL261-bearing brain at 12 h post-injection, which is 1.9-fold higher than that in the DSPC-LNPs. This provided more reliable evidence for the BBB transport and GBM targeting efficacy of the A2-conjugated DCPA-LNP system.

The revised biodistribution data, including the relative proportion data, have been updated in **Figure 4h-j** of the revised manuscript, with detailed calculation methods described in the Materials and Methods section. We hope these revisions make the biodistribution results more informative and interpretable.

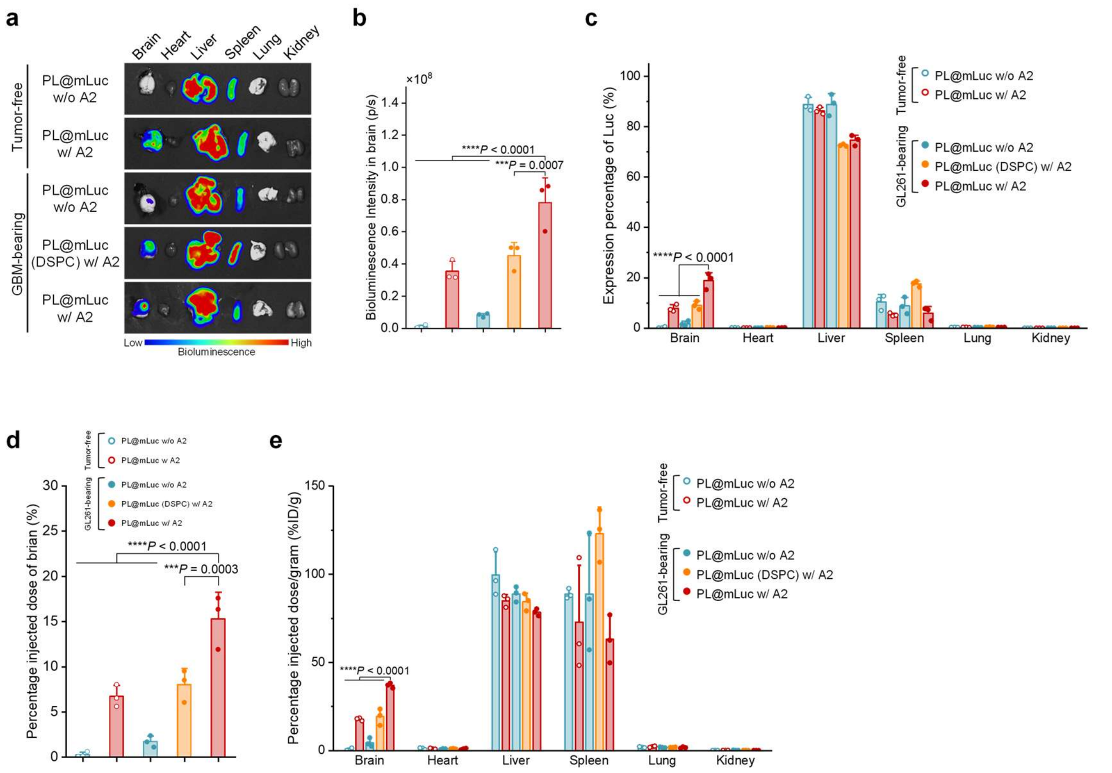

Figure R35. a Representative bioluminescence imaging of isolated brains from tumor-free versus GBM-bearing mice at 12 h post-administration of various formulations. **b** Quantitative analysis of brain bioluminescence intensity in tumor-free versus GBM-bearing mice at 12 h post-administration of various formulations, n = 3 biologically independent replicates. **c** Quantification analysis of the percentage of total bioluminescence signal attributed to major organs (brain, heart, liver, spleen, lung, kidney), n = 3 biologically independent replicates. Data are presented as mean ± SD, statistical analysis was performed by one-way ANOVA with Fisher's LSD post-hoc test. **d** Quantitative analysis of brain bioluminescence presented as percentage injected dose (%ID) in tumor-free versus GBM-bearing mice at 12 h post-administration of various formulations, n = 3 biologically independent replicates. **e** Quantification analysis of bioluminescence in major organs (brain, heart, liver, spleen, lung, kidney) expressed as percentage injected dose/gram (%ID/g). Data are presented as mean ± SD, n = 3 biologically independent replicates; one-way ANOVA with Fisher's LSD post-hoc test.

Additional Point

- Figure 5g is referenced in the text but is missing from the manuscript. This inconsistency should be addressed.

This error occurred during the final compilation of the figure file, where Figure 5g (Ki67) was combined to form the **Figure 5f**. We have revised the text of figure reference.

Reviewer #5 (Remarks to the Author):

I co-reviewed this manuscript with one of the reviewers who provided the listed reports. This is part of the Nature Communications initiative to facilitate training in peer review and to provide appropriate recognition for Early Career Researchers who co-review manuscripts.

Response:

Thank you for your note regarding the co-review of our manuscript, and we greatly appreciate the efforts you and the other reviewer have devoted to the peer review process.

We will carefully study the review reports submitted by you and the co-reviewer, and sincerely welcome any further questions, suggestions or discussions on the manuscript. We are committed to revising the manuscript meticulously and promptly in accordance with the review opinions to improve its academic quality.

References:

1. Voissière A, *et al.* The CSF-1R inhibitor pexidartinib affects FLT3-dependent DC differentiation and may antagonize durvalumab effect in patients with advanced cancers. *Science Translational Medicine* **16**, eadd1834 (2024).

2. Quail DF, *et al.* The tumor microenvironment underlies acquired resistance to CSF-1R inhibition in gliomas. *Science* **352**, aad3018 (2016).

3. Rao R, *et al.* Glioblastoma genetic drivers dictate the function of tumor-associated macrophages/microglia and responses to CSF1R inhibition. *Neuro-Oncology* **24**, 584-597 (2022).

4. Rolin C, Zimmer J, Seguin-Devaux C. Bridging the gap with multispecific immune cell engagers in cancer and infectious diseases. *Cell Mol Immunol* **21**, 643-661 (2024).

5. Fenis A, Demaria O, Gauthier L, Vivier E, Narni-Mancinelli E. New immune cell engagers for cancer immunotherapy. *Nature Reviews Immunology* **24**, 471-486 (2024).

6. Bucci L, *et al.* Bispecific T cell engager therapy for refractory rheumatoid arthritis. *Nature Medicine* **30**, 1593-1601 (2024).

7. Huang C, *et al.* Lipid Nanoparticle Delivery System for mRNA Encoding B7H3-redirected Bispecific Antibody Displays Potent Antitumor Effects on Malignant Tumors. *Adv Sci (Weinh)* **10**, e2205532 (2023).

8. Huang Y, *et al.* Organ-specific delivery of an mRNA-encoded bispecific T cell engager targeting glypican-3 in hepatocellular carcinoma. *Nat Commun* **16**, 11111 (2025).

9. Chen MZ, *et al.* A versatile antibody capture system drives specific in vivo delivery of mRNA-loaded lipid nanoparticles. *Nature Nanotechnology* **20**, 1273-1284 (2025).

10. Kim HJ, Kim DY. Present and Future of Anti-Glioblastoma Therapies: A Deep Look into Molecular Dependencies/Features. *Molecules* **25**, (2020).

11. Ahmed N, *et al.* HER2-specific T cells target primary glioblastoma stem cells and induce regression of autologous experimental tumors. *Clin Cancer Res* **16**, 474-485 (2010).

12. Li X, Zhao L, Li W, Gao P, Zhang N. HER2-targeting CAR-T cells show highly efficient anti-tumor activity against glioblastoma both in vitro and in vivo. *Genes Immun* **25**, 201-208 (2024).

13. Khalsa JK, *et al.* Immune phenotyping of diverse syngeneic murine brain tumors identifies immunologically distinct types. *Nat Commun* **11**, 3912 (2020).

14. Noffsinger B*, et al.* Technical choices significantly alter the adaptive immune response against immunocompetent murine gliomas in a model-dependent manner. *J Neurooncol* **154**, 145-157 (2021).

15. Wouters R, Bevers S, Riva M, De Smet F, Coosemans A. Immunocompetent Mouse Models in the Search for Effective Immunotherapy in Glioblastoma. *Cancers (Basel)* **13**, (2020).

16. Zhao Y*, et al.* Low reactogenicity and high tumour antigen expression from mRNA-LNPs with membrane-destabilizing zwitterionic lipids. *Nature Biomedical Engineering*, (2025).

17. Inam W*, et al.* Interactions between polymeric nanoparticles and different buffers as investigated by zeta potential measurements and molecular dynamics simulations. *VIEW* **3**, 20210009 (2022).

18. Parsons DF, Carucci C, Salis A. Buffer-specific effects arise from ionic dispersion forces. *Physical Chemistry Chemical Physics* **24**, 6544-6551 (2022).

19. Wang Q*, et al.* CD206(+)IL-4Rα(+) Macrophages Are Drivers of Adverse Cardiac Remodeling in Ischemic Cardiomyopathy. *Circulation* **152**, 257-273 (2025).

20. Song H*, et al.* Circular RNA Cdyl promotes abdominal aortic aneurysm formation by inducing M1 macrophage polarization and M1-type inflammation. *Mol Ther* **30**, 915-931 (2022).
